# Intercepting an avoided α-iminol rearrangement with a Petasis reaction for the synthesis of 2,3-diaryl substituted indoles

**DOI:** 10.1038/s42004-025-01528-9

**Published:** 2025-05-15

**Authors:** Hui-Min Zhu, Tong Lei, Zhi-Xin Liao, Jia-Chen Xiang, An-Xin Wu

**Affiliations:** 1 School of Chemistry and Chemical Engineering, Southeast University, Nanjing, 211189 PR China; 2 State Key Laboratory of Green Pesticide, International Joint Research Center for Intelligent Biosensor Technology and Health, College of Chemistry, Central China Normal University, Wuhan, 430079 PR China

**Keywords:** Synthetic chemistry methodology, Reaction mechanisms, Homogeneous catalysis, Synthetic chemistry methodology

## Abstract

Rearrangement reactions are generally considered to be a rapid and synergistic intramolecular reconstructing process that is insensitive to intermolecular intruders. We report that α-iminol rearrangements could be strategically redirected by the interception of Petasis reactions, in the context of being avoided by strong electron-withdrawing groups on the migrative aryl units. 1,4-Aryl migration prevails over 1,2-aryl migration via forming a boron-ate complex. By leveraging this reactivity, we developed a regiospecific synthesis of unsymmetrically 2,3-diaryl substituted indoles from three readily available feedstocks: an amine, an arylglyoxal, and a boronic acid. While traditional Petasis reactions with similar three-component inputs are typically applied to build C(sp^3^)-C(sp^2^) and C(sp^3^)-C(sp^3^) bonds, the present transformation offers a special opportunity for constructing a C(sp^2^)-C(sp^2^) linkage. Highly substituted indole motifs with structural diversity in the C2 position are easily accessed by this three-component reaction. A mechanism containing a copper-cobalt collaborative promotion process was suggested.

## Abstract (web summary)

2,3-Disubstituted indoles are widely present in natural products and bioactive compounds with medicinal importance, so developing an efficient synthesis of unsymmetrical 2,3-diaryl-substituted indoles remains desirable. Here, the authors report a three-component reaction of an arylamine, an arylglyoxal, and a boronic acid, achieving the selective synthesis of 2,3-diaryl substituted indoles and 2,2-diaryl substituted indolin-3-ones, by redirecting the α-iminol rearrangements via Petasis reactions.

## Introduction

2,3-Disubstituted indoles are widely present in natural products and are displayed as privileged motifs in biologically active compounds^1–4^. As a special array, unsymmetrically 2,3-diaryl substituted indoles have received sustained attention due to their medicinal importance^5–7^ and synthetic intractability^6–14^. As powerful named reactions, Fischer^6,15–17^ and Cacchi^18–25^ indole syntheses are the most versatile methods for constructing such skeletons, although their respective reactants need to be pre-synthesized. Pioneering work on transition-metal-catalyzed aniline-alkyne cyclization and their variants^26–39^, led by Larock^26–28^, Fagnou^29,30^, and Glorius^31,32^, respectively, provides efficient strategies for synthesizing 2,3-diaryl substituted indoles, despite their intrinsic difficulty in controlling the regioselectivity. In this respect, Yoshikai^40,41^ and Wan^42^ individually provided elegant options. Nevertheless, a regiospecific synthesis of unsymmetrical 2,3-diaryl-substituted indoles using noble-metal-free conditions and readily available starting materials remains valuable and desirable.

**Fig. 1:**
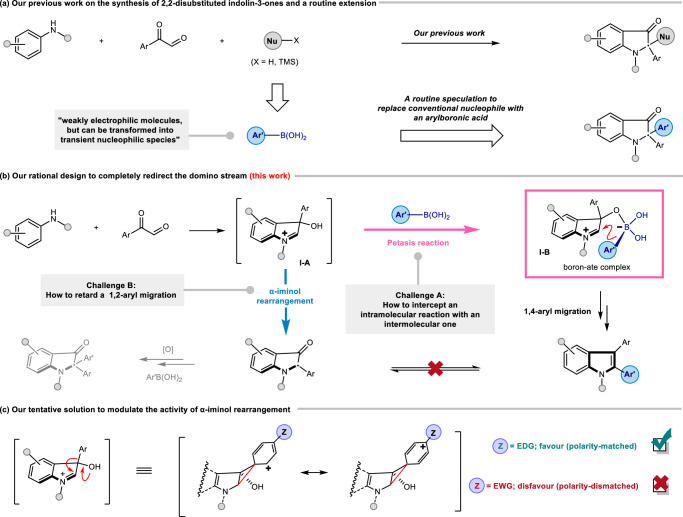
A brief introduction of our previous work and the current working hypothesis. **a** Our previous work on the synthesis of 2,2-disubstituted indolin-3-ones and a routine extension. **b** Our rational design to completely redirect the domino stream. **c** Our tentative solution to modulate the activity of α-iminol rearrangement.

Recently, we reported an aerobic copper-catalyzed synthesis of 2,2-disubstituted indolin-3-ones using an amine, an arylglyoxal, and a nucleophile^43^. As a logical extension of this diverted Mannich reaction, we anticipated that using an aryl boronic acid, a masked aryl nucleophile, would lead to the formation of 2,2-diaryl substituted indolin-3-ones based on a similar principle (Fig. 1a). However, beyond such routine extension, we predicted that it was possible to alter the domino sequence to afford a product with a completely distinct backbone utilizing identical substrates, therefore enriching the reaction diversity. Herein, we emphasize that an α-hydroxyiminium intermediate (e.g., **I-A** generated from the condensation of an amine and an arylglyoxal) is not only a precursor for α-iminol rearrangement^44–49^ but also an ideal partner for the Petasis reaction^50–62^. Then a boron-ate complex **I-B** would intercept the original pathway and redirect the transformation into a 2,3-diaryl substituted indole (Fig. 1b). It is not surprising that transformations that switch between these two classical reactions have not been reported, since such a design faces two challenges: (A) Using an intermolecular reaction to intercept an intramolecular one is kinetically difficult. (B) Tactics for intervening in the 1,2-aryl migration which belongs to the α-iminol rearrangement are still unknown, as the method for avoiding such a rapid reconstructing process is underexplored^63–67^. Inspired by Cram’s impactful study on phenonium ion theory in connection with Wagner-Meerwein rearrangements^68–71^, we assessed that the *para* substitution on the migrative aryl units affects the migratory aptitude. In general, electron-donating groups stabilize the discrete carbon cation at its *α*- and conjugated *γ*-site, thus favoring such anionotropic rearrangement^72,73^. In sharp contrast, intermediate **I-A** bearing an aryl with an electron-withdrawing *para*-substitution would have a lower migrative reactivity but a longer lifetime due to its mismatched polarity, pending the collision of boric acid (Fig. 1c). Through 1,4-aryl migration, highly substituted indole motifs could be obtained after elimination. Moreover, Petasis reaction and its variants, using amines, aldehydes, and boric acids, are employed extensively for C(sp^3^)-C(sp^2^)^56–59^ bond formation, as well as recently emerged C(sp^3^)-C(sp^3^)^60–62^ bond connections. The application of this versatile named reaction to forge a C(sp^2^)-C(sp^2^) linkage remains elusive, thus further encouraging us to verify our hypothesis. Here, we report a three-component reaction of an arylamine, an arylglyoxal, and a boronic acid, achieving the selective synthesis of 2,3-diaryl substituted indoles and 2,2-diaryl substituted indolin-3-ones. Catalytic amount of copper trifluoroacetate hydrate and Co(salen) (II) are used as the reagents. More than 50 diverse examples are presented.

## Results

### Transformation developments

Through screening a series of variables and conditions (Please see Supplementary Table 1 on page S2 for details), we found that treating three commercially available materials: bis(4-methoxyphenyl)amine (**1a**), 4-nitrophenylglyoxal (**2a**) and phenylboronic acid (**3a**) with a catalytic manifold of copper trifluoroacetate hydrate (20 mol%) and Co(salen) (II) (20 mol%) in a solution of DCE (0.1 M) at 80 °C in the air atmosphere for 4 h led to the formation of the corresponding indole product (**4a**) in 56% yield (Table 1, entry 1). These optimum conditions are recognized as our standard conditions in the following sections. Notably, the replacement of **2a** with other counterparts bearing electron-withdrawing groups, such as 4-Cl, 4-CF_3_,4-CN, failed to afford the desired product **4**, but provided **4ac** type indolin-3-one product in varying rates of yield (entry 2). Those results indicated that only *para*-NO_2_, which is the strongest electron-withdrawing group according to the Hammett constant^74^, could effectively avoid α-iminol rearrangement to match intermolecular interception under our conditions. Replacement of copper trifluoroacetate hydrate with TFA also produced **4a**, despite reducing the yield (entry 4), suggesting that the copper salt acts as a Lewis acid. Using Cobaloxime(III) instead of Co(salen) (II) could still afford **4a** in a useful yield (entry 5). More controlled experiments demonstrated that this reaction is facilitated by copper and cobalt (entries 6-8). Although the role of cobalt is still unclear, we tentatively envisage that this cobalt-Schiff base might assist in the deprotonation process during the generation of the boron-ate complex through forming Co(salen)/O_2_ adducts (Please see Supplementary Discussion on page S71 for details)^75–80^. We have also attempted to screen other additives besides cobalt, such as oxidants, bases, and additional Brønsted or Lewis acids, but have not been able to improve yields. Next, using a co-solvent of DCE and HFIP was unable to further improve the yield (entry 9), although it has been reported that HFIP can promote the Petasis process^81^. A relatively high temperature and an air atmosphere are beneficial (entries 10-11). Employing phenylboronic acid pinacol ester (PhBpin) or potassium phenyltrifluoroborate (PhBF_3_K) instead of phenylboronic acid failed to produce **4a** (entry 12). These results suggest that the reaction process may not involve a metal insertion. It is worth mentioning that the regioisomer **4ab** and indolin-3-one byproduct **4ac** were not detected throughout the screening process. We also did not detect the classical linear Petasis product **4ad** during the optimization. This may be due to the lack of the adjacent -OH group in the **2a** substrate, compared to the conventional Petasis substrates (e.g., glyoxylic acid, glycolaldehyde, and salicylaldehyde)^56–62^, therefore unable to activate the boric acid to trigger such a reaction.

**Table 1:** Screening of reaction conditions.^a^

		
Entry	Variation from ‘standard conditions’	Yield of 4 (%)^*b*^
1	none	56
2	Ar^1^/Ar^2^/Ar^3^ instead of Ar^4^	N.D
3	CuBr as a copper source	17
4	TFA instead of Cu(TFA)_2_·xH_2_O	30
5	Cobaloxime(III) instead of Co(salen)	50
6	without Co(salen)	42
7	without Cu(TFA)_2_·xH_2_O	25
8	without both Co(salen) and Cu(TFA)_2_·xH_2_O	10
9	DCE: HFIP = 5:1 as solvent	31
10	under 60 ^o^C	33
11	under N_2_	20
12	PhBpin/PhBF_3_K instead of **3a**	N.D/N.D

^*a*^Reaction conditions: **1a** (0.2 mmol), **2** (0.4 mmol, 2.0 equiv), **3a** (0.6 mmol, 3.0 equiv), Cu(TFA)_2_·xH_2_O (0.04 mmol, 0.2 equiv), Co(salen) (0.04 mmol, 0.2 equiv), DCE (2.0 mL, *c* = 0.1 M), 80 °C, 4 h, air atmosphere. ^*b*^Isolated yield. N.D. Not detected.

### Synthesis of 2,3-diaryl substituted indoles by the reaction of amine, arylglyoxal, and a boronic acid

**Fig. 2:**
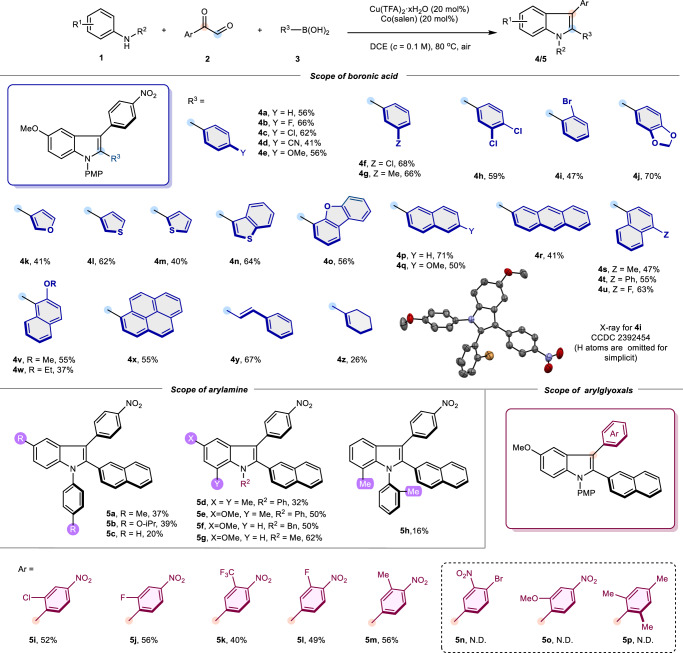
Reaction scope for the synthesis of 2,3-diaryl substituted indoles. Reaction conditions: **1** (0.2 mmol), **2** (0.4 mmol, 2.0 equiv), **3** (0.6 mmol, 3.0 equiv), Cu(TFA)_2_·xH_2_O (0.04 mmol, 0.2 equiv), Co(salen) (0.04 mmol, 0.2 equiv), DCE (2.0 mL, *c* = 0.1 M), 80 °C, air atmosphere. Isolated yield.

With the optimum conditions in hand, the scope of organoboronic acids was then examined (Fig. 2). Arylboronic acids with electron-donating substitutions (**4e,** **4** **g,** **4j**), halogens (**4b,** **4c,** **4** **f,** **4i**) on either *ortho/para/meta* position, as well as di-halogens (**4** **h**) performed good reactivity affording desired products in moderate to high yields. Electron-deficient arylboronic acids, such as 4-cyanophenyl, were also tolerated in the transformation, with a relatively low yield. These reactive tendencies are consistent with previous reports of typic Petasis reactions concomitant with an aryl migration^82^. Furthermore, heteroaryl boronic acids bearing 3-furanyl, 2- or 3-thienyl rings were suitable substrates (**4k**-**4m**). Sterically hindered heterocycles with fused-ring systems, such as 3-benzothiophene, and 4-benzo[b,d]furan proceeded well in this reaction (**4n,** **4o**). A range of boronic acids bearing polycyclic aromatic hydrocarbons, no matter in the form of C1- or C2-substituted, were well accommodated to afford indole motifs which are not easily accessed by other methods (**4p**-**4x**). To our delight, a high yield was observed when vinyl boronic acid was employed (**4** **y**). Since the olefin motif can be easily reduced into a saturated one, this result further broadens the utility of the present indole synthesis. Finally, it was found that cyclohexene-1-boronic acid could also participate in the reaction, albeit with a low yield (**4z**). The substrate scope of arylamines as well as arylglyoxals appears to be more restricted compared to that of boronic acids. In general, at least one electron-donating group is required in aryl substituents of diarylamine for obtaining a better yield (**5a, 5b, 5** **d, 5e**). When diphenylamine was used, only 20% yield of the target product was obtained (**5c**). *N*-benzyl-4-methoxyaniline and 4-methoxy-N-methylaniline afforded the corresponding *N*-benzyl (**5** **f**) and *N*-methyl (**5** **g**) indole products, respectively. When di-*o*-tolylamine was submitted into the reaction, the corresponding product was obtained in only 16% yield (**5** **h**). Moreover, after extensive testing, we found that only arylglyoxal substrates with a nitro group attached in the *para* position of the phenyl ring could fulfill the activity requirement for this transformation. Bearing halogen (-Cl, -F) or electron-withdrawing group (-CF_3_) at *ortho/meta* positions of the *p*-nitrophenyl substituent can also realize the conversions smoothly (**5i**-**5l**). Interestingly, good reactivities were obtained when the methyl group was attached to *meta* positions of the *p*-nitrophenyl (**5** **m**). In contrast, transformations were completely muted when 2-methoxy-4-nitrophenyl (**5o**) or even 3-nitro-4-bromophenyl glyoxal (**5n**) were employed. These results suggest that the electronic properties of the carbon linked to the indole C3 position are critical. If its electron density is enhanced (e.g., affected by an *ortho* methoxyl group), the α-iminol rearrangement reaction would not be avoided, thus unable to be intercepted by the Petasis reaction. Furthermore, 2,4,6-trimethyl phenyl glyoxal turned out to be an unsuitable substrate illustrating steric effects of arylglyoxal might not be crucial to avoid 1,2-aryl migration (**5p**). Finally, the structure of **4i** was determined by X-ray crystallographic analysis. Notably, the current method might have the synthetic potential to create indoles bearing a chiral C2- (**4i, 4** **v, 4w**) or C3- (**5i,** **5j**) aryl axis^25^, if a suitable asymmetric catalytic condition is finely established.

### Control experiments and mechanistic considerations

**Fig. 3:**
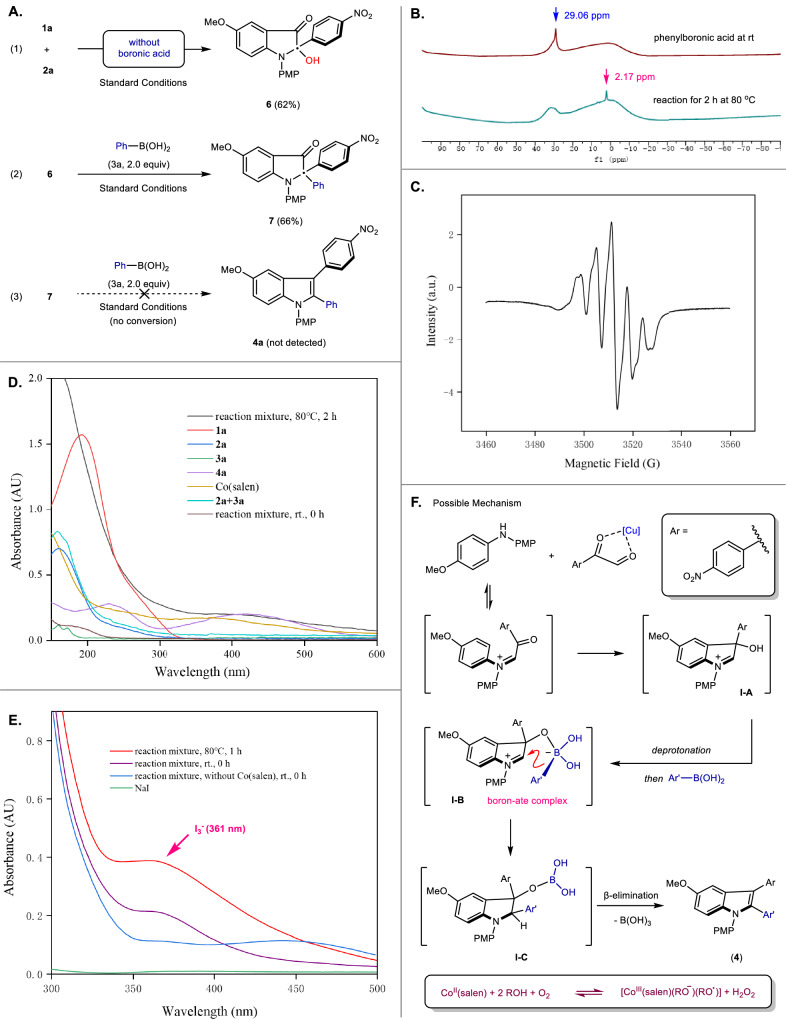
Control experiments and mechanistic considerations. **A** Control experiments. **B** ^11^B NMR spectroscopy experiments. **C** EPR experiment. **D** UV-vis absorption spectra of different reaction components. **E** Measurement of the H_2_O_2_ in the reaction mixture. **F** Possible mechanism.

Control experiments were then conducted to provide more insights into the reaction mechanism (Fig. 3A). Exposure of **1a** and **2a** under our standard conditions in the absence of phenylboronic acid (**3a**) afforded **6** in 62% yield (Fig. 3A-1). This reactivity is consistent with the experimental results reported in our previous work^43^. Next, treating **6** under the standard conditions in the presence of **3a** rendered **4ac**-type indolin-3-one **7** as a classic Petasis product^83^ in 66% yield (Fig. 3A-2). Furthermore, stirring **7** with extra phenylboronic acids (**3a**) failed to produce **4a**, indicating neither **6** nor **7** was the intermediate of our model transformation (Fig. 3A-3)^84^. These results further support our original mechanistic speculations (Fig. 1b and c). ^11^B NMR spectroscopy experiments were then carried out to further demonstrate the formation of boron-ate complex during the reaction. An obvious up-field shift (from 29.1 ppm to 2.2 ppm) indicated the tetracoordinated boronate species were likely to be generated as we expected (Fig. 3B)^85–87^. Furthermore, UV–vis absorption spectrometry of the reaction mixture indicated there was no definitive proof for the formation of the EDA complex between the boronate anion and *p*-nitrophenyl region during the formation of the boron-ate complex (**I-B**) (Fig. 3D)^88^. These preliminary experimental results indicate that the successful occurrence of this domino process is due to the strong electron-withdrawing group slowing down the α-iminol rearrangement rather than forming a strong EDA complex that accelerates the Petasis process.

To get more insights into the mechanism, an EPR experiment was performed. An obvious signal was captured when 5,5-dimethyl-1-pyrroline N-oxide (DMPO) was employed as a radical scavenger suggests that our reaction may involve the formation of radicals (Fig. 3 C). Furthermore, determined spectroscopically by the iodimetry method, H_2_O_2_ should be produced by cobalt during the reaction (Fig. 3E) which suggests a two-electron, two-proton reduction of oxygen may be involve in the reaction. Furthermore, it is reported that Petasis reaction is more favorable when it undergoes a mechanism *via* a deprotonated ate complex^89^. Indeed, our density functional theory (DFT) calculations illustrated that the process of forming boron-ate complex from a deprotonated hydroxyl group is 36.9 kcal mol^-1^ lower in energy than the process without deprotonation in this reaction (Please see Supplementary Fig. S7). According to these experimental results, a possible mechanism is suggested in Fig. 3F. The role of copper salt is to activate **1a** by coordination with the adjacent dicarbonyl group. Given that cobalt acts primarily as a reaction accelerator rather than an essential additive (Table 1, entry 6), we propose that the cobalt-oxygen synergy facilitates intermediate **I-A** deprotonation probably through in situ base generation via cobalt-mediated oxygen reduction^78–80^. We discuss these details more specifically in the Supplementary Discussion on page S71. At this stage, we are still unable to further explain or prove this mechanism, especially the role of cobalt. We hope to continue to explore the mechanism of action of this bimetallic reagent in our subsequent studies.

**Fig. 4:**
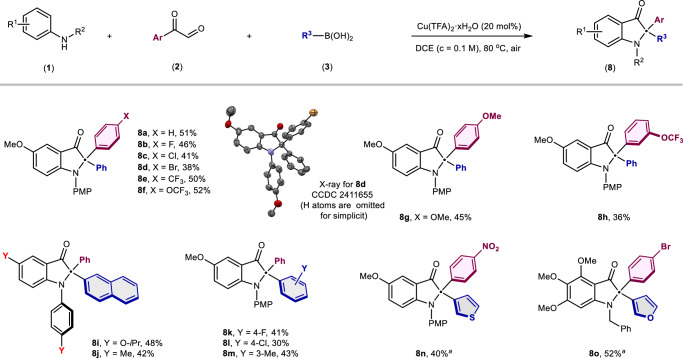
Synthesis of 2,2-diaryl substituted indolin-3-ones under modified conditions. Reaction conditions: **1** (0.2 mmol), **2** (0.4 mmol, 2.0 equiv), **3** (0.6 mmol, 3.0 equiv), Cu(TFA)_2_·xH_2_O (0.04 mmol, 0.2 equiv), DCE (2.0 mL, *c* = 0.1 M), 80 °C, air atmosphere. Isolated yield. ^*a*^Reaction conditions for one-pot two stage protocol: **1** (0.2 mmol), **2** (0.4 mmol, 2.0 equiv), Cu(TFA)_2_·xH_2_O (0.04 mmol, 0.2 equiv), DCE (2.0 mL, *c* = 0.1 M), 80 °C, air atmosphere for 4 h, then **3** (0.6 mmol, 3.0 equiv) and additional Cu(TFA)_2_·xH_2_O (0.04 mmol, 0.2 equiv) were added, then react for 8 h at 80 °C, air atmosphere.

Based on our understanding of the reaction mechanism, we further adapted the transformation using arylglyoxal bearing non-strongly electron-withdrawing groups. 2,2-Diaryl substituted indolin-3-ones were successfully obtained under the identical reaction conditions otherwise in the absence of Co(salen) (Fig. 4). 2,2-Diaryl substituted indolin-3-ones are privileged motifs but not easy to access, especially for those with neither of the two aromatic rings bearing an electron-donating group^90–93^. An elegant work was reported to synthesize this motif by Biju et al. just recently^94^. We are delighted to see that through slight adjustment of our additives, these valuable *N*-heterocyclic frameworks were obtained in a modular manner. It is noteworthy that 4-nitro-phenylglyoxals can be employed in the synthesis of the corresponding 2,2-diaryl substituted indolin-3-one (**8n**) through a one-pot, two-stage approach, whereby the arylglyoxals are allowed to react preferentially with the amine for 4 h, and then boric acid is added.

## Conclusions

In summary, we developed a regiospecific synthesis of unsymmetrically 2,3-diaryl substituted indoles by a multicomponent reaction of three easy-accessible linear substrates: an arylamine, an arylglyoxal, and a boronic acid. Facilitated by Cu(II), Co(II), and air, this transformation features operational simplicity and is suitable for a range of boronic acids bearing heteroaromatic rings, sterically hindered polyaromatic rings, as well as vinylic substitutions. A copper-cobalt additive manifold was developed to serve as an acid-base pair. This indole synthesis incorporates a Petasis process but with a rarely seen aryl-aryl bond formation, which provides an additional application scenario for this versatile named reaction. We also anticipate that our strategy using a robust intermolecular reaction to intercept an avoided intramolecular rearrangement would provide accessibility for other synthetically valuable targets, as well as expand the functionality of classical rearrangements from an updated perspective.

## Methods

In a 35 mL heavy-wall pressure tube were added 4,4′-dimethoxydiphenylamine **1a** (45.9 mg, 0.2 mmol), 4-nitrophenylglyoxal **2a** (71.7 mg, 0.4 mmol), boronic acid **3a** (73.2 mg, 0.6 mmol), Cu(TFA)_2_·xH_2_O (12.3 mg, 0.04 mmol), Co(salen) (13.1 mg, 0.04 mmol) and DCE (2.0 mL, *c* 0.1 M), and the resulting mixture was stirred at 80 °C (heating block) for 4 h until substrate conversion was almost complete by TLC analysis. After the reaction stopped and cooled at room temperature, the reaction mixture was quenched with saturated NaHCO_3_ solution (50 mL) and NaCl solution (200 mL). The mixture was then extracted with EtOAc (150 mL × 2), and the organic layers were separated and merged. The mixture was dried with anhydrous Na_2_SO_4_ and concentrated under reduced pressure. The crude product was purified by column chromatography on silica gel (200–300 mesh, eluted with PE : DCM = 3:1) to afford the product **4a**.

## Supplementary information

Transparent Peer Review file

Supplementary Information

Description of Additional Supplementary Files

Supplementary Data 1

Supplementary Data 2

## Data Availability

The X-ray crystallographic coordinates for structures reported in this Article have been deposited at the Cambridge Crystallographic Data Centre (CCDC), under deposition numbers CCDC 2392454 (Supplementary Data 1) and 2411655 (Supplementary Data 2). These data can be obtained free of charge from The Cambridge Crystallographic Data Center via https://www.ccdc.cam.ac.uk/ structures/. All other data supporting the findings of the study, including experimental procedures and compound characterization, UV–vis absorption spectrometry, DFT calculations, EPR experiments are available within the paper and the Supplementary Information. Correspondence and requests for materials should be addressed to J.C.X, all data are available from the corresponding author upon request.

## References

[1] Somei, M. & Yamada, F. Simple indole alkaloids and those with a non-rearranged monoterpenoid unit. *Nat. Prod. Rep.* **22**, 73–103 (2005). 15692618 10.1039/b316241a

[2] Ishikura, M., Abe, T., Choshi, T. & Hibino, S. Simple indole alkaloids and those with a non-rearranged monoterpenoid unit. *Nat. Prod. Rep.* **30**, 694–752 (2013). 23467716 10.1039/c3np20118j

[3] Kaushik, N. et al. Biomedical Importance of Indoles. *Molecules* **18**, 6620–6662 (2013). 23743888 10.3390/molecules18066620 PMC6270133

[4] Inman, M. & Moody, C. J. Indole Synthesis – Something Old, Something New. *Chem. Sci.* **4**, 29–41 (2013).

[5] Flynn, B. L., Hamel, E. & Jung, M. K. One-Pot Synthesis of Benzo[b]Furan and indole inhibitors of tubulin polymerization. *J. Med. Chem.* **45**, 2670–2673 (2002). 12036378 10.1021/jm020077t

[6] Hu, W. et al. Synthesis and biological evaluation of substituted 2-sulfonyl-phenyl-3-phenyl-indoles: a new series of selective COX-2 Inhibitors. *Bioorgan. Med. Chem.* **11**, 1153–1160 (2003). 10.1016/s0968-0896(03)00046-4 12628642

[7] Hu, W. et al. Discovery of 2-phenyl-3-sulfonylphenyl-indole derivatives as a new class of selective COX-2 inhibitors. *Bioorgan. Med. Chem.* **11**, 5539–5544 (2003). 10.1016/j.bmc.2003.09.008 14642598

[8] Dou, X., Yao, W., Wen, S. & Lu, Y. Facile Regiospecific Synthesis of 2,3-Disubstituted Indoles From Isatins. *Chem. Commun.* **50**, 9469–9472 (2014). 10.1039/c4cc04555f 25011926

[9] Lu, C. et al. Chemoselective metal-free indole arylation with cyclohexanones. *Org. Chem. Front.* **6**, 2738–2743 (2019).

[10] Kasiotis, K. M. & Haroutounian, S. A. 2-Pyridin-2-Yl-1H-indole derivatives: synthesis, estrogen receptor binding affinity, and photophysical properties. *Bioorg. Chem.* **34**, 1–14 (2006). 16325224 10.1016/j.bioorg.2005.10.005

[11] Li, J. et al. Palladium-catalyzed C–S bond activation and functionalization of 3-sulfenylindoles and related electron-rich heteroarenes. *Org. Chem. Front.* **4**, 1590–1594 (2017).

[12] Kraus, G. A. & Guo, H. A Flexible Synthesis of 2,3-Disubstituted Indoles From Aminobenzyl Phosphonium Salts. A Direct Synthesis of Rutaecarpine. *J. Org. Chem.* **74**, 5337–5341 (2009). 19527008 10.1021/jo900718g

[13] Maurya, R. K., Patel, O. P. S., Anand, D. & Yadav, P. P. Substrate selective synthesis of indole, tetrahydroquinoline and quinoline derivatives via intramolecular addition of hydrazones and imines. *Org. Chem. Front.* **5**, 1170–1175 (2018).

[14] Mohammadi, N., Mohaghegh, F., Ghasemi, M. & Jafarpour, F. Combining trifunctionalization of alkynoic acids, arene ortho C–H functionalization and amination: an approach to unsymmetrical 2,3-diaryl substituted indoles. *Org. Lett.* **26**, 9492–9497 (2024). 39475346 10.1021/acs.orglett.4c03446

[15] Fischer, E. & Jourdan, F. Ueber Die Hydrazine Der Brenztraubensäure. *Ber. Dtsch. Chem. Ges.* **16**, 2241–2245 (1883).

[16] Mun, H. S., Ham, W. H. & Jeong, J. H. Synthesis of 2,3-Disubstituted Indole On Solid Phase by the Fischer Indole Synthesis. *J. Comb. Chem.* **7**, 130–135 (2005). 15638492 10.1021/cc049922e

[17] Linnepe Née Köhling, P., Schmidt, A. M. & Eilbracht, P. 2,3-Disubstituted Indoles From Olefins and Hydrazines Via Tandem Hydroformylation–Fischer Indole Synthesis and Skeletal Rearrangement. *Org. Biomol. Chem.* **4**, 302–313 (2006). 16391773 10.1039/b513364e

[18] Antonio, A., Sandro, C. & Fabio, M. A Versatile Approach to 2,3-Disubstituted Indoles through the Palladium-Catalysed Cyclization of O-Alkynyltrifluoroacetanilides with Vinyl Triflates and Aryl Halides. *Tetrahedron Lett.* **33**, 3915–3918 (1992).

[19] Cacchi, S., Fabrizi, G. & Pace, P. Palladium-catalyzed cyclization of O-Alkynyltrifluoroacetanilides with allyl esters. A regioselective synthesis of 3-allylindoles. *J. Org. Chem.* **63**, 1001–1011 (1998).

[20] Arcadi, A. et al. 2-substituted 3-arylindoles through palladium-catalyzed arylative cyclization of 2-alkynyltrifluoroacetanilides with arylboronic acids under oxidative conditions. *Org. Biomol. Chem.* **11**, 545–548 (2013). 23223975 10.1039/c2ob27125g

[21] Cacchi, S. et al. 2,3-disubstituted indoles via palladium-catalyzed reaction of 2-alkynyltrifluoroacetanilides with arenediazonium tetrafluoroborates. *Org. Lett.* **12**, 3279–3281 (2010). 20568827 10.1021/ol101321g

[22] Luo, Y. et al. Palladium-Catalyzed Synthesis of 2,3-Diaryl-N-Methylindoles From Ortho-Alkynylanilines and Aryl Pinacol Boronic Esters. *Org. Lett.* **20**, 6872–6876 (2018). 30345761 10.1021/acs.orglett.8b02835

[23] Lu, B. Z. et al. A Practical Mild, One-Pot, Regiospecific Synthesis of 2,3-Disubstituted Indoles Via Consecutive Sonogashira and Cacchi Reactions. *Org. Lett.* **8**, 3271–3274 (2006). 16836383 10.1021/ol061136q

[24] Lu, B. Z. et al. One-Pot and Regiospecific Synthesis of 2,3-Disubstituted Indoles From 2-Bromoanilides Via Consecutive Palladium-Catalyzed Sonogashira Coupling, Amidopalladation, and Reductive Elimination. *J. Org. Chem.* **78**, 4558–4562 (2013). 23544431 10.1021/jo302679f

[25] He, Y. P., Wu, H., Wang, Q. & Zhu, J. Palladium-Catalyzed Enantioselective Cacchi Reaction: Asymmetric Synthesis of Axially Chiral 2,3-Disubstituted Indoles. *Angew. Chem. Int. Ed.* **59**, 2105–2109 (2020). 10.1002/anie.201914049 31756260

[26] Larock, R. C. & Yum, E. K. Synthesis of Indoles Via Palladium-Catalyzed Heteroannulation of Internal Alkynes. *J. Am. Chem. Soc.* **113**, 6689–6690 (1991).

[27] Larock, R. C., Yum, E. K. & Refvik, M. D. Synthesis of 2,3-Disubstituted Indoles Via Palladium-Catalyzed Annulation of Internal Alkynes. *J. Org. Chem.* **63**, 7652–7662 (1998).

[28] Shen, M. et al. The First Regioselective Palladium-Catalyzed Indolization of 2-Bromo- Or 2-Chloroanilines with Internal Alkynes: a New Approach to 2,3-Disubstituted Indoles. *Org. Lett.* **6**, 4129–4132 (2004). 15496116 10.1021/ol048114t

[29] Stuart, D. R., Bertrand-Laperle, M., Burgess, K. M. N. & Fagnou, K. Indole Synthesis Via Rhodium Catalyzed Oxidative Coupling of Acetanilides and Internal Alkynes. *J. Am. Chem. Soc.* **130**, 16474–16475 (2008). 19554684 10.1021/ja806955s

[30] Stuart, D. R., Alsabeh, P., Kuhn, M. & Fagnou, K. Rhodium(III)-Catalyzed Arene and Alkene C−H Bond Functionalization Leading to Indoles and Pyrroles. *J. Am. Chem. Soc.* **132**, 18326–18339 (2010). 21133376 10.1021/ja1082624

[31] Zhao, D., Shi, Z. & Glorius, F. Indole Synthesis by Rhodium(III)-Catalyzed Hydrazine-Directed C-H Activation: Redox-Neutral and Traceless by N-N Bond Cleavage. *Angew. Chem. Int. Ed.* **52**, 12426–12429 (2013). 10.1002/anie.201306098 24222579

[32] Lerchen, A., Vásquez-Céspedes, S. & Glorius, F. Cobalt(III)-Catalyzed Redox-Neutral Synthesis of Unprotected Indoles Featuring an N−N Bond Cleavage. *Angew. Chem. Int. Ed.* **55**, 3208–3211 (2016). 10.1002/anie.201510705 26836438

[33] Wang, C., Sun, H., Fang, Y. & Huang, Y. General and Efficient Synthesis of Indoles through Triazene-Directed C-H Annulation. *Angew. Chem. Int. Ed.* **52**, 5795–5798 (2013). 10.1002/anie.201301742 23606211

[34] Zhang, G., Yu, H., Qin, G. & Huang, H. Rh-Catalyzed Oxidative C–H Activation/Annulation: Converting Anilines to Indoles Using Molecular Oxygen as the Sole Oxidant. *Chem. Commun.* **50**, 4331–4334 (2014). 10.1039/c3cc49751h 24643726

[35] Hoshino, Y., Shibata, Y. & Tanaka, K. Oxidative Annulation of Anilides with Internal Alkynes Using an (Electron-Deficient η5-Cyclopentadienyl)Rhodium(III) Catalyst Under Ambient Conditions. *Adv. Synth. Catal.* **356**, 1577–1585 (2014).

[36] Zhou, Z., Liu, G., Chen, Y. & Lu, X. Rhodium(III)-Catalyzed Redox-Neutral C-H Annulation of Arylnitrones and Alkynes for the Synthesis of Indole Derivatives. *Adv. Synth. Catal.* **357**, 2944–2950 (2015).

[37] Ozkaya, B., Bub, C. L. & Patureau, F. W. Step and redox efficient nitroarene to indole synthesis. *Chem. Commun.* **56**, 13185–13188 (2020). 10.1039/d0cc03258a 33020764

[38] Kumar, A. & Tadigoppula, N. Synthesis of unprotected and highly substituted indoles by the ruthenium(II)-catalyzed reaction of phenyl isocyanates with diaryl/diheteroaryl alkynes/ethyl-3-phenyl propiolates. *Org. Lett.* **23**, 8–12 (2021). 33353305 10.1021/acs.orglett.0c02793

[39] Jagtap, P. A., Lokolkar, M. S. & Bhanage, B. M. Cu-mediated tandem 2,3-disubstituted indole synthesis from simple anilines and internal alkynes via C–H annulation. *J. Org. Chem.* **88**, 10960–10973 (2023). 37463299 10.1021/acs.joc.3c00954

[40] Wei, Y., Deb, I. & Yoshikai, N. Palladium-catalyzed aerobic oxidative cyclization of N-aryl imines: indole synthesis from anilines and ketones. *J. Am. Chem. Soc.* **134**, 9098–9101 (2012). 22612535 10.1021/ja3030824

[41] Shi, Z. & Glorius, F. Efficient and versatile synthesis of indoles from enamines and imines by cross-dehydrogenative coupling. *Angew. Chem. Int. Ed.* **51**, 9220–9222 (2012). 10.1002/anie.201205079 22903775

[42] Yan, H. et al. Rhodium-Catalyzed C-H Annulation of Nitrones with Alkynes: A Regiospecific Route to Unsymmetrical 2,3-Diaryl-Substituted Indoles. *Angew. Chem. Int. Ed.* **54**, 10613–10617 (2015). 10.1002/anie.201503997 26177605

[43] Xiang, J. et al. Diverting the Mannich Reaction to Access 2,2-Disubstituted Indolin-3-Ones by Merging 1,2-Aryl Migration and Copper-Catalyzed Aerobic Oxidation. *Org. Chem. Front.* **11**, 3186–3195 (2024).

[44] Shoppee, C. W. & Prins, D. A. Ü. ber Bestandteile Der Nebennierenrinde Und Verwandte Stoffe. 57. Mitteilung. 17-Oxy-20-Keto-Steroide Und Der Mechanismus Ihrer Umlagerung Zu Polyhydrochrysen-Derivaten. *Helv. Chim. Acta* **26**, 185–200 (1943).

[45] Paquette, L. A. & Hofferberth, J. E. The α-Hydroxy Ketone (α-Ketol) and Related Rearrangements. *Org. React.* **62**, 477–567 (2003).

[46] Compain, P., Goré, J. & Vatèle, J. Rearrangement of α-Hydroxy Imines to α-Amino Ketones: Mechanistic Aspects and Synthetic Applications. *Tetrahedron* **52**, 6647–6664 (1996).

[47] Zhang, Z., Ren, Z., Wang, Y. & Guan, Z. Cu(TFA)_2_-Catalyzed Oxidative Tandem Cyclization/1,2-Alkyl Migration of Enamino Amides for Synthesis of Pyrrolin-4-Ones. *Org. Lett.* **15**, 4822–4825 (2013). 23988157 10.1021/ol4022222

[48] Zhang, X., Staples, R. J., Rheingold, A. L. & Wulff, W. D. Catalytic Asymmetric α-Iminol Rearrangement: New Chiral Platforms. *J. Am. Chem. Soc.* **136**, 13971–13974 (2014). 25247674 10.1021/ja5065685 PMC4195388

[49] Das, R. C. et al. N‑Heterocyclic Carbene-Catalyzed Imine Umpolung/Semipinacol Rearrangement Cascade for the Synthesis of Indoxyls. *ACS Catal.* **14**, 4202–4210 (2024).

[50] Petasis, N. A. & Akritopoulou, I. The Boronic Acid Mannich Reaction: A New Method for the Synthesis of Geometrically Pure Allylamines. *Tetrahedron Lett.* **34**, 583–586 (1993).

[51] Petasis, N. A., Goodman, A. & Zavialov, I. A. A New Synthesis of α-Arylglycines From Aryl Boronic Acids. *Tetrahedron* **53**, 16463–16470 (1997).

[52] Petasis, N. A. & Patel, Z. D. Synthesis of Piperazinones and Benzopiperazinones From 1,2-Diamines and Organoboronic Acids. *Tetrahedron Lett.* **41**, 9607–9611 (2000).

[53] Petasis, N. A. & Boral, S. One-Step Three-Component Reaction Among Organoboronic Acids, Amines and Salicylaldehydes. *Tetrahedron Lett.* **42**, 539–542 (2001).

[54] Petasis, N. A. & Zavialov, I. A. A New and Practical Synthesis of α-Amino Acids From Alkenyl Boronic Acids. *J. Am. Chem. Soc.* **119**, 445–446 (1997).

[55] Petasis, N. A. & Zavialov, I. A. Highly Stereocontrolled One-Step Synthesis of Anti-β-Amino Alcohols From Organoboronic Acids, Amines, and α-Hydroxy Aldehydes. *J. Am. Chem. Soc.* **120**, 11798–11799 (1998).

[56] Candeias, N. R., Montalbano, F., Cal, P. M. S. D. & Gois, P. M. P. Boronic Acids and Esters in the Petasis-Borono Mannich Multicomponent Reaction. *Chem. Rev.* **110**, 6169–6193 (2010). 20677749 10.1021/cr100108k

[57] Wu, P., Givskov, M. & Nielsen, T. E. Reactivity and Synthetic Applications of Multicomponent Petasis Reactions. *Chem. Rev.* **119**, 11245–11290 (2019). 31454230 10.1021/acs.chemrev.9b00214 PMC6813545

[58] Yamaoka, Y., Miyabe, H. & Takemoto, Y. Catalytic Enantioselective Petasis-Type Reaction of Quinolines Catalyzed by a Newly Designed Thiourea Catalyst. *J. Am. Chem. Soc.* **129**, 6686–6687 (2007). 17488015 10.1021/ja071470x

[59] Jiang, Y. & Schaus, S. E. Asymmetric Petasis Borono-Mannich Allylation Reactions Catalyzed by Chiral Biphenols. *Angew. Chem. Int. Ed.* **56**, 1544–1548 (2017). 10.1002/anie.201611332 PMC5716625 28052567

[60] Hu, C. et al. A General Three-Component Alkyl Petasis Boron–Mannich Reaction. *J. Am. Chem. Soc.* **146**, 21769–21777 (2024). 39072677 10.1021/jacs.4c05940 PMC11815978

[61] Yi, J., Badir, S. O., Alam, R. & Molander, G. A. Photoredox-Catalyzed Multicomponent Petasis Reaction with Alkyltrifluoroborates. *Org. Lett.* **21**, 4853–4858 (2019). 31145628 10.1021/acs.orglett.9b01747 PMC7086342

[62] Oliva, M. et al. Photoredox-Catalyzed Multicomponent Petasis Reaction in Batch and Continuous Flow with Alkyl Boronic Acids. *Iscience* **24**, 103134 (2021). 34632333 10.1016/j.isci.2021.103134 PMC8487034

[63] Sun, K., Liu, S., Bec, P. M. & Driver, T. G. Rhodium-Catalyzed Synthesis of 2,3-Disubstituted Indoles From β,β-Disubstituted Stryryl Azides. *Angew. Chem. Int. Ed.* **50**, 1702–1706 (2011). 10.1002/anie.201006917 PMC3154515 21308937

[64] Wu, P., Wu, K., Wang, L. & Yu, Z. Iron-Promoted Difunctionalization of Alkenes by Phenylselenylation/1,2-Aryl Migration. *Org. Lett.* **19**, 5450–5453 (2017). 28937223 10.1021/acs.orglett.7b02751

[65] He, Y., Wu, H., Wang, Q. & Zhu, J. Catalytic Enantioselective Synthesis of Morpholinones Enabled by Aza-Benzilic Ester Rearrangement. *J. Am. Chem. Soc.* **143**, 7320–7325 (2021). 33955753 10.1021/jacs.1c03915

[66] He, Y., Quan, R., Li, X., Zhu, J. & Wu, H. Asymmetric Construction of α,α-Disubstituted Piperazinones Enabled by Benzilic Amide Rearrangement. *Angew. Chem. Int. Ed.* **62**, e202217954 (2023). 10.1002/anie.202217954 36869401

[67] Li, X. Z., He, Y. P. & Wu, H. Zinc Chloride-Catalyzed Cyclizative 1,2-Rearrangement Enables Facile Access to Morpholinones Bearing Aza-Quaternary Carbons. *Commun. Chem.* **6**, 216 (2023). 37805578 10.1038/s42004-023-01016-y PMC10560277

[68] Cram, D. J. Phenonium Ions as Discrete Intermediates in Certain Wagner-Meerwein Rearrangements. *J. Am. Chem. Soc.* **86**, 3767–3772 (1964).

[69] Cram, D. J. Studies in Stereochemistry. I. The Stereospecific Wagner-Meerwein Rearrangement of the Isomers of 3-Phenyl-2-Butanol. *J. Am. Chem. Soc.* **71**, 3863–3870 (1949).

[70] Cram, D. J. & Davis, R. Studies in Stereochemistry. II. The Preparation and Complete Resolution of 3-Phenyl-2-Pentanol and 2-Phenyl-3-Pentanol. *J. Am. Chem. Soc.* **71**, 3871–3875 (1949).

[71] Cram, D. J. Studies in Stereochemistry. III. The Wagner-Meerwein Rearrangement in the 2-Phenyl-3-Pentanol and 3-Phenyl-2-Pentanol Systems. *J. Am. Chem. Soc.* **71**, 3875–3883 (1949).

[72] Wu, H., Wang, Q. & Zhu, J. Recent advances in catalytic enantioselective rearrangement. *Eur. J. Org. Chem.* **2019**, 1964–1980 (2019).

[73] Crone, B. & Kirsch, S. F. 1,2-alkyl migration as a key element in the invention of cascade reactions catalyzed by ∏-Acids. *Chem. Eur. J.* **14**, 3514–3522 (2008). 18318026 10.1002/chem.200701985

[74] Hansch, C., Leo, A. & Taft, R. W. A survey of Hammett substituent constants and resonance and field parameters. *Chem. Rev.* **91**, 165–195 (1991).

[75] Bozell, J. J., Hames, B. R. & Dimmel, D. R. Cobalt-Schiff base complex catalyzed oxidation of para-substituted phenolics. Preparation of benzoquinones. *J. Org. Chem.* **60**, 2398–2404 (1995).

[76] Kholdeeva, O. A. & Zalomaeva, O. V. Recent advances in transition-metal-catalyzed selective oxidation of substituted phenols and methoxyarenes with environmentally benign oxidants. *Coord. Chem. Rev.* **306**, 302–330 (2016).

[77] Canevali, C. et al. Oxidative degradation of monomeric and dimeric phenylpropanoids: reactivity and mechanistic investigation, *J. Chem. Soc. Dalton Trans*. 3007–3014 (2002).

[78] Vinck, E., Murphy, D. M., Fallis, I. A., Strevens, R. R. & Doorslaer, S. V. Formation of a Cobalt(III)−phenoxyl radical complex by acetic acid promoted aerobic oxidation of a Co(II)salen complex. *Inorg. Chem.* **49**, 2083–2092 (2010). 20121216 10.1021/ic901849e

[79] Reiss, H. et al. Cobalt(II)[salen]-catalyzed selective aerobic oxidative cross-coupling between electron-rich phenols and 2-naphthols. *J. Org. Chem.* **84**, 7950–7960 (2019). 10.1021/acs.joc.9b00822 31064184

[80] Bolzacchini, E. et al. Spectromagnetic investigation of the active species in the oxidation of propenoidic phenols catalysed by [N,N[prime or minute]-bis(salicylidene)ethane-1,2-diaminato]cobalt- (II). *J. Chem. Soc., Dalton Trans*. 4695–4700 (1997).

[81] Nanda, K. K. & Wesley Trotter, B. Diastereoselective petasis mannich reactions accelerated by hexafluoroisopropanol: a pyrrolidine-derived arylglycine synthesis. *Tetrahedron Lett.* **46**, 2025–2028 (2005).

[82] Beisel, T. & Manolikakes, G. A Lewis acid palladium(II)-catalyzed three-component synthesis of α-substituted amides. *Org. Lett.* **15**, 6046–6049 (2013). 24206118 10.1021/ol402949t

[83] Rand, A. W., Gonzalez, K. J., Reimann, C. E., Virgil, S. C. & Stoltz, B. M. Total synthesis of strempeliopidine and non-natural stereoisomers through a convergent petasis Borono–Mannich Reaction. *J. Am. Chem. Soc.* **145**, 7278–7287 (2023). 36952571 10.1021/jacs.2c13146 PMC10281614

[84] Zhang, J. L., Chen, J. X., Ding, J. C., Liu, M. C. & Wu, H. Y. Copper-catalyzed arylation of indolin-2,3-ones with arylboronic acids. *Tetrahedron* **67**, 9347–9351 (2011).

[85] Hermanek, S. Boron-11 NMR spectra of boranes, main-group heteroboranes, and substituted derivatives. Factors influencing chemical shifts of skeletal atoms. *Chem. Rev.* **92**, 325–362 (1992).

[86] Sumida, Y., Kato, T. & Hosoya, T. Generation of arynes via Ate complexes of arylboronic esters with an ortho-leaving group. *Org. Lett.* **15**, 2806–2809 (2013). 23687992 10.1021/ol401140d

[87] Shaff, A. B., Yang, L. X., Lee, M. T. & Lalic, G. Stereospecific and regioselective synthesis of E‑allylic alcohols through reductive cross coupling of terminal alkynes. *J. Am. Chem. Soc.* **145**, 24615–24624 (2023). 10.1021/jacs.3c06963 37917569

[88] Lu, J. et al. Photoinduced Electron Donor Acceptor Complex-Enabled α-C(Sp^3^)-H Alkenylation of Amines. *Angew. Chem. Int. Ed*. e202409310 (2024). 10.1002/anie.202409310 39001611

[89] Candeias, N. R. et al. Water as the reaction medium for multicomponent reactions based on boronic acids. *Tetrahedron* **66**, 2736–2745 (2010).

[90] Liu, Y. & McWhorter, W. W. Study of the Addition of Grignard Reagents to 2-Aryl-3H-Indol-3-Ones. *J. Org. Chem.* **68**, 2618–2622 (2003). 12662030 10.1021/jo020715f

[91] Okuma, K., Matsunaga, N., Nagahora, N., Shioji, K. & Yokomori, Y. Reaction of arynes with amino acid esters. *Chem. Commun.* **47**, 5822–5824 (2011). 10.1039/c1cc11234a 21487614

[92] Mandal, T., Chakraborti, G., Maiti, S. & Dash, J. Domino grignard addition and oxidation for the one-pot synthesis of C2-Quaternary 2-Hydroxyindoxyls. *Org. Lett.* **21**, 8044–8048 (2019). 31524398 10.1021/acs.orglett.9b03022

[93] Xu, H., Ye, M., Yang, K. & Song, Q. Regioselective cross-coupling of isatogens with boronic acids to construct 2,2-disubstituted indolin-3-one derivatives. *Org. Lett.* **23**, 7776–7780 (2021). 34617759 10.1021/acs.orglett.1c02808

[94] Das, R. C. et al. N-heterocyclic carbene-catalyzed imine umpolung/semipinacol rearrangement cascade for the synthesis of indoxyls. *Acs Catal.* **14**, 4202–4210 (2024).

