# Supplementary material for: Intercepting an avoided α-iminol rearrangement with a Petasis reaction for the synthesis of 2,3-diaryl substituted indoles
Source: Commun Chem. 2025 May 15;8:152. doi: 10.1038/s42004-025-01528-9 (PMC12081858; doi:10.1038/s42004-025-01528-9)
Supplement: Supplementary file 2 — Supplementary Information [file 42004_2025_1528_MOESM2_ESM.pdf]

Supporting Information

Intercepting an avoided α-iminol rearrangement with a Petasis reaction for the synthesis of 2,3-diaryl substituted indoles

Hui-Min Zhu,[1] Tong Lei,[1] Zhixin Liao,[1] Jia-Chen Xiang,[1]* and An-Xin Wu.[2]*

[1]*School of Chemistry and Chemical Engineering, Southeast University, Nanjing 211189, P.R. China. Email: xiangjiachen@seu.edu.cn.*

[2]*State Key Laboratory of Green Pesticide, International Joint Research Center for Intelligent Biosensor Technology and Health, College of Chemistry, Central China Normal University, Wuhan 430079, P.R. China. Email: chwuax@mail.ccnu.edu.cn.*

Table of Contents

1. General Information 1

2. Optimization of the reaction conditions 2

3. General procedure 3

4. Synthesis and characterization data of compounds 7

5. ^{11}B NMR spectroscopy experiments 52

6. EPR experiment 54

7. UV-vis absorption spectra of different components 55

8. Measurement of the H_2O_2 in the reaction mixture 56

9. Computational Details 57

10. Discussion of the possible mechanistic details 71

11. Copies of the ^{1}H, ^{13}C and ^{19}F spectra 73

12. Crystallographic data and molecular structure of 4i and 8d 134

1. General Information

NMR spectra were recorded on Bruker AVANCE III HD 600MHz. Chemical shifts (*δ*) were reported in parts per million (ppm) relative to residual solvent peaks rounded to the nearest 0.01 for proton and 0.1 for carbon (*ref: $CDCl_3$[^{1}H: 7.26, ^{13}C: 77.16]*, *DMSO-d_6 [^{1}H: 2.5,3.3, ^{13}C:39.52]*). Coupling constants (*J*) were reported in Hz to the nearest 0.1 Hz. Multiplicities are abbreviated as follows: singlet (s), doublet (d), triplet (t), quartet (q), doublet-doublet (dd), quintet (quint), sextet (sext), septet (sept), multiplet (m), and broad (b). High-resolution mass spectrometry (HRMS) data was obtained on Thermo Scientific Q Exactive instrument (ESI or APCI source, mass analyzer type is orbitrap).

Materials and Methods: Unless otherwise stated, starting materials were purchased from commercial sources. Solvents were purchased in HPLC quality. Reactions were monitored by thin layer chromatography (TLC). Compounds were visualized by UV-light at 254 nm and 365 nm and by dipping the plates in a phosphomolybdic acid ethanol solution followed by heating. Flash column chromatography was performed over silica gel (300-400 mesh). The $CDCl_3$ used in the NMR experiments was stored over anhydrous K_2CO_3 before use.

2. Optimization of the reaction conditions

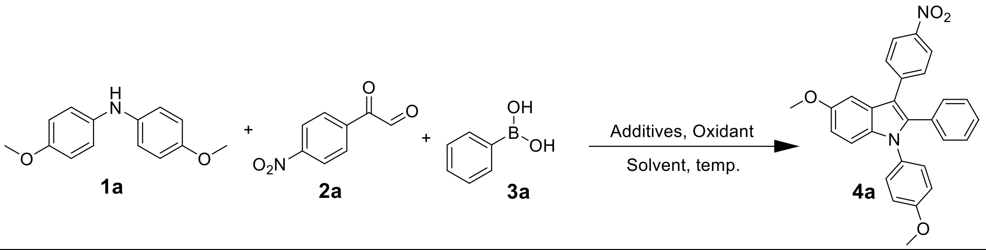

Entry	Additives (equiv)	Oxidant (equiv)	Solvent	T(°C)	Atmosphere	Yield%
1	CuBr (0.2)	CoPyCl (0.2)	DCE	80	air	17
2	CuCl (0.2)	CoPyCl (0.2)	DCE	80	air	trace
3	$Cu(acac)_2$ (0.2)	CoPyCl (0.2)	DCE	80	air	trace
4	$Cu(hfac)_2$ (0.2)	CoPyCl (0.2)	DCE	80	air	40
5	Cu $(CF_3SO_3)_2$ (0.2)	CoPyCl (0.2)	DCE	80	air	trace
6	$Cu(TFA)_2{\cdot}xH_2O$ (0.2)	CoPyCl (0.2)	DCE	80	air	50
7	TFA (0.2)	CoPyCl (0.2)	DCE	80	air	30
8	TFAA (0.2)	CoPyCl (0.2)	DCE	80	air	27
9	$Cu(TFA)_2{\cdot}xH_2O$(0.2)	Co(salen) (0.2)	DCE	80	air	56
10	$Cu(TFA)_2{\cdot}xH_2O$(0.2)	DDQ (0.2)	DCE	80	air	trace
11	$Cu(TFA)_2{\cdot}xH_2O$(0.2)	$Na_2S_2O_8$ (0.2)	DCE	80	air	trace
12	$Cu(TFA)_2{\cdot}xH_2O$(0.2)	/	DCE	80	air	42
13	/	Co(salen) (0.2)	DCE	80	air	25
14	/	/	DCE	80	air	10
15	$Cu(TFA)_2{\cdot}xH_2O$ (0.2)	Co(salen) (0.2)	DCE	80	N_2	20
16	$Cu(TFA)_2{\cdot}xH_2O$ (0.2)	Co(salen) (0.2)	DCE+HFIP	80	air	31
17	$Cu(TFA)_2{\cdot}xH_2O$ (0.2)	Co(salen) (0.2)	DCE	60	air	33

Table S1. **1a** (0.2 mmol), **2a** (0.4 mmol), **3a** (0.6 mmol), additives (0.04 mmol), oxidant (0.04 mmol) and solvent (2.0 mL, *c* 0.1 M) were sealed in a heavy-wall pressure vessel and heated with stirring at 80 °C for 4 h.

CoPyCl = Chlorobis(dimethylglyoximato)(pyridine)cobalt(III)

Co(salen) = N,N’-Disalicylidene-ethylenediamine cobalt(II) salt

3. General procedure

3.1 General procedure for the synthesis of 9

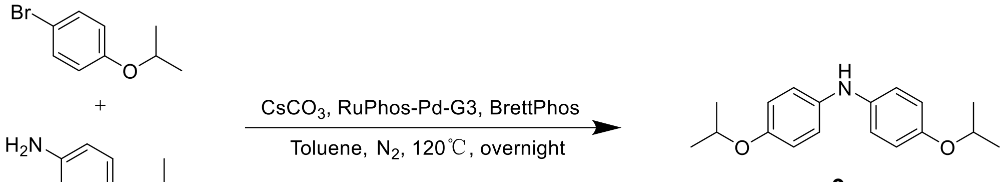

9

1-bromo-4-isopropoxybenzene (860 mg, 4 mmoL), 4-isopropoxyaniline (665 μL, 4.4 mmoL), $CsCO_3$ (1.9 g, 6 mmoL), RuPhos-Pd-G3 (8.8 mg, 2.6%) and BrettPhos (7 mg, 3.3%) was added to the reaction flask, anhydrous and oxygen-free toluene (5 mL) was added under N_2 atmosphere, and the reaction was warmed up to 120 °C overnight. After the reaction was completed, the mixture was filtered through a Celite pad and concentrated under reduced pressure. The crude product was purified by flash column chromatography on silica gel (eluted with PE : DCM = 3:1) to obtain compound **9**.

3.2 General procedure for the synthesis of 2

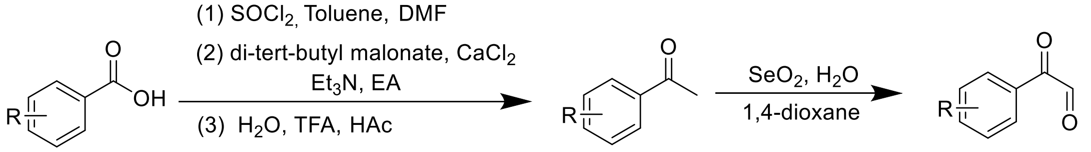

2

Take 2-(3-fluoro-4-nitrophenyl)-2-oxoacetaldehyde as an example. To a solution containing 3-fluoro-4-nitrobenzoic acid (2.8 g, 15 mmol), DMF (150 μL) and toluene (15 mL) was added thionyl chloride (1.6 mL, 22.5 mmol) dropwise with stirring at 60 ℃, then the mixture was heated up to 110 ℃ and refluxed for 4 h. After completion of the reaction, the mixture was cooled down to room temperature, then the excess sulfoxide chloride and toluene were concentrated under reduced pressure and used directly in the next step of the reaction.

To the mixture from the previous step was added di-tert-butyl malonate (3.4 mL, 15 mmol), anhydrous calcium chloride (750 mg), ethyl acetate (15 mL) and triethylamine (3 mL). The reaction was carried out at 60 °C for 2 h. The organic layer was extracted with

ethyl acetate, dried over Na_2SO_4, filtered and concentrated under reduced pressure. The resulting mixture was used in the next step.

To the reaction solution obtained in the previous step, H_2O (15 mL), TFA (300 μL) and HAc (6 mL) were added. the reaction was heated to 105 °C and refluxed for 4 h. The pH was adjusted to neutral with NaOH. The organic layer was extracted with ethyl acetate, dried over Na_2SO_4, filtered and concentrated under reduced pressure. The crude product was purified by flash column chromatography on silica gel to obtain acetophenone.

To a 50 ml round bottom flask was added acetophenone obtained in the previous step, SeO_2 (1.1g, 10 mmoL), H_2O (160 μL) and 1,4-dioxane (3.2 mL). The reaction was heated up to 105 °C and refluxed for 24 h. After the completion of the reaction, the mixture was filtered through a Celite pad and concentrated under reduced pressure. The crude product was recrystallised in hot water (80 °C) to obtain compound **2**.

3.3 General procedure for the synthesis of 4 and 5

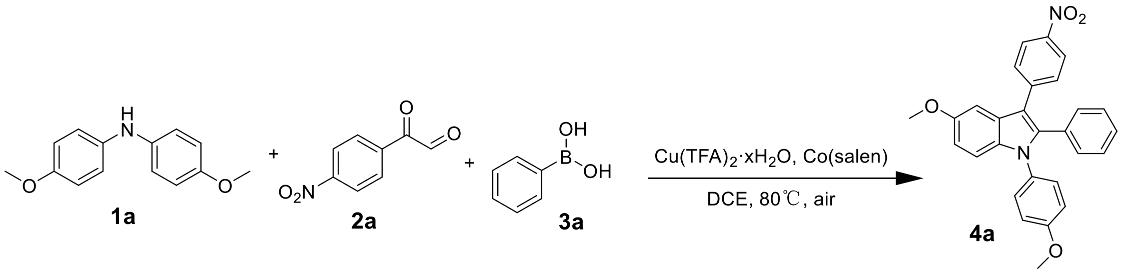

Take the synthesis of **4a** as an example, **1a** (0.2 mmol), **2a** (0.4 mmol), **3a** (0.6 mmol), $Cu(TFA)_2 \cdot xH_2O$ (0.04 mmol), Co(salen) (0.04 mmol) and DCE (2.0 mL, *c* 0.1 M) were sealed in a heavy-wall pressure vessel and heated with stirring at 80 °C for 4 h. The reaction was cooled to room temperature and extracted with ethyl acetate. The organic layer was washed sequentially with $NaHCO_3$ saturated solution and NaCl saturated solution, dried over Na_2SO_4, filtered and concentrated under reduced pressure. The crude product was purified by flash column chromatography on silica gel (eluted with PE : DCM = 3:1) to obtain compound **4a**.

3.4 General procedure for the synthesis of 6

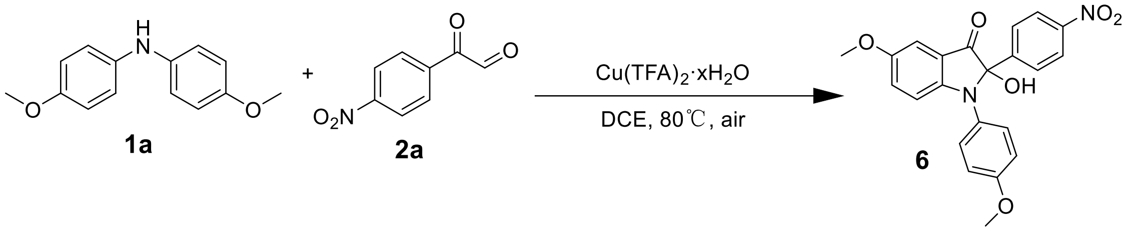

1a (0.2 mmol), **2a** (0.4 mmol), Cu(TFA)$_2$·xH$_2$O (0.04 mmol) and DCE (2.0 mL, *c* 0.1 M) were sealed in a heavy-wall pressure vessel and heated with stirring at 80 °C for 4 h. The reaction was cooled to room temperature and extracted with ethyl acetate. The organic layer was washed sequentially with $NaHCO_3$ saturated solution and NaCl saturated solution, dried over Na_2SO_4, filtered and concentrated under reduced pressure. The crude product was purified by flash column chromatography on silica gel (eluted with PE : EA = 9:1) to obtain compound **6**.

3.5 General procedure for the synthesis of 7

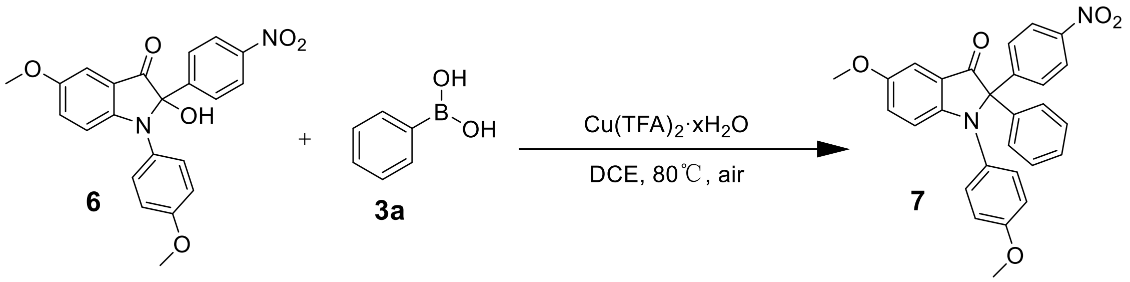

6 (0.1 mmol), **3a** (0.2 mmol), Cu(TFA)$_2$·xH$_2$O (0.02 mmol) and DCE (1.0 mL, *c* 0.1 M) were sealed in a heavy-wall pressure vessel and heated with stirring at 80 °C for 24 h. The reaction was cooled to room temperature and extracted with ethyl acetate. The organic layer was washed sequentially with $NaHCO_3$ saturated solution and NaCl saturated solution, dried over Na_2SO_4, filtered and concentrated under reduced pressure. The crude product was purified by flash column chromatography on silica gel (eluted with PE : DCM = 3:1) to obtain compound **7**.

3.6 General procedure for the synthesis of 8a - 8m

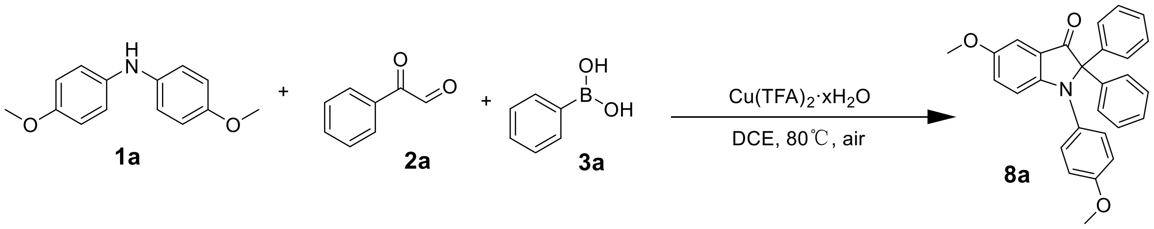

Take the synthesis of **8a** as an example, 1a (0.2 mmol), 2a (0.4 mmol), 3a (0.6 mmol), $Cu(TFA)_2 \cdot xH_2O$ (0.04 mmol) and DCE (2.0 mL, *c* 0.1 M) were sealed in a heavy-wall pressure vessel and heated with stirring at 80 °C for 4 h. The reaction was cooled to room temperature and extracted with ethyl acetate. The organic layer was washed sequentially with $NaHCO_3$ saturated solution and NaCl saturated solution, dried over Na_2SO_4, filtered and concentrated under reduced pressure. The crude product was purified by flash column chromatography on silica gel (eluted with PE : DCM = 3:1) to obtain compound **8a**.

3.7 General procedure for the synthesis of 8n and 8o

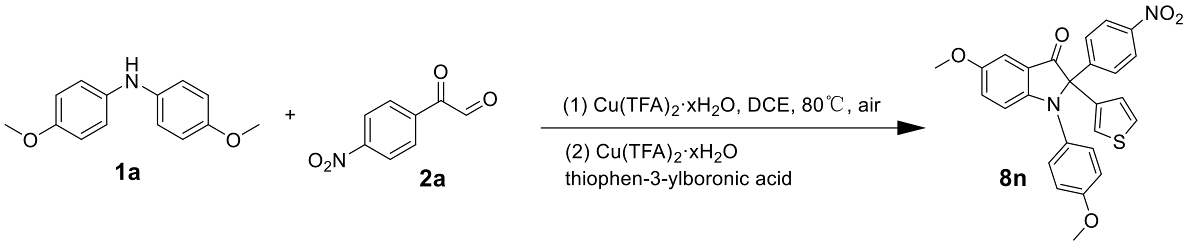

Take the synthesis of **8n** as an example, **1a** (0.2 mmol), **2a** (0.4 mmol), $Cu(TFA)_2 \cdot xH_2O$ (0.04 mmol) and DCE (2.0 mL, *c* 0.1 M) were sealed in a heavy-wall pressure vessel and heated with stirring at 80 °C for 4 h. After the completion of the reaction was monitored by TLC, the reaction was continued for 8 h by adding $Cu(TFA)_2 \cdot xH_2O$ (0.04 mmol) and thiophen-3-ylboronic acid (0.6 mmol) to the reaction solution. The reaction was cooled to room temperature and extracted with ethyl acetate. The organic layer was washed sequentially with $NaHCO_3$ saturated solution and NaCl saturated solution, dried over Na_2SO_4, filtered and concentrated under reduced pressure. The crude product was purified by flash column chromatography on silica gel (eluted with PE : EA = 6:1) to obtain compound **8n**.

4. Synthesis and characterization data of compounds

5-methoxy-1-(4-methoxyphenyl)-3-(4-nitrophenyl)-2-phenyl-1H-indole (**4a**)

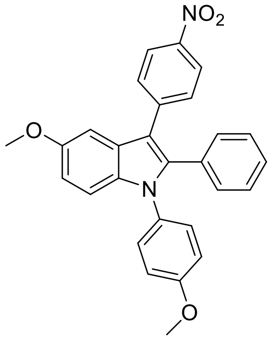

Compound **4a** was synthesized according to general procedure 3.3 starting from bis(4-methoxyphenyl)amine, 4-nitrophenylglyoxal and phenylboronic acid. Compound **4a** was obtained in 56% yield (50.2 mg) as a yellow solid.

^{1}H NMR (600 MHz, $CDCl_3$): δ 8.17 (d, J = 8.8 Hz, 2H), 7.48 (d, J = 8.8 Hz, 2H), 7.26 – 7.23 (m, 2H), 7.19 (t, J = 8.5 Hz, 3H), 7.13 (d, J = 8.8 Hz, 2H), 7.08 (d, J = 7.0 Hz, 2H), 6.93 (dd, J = 8.9 Hz, 2.4 Hz, 1H), 6.89 (d, J = 8.8 Hz, 2H), 3.88 (s, 3H), 3.82 (s, 3H).

^{13}C NMR (151 MHz, $CDCl_3$): δ 158.9, 155.8, 145.6, 143.1, 139.5, 133.9, 131.2, 131.1, 130.4, 130.2, 129.4, 128.4, 128.2, 127.0, 123.9, 114.5, 114.0, 113.3, 112.1, 100.8, 56.2, 55.6.

HRMS (ESI): m/z calcd for $C_{28}H_{23}N_2O_4^+$ $(M+H)^+$ 451.16523, found 451.16583.

2-(4-fluorophenyl)-5-methoxy-1-(4-methoxyphenyl)-3-(4-nitrophenyl)-1H-indole (**4b**)

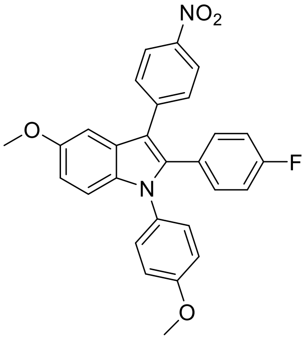

Compound **4b** was synthesized according to general procedure 3.3 starting from bis(4-methoxyphenyl)amine, 4-nitrophenylglyoxal and (4-fluorophenyl)boronic acid. Compound **4b** was obtained in 66% yield (62.1 mg) as a yellow solid.

^{1}H NMR (600 MHz, $CDCl_3$):δ 8.19 (d, J = 8.8 Hz, 2H), 7.47 (d, J = 8.8 Hz, 2H), 7.23 (d, J = 2.4 Hz, 1H), 7.17 (d, J = 8.9 Hz, 1H), 7.11 (d, J = 8.8 Hz, 2H), 7.06 (d, J =5.5 Hz, 1H), 7.04 (d, J = 5.5 Hz, 1H), 6.94 – 6.89 (m, 5H), 3.88 (s, 3H), 3.84 (s, 3H).

^{13}C NMR (151 MHz, $CDCl_3$):δ 162.5 (d, J = 248.9 Hz), 159.0, 155.8, 145.7, 142.8, 138.3, 133.9, 132.9 (d, J = 8.2 Hz), 130.2, 130.2, 129.4, 127.2 (d, J = 3.4 Hz), 126.9, 124.0, 115.7 (d, J = 21.6 Hz), 114.6, 114.2, 113.4, 112.1, 100.8, 56.2, 55.6.

^{19}F NMR (565 MHz, $CDCl_3$): δ -112.57 (ddd, J = 14.0 Hz, 8.8 Hz, 5.3 Hz).

HRMS (ESI): m/z calcd for $C_{28}H_{22}FN_2O_4^+$ $(M+H)^+$ 469.15581, found 469.15598.

2-(4-chlorophenyl)-5-methoxy-1-(4-methoxyphenyl)-3-(4-nitrophenyl)-1H-indole (**4c**)

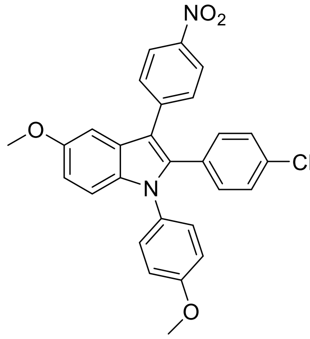

Compound **4c** was synthesized according to general procedure 3.3 starting from bis(4-methoxyphenyl)amine, 4-nitrophenylglyoxal and (4-chlorophenyl)boronic acid. Compound **4c** was obtained in 62% yield (60.1 mg) as a yellow solid.

^{1}H NMR (600 MHz, $CDCl_3$): δ 8.19 (d, J = 8.3 Hz, 2H), 7.49 (d, J = 8.3 Hz, 2H), 7.23 (d, J = 2.4 Hz, 1H), 7.18 (d, J = 8.5 Hz, 3H), 7.12 (d, J = 8.4 Hz, 2H), 7.02 (d, J = 8.1 Hz, 2H), 6.94–6.92 (m, 3H), 3.87 (s, 3H), 3.85 (s, 3H).

^{13}C NMR (151 MHz, $CDCl_3$): δ 159.0, 155.8, 145.7, 142.6, 137.8, 134.2, 133.9, 132.3, 130.2, 130.1, 129.5, 129.3, 128.7, 126.8, 123.9, 114.6, 114.3, 113.5, 112.1, 100.7, 56.0, 55.5.

HRMS (ESI): m/z calcd for $C_{28}H_{21}ClN_2O_4Na^+$ $(M+Na)^+$ 507.10821, found 507.10846.

4-(5-methoxy-1-(4-methoxyphenyl)-3-(4-nitrophenyl)-1H-indol-2-yl)benzonitrile (**4d**)

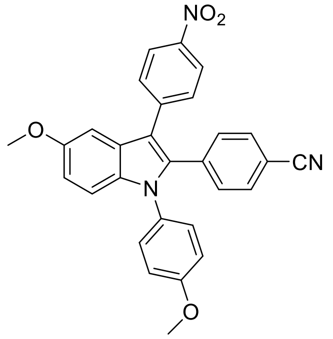

Compound **4d** was synthesized according to general procedure 3.3 starting from bis(4-methoxyphenyl)amine, 4-nitrophenylglyoxal and (4-cyanophenyl)boronic acid. Compound **4d** was obtained in 41% yield (38.6 mg) as a yellow solid.

^{1}H NMR (600 MHz, CDCl$_3$): δ 8.21 (d, J = 8.7 Hz, 2H), 7.47 (d, J = 4.3 Hz, 2H), 7.46 (d, J = 4.8 Hz, 2H), 7.19 – 7.16 (m, 4H), 7.10 (d, J = 8.8 Hz, 2H), 6.95 (dd, J = 8.9Hz, 2.4 Hz, 1H), 6.92 (d, J = 8.8 Hz, 2H), 3.86 (s, 3H), 3.85 (s, 3H).

^{13}C NMR (151 MHz, CDCl$_3$): δ 159.2, 156.0, 146.1, 142.1, 136.6, 135.9, 134.3, 132.1, 131.6, 130.5, 129.8, 129.3, 126.9, 124.2, 118.5, 115.5, 114.8, 114.5, 112.3, 111.6, 100.6, 56.1, 55.6.

HRMS (ESI): m/z calcd for $C_{29}H_{21}N_3O_4Na^+$ $(M+Na)^+$ 498.14243, found 498.14264.

5-methoxy-1,2-bis(4-methoxyphenyl)-3-(4-nitrophenyl)-1H-indole (**4e**)

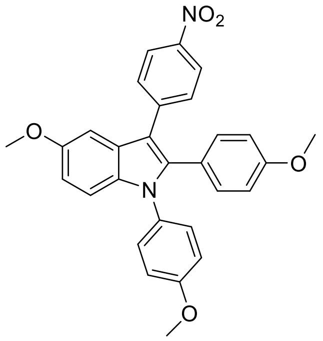

Compound **4e** was synthesized according to general procedure 3.3 starting from bis(4-methoxyphenyl)amine, 4-nitrophenylglyoxal and (4-methoxyphenyl)boronic acid. Compound **4e** was obtained in 56% yield (53.5 mg) as a yellow solid.

^{1}H NMR (600 MHz, CDCl$_3$): δ 8.18 (d, J = 8.4 Hz, 2H), 7.49 (d, J = 8.4 Hz, 2H), 7.25 (d, J = 2.4 Hz, 1H), 7.17 (d, J = 8.9 Hz, 1H), 7.13 (d, J = 8.7 Hz, 2H), 6.99 (d, J = 8.4 Hz, 2H), 6.90 (d, J = 8.9 Hz, 3H), 6.73 (d, J = 8.5 Hz, 2H), 3.88 (s, 3H), 3.83 (s, 3H), 3.77 (s, 3H).

^{13}C NMR (151 MHz, CDCl$_3$): δ 159.4, 158.8, 155.7, 145.5, 143.3, 139.5, 133.9, 132.4, 130.5, 130.1, 129.4, 127.0, 123.9, 123.2, 114.5, 113.9, 113.6, 112.9, 112.0, 100.7, 56.1, 55.6, 55.2.

HRMS (ESI): m/z calcd for $C_{29}H_{24}N_2O_5Na^+$ $(M+Na)^+$ 503.15774, found 503.15784.

2-(3-chlorophenyl)-5-methoxy-1-(4-methoxyphenyl)-3-(4-nitrophenyl)-1H-indole (**4f**)

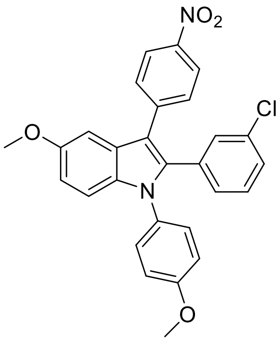

Compound **4f** was synthesized according to general procedure 3.3 starting from bis(4-methoxyphenyl)amine, 4-nitrophenylglyoxal and (3-chlorophenyl)boronic acid. Compound **4f** was obtained in 68% yield (65.6 mg) as a yellow solid.

^{1}H NMR (600 MHz, CDCl$_3$): δ 8.20 (d, J = 8.7 Hz, 2H), 7.48 (d, J = 8.8 Hz, 2H), 7.22 (d, J = 2.6 Hz, 2H), 7.17 (d, J = 8.9 Hz, 1H), 7.14 – 7.12 (m, 3H), 7.07 (t, J = 1.8 Hz, 1H), 6.96 (dt, J = 7.8 Hz, 1.4 Hz, 1H), 6.94 – 6.91 (m, 3H), 3.87 (s, 3H), 3.84 (s, 3H).

^{13}C NMR (151 MHz, CDCl$_3$): δ 159.1, 155.9, 145.8, 142.5, 137.6, 134.2, 134.0, 133.0, 131.0, 130.3, 130.0, 129.7, 129.4, 129.4, 128.3, 126.8, 124.0, 114.7, 114.6, 113.8, 112.2, 100.8, 56.1, 55.6.

HRMS (ESI): m/z calcd for $C_{28}H_{21}ClN_2O_4Na^+$ $(M+Na)^+$ 507.10821, found 507.10837.

5-methoxy-1-(4-methoxyphenyl)-3-(4-nitrophenyl)-2-(m-tolyl)-1H-indole (**4g**)

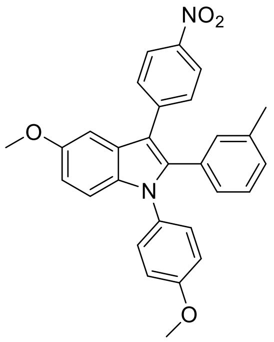

Compound **4g** was synthesized according to general procedure 3.3 starting from bis(4-methoxyphenyl)amine, 4-nitrophenylglyoxal and m-tolylboronic acid. Compound **4g** was obtained in 66% yield (61.3 mg) as a yellow solid.

^{1}H NMR (600 MHz, CDCl$_3$): δ 8.17 (d, J = 8.8 Hz, 2H), 7.49 (d, J = 8.7 Hz, 2H), 7.25 (d, J = 2.4 Hz, 1H), 7.18 (d, J = 8.9 Hz, 1H), 7.13 (d, J = 8.8 Hz, 2H), 7.08 (t, J = 7.5 Hz, 1H), 7.05 (d, J = 7.6 Hz, 1H), 6.92 (dd, J = 8.9Hz, 2.4 Hz, 1H), 6.90 – 6.87 (m, 4H), 3.88 (s, 3H), 3.83 (s, 3H), 2.18 (s, 3H).

^{13}C NMR (151 MHz, CDCl$_3$): δ 158.9, 155.7, 145.5, 143.2, 139.7, 138.0, 133.9, 131.8, 131.0, 130.5, 130.2, 129.4, 129.0, 128.3, 128.3, 126.9, 123.8, 114.4, 113.9, 113.1, 112.1, 100.8, 56.1, 55.6, 21.5.

HRMS (ESI): m/z calcd for $C_{29}H_{24}N_2O_4Na^+$ $(M+Na)^+$ 487.16283, found 487.16296.

2-(3,4-dichlorophenyl)-5-methoxy-1-(4-methoxyphenyl)-3-(4-nitrophenyl)-1H-indole (**4h**)

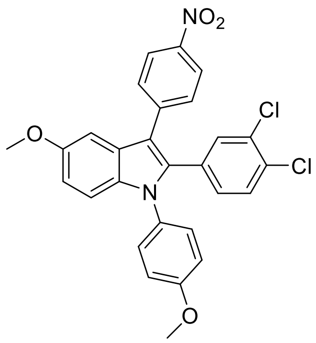

Compound **4h** was synthesized according to general procedure 3.3 starting from bis(4-methoxyphenyl)amine, 4-nitrophenylglyoxal and (3,4-dichlorophenyl)boronic acid. Compound **4h** was obtained in 59% yield (61.2 mg) as a yellow solid.

^{1}H NMR (600 MHz, CDCl$_3$):δ 8.21 (d, J = 8.6 Hz, 2H), 7.47 (d, J = 8.6 Hz, 2H), 7.25 (d, J = 3.1 Hz, 1H), 7.18 (d, J = 2.4 Hz, 1H), 7.15 (dd, J = 5.5Hz, 3.5 Hz, 2H), 7.11 (d, J = 8.7 Hz, 2H), 6.93 (d, J = 8.6 Hz, 3H), 6.89 (dd, J = 8.3Hz, 2.0 Hz, 1H), 3.86 (s, 3H), 3.84 (s, 3H).

^{13}C NMR (151 MHz, CDCl$_3$):δ 159.2, 155.9, 146.0, 142.2, 136.3, 134.1, 132.7, 132.7, 132.5, 131.2, 130.5, 130.3, 130.3, 129.9, 129.4, 126.8, 124.2, 114.9, 114.8, 114.1, 112.2, 100.7, 56.1, 55.7.

HRMS (ESI): m/z calcd for $C_{28}H_{20}Cl_2N_2O_4Na^+$ $(M+Na)^+$ 541.0692337, found 541.06934.

2-(2-bromophenyl)-5-methoxy-1-(4-methoxyphenyl)-3-(4-nitrophenyl)-1H-indole (**4i**)

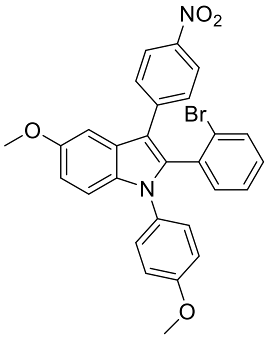

Compound **4i** was synthesized according to general procedure 3.3 starting from bis(4-methoxyphenyl)amine, 4-nitrophenylglyoxal and (2-bromophenyl)boronic acid. Compound **4i** was obtained in 47% yield (49.6 mg) as a yellow solid.

^{1}H NMR (600 MHz, CDCl$_3$):δ 8.15 (d, J = 8.6 Hz, 2H), 7.49 (t, J = 8.9 Hz, 3H), 7.31 (d, J = 2.4 Hz, 1H), 7.24 – 7.16 (m, 6H), 6.96 (dd, J = 8.9 Hz, 2.4 Hz, 1H), 6.85 (d, J = 8.5 Hz, 2H). 3.89(s, 3H), 3.80(s, 3H).

^{13}C NMR (151 MHz, CDCl$_3$):δ 159.0, 155.8, 145.7, 142.7, 138.2, 133.7, 133.5, 133.0, 130.6, 129.9, 129.3, 129.2, 127.3, 126.4, 125.7, 123.9, 114.9, 114.3, 113.6, 112.2, 101.0, 56.2, 55.5.

HRMS (ESI): m/z calcd for $C_{28}H_{21}BrN_2O_4Na^+$ $(M+Na)^+$ 551.05769, found 551.05780.

2-(benzo[d][1,3]dioxol-5-yl)-5-methoxy-1-(4-methoxyphenyl)-3-(4-nitrophenyl)-1H-indole (**4j**)

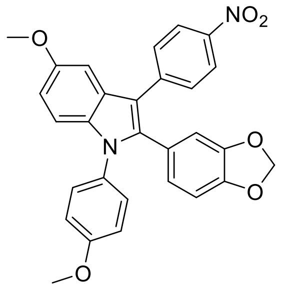

Compound **4j** was synthesized according to general procedure 3.3 starting from bis(4-methoxyphenyl)amine, 4-nitrophenylglyoxal and benzo[d][1,3]dioxol-5-ylboronic acid. Compound **4j** was obtained in 70% yield (69.5 mg) as a yellow solid.

^{1}H NMR (600 MHz, CDCl$_3$):δ 8.19 (d, J = 8.8 Hz, 2H), 7.50 (d, J = 8.4 Hz, 2H), 7.23 (d, J = 2.4 Hz, 1H), 7.14 (t, J = 9.3 Hz, 3H), 6.92 – 6.90 (m, 3H), 6.64 (d, J = 8.0 Hz, 1H), 6.57 (d, J = 8.0 Hz, 1H), 6.53 (s, 1H), 5.94 (s, 2H), 3.87 (s, 3H), 3.84 (s, 3H).

^{13}C NMR (151 MHz, CDCl$_3$):δ 158.9, 155.8, 147.64, 147.62, 145.6, 143.0, 139.2, 133.8, 130.4, 130.1, 129.4, 126.8, 125.4, 124.7, 123.9, 114.6, 113.9, 113.2, 112.1, 111.2, 108.5, 101.4, 100.8, 56.1, 55.6.

HRMS (ESI): m/z calcd for $C_{29}H_{22}N_2O_6Na^+$ $(M+Na)^+$ 517.13701, found 517.13727.

2-(furan-3-yl)-5-methoxy-1-(4-methoxyphenyl)-3-(4-nitrophenyl)-1H-indole (**4k**)

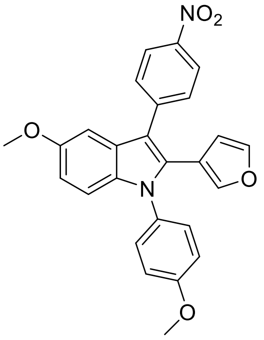

Compound **4k** was synthesized according to general procedure 3.3 starting from bis(4-methoxyphenyl)amine, 4-nitrophenylglyoxal and furan-3-ylboronic acid. Compound **4k** was obtained in 41% yield (36.3 mg) as a yellow solid.

^{1}H NMR (600 MHz, CDCl$_3$):δ 8.26 (d, J = 8.8 Hz, 2H), 7.63 (d, J = 8.8 Hz, 2H), 7.27 (s, 1H), 7.22 (d, J = 8.8 Hz, 2H), 7.14 (d, J = 2.4 Hz, 1H), 7.07 (d, J = 8.9 Hz, 1H), 6.99 – 6.98 (m, 3H), 6.88 (dd, J = 8.9 Hz, 2.4 Hz, 1H), 5.98 (s, 1H), 3.88 (s, 3H), 3.85 (s, 3H).
^{13}C NMR (151 MHz, CDCl$_3$):δ 159.5, 155.7, 146.0, 143.0, 142.9, 142.1, 134.2, 131.1, 130.4, 129.7, 127.0, 124.0, 116.0, 114.7, 114.1, 113.3, 111.9, 111.6, 100.5, 56.1, 55.7.
HRMS (ESI): m/z calcd for $C_{26}H_{20}N_2O_5Na^+$ $(M+Na)^+$ 463.12644, found 463.12646.

5-methoxy-1-(4-methoxyphenyl)-3-(4-nitrophenyl)-2-(thiophen-3-yl)-1H-indole (**4l**)

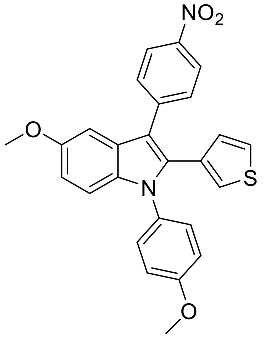

Compound **4l** was synthesized according to general procedure 3.3 starting from bis(4-methoxyphenyl)amine, 4-nitrophenylglyoxal and thiophen-3-ylboronic acid. Compound **4l** was obtained in 62% yield (56.7 mg) as a yellow solid.

^{1}H NMR (600 MHz, $CDCl_3$): δ 8.21 (d, J = 8.4 Hz, 2H), 7.53 (d, J = 8.4 Hz, 2H), 7.20 (s, 1H), 7.17 (d, J = 8.5 Hz, 3H), 7.14 (d, J = 9.1 Hz, 1H), 6.94 (d, J = 8.4 Hz, 2H), 6.91 (dd, J = 9.1 Hz, 2.5 Hz 2H), 6.69 (d, J = 5.0 Hz, 1H), 3.87 (s, 3H), 3.86 (s, 3H).

^{13}C NMR (151 MHz, $CDCl_3$): δ 159.1, 155.7, 145.7, 143.0, 134.6, 133.9, 131.1, 130.4, 130.2, 129.3, 129.1, 126.9, 126.4, 125.6, 123.9, 114.6, 114.1, 113.3, 112.0, 100.6, 56.1, 55.6.

HRMS(ESI): m/z calcd for $C_{26}H_{20}N_2O_4SNa^+$ $(M+Na)^+$ 479.10360, found 479.10367.

5-methoxy-1-(4-methoxyphenyl)-3-(4-nitrophenyl)-2-(thiophen-2-yl)-1H-indole (**4m**)

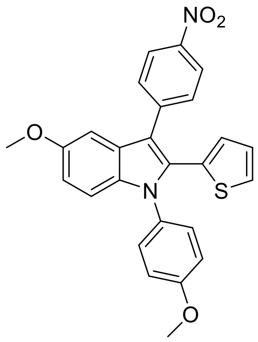

Compound **4m** was synthesized according to general procedure 3.3 starting from bis(4-methoxyphenyl)amine, 4-nitrophenylglyoxal and thiophen-2-ylboronic acid. Compound **4m** was obtained in 40% yield (36.4 mg) as a yellow solid.

^{1}H NMR (600 MHz, $CDCl_3$): δ 8.22 (d, J = 8.5 Hz, 2H), 7.57 (d, J = 8.2 Hz, 2H), 7.25 (s, 1H), 7.20 (d, J = 8.7 Hz, 2H), 7.16 (d, J = 2.4 Hz, 1H), 7.10 (d, J = 8.9 Hz, 1H), 6.94 (d, J = 8.8 Hz, 2H), 6.91 (dd, J = 8.9 Hz, 2.4 Hz, 1H), 6.87 (dd, J = 5.1 Hz, 3.6 Hz, 1H), 6.73 (d, J = 3.8 Hz, 1H), 3.85 (s, 3H), 3.85 (s, 3H).

^{13}C NMR (151 MHz, $CDCl_3$): δ 159.4, 155.8, 146.0, 142.7, 134.2, 132.3, 131.8, 130.4, 130.1, 129.7, 128.1, 127.1, 126.8, 123.9, 115.4, 114.6, 113.9, 112.2, 100.6, 56.1, 55.6.

HRMS (ESI): m/z calcd for $C_{26}H_{20}N_2O_4SNa^+$ $(M+Na)^+$ 479.10360, found 479.10373.

2-(benzo[b]thiophen-3-yl)-5-methoxy-1-(4-methoxyphenyl)-3-(4-nitrophenyl)-1H-indole (**4n**)

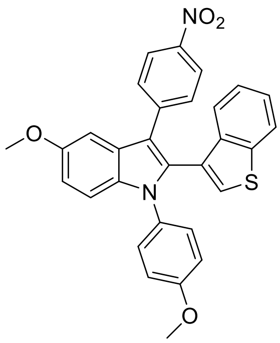

Compound **4n** was synthesized according to general procedure 3.3 starting from bis(4-methoxyphenyl)amine, 4-nitrophenylglyoxal and benzo[b]thiophen-2-ylboronic acid. Compound **4n** was obtained in 64% yield (65.2 mg) as a yellow solid.

^{1}H NMR (600 MHz, $CDCl_3$): δ 8.05 (d, J = 8.9 Hz, 2H), 7.77 (d, J = 8.1 Hz, 1H), 7.47 (d, J = 8.9 Hz, 2H), 7.34 – 7.32 (m, 2H), 7.25 – 7.22 (m, 3H), 7.20 (s, 1H), 7.14 – 7.11 (m, 2H), 6.96 (dd, J = 8.9 Hz, 2.4 Hz, 1H), 6.78 (d, J = 8.5 Hz, 2H), 3.90 (s, 3H), 3.74 (s, 3H).

^{13}C NMR (151 MHz, $CDCl_3$): δ 158.9, 155.8, 145.5, 142.8, 139.6, 138.1, 134.0, 133.3, 130.2, 129.2, 129.1, 128.7, 127.0, 126.7, 124.8, 124.6, 123.9, 123.1, 122.7, 115.4, 114.4, 113.5, 112.2, 100.9, 56.2, 55.5.

HRMS (ESI): m/z calcd for $C_{30}H_{22}N_2O_4SNa^+$ $(M+Na)^+$ 529.11925, found 529.11938.

2-(dibenzo[b,d]furan-4-yl)-5-methoxy-1-(4-methoxyphenyl)-3-(4-nitrophenyl)-1H-indole (**4o**)

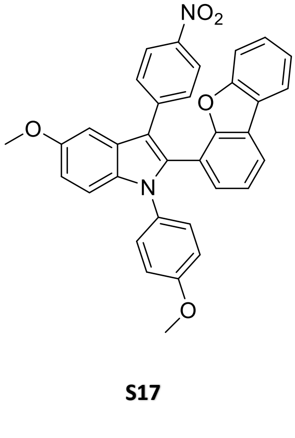

Compound **4o** was synthesized according to general procedure 3.3 starting from bis(4-methoxyphenyl)amine, 4-nitrophenylglyoxal and dibenzo[b,d]furan-4-ylboronic acid. Compound **4o** was obtained in 56% yield (60.5 mg) as a yellow solid.

^{1}H NMR (600 MHz, CDCl$_3$):δ 8.07 (d, J = 8.9 Hz, 2H), 7.90 – 7.87 (m, 2H), 7.51 (d, J = 8.8 Hz, 2H), 7.41 – 7.38 (m, 1H), 7.34 (dd, J = 5.4 Hz, 2.8 Hz, 2H), 7.32 – 7.30 (m, 1H), 7.25 (d, J = 2.7 Hz, 1H), 7.22 – 7.18 (m, 4H), 6.99 (dd, J = 8.9 Hz, 2.4 Hz, 1H), 6.76 (d, J = 8.7 Hz, 2H), 3.91 (s, 3H), 3.70 (s, 3H).

^{13}C NMR (151 MHz, CDCl$_3$):δ 158.8, 156.0, 155.8, 154.3, 145.6, 143.0, 134.2, 130.3, 129.9, 129.5, 129.0, 127.6, 126.9, 124.7, 123.8, 123.8, 123.1, 122.9, 121.4, 120.9, 116.0, 115.6, 114.2, 113.6, 112.2, 111.6, 100.9, 56.2, 55.4.

HRMS (ESI): m/z calcd for $C_{34}H_{24}N_2O_5Na^+$ $(M+Na)^+$ 563.15774, found 563.15790.

5-methoxy-1-(4-methoxyphenyl)-2-(naphthalen-2-yl)-3-(4-nitrophenyl)-1H-indole (**4p**)

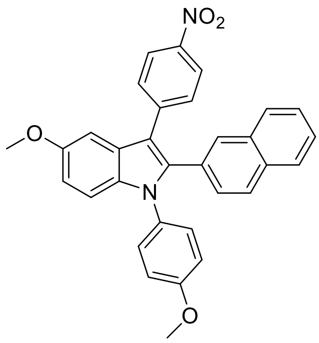

Compound **4p** was synthesized according to general procedure 3.3 starting from bis(4-methoxyphenyl)amine, 4-nitrophenylglyoxal and naphthalen-2-ylboronic acid. Compound **4p** was obtained in 71% yield (70.8 mg) as a yellow solid.

^{1}H NMR (600 MHz, CDCl$_3$):δ 8.14 (d, J = 8.7 Hz, 2H), 7.78 (d, J = 8.1 Hz, 1H), 7.67 (d, J = 8.5 Hz, 1H), 7.61 (d, J = 8.1 Hz, 1H), 7.59 (s, 1H), 7.51 (d, J = 8.7 Hz, 2H), 7.48 (t, J = 7.1 Hz, 1H), 7.44 (t, J = 7.8 Hz, 1H), 7.29 (d, J = 2.4 Hz, 1H), 7.22 (d, J = 8.9 Hz, 1H), 7.19 – 7.17 (m, 3H), 6.96 (dd, J = 8.9 Hz, 2.4 Hz, 1H), 6.86 (d, J = 8.8 Hz, 2H), 3.90 (s, 3H), 3.79 (s, 3H).

^{13}C NMR (151 MHz, CDCl$_3$): δ 158.9, 155.8, 145.6, 143.0, 139.3, 134.1, 133.0, 132.7, 130.9, 130.4, 130.3, 129.4, 128.5, 128.3, 128.3, 128.1, 127.8, 127.0, 126.8, 126.5, 123.9, 114.6, 114.4, 113.4, 112.1, 100.8, 56.1, 55.5.

HRMS (ESI): m/z calcd for $C_{34}H_{24}N_2O_4Na^+$ $(M+Na)^+$ 523.16283, found 523.16309.

5-methoxy-2-(6-methoxynaphthalen-2-yl)-1-(4-methoxyphenyl)-3-(4-nitrophenyl)-1H-indole (**4q**)

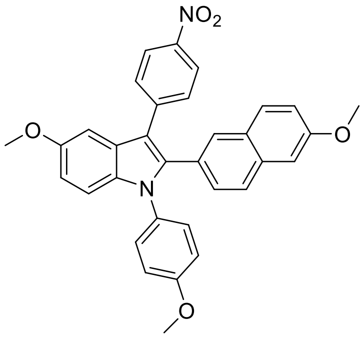

Compound **4q** was synthesized according to general procedure 3.3 starting from bis(4-methoxyphenyl)amine, 4-nitrophenylglyoxal and (6-methoxynaphthalen-2-yl)boronic acid. Compound **4q** was obtained in 50% yield (53.5 mg) as a yellow solid.

^{1}H NMR (600 MHz, CDCl$_3$): δ 8.14 (d, J = 8.8 Hz, 2H), 7.56 (d, J = 8.5 Hz, 1H), 7.50 (d, J = 8.9 Hz, 4H), 7.28 (d, J = 2.4 Hz, 1H), 7.21 (d, J = 8.9 Hz, 1H), 7.16 (d, J = 8.8 Hz, 2H), 7.13 (dd, J = 8.5 Hz, 1.7 Hz, 1H), 7.10 (dd, J = 8.9 Hz, 2.5 Hz, 1H), 7.07 (d, J = 2.5 Hz, 1H), 6.94 (dd, J = 8.9 Hz, 2.4 Hz, 1H), 6.86 (d, J = 8.9 Hz, 2H), 3.91 (s, 3H), 3.90 (s, 3H), 3.79 (s, 3H).

^{13}C NMR (151 MHz, CDCl$_3$): δ 158.8, 158.5, 155.8, 145.5, 143.2, 139.6, 134.0, 130.6, 130.5, 130.2, 129.8, 129.4, 128.9, 128.5, 127.0, 126.9, 126.2, 123.9, 119.3, 114.5, 114.1, 113.2, 112.1, 105.7, 100.8, 56.2, 55.51, 55.47.

HRMS (ESI): m/z calcd for $C_{33}H_{26}N_2O_5Na^+$ $(M+Na)^+$ 553.17339, found 553.17407.

2-(anthracen-2-yl)-5-methoxy-1-(4-methoxyphenyl)-3-(4-nitrophenyl)-1H-indole (**4r**)

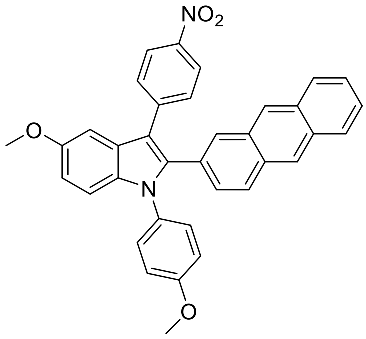

Compound **4r** was synthesized according to general procedure 3.3 starting from bis(4-methoxyphenyl)amine, 4-nitrophenylglyoxal and anthracen-2-ylboronic acid. Compound **4r** was obtained in 41% yield (45.3 mg) as a yellow solid.

^{1}H NMR (600 MHz, CDCl$_3$):δ 8.34 (s, 1H), 8.19 (s, 1H), 8.15 (d, J = 8.8 Hz, 2H), 7.98 – 7.96 (m, 1H), 7.93 – 7.92 (m, 1H), 7.82 (d, J = 8.8 Hz, 1H), 7.75 (s, 1H), 7.55 (d, J = 8.8 Hz, 2H), 7.46 (hept, J = 4.7 Hz, 2H), 7.31 (d, J = 2.4 Hz, 1H), 7.25 (d, J = 4.5 Hz, 1H), 7.21 (d, J = 8.8 Hz, 2H), 7.14 (dd, J = 8.7 Hz, 1.7 Hz, 1H), 6.98 (dd, J = 8.9 Hz, 2.4 Hz, 1H), 6.86 (d, J = 8.8 Hz, 2H), 3.91 (s, 3H), 3.76 (s, 3H).

^{13}C NMR (151 MHz, CDCl$_3$):δ 158.9, 155.8, 145.6, 143.0, 139.3, 134.1, 132.3, 132.0, 131.2, 131.1, 130.6, 130.4, 130.3, 129.3, 128.3, 128.2, 128.2, 127.9, 127.6, 127.1, 126.8, 126.2, 126.0, 125.9, 124.0, 114.6, 114.5, 113.4, 112.2, 100.8, 56.1, 55.5.

HRMS (ESI): m/z calcd for $C_{36}H_{26}N_2O_4Na^+$ $(M+Na)^+$ 573.17848, found 573.17914.

5-methoxy-1-(4-methoxyphenyl)-2-(4-methylnaphthalen-1-yl)-3-(4-nitrophenyl)-1H-indole (**4s**)

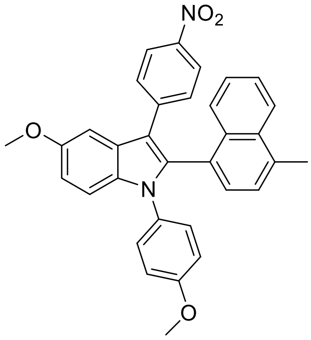

Compound **4s** was synthesized according to general procedure 3.3 starting from bis(4-methoxyphenyl)amine, 4-nitrophenylglyoxal and (4-methylnaphthalen-1-yl)boronic acid. Compound **4s** was obtained in 47% yield (48.2 mg) as a yellow solid.

^{1}H NMR (600 MHz, CDCl$_3$): δ 7.99 (d, J = 8.9 Hz, 2H), 7.94 (d, J = 8.4 Hz, 1H), 7.65 (d, J = 7.9 Hz, 1H), 7.44 – 7.38 (m, 3H), 7.36 (d, J = 2.4 Hz, 1H), 7.29 (ddd, J = 8.2 Hz, 6.7 Hz, 1.2 Hz, 1H), 7.25 – 7.19 (m, 3H), 7.06 (d, J = 8.3 Hz, 2H), 6.97 (dd, J = 8.9 Hz, 2.4 Hz, 1H), 6.68 (d, J = 8.4 Hz, 2H), 3.92 (s, 3H), 3.70 (s, 3H), 2.66 (s, 3H).

^{13}C NMR (151 MHz, CDCl$_3$): δ 158.7, 155.8, 145.3, 143.0, 138.5, 136.0, 133.9, 132.8, 132.6, 130.3, 130.2, 129.1, 128.9, 127.3, 126.7, 126.4, 126.3, 126.1, 126.0, 124.6, 123.8, 115.4, 114.2, 113.1, 112.2, 101.0, 56.3, 55.4, 19.8.

HRMS (ESI): m/z calcd for $C_{33}H_{26}N_2O_4Na^+$ $(M+Na)^+$ 537.17848, found 537.17889.

5-methoxy-1-(4-methoxyphenyl)-3-(4-nitrophenyl)-2-(4-phenylnaphthalen-1-yl)-1H-indole (**4t**)

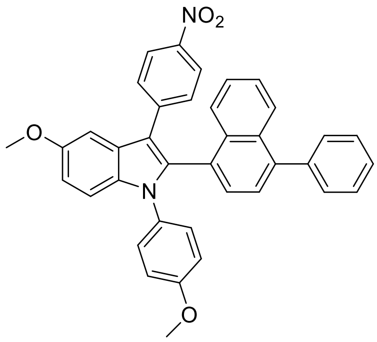

Compound **4t** was synthesized according to general procedure 3.3 starting from bis(4-methoxyphenyl)amine, 4-nitrophenylglyoxal and (4-phenylnaphthalen-1-yl)boronic acid. Compound **4t** was obtained in 55% yield (63.5 mg) as a yellow solid.

^{1}H NMR (600 MHz, CDCl$_3$): δ 8.04 (d, J = 8.9 Hz, 2H), 7.87 (d, J = 9.8 Hz, 1H), 7.73 (d, J = 9.0 Hz, 1H), 7.50 – 7.39 (m, 7H), 7.41 (d, J = 7.2 Hz, 1H), 7.39 (d, J = 2.4 Hz, 1H), 7.32 (q, J = 6.9 Hz, 3H), 7.24 (s, 1H), 7.12 (d, J = 8.3 Hz, 2H), 7.00 (dd, J = 8.9 Hz, 2.4 Hz, 1H), 6.73 (d, J = 8.4 Hz, 2H), 3.94 (s, 3H), 3.72 (s, 3H).

^{13}C NMR (151 MHz, $CDCl_3$): δ 158.7, 155.9, 145.4, 142.9, 141.6, 140.3, 138.1, 134.0, 133.2, 131.6, 130.3, 130.2, 130.0, 129.2, 128.9, 128.5, 128.4, 127.6, 126.8, 126.6, 126.5, 126.2, 126.2, 126.1, 123.8, 115.6, 114.2, 113.3, 112.2, 101.0, 56.2, 55.4.

HRMS (ESI): m/z calcd for $C_{38}H_{28}N_2O_4Na^+$ $(M+Na)^+$ 599.19413, found 599.19470.

2-(4-fluoronaphthalen-1-yl)-5-methoxy-1-(4-methoxyphenyl)-3-(4-nitrophenyl)-1H-indole (**4u**)

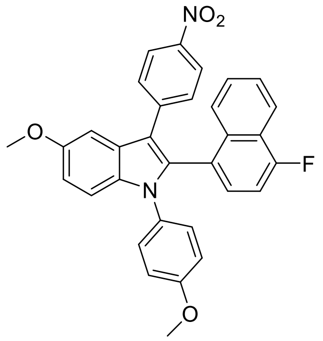

Compound **4u** was synthesized according to general procedure 3.3 starting from bis(4-methoxyphenyl)amine, 4-nitrophenylglyoxal and (4-fluoronaphthalen-1-yl)boronic acid. Compound **4u** was obtained in 63% yield (65.5 mg) as a yellow solid.

^{1}H NMR (600 MHz, $CDCl_3$): δ 8.06 (d, J = 8.4 Hz, 1H), 8.02 (d, J = 8.9 Hz, 2H), 7.64 (d, J = 8.5 Hz, 1H), 7.46 (t, J = 7.6 Hz, 1H), 7.40 (d, J = 8.8 Hz, 2H), 7.37 – 7.35 (m, 2H), 7.30 (dd, J = 7.9 Hz, 5.2 Hz, 1H), 7.23 (d, J = 8.9 Hz, 1H), 7.06 – 7.03 (m, 3H), 6.99 (dd, J = 8.9 Hz, 2.4 Hz, 1H), 6.71 (d, J = 8.5 Hz, 2H), 3.92 (s, 3H), 3.71 (s, 3H).

^{13}C NMR (151 MHz, $CDCl_3$): δ 159.2 (d, J = 255.2 Hz), 158.8, 155.9, 145.5, 142.7, 137.2, 134.2 (d, J = 5.1 Hz), 133.9, 130.5 (d, J = 8.4 Hz), 130.1, 129.1, 128.8, 127.7, 126.7, 126.6, 125.7 (d, J = 2.4 Hz), 125.2 (d, J = 4.4 Hz), 123.8, 123.7 (d, J = 16.4 Hz), 121.0 (d, J = 5.3 Hz), 115.7, 114.3, 113.4, 112.2, 109.1 (d, J = 20.4 Hz), 101.0, 56.2, 55.4.

^{19}F NMR (565 MHz, $CDCl_3$): δ -120.36 (dd, J = 10.8 Hz, 5.4 Hz).

HRMS (ESI): m/z calcd for $C_{32}H_{23}FN_2O_4Na^+$ $(M+Na)^+$ 541.15341, found 541.15387.

5-methoxy-2-(2-methoxynaphthalen-1-yl)-1-(4-methoxyphenyl)-3-(4-nitrophenyl)-1H-indole (**4v**)

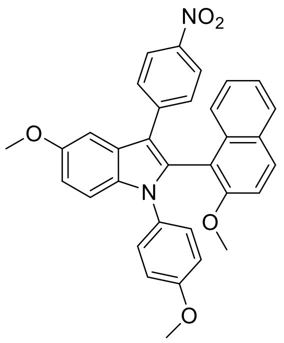

Compound **4v** was synthesized according to general procedure 3.3 starting from bis(4-methoxyphenyl)amine, 4-nitrophenylglyoxal and (2-methoxynaphthalen-1-yl)boronic acid. Compound **4v** was obtained in 55% yield (58.8 mg) as a yellow solid.

^{1}H NMR (600 MHz, $CDCl_3$): δ 8.00 (d, J = 8.9 Hz, 2H), 7.83 (d, J = 9.1 Hz, 1H), 7.71 – 7.70 (m, 1H), 7.46 (d, J = 8.9 Hz, 2H), 7.39 (dd, J = 6.2 Hz, 3.4 Hz, 2H), 7.25 – 7.24 (m, 2H), 7.22 (d, J = 8.9 Hz, 1H), 7.17 (d, J = 9.1 Hz, 1H), 7.08 (s, 2H), 6.96 (dd, J = 8.9 Hz, 2.4 Hz, 1H), 6.68 (s, 2H), 3.92 (s, 3H), 3.70 (s, 6H).

^{13}C NMR (151 MHz, $CDCl_3$): δ 158.7, 156.4, 155.6, 145.3, 143.3, 134.9, 134.1, 134.0, 131.6, 130.4, 128.6, 128.6, 128.4, 128.2, 127.4, 126.9, 124.5, 123.9, 123.8, 115.4, 114.1, 113.9, 112.9, 112.6, 112.1, 101.1, 56.3, 56.1, 55.4.

HRMS (ESI): m/z calcd for $C_{33}H_{26}N_2O_5Na^+$ $(M+Na)^+$ 553.17339, found 553.17383.

2-(2-ethoxynaphthalen-1-yl)-5-methoxy-1-(4-methoxyphenyl)-3-(4-nitrophenyl)-1H-indole (**4w**)

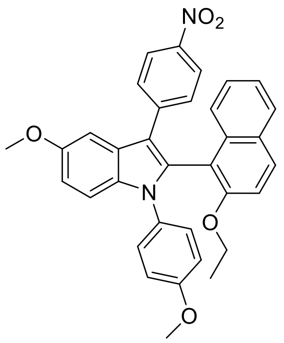

Compound **4w** was synthesized according to general procedure 3.3 starting from bis(4-methoxyphenyl)amine, 4-nitrophenylglyoxal and (2-ethoxynaphthalen-1-yl)boronic acid. Compound **4w** was obtained in 37% yield (40.4 mg) as a yellow solid.

^{1}H NMR (600 MHz, CDCl$_3$): δ 8.00 (d, J = 8.9 Hz, 2H), 7.81 (d, J = 9.1 Hz, 1H), 7.70 – 7.69 (m, 1H), 7.46 (d, J = 8.9 Hz, 2H), 7.40 (d, J = 2.4 Hz, 1H), 7.38 – 7.36 (m, 1H), 7.25 – 7.22 (m, 3H), 7.15 (d, J = 9.1 Hz, 1H), 7.09 (s, 2H), 6.96 (dd, J = 8.9 Hz, 2.4 Hz, 1H), 6.67 (d, J = 7.7 Hz, 2H), 4.01 – 3.95 (m, 2H), 3.93 (s, 3H), 3.69 (s, 3H), 1.18 (t, J = 7.0 Hz, 3H).

^{13}C NMR (151 MHz, CDCl$_3$): δ 158.7, 155.8, 155.6, 145.3, 143.4, 135.1, 134.5, 133.9, 131.4, 130.4, 128.6, 128.5, 128.4, 128.1, 127.3, 127.0, 124.5, 123.8, 123.7, 115.4, 114.1, 113.9, 113.4, 112.7, 112.0, 101.1, 64.2, 56.2, 55.4, 15.1.

HRMS (ESI): m/z calcd for $C_{34}H_{28}N_2O_5Na^+$ $(M+Na)^+$ 567.18904, found 567.18976.

5-methoxy-1-(4-methoxyphenyl)-3-(4-nitrophenyl)-2-(pyren-1-yl)-1H-indole (**4x**)

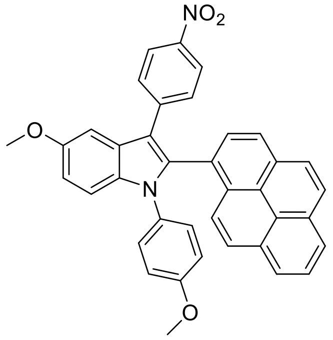

Compound **4x** was synthesized according to general procedure 3.3 starting from bis(4-methoxyphenyl)amine, 4-nitrophenylglyoxal and pyren-1-ylboronic acid. Compound **4x** was obtained in 55% yield (63.9 mg) as a yellow solid.

^{1}H NMR (600 MHz, CDCl$_3$): δ 8.20 (d, J = 7.7 Hz, 1H), 8.12 (d, J = 7.7 Hz, 1H), 8.10 (d, J = 8.9 Hz, 1H), 8.06 (d, J = 7.8 Hz, 1H), 8.04 – 7.99 (m, 2H), 7.93 – 7.91 (m, 4H), 7.84 (d, J = 7.8 Hz, 1H), 7.43 (d, J = 2.4 Hz, 1H), 7.40 (d, J = 8.9 Hz, 2H), 7.30 (d, J = 8.9 Hz, 1H), 7.11 (d, J = 8.2 Hz, 2H), 7.03 (dd, J = 8.9 Hz, 2.4 Hz, 1H), 6.61 (d, J = 9.1 Hz, 2H), 3.96 (s, 3H). 3.59 (s, 3H).

^{13}C NMR (151 MHz, CDCl$_3$): δ 158.6, 155.9, 145.3, 142.9, 138.5, 134.1, 131.7, 131.3, 130.9, 130.7, 130.2, 129.7, 129.2, 128.9, 128.4, 128.4, 127.4, 126.9, 126.4, 126.1, 125.8, 125.6, 124.9, 124.7, 124.6, 124.5, 123.8, 116.0, 114.3, 113.4, 112.2, 101.1, 56.3, 55.3.

HRMS (ESI): m/z calcd for $C_{38}H_{26}N_2O_4Na^+$ $(M+Na)^+$ 597.1784785, found 597.17883.

(E)-5-methoxy-1-(4-methoxyphenyl)-3-(4-nitrophenyl)-2-styryl-1H-indole (**4y**)

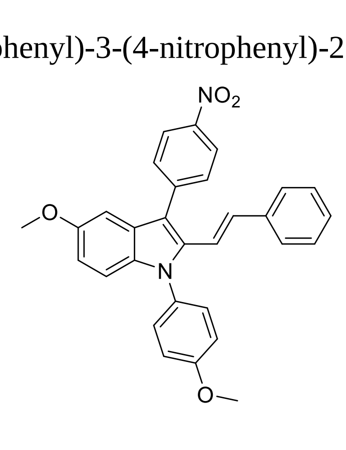

Compound **4y** was synthesized according to general procedure 3.3 starting from bis(4-methoxyphenyl)amine, 4-nitrophenylglyoxal and (E)-styrylboronic acid. Compound **4y** was obtained in 67% yield (63.6 mg) as a yellow solid.

^{1}H NMR (600 MHz, CDCl$_3$): δ 8.37 (d, J = 8.7 Hz, 2H), 7.79 (d, J = 8.7 Hz, 2H), 7.35 (d, J = 8.8 Hz, 2H), 7.27 (d, J = 7.1 Hz, 1H), 7.25 (s, 1H), 7.22 (t, J = 7.3 Hz, 1H), 7.15 (d, J = 7.3 Hz, 2H), 7.10 – 7.09 (m, 2H), 7.08 (s, 1H), 7.03 (d, J = 8.9 Hz, 1H), 6.93 (d, J = 16.7 Hz, 1H), 6.89 (dd, J = 8.9 Hz, 2.4 Hz, 1H), 6.38 (d, J = 16.6 Hz, 1H), 3.93 (s, 3H), 3.85 (s, 3H).

^{13}C NMR (151 MHz, CDCl$_3$): δ 159.6, 155.7, 146.1, 143.2, 136.9, 135.6, 134.8, 133.6, 130.8, 130.7, 129.7, 128.8, 128.2, 127.2, 126.4, 124.2, 116.7, 115.5, 115.0, 113.9, 111.8, 100.3, 56.1, 55.7.

HRMS (ESI): m/z calcd for $C_{30}H_{24}N_2O_4Na^+$ $(M+Na)^+$ 499.16283, found 499.16293.

2-(cyclohex-1-en-1-yl)-5-methoxy-1-(4-methoxyphenyl)-3-(4-nitrophenyl)-1H-indole (**4z**)

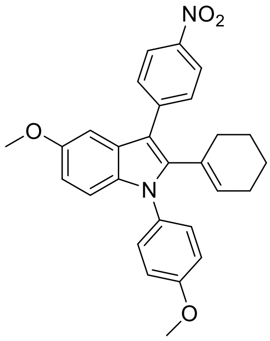

Compound **4z** was synthesized according to general procedure 3.3 starting from bis(4-methoxyphenyl)amine, 4-nitrophenylglyoxal and cyclohex-1-en-1-ylboronic acid. Compound **4z** was obtained in 26% yield (23.8 mg) as a yellow solid.

^{1}H NMR (600 MHz, CDCl$_3$): δ 8.27 (d, J = 8.8 Hz, 2H), 7.70 (d, J = 8.8 Hz, 2H), 7.26 – 7.25 (m, 2H), 7.19 (d, J = 2.4 Hz, 1H), 7.09 (d, J = 8.9 Hz, 1H), 7.00 (d, J = 8.8 Hz, 2H),

6.85 (dd, J = 8.9 Hz, 2.4 Hz, 1H), 5.71 (dt, J = 3.9 Hz, 2.0 Hz, 1H), 3.88 (s, 3H), 3.84 (s, 3H), 1.98 – 1.96 (m, 2H), 1.84 (dd, J = 4.4 Hz, 2.1 Hz, 2H), 1.49 – 1.47 (m, 4H).

^{13}C NMR (151 MHz, $CDCl_3$):δ 159.0, 155.6, 145.5, 143.8, 142.6, 134.9, 133.3, 130.8, 129.5, 128.9, 128.9, 126.7, 123.9, 114.4, 112.6, 112.5, 111.8, 100.8, 56.2, 55.7, 29.3, 25.8, 22.7, 21.7.

HRMS (ESI): m/z calcd for $C_{28}H_{26}N_2O_4Na^+$ $(M+Na)^+$ 477.17848, found 477.17871.

5-methyl-2-(naphthalen-2-yl)-3-(4-nitrophenyl)-1-(p-tolyl)-1H-indole (**5a**)

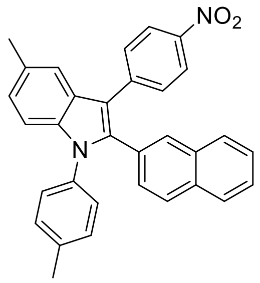

Compound **5a** was synthesized according to general procedure 3.3 starting from di-p-tolylamine, 4-nitrophenylglyoxal and naphthalen-2-ylboronic acid. Compound **5a** was obtained in 37% yield (34.9 mg) as a yellow solid.

^{1}H NMR (600 MHz, $CDCl_3$):δ 8.15 (d, J = 8.5 Hz, 2H), 7.80 (d, J = 8.1 Hz, 1H), 7.68 (d, J = 8.5 Hz, 1H), 7.65 (s, 1H), 7.63 – 7.61 (m, 2H), 7.53 (d, J = 8.4 Hz, 2H), 7.49 (t, J = 7.5 Hz, 1H), 7.45 (t, J = 7.4 Hz, 1H), 7.27 (s, 1H), 7.20 (d, J = 8.5 Hz, 1H), 7.15 (d, J = 9.7 Hz, 5H), 2.54 (s, 3H), 2.36 (s, 3H).

^{13}C NMR (151 MHz, $CDCl_3$):δ 145.6, 143.0, 138.7, 137.6, 137.0, 135.1, 133.1, 132.7, 131.2, 130.9, 130.5, 130.0, 128.6, 128.4, 128.3, 128.1, 127.8, 127.0, 126.8, 126.4, 125.0, 123.9, 118.6, 114.5, 111.1, 21.7, 21.3.

HRMS (ESI): m/z calcd for $C_{32}H_{24}N_2O_2Na^+$ $(M+Na)^+$ 491.17300, found 491.17328.

5-isopropoxy-1-(4-isopropoxyphenyl)-2-(naphthalen-2-yl)-3-(4-nitrophenyl)-1H-indole (**5b**)

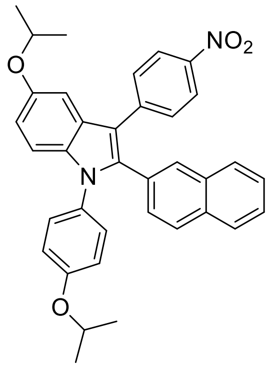

Compound **5b** was synthesized according to general procedure 3.3 starting from **9**, 4-nitrophenylglyoxal and naphthalen-2-ylboronic acid. Compound **5b** was obtained in 39% yield (43.9 mg) as a yellow solid.

^{1}H NMR (600 MHz, CDCl$_3$): δ 8.13 (d, J = 8.8 Hz, 2H), 7.78 (d, J = 8.1 Hz, 1H), 7.66 (d, J = 8.5 Hz, 1H), 7.60 (d, J = 8.1 Hz, 1H), 7.58 (s, 1H), 7.49 (d, J = 8.8 Hz, 2H), 7.48 – 7.46 (m, 1H), 7.44 – 7.41 (m, 1H), 7.33 (d, J = 2.3 Hz, 1H), 7.23 (d, J = 8.9 Hz, 1H), 7.17 (dd, J = 8.4 Hz, 1.7 Hz, 1H), 7.14 (d, J = 8.8 Hz, 2H), 6.95 (dd, J = 8.9 Hz, 2.3 Hz, 1H), 6.83 (d, J = 8.9 Hz, 2H), 4.58 (p, J = 6.1 Hz, 1H), 4.50 (p, J = 6.0 Hz, 1H), 1.39 (s, 3H), 1.38 (s, 3H), 1.33 (s, 3H), 1.32 (s, 3H).

^{13}C NMR (151 MHz, CDCl$_3$): δ 157.2, 153.7, 145.6, 143.0, 139.3, 134.3, 133.1, 132.7, 130.9, 130.3, 130.1, 129.4, 128.6, 128.3, 128.3, 128.0, 127.8, 127.1, 126.8, 126.4, 123.9, 116.3, 115.3, 114.3, 112.1, 104.9, 71.6, 70.3, 22.3, 22.1.

HRMS (ESI): m/z calcd for $C_{36}H_{32}N_2O_4Na^+$ $(M+Na)^+$ 579.22543, found 579.22577.

2-(naphthalen-2-yl)-3-(4-nitrophenyl)-1-phenyl-1H-indole (**5c**)

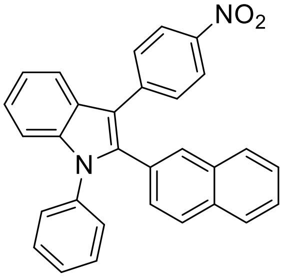

Compound **5c** was synthesized according to general procedure 3.3 starting from diphenylamine, 4-nitrophenylglyoxal and naphthalen-2-ylboronic acid. Compound **5c** was obtained in 20% yield (17.8 mg) as a yellow solid.

^{1}H NMR (600 MHz, $CDCl_3$): δ 8.05 (d, J = 8.7 Hz, 2H), 7.78 – 7.73 (m, 1H), 7.68 (d, J = 8.1 Hz, 1H), 7.56 (d, J = 8.6 Hz, 1H), 7.52 – 7.47 (m, 2H), 7.43 (d, J = 8.4 Hz, 2H), 7.39 (t, J = 7.5 Hz, 1H), 7.34 (t, J = 7.5 Hz, 1H), 7.28 (d, J = 7.1 Hz, 2H), 7.22 (dd, J = 10.6 Hz, 4.7 Hz, 3H), 7.18 (d, J = 8.0 Hz, 3H), 7.07 (d, J = 8.4 Hz, 1H).

^{13}C NMR (151 MHz, $CDCl_3$): δ 145.8, 142.7, 138.6, 138.4, 137.6, 133.1, 132.7, 131.0, 130.5, 129.5, 128.4, 128.3, 128.3, 128.2, 128.1, 127.9, 127.8, 126.9, 126.5, 123.9, 123.6, 121.9, 119.2, 115.1, 111.3.

HRMS (ESI): m/z calcd for $C_{30}H_{21}N_2O_2^+$ $(M+H)^+$ 441.15975, found 441.15979.

5,7-dimethyl-2-(naphthalen-2-yl)-3-(4-nitrophenyl)-1-phenyl-1H-indole (**5d**)

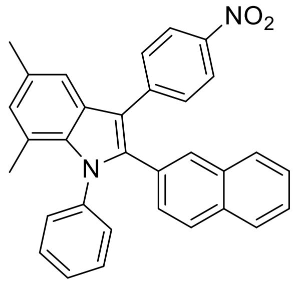

Compound **5d** was synthesized according to general procedure 3.3 starting from 2,4-dimethyl-N-phenylaniline, 4-nitrophenylglyoxal and naphthalen-2-ylboronic acid. Compound **5d** was obtained in 32% yield (30.3 mg) as a yellow solid.

^{1}H NMR (600 MHz, CDCl$_3$): δ 8.14 (d, J = 8.8 Hz, 2H), 7.87 (d, J = 7.1 Hz, 1H), 7.77 (d, J = 8.1 Hz, 1H), 7.65 (d, J = 8.5 Hz, 1H), 7.61 (d, J = 7.3 Hz, 2H), 7.55 (d, J = 8.8 Hz, 2H), 7.47 (t, J = 6.8 Hz, 1H), 7.43 (t, J = 6.9 Hz, 1H), 7.31 – 7.27 (m, 2H), 7.21 (d, J = 7.4 Hz, 2H), 7.04 (dd, J = 12.8 Hz, 5.7 Hz, 3H), 2.32 (s, 3H), 1.89 (s, 3H).

^{13}C NMR (151 MHz, CDCl$_3$): δ 145.6, 143.0, 139.2, 138.7, 138.5, 136.5, 133.9, 133.0, 132.7, 131.9, 130.6, 130.4, 129.6, 128.6, 128.3, 128.2, 128.0, 127.8, 127.6, 126.8, 126.7, 126.4, 123.9, 123.4, 121.6, 119.1, 114.4, 111.5, 21.3, 17.7.

HRMS (ESI): m/z calcd for $C_{32}H_{24}N_2O_2Na^+$ $(M+Na)^+$ 491.17300, found 491.17340.

5-methoxy-7-methyl-2-(naphthalen-2-yl)-3-(4-nitrophenyl)-1-phenyl-1H-indole (**5e**)

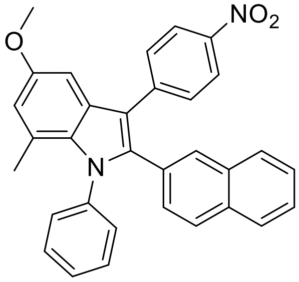

Compound **5e** was synthesized according to general procedure 3.3 starting from 4-methoxy-2-methyl-N-phenylaniline, 4-nitrophenylglyoxal and naphthalen-2-ylboronic acid. Compound **5e** was obtained in 50% yield (48.3 mg) as a yellow solid.

^{1}H NMR (600 MHz, CDCl$_3$): δ 8.14 (d, J = 8.9 Hz, 2H), 7.87 (d, J = 6.8 Hz, 1H), 7.77 (d, J = 8.1 Hz, 1H), 7.66 (d, J = 8.5 Hz, 1H), 7.61 (d, J = 9.3 Hz, 2H), 7.55 (d, J = 8.5 Hz, 2H), 7.48 (t, J = 7.5 Hz, 1H), 7.44 – 7.42 (m, 1H), 7.29 (ddd, J = 6.5 Hz, 3.9 Hz, 1.4 Hz, 2H), 7.25 (d, J = 8.6 Hz, 1H), 7.21 (d, J = 8.4 Hz, 1H), 7.05 (d, J = 8.6 Hz, 1H), 6.77 (dd, J = 8.6 Hz, 2.9 Hz, 1H), 6.73 (d, J = 2.8 Hz, 1H), 3.78 (s, 3H), 1.89 (s, 3H).

^{13}C NMR (151 MHz, CDCl$_3$): δ 159.5, 145.6, 143.0, 139.3, 138.6, 138.24, 133.0, 132.7, 130.8, 130.6, 130.4, 129.2, 128.6, 128.3, 128.2, 128.1, 127.8, 126.8, 126.6, 126.4, 123.9, 123.4, 121.6, 119.1, 116.1, 114.3, 112.2, 111.5, 55.5, 18.1.

HRMS (ESI): m/z calcd for $C_{32}H_{24}N_2O_3Na^+$ $(M+Na)^+$ 507.16791, found 507.16852.

1-benzyl-5-methoxy-2-(naphthalen-2-yl)-3-(4-nitrophenyl)-1H-indole (**5f**)

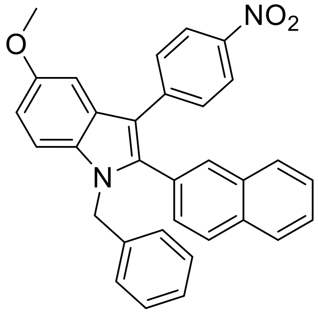

Compound **5f** was synthesized according to general procedure 3.3 starting from N-benzyl-4-methoxyaniline, 4-nitrophenylglyoxal and naphthalen-2-ylboronic acid. Compound **5f** was obtained in 50% yield (48.4 mg) as a yellow solid.

^{1}H NMR (600 MHz, CDCl$_3$): δ 8.09 (d, J = 8.8 Hz, 2H), 7.85 (d, J = 8.0 Hz, 1H), 7.79 (d, J = 8.4 Hz, 1H), 7.72 (s, 1H), 7.67 (d, J = 8.1 Hz, 1H), 7.56 – 7.52 (m, 1H), 7.52 – 7.48 (m,

1H), 7.46 (d, J = 8.8 Hz, 2H), 7.30 (dd, J = 5.0 Hz, 2.1 Hz, 2H), 7.27 (d, J = 7.5 Hz, 2H), 7.25 (s, 1H), 7.22 (d, J = 8.9 Hz, 1H), 7.01 (d, J = 6.5 Hz, 2H), 6.94 (dd, J = 8.9 Hz, 2.4 Hz, 1H), 5.31 (s, 2H), 3.88 (s, 3H).

^{13}C NMR (151 MHz, $CDCl_3$):δ 155.7, 145.4, 143.0, 140.1, 137.8, 133.2, 133.2, 132.6, 130.5, 129.8, 128.9, 128.7, 128.5, 128.3, 128.2, 128.0, 127.6, 127.2, 127.1, 126.8, 126.2, 123.9, 113.9, 113.2, 112.0, 101.2, 56.1, 48.1.

HRMS (ESI): m/z calcd for $C_{32}H_{25}N_2O_3^+$ $(M+H)^+$ 485.18597, found 485.18591.

5-methoxy-1-methyl-2-(naphthalen-2-yl)-3-(4-nitrophenyl)-1H-indole (**5g**)

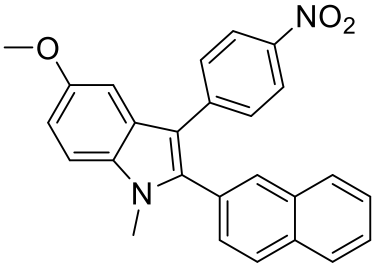

Compound **5g** was synthesized according to general procedure 3.3 starting from 4-methoxy-N-methylaniline, 4-nitrophenylglyoxal and naphthalen-2-ylboronic acid. Compound **5g** was obtained in 62% yield (50.4 mg) as a yellow solid.

^{1}H NMR (600 MHz, $CDCl_3$):δ 8.08 (d, J = 8.4 Hz, 2H), 7.91 (t, J = 9.3 Hz, 2H), 7.83 (d, J = 9.3 Hz, 2H), 7.57 (p, J = 6.9 Hz, 2H), 7.44 (d, J = 8.4 Hz, 2H), 7.38 (dd, J = 8.5 Hz, 4.4 Hz, 2H), 7.30 (s, 1H), 7.05 (dd, J = 8.8 Hz, 2.4 Hz, 1H), 3.91 (s, 3H), 3.72 (s, 3H).

^{13}C NMR (151 MHz, $CDCl_3$):δ 155.6, 145.2, 143.2, 139.9, 133.3, 133.1, 133.1, 130.5, 129.7, 128.7, 128.7, 128.3, 128.2, 128.0, 127.1, 126.9, 126.8, 123.9, 113.3, 113.0, 111.0, 101.1, 56.2, 31.4.

HRMS (ESI): m/z calcd for $C_{26}H_{21}N_2O_3^+$ $(M+H)^+$ 409.15467, found 409.15475.

7-methyl-2-(naphthalen-2-yl)-3-(4-nitrophenyl)-1-(o-tolyl)-1H-indole (**5h**)

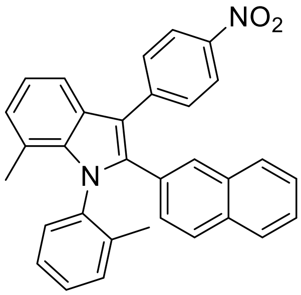

Compound **5h** was synthesized according to general procedure 3.3 starting from di-o-tolylamine, 4-nitrophenylglyoxal and naphthalen-2-ylboronic acid. Compound **5h** was obtained in 16% yield (15.4 mg) as a yellow solid.

^{1}H NMR (600 MHz, CDCl$_3$): δ 8.11 (d, J = 8.8 Hz, 2H), 7.75 (d, J = 9.0 Hz, 2H), 7.62 (d, J = 8.5 Hz, 1H), 7.60 (d, J = 8.2 Hz, 1H), 7.58 (s, 1H), 7.52 (d, J = 8.8 Hz, 2H), 7.48 (dd, J = 7.7 Hz, 1.5 Hz, 1H), 7.45 (dd, J = 8.1, 1.4 Hz, 1H), 7.43 – 7.40 (m, 1H), 7.24 (td, J = 7.5 Hz, 1.5 Hz, 1H), 7.22 – 7.17 (m, 3H), 7.10 (d, J = 7.5 Hz, 1H), 7.04 (d, J = 7.1 Hz, 1H), 1.91 (s, 3H), 1.90 (s, 3H).

^{13}C NMR (151 MHz, CDCl$_3$): δ 145.6, 143.0, 139.5, 138.6, 138.1, 136.1, 132.9, 132.7, 131.2, 130.9, 130.5, 130.3, 129.2, 128.6, 128.5, 128.2, 127.8, 127.8, 127.1, 126.8, 126.4, 126.2, 126.0, 123.8, 122.5, 121.5, 117.2, 114.9, 18.7, 17.7.

HRMS (APCI): m/z calcd for $C_{32}H_{25}N_2O_2^+$ $(M+H)^+$ 469.19105, found 469.19141.

3-(2-chloro-4-nitrophenyl)-5-methoxy-1-(4-methoxyphenyl)-2-(naphthalen-1-yl)-1H-indole (**5i**)

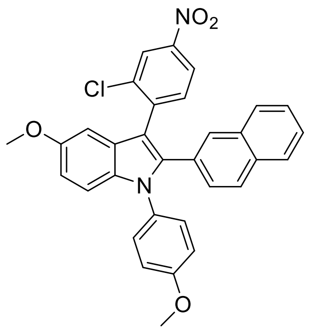

Compound **5i** was synthesized according to general procedure 3.3 starting from bis(4-methoxyphenyl)amine, 2-(2-chloro-4-nitrophenyl)-2-oxoacetaldehyde and naphthalen-2-ylboronic acid. Compound **5i** was obtained in 52% yield (55.6 mg) as a yellow solid.

^{1}H NMR (600 MHz, CDCl$_3$): δ 8.36 (s, 1H), 8.01 (dd, J = 8.5 Hz, 2.3 Hz, 1H), 7.74 (d, J = 8.1 Hz, 1H), 7.60 (d, J = 8.5 Hz, 1H), 7.58 (d, J = 8.2 Hz, 1H), 7.49 (s, 1H), 7.46 – 7.43 (m, 2H), 7.41 (t, J = 7.5 Hz, 1H), 7.24 (s, 1H), 7.22 (d, J = 7.7 Hz, 2H), 7.10 (d, J = 8.5 Hz, 1H), 6.94 (d, J = 8.8 Hz, 1H), 6.89 (d, J = 9.6 Hz, 3H), 3.85 (s, 3H), 3.80 (s, 3H).

^{13}C NMR (151 MHz, CDCl$_3$): δ 158.8, 155.4, 147.0, 142.1, 139.4, 135.7, 134.1, 133.6, 133.0, 132.5, 130.6, 130.2, 129.3, 128.7, 128.2, 127.9, 127.7, 127.3, 126.7, 126.4, 125.4, 121.6, 114.6, 113.5, 112.7, 112.1, 101.2, 56.0, 55.5.

HRMS (ESI): m/z calcd for $C_{32}H_{23}ClN_2O_4Na^+$ $(M+Na)^+$ 557.12386, found 557.12415.

3-(2-fluoro-4-nitrophenyl)-5-methoxy-1-(4-methoxyphenyl)-2-(naphthalen-1-yl)-1H-indole (**5j**)

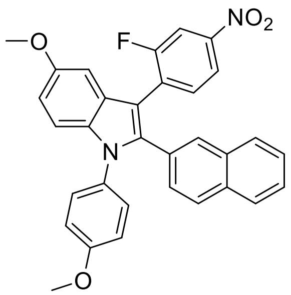

Compound **5j** was synthesized according to general procedure 3.3 starting from bis(4-methoxyphenyl)amine, 2-(2-fluoro-4-nitrophenyl)-2-oxoacetaldehyde and naphthalen-2-ylboronic acid. Compound **5j** was obtained in 56% yield (57.8 mg) as a yellow solid.

^{1}H NMR (600 MHz, CDCl$_3$): δ 7.99 (d, J = 9.5 Hz, 1H), 7.94 (dd, J = 8.5 Hz, 2.3 Hz, 1H), 7.77 (d, J = 8.1 Hz, 1H), 7.65 (d, J = 8.5 Hz, 1H), 7.61 (d, J = 8.1 Hz, 1H), 7.57 (s, 1H), 7.47 (d, J = 6.6 Hz, 1H), 7.45 (d, J = 7.3 Hz, 1H), 7.44 – 7.41 (m, 1H), 7.24 (d, J = 9.0 Hz, 1H), 7.20 (d, J = 8.6 Hz, 2H), 7.16 (d, J = 8.5 Hz, 1H), 7.06 (s, 1H), 6.96 (dd, J = 9.0 Hz, 2.4 Hz, 1H), 6.88 (d, J = 8.8 Hz, 2H), 3.89 (s, 3H), 3.80 (s, 3H).

^{13}C NMR (151 MHz, CDCl$_3$): δ 159.6 (d, J = 251.7 Hz), 158.9, 155.6, 146.9 (d, J = 8.4 Hz), 140.1, 133.9, 133.2 (d, J = 4.3 Hz), 133.0, 132.6, 131.0 (d, J = 16.0 Hz), 130.4, 130.3, 129.3, 128.6, 128.2, 128.0, 127.8, 127.3, 126.8, 126.4, 119.4 (d, J = 3.3 Hz), 114.6, 113.5, 112.2 (d, J = 27.7 Hz), 112.1, 108.6, 56.1, 55.5.

^{19}F NMR (565 MHz, CDCl$_3$): δ -107.84 (t, J = 8.5 Hz).

HRMS (ESI): m/z calcd for $C_{32}H_{23}FN_2O_4Na^+$ $(M+Na)^+$ 541.15341, found 541.15387.

5-methoxy-1-(4-methoxyphenyl)-2-(naphthalen-2-yl)-3-(4-nitro-3-(trifluoromethyl)phenyl)-1H-indole (**5k**)

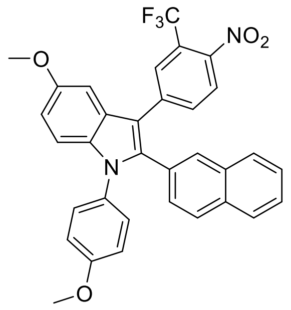

Compound **5k** was synthesized according to general procedure 3.3 starting from bis(4-methoxyphenyl)amine, 2-(4-nitro-3-(trifluoromethyl)phenyl)-2-oxoacetaldehyde and naphthalen-2-ylboronic acid. Compound **5k** was obtained in 40% yield (45.1 mg) as a yellow solid.

^{1}H NMR (600 MHz, $CDCl_3$): δ 7.95 (s, 1H), 7.80 (d, J = 3.9 Hz, 1H), 7.78 (d, J = 4.3 Hz, 1H), 7.70 (d, J = 8.5 Hz, 1H), 7.65 (d, J = 8.1 Hz, 1H), 7.60 (s, 1H), 7.52 – 7.49 (m, 2H), 7.48 (d, J = 8.7 Hz, 1H), 7.45 (d, J = 7.0 Hz, 1H), 7.22 (d, J = 8.9 Hz, 1H), 7.18 – 7.16 (m, 3H), 6.97 (dd, J = 8.9 Hz, 2.4 Hz, 1H), 6.86 (d, J = 8.8 Hz, 2H), 3.90 (s, 3H), 3.78 (s, 3H).
^{13}C NMR (151 MHz, $CDCl_3$): δ 159.0, 156.0, 145.0, 141.6, 139.8, 134.0, 133.3, 133.0, 132.8, 130.8, 130.1, 129.4, 128.6 (q, J = 5.5 Hz), 128.4, 128.3, 128.0 (d, J = 3.2 Hz), 127.8, 127.0, 126.6, 125.8, 124.2 (d, J = 33.6 Hz), 122.2 (d, J = 273.5 Hz), 114.6, 113.6, 113.0, 112.4, 100.2, 56.1, 55.5.
^{19}F NMR (565 MHz, $CDCl_3$): δ -60.01.
HRMS (ESI): m/z calcd for $C_{33}H_{23}F_3N_2O_4Na^+$ $(M+Na)^+$ 591.15021, found 591.15057.

3-(3-fluoro-4-nitrophenyl)-5-methoxy-1-(4-methoxyphenyl)-2-(naphthalen-1-yl)-1H-indole (**5l**)

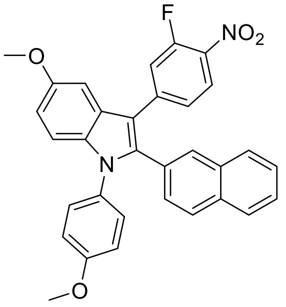

Compound **5l** was synthesized according to general procedure 3.3 starting from bis(4-methoxyphenyl)amine, 2-(3-fluoro-4-nitrophenyl)-2-oxoacetaldehyde and naphthalen-2-ylboronic acid. Compound **5l** was obtained in 49% yield (51.0 mg) as a yellow solid.

^{1}H NMR (600 MHz, CDCl$_3$):δ 7.95 (t, J = 8.2 Hz, 1H), 7.80 (d, J = 8.1 Hz, 1H), 7.70 (d, J = 8.5 Hz, 1H), 7.64 (d, J = 8.1 Hz, 1H), 7.60 (s, 1H), 7.50 (t, J = 7.4 Hz, 1H), 7.46 (t, J = 7.3 Hz, 1H), 7.32 (d, J = 12.3 Hz, 1H), 7.28 (d, J = 2.4 Hz, 1H), 7.21 (d, J = 8.9 Hz, 1H), 7.17 (dd, J = 13.9 Hz, 8.6 Hz, 4H), 6.96 (dd, J = 8.9 Hz, 2.3 Hz, 1H), 6.86 (d, J = 8.5 Hz, 2H), 3.91 (s, 3H), 3.78 (s, 3H).

^{13}C NMR (151 MHz, CDCl$_3$):δ 159.0, 156.0 (d, J = 264.1 Hz), 156.0, 144.8 (d, J = 9.3 Hz), 140.0, 134.4 (d, J = 6.8 Hz), 134.0, 133.0, 132.8, 130.9, 130.1, 129.4, 128.3 (d, J = 2.9 Hz), 128.1, 128.1, 127.8, 127.0, 126.7, 126.6, 126.4, 125.6 (d, J = 3.2 Hz), 118.5 (d, J = 21.1 Hz), 114.6, 113.5, 113.3, 112.3, 100.6, 56.2, 55.5.

^{19}F NMR (565 MHz, CDCl$_3$):δ -116.93 (dd, J = 12.4 Hz, 8.2 Hz).

HRMS (ESI): m/z calcd for $C_{32}H_{23}FN_2O_4Na^+$ $(M+Na)^+$ 541.15341, found 541.15393.

5-methoxy-1-(4-methoxyphenyl)-3-(3-methyl-4-nitrophenyl)-2-(naphthalen-1-yl)-1H-indole (**5m**)

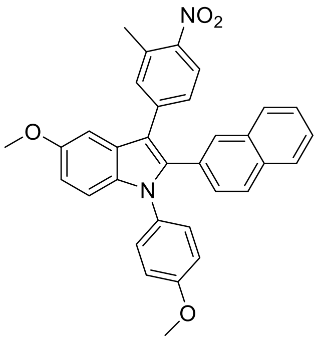

Compound **5m** was synthesized according to general procedure 3.3 starting from bis(4-methoxyphenyl)amine, 2-(3-methyl-4-nitrophenyl)-2-oxoacetaldehyde and naphthalen-2-ylboronic acid. Compound **5m** was obtained in 56% yield (57.8 mg) as a yellow solid.

^{1}H NMR (600 MHz, CDCl$_3$):δ 7.93 (d, J = 8.5 Hz, 1H), 7.78 (d, J = 8.1 Hz, 1H), 7.67 (d, J = 8.5 Hz, 1H), 7.63 (d, J = 8.1 Hz, 1H), 7.60 (s, 1H), 7.47 (t, J = 7.7 Hz, 1H), 7.44 (d, J = 9.6 Hz, 2H), 7.29 (d, J = 2.4 Hz, 1H), 7.23 (t, J = 8.7 Hz, 2H), 7.20 – 7.16 (m, 3H), 6.96 (dd, J = 8.9 Hz, 2.4 Hz, 1H), 6.86 (d, J = 8.8 Hz, 2H), 3.91 (s, 3H), 3.78 (s, 3H), 2.56 (s, 3H).
^{13}C NMR (151 MHz, CDCl$_3$):δ 158.8, 155.7, 146.5, 141.4, 139.1, 134.3, 134.0, 133.7, 133.0, 132.6, 130.8, 130.5, 129.4, 128.6, 128.4, 128.3, 128.2, 127.9, 127.8, 127.2, 126.7, 126.4, 125.3, 114.5, 114.3, 113.1, 112.1, 101.0, 56.2, 55.5, 21.2.
HRMS (ESI): m/z calcd for $C_{33}H_{26}N_2O_4Na^+$ $(M+Na)^+$ 537.17848, found 537.17896.

2-hydroxy-5-methoxy-1-(4-methoxyphenyl)-2-(4-nitrophenyl)indolin-3-one (**6**)

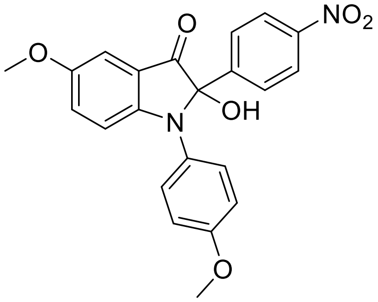

Compound **6** was synthesized according to general procedure 3.4 starting from bis(4-methoxyphenyl)amine and 4-nitrophenylglyoxal. Compound **6** was obtained in 62% yield (50.0 mg) as a orange solid.

^{1}H NMR (600 MHz, $CDCl_3$): δ 8.02 (d, J = 8.6 Hz, 2H), 7.56 (d, J = 8.6 Hz, 2H), 7.13 – 7.10 (m, 3H), 6.91 (d, J = 2.8 Hz, 1H), 6.82 (d, J = 8.9 Hz, 1H), 6.72 (d, J = 9.0 Hz, 2H), 5.09 (s, 1H), 3.70 (s, 3H), 3.68 (s, 3H).

^{13}C NMR (151 MHz, $CDCl_3$): δ 199.5, 157.7, 156.0, 153.5, 147.8, 144.5, 144.5, 130.6, 129.2, 127.4, 126.8, 123.6, 117.1, 114.6, 112.1, 105.9, 91.6, 55.8, 55.3.

HRMS (ESI): m/z calcd for $C_{22}H_{18}N_2O_6Na^+$ $(M+Na)^+$ 429.10571, found 429.10596.

5-methoxy-1-(4-methoxyphenyl)-2-(4-nitrophenyl)-2-phenylindolin-3-one (**7**)

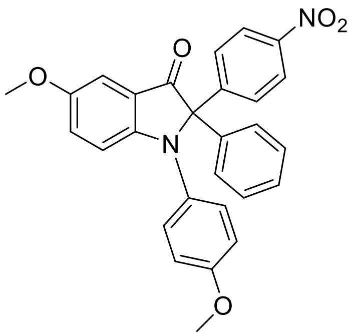

Compound **7** was synthesized according to general procedure 3.5 starting from **6** and **3a**. Compound **7** was obtained in 66% yield (30.2 mg) as a yellow solid.

^{1}H NMR (600 MHz, DMSO-d$_6$):δ 8.20 (d, J = 8.4 Hz, 2H), 7.55 (d, J = 8.4 Hz, 2H), 7.31 (d, J = 6.4 Hz, 4H), 7.12 (d, J = 8.3 Hz, 2H), 7.07 (s, 1H), 6.94 (d, J = 9.0 Hz, 1H), 6.88 (d, J = 8.5 Hz, 2H), 6.74 (d, J = 8.4 Hz, 2H), 3.77 (s, 2H), 3.64 (s, 2H).

^{13}C NMR (151 MHz, CDCl$_3$):δ 200.3, 158.0, 157.0, 154.1, 147.6, 146.6, 138.0, 132.9, 130.1, 129.1, 129.0, 128.6, 128.0, 123.5, 119.4, 114.5, 113.8, 105.0, 83.2, 56.0, 55.5.

HRMS (ESI): m/z calcd for $C_{28}H_{22}N_2O_5Na^+$ $(M+Na)^+$ 489.14209, found 489.14261.

5-methoxy-1-(4-methoxyphenyl)-2,2-diphenylindolin-3-one (**8a**)

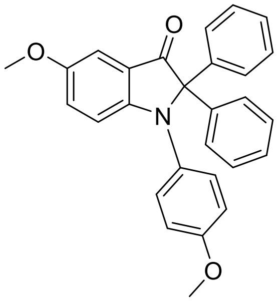

Compound **8a** was synthesized according to general procedure 3.6 starting from bis(4-methoxyphenyl)amine, 2-oxo-2-phenylacetaldehyde and phenylboronic acid. Compound **8a** was obtained in 51% yield (42.8 mg) as a yellow solid.

^{1}H NMR (600 MHz, CDCl$_3$):δ 7.32 (dd, J = 6.8, 3.0 Hz, 4H), 7.25 – 7.24 (m, 6H), 7.16 (dd, J = 9.0, 2.8 Hz, 1H), 7.11 (d, J = 2.7 Hz, 1H), 6.92 – 6.89 (m, 3H), 6.64 (d, J = 9.0 Hz, 2H), 3.80 (s, 3H), 3.70 (s, 3H).

^{13}C NMR (151 MHz, CDCl$_3$):δ 201.7, 157.5, 156.7, 153.5, 138.8, 133.4, 129.2, 128.4, 128.3, 128.0, 128.0, 119.6, 114.2, 113.3, 105.0, 83.7, 55.9, 55.4.

HRMS (ESI): m/z calcd for $C_{28}H_{24}NO_3^+$ $(M+H)^+$ 422.17507, found 422.17474.

2-(4-fluorophenyl)-5-methoxy-1-(4-methoxyphenyl)-2-phenylindolin-3-one (**8b**)

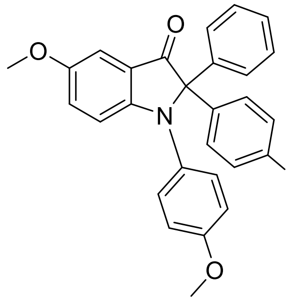

Compound **8b** was synthesized according to general procedure 3.6 starting from bis(4-methoxyphenyl)amine, 2-(4-fluorophenyl)-2-oxoacetaldehyde and phenylboronic acid. Compound **8b** was obtained in 46% yield (40.0 mg) as a yellow solid.

^{1}H NMR (600 MHz, $CDCl_3$): δ 7.29 (dd, J = 6.8, 3.0 Hz, 2H), 7.25 – 7.23 (m, 5H), 7.14 (dd, J = 9.0, 2.7 Hz, 1H), 7.08 (d, J = 2.7 Hz, 1H), 6.90 (t, J = 8.7 Hz, 2H), 6.88 – 6.85 (m, 3H), 6.63 (d, J = 8.9 Hz, 2H), 3.77 (s, 3H), 3.69 (s, 3H).

^{13}C NMR (151 MHz, $CDCl_3$): δ 201.6, 162.5 (d, J = 247.3 Hz), 157.7, 156.8, 153.7, 138.7, 134.7 (d, J = 3.2 Hz), 133.3, 131.1 (d, J = 8.1 Hz), 129.0, 128.6, 128.4, 128.2, 128.0, 119.5, 115.2 (d, J = 21.3 Hz), 114.3, 113.5, 105.0, 83.1, 55.9, 55.4.

^{19}F NMR (565 MHz, $CDCl_3$): δ -114.03 (p, J = 6.9 Hz).

HRMS (ESI): m/z calcd for $C_{28}H_{23}FNO_3^+$ $(M+H)^+$ 440.16565, found 440.16541.

2-(4-chlorophenyl)-5-methoxy-1-(4-methoxyphenyl)-2-phenylindolin-3-one (**8c**)

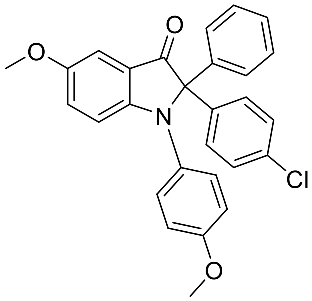

Compound **8c** was synthesized according to general procedure 3.6 starting from bis(4-methoxyphenyl)amine, 2-(4-chlorophenyl)-2-oxoacetaldehyde and phenylboronic acid. Compound **8c** was obtained in 41% yield (37.1 mg) as a yellow solid.

^{1}H NMR (600 MHz, $CDCl_3$): δ 7.2 – 7.20 (m, 9H), 7.15 (dd, J = 9.0, 2.8 Hz, 1H), 7.09 (d, J = 2.7 Hz,1H), 6.87 (dd, J = 9.0, 3.7 Hz, 3H), 6.64 (d, J = 8.9 Hz, 2H), 3.79 (s, 3H), 3.70 (s, 3H).

^{13}C NMR (151 MHz, $CDCl_3$): δ 201.2, 157.7, 156.7, 153.7, 138.5, 137.5, 134.1, 133.2, 130.6, 129.1, 128.6, 128.5, 128.4, 128.2, 128.0, 119.5, 114.3, 113.5, 105.0, 83.1, 56.0, 55.4.

HRMS (ESI): m/z calcd for $C_{28}H_{23}ClNO_3^+$ $(M+H)^+$ 456.13610, found 456.13593.

2-(4-bromophenyl)-5-methoxy-1-(4-methoxyphenyl)-2-phenylindolin-3-one (**8d**)

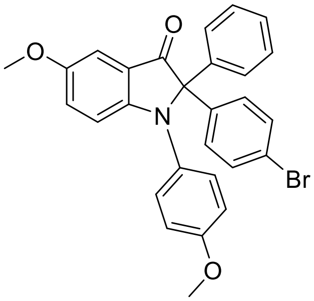

Compound **8d** was synthesized according to general procedure 3.6 starting from bis(4-methoxyphenyl)amine, 2-(4-bromophenyl)-2-oxoacetaldehyde and phenylboronic acid. Compound **8d** was obtained in 38% yield (38.4 mg) as a yellow solid.

^{1}H NMR (600 MHz, $CDCl_3$): δ 7.37 (d, J = 8.6 Hz, 2H), 7.25 – 7.23 (m, 5H), 7.20 (d, J = 8.6 Hz, 2H), 7.15 (dd, J = 9.0, 2.7 Hz, 1H), 7.09 (d, J = 2.7 Hz, 1H), 6.87 (dd, J = 9.1, 3.3 Hz, 3H), 6.64 (d, J = 9.0 Hz, 2H), 3.80 (s, 3H), 3.71 (s, 3H).

^{13}C NMR (151 MHz, $CDCl_3$): δ 201.2, 157.8, 156.8, 153.8, 138.5, 138.1, 133.2, 131.4, 131.0, 129.1, 128.6, 128.4, 128.3, 128.0, 122.4, 119.5, 114.4, 113.5, 105.1, 83.2, 56.0, 55.5.

HRMS (ESI): m/z calcd for $C_{28}H_{23}BrNO_3^+$ $(M+H)^+$ 500.0855827, found 500.08569.

5-methoxy-1-(4-methoxyphenyl)-2-phenyl-2-(4-(trifluoromethyl)phenyl)indolin-3-one (**8e**)

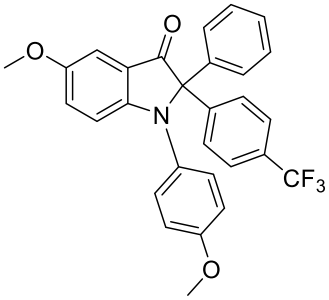

Compound **8e** was synthesized according to general procedure 3.6 starting from bis(4-methoxyphenyl)amine, 2-oxo-2-(4-(trifluoromethyl)phenyl)acetaldehyde and phenylboronic acid. Compound **8e** was obtained in 50% yield (49.1 mg) as a yellow solid.

^{1}H NMR (600 MHz, CDCl$_3$): δ 7.53 (d, J = 8.6 Hz, 2H), 7.50 (d, J = 8.6 Hz, 2H), 7.25 – 7.21 (m, 3H), 7.20 – 7.19 (m, 2H), 7.17 (dd, J = 9.0, 2.8 Hz, 1H), 7.10 (d, J = 2.7 Hz, 1H), 6.87 (dd, J = 9.0, 7.1 Hz, 3H), 6.64 (d, J = 9.0 Hz, 2H), 3.80 (s, 3H), 3.71 (s, 3H).

^{13}C NMR (151 MHz, CDCl$_3$): δ 200.9, 157.8, 156.9, 153.9, 143.1, 138.4, 133.1, 130.20 (d, J = 32.6 Hz), 129.5, 129.2, 128.8, 128.5, 128.4, 128.1, 125.3 (q, J = 3.7 Hz), 124.17 (d, J = 272.1 Hz), 119.5, 114.4, 113.6, 106.0, 83.3, 56.0, 55.5.

^{19}F NMR (565 MHz, CDCl$_3$): δ -62.57.

HRMS (ESI): m/z calcd for $C_{29}H_{23}F_3NO_3^+$ $(M+H)^+$ 490.16245, found 490.16260.

5-methoxy-1-(4-methoxyphenyl)-2-phenyl-2-(4-(trifluoromethoxy)phenyl)indolin-3-one (**8f**)

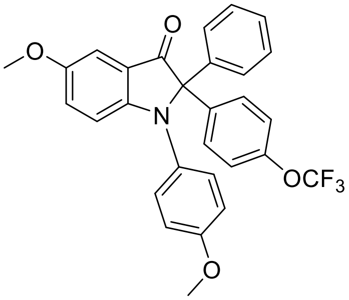

Compound **8f** was synthesized according to general procedure 3.6 starting from bis(4-methoxyphenyl)amine, 2-oxo-2-(4-(trifluoromethoxy)phenyl)acetaldehyde and phenylboronic acid. Compound **8f** was obtained in 52% yield (52.5 mg) as a yellow solid.

^{1}H NMR (600 MHz, CDCl$_3$):δ 7.35 (d, J = 8.8 Hz, 2H), 7.24 (d, J = 5.0 Hz, 5H), 7.15 (dd, J = 9.0, 2.8 Hz, 1H), 7.08 (d, J = 8.0 Hz, 3H), 6.86 (d, J = 8.9 Hz, 3H), 6.63 (d, J = 8.9 Hz, 2H), 3.79 (s, 3H), 3.69 (s, 3H).

^{13}C NMR (151 MHz, CDCl$_3$):δ 201.3, 157.8, 156.9, 153.8, 149.0, 138.6, 137.7, 133.2, 130.7, 129.1 128.7, 128.4, 128.3, 128.1, 120.62, 120.52 (d, J = 257.5 Hz), 119.5, 114.4, 113.6, 105.0, 83.1, 56.0, 55.4.

^{19}F NMR (565 MHz, CDCl$_3$):δ -57.76.

HRMS (ESI): m/z calcd for $C_{29}H_{23}F_3NO_4^+$ $(M+H)^+$ 506.15737, found 506.15753.

5-methoxy-1,2-bis(4-methoxyphenyl)-2-phenylindolin-3-one (**8g**)

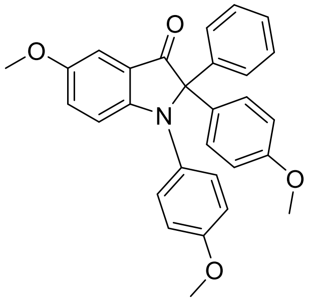

Compound **8g** was synthesized according to general procedure 3.6 starting from bis(4-methoxyphenyl)amine, 2-(4-methoxyphenyl)-2-oxoacetaldehyde and phenylboronic acid. Compound **8g** was obtained in 45% yield (41.4 mg) as a yellow solid.

^{1}H NMR (600 MHz, CDCl$_3$): δ 7.34 – 7.32 (m, 2H), 7.25 – 7.23 (m, 3H), 7.20 (d, J = 8.9 Hz, 2H), 7.14 (dd, J = 9.0, 2.7 Hz, 1H), 7.10 (d, J = 2.7 Hz, 1H), 6.90 (d, J = 8.9 Hz, 2H), 6.88 (d, J = 9.0 Hz, 1H), 6.76 (d, J = 8.9 Hz, 2H), 6.64 (d, J = 9.0 Hz, 2H), 3.79 (s, 3H), 3.76 (s, 3H), 3.70 (s, 3H).

^{13}C NMR (151 MHz, CDCl$_3$): δ 201.9, 159.3, 157.5, 156.6, 153.5, 139.0, 133.5, 130.8, 130.5, 129.1, 128.3, 128.3, 128.0, 127.9, 119.6, 114.2, 113.7, 113.3, 105.1, 83.2, 56.0, 55.4, 55.3.

HRMS (ESI): m/z calcd for $C_{29}H_{26}NO_4^+$ $(M+H)^+$ 452.18563, found 452.18533.

5-methoxy-1-(4-methoxyphenyl)-2-phenyl-2-(3-(trifluoromethoxy)phenyl)indolin-3-one (**8h**)

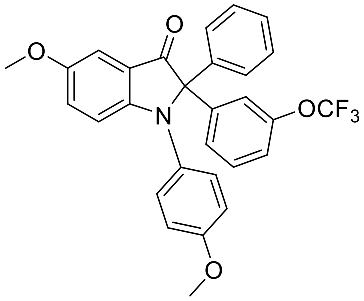

Compound **8h** was synthesized according to general procedure 3.6 starting from bis(4-methoxyphenyl)amine, 2-oxo-2-(3-(trifluoromethoxy)phenyl)acetaldehyde and phenylboronic acid. Compound **8h** was obtained in 36% yield (36.0 mg) as a yellow solid.

^{1}H NMR (600 MHz, CDCl$_3$): δ 7.25 – 7.24 (m,7H), 7.15 – 7.13 (m, 2H), 7.10 – 7.07 (m, 2H), 6.85 (dd, J = 8.9, 6.1 Hz, 3H), 6.61 (d, J = 8.9 Hz, 2H), 3.78 (s, 3H), 3.68 (s, 3H).

^{13}C NMR (151 MHz, CDCl$_3$):δ 201.0, 157.9, 156.9, 153.9, 141.5, 138.3, 133.2, 129.7, 129.1, 128.7, 128.5, 128.4, 128.1, 127.7, 122.3, 120.7, 120.5 (d, J = 257.4 Hz), 119.6, 114.4, 113.6, 105.1, 83.2, 56.0, 55.5.

^{19}F NMR (565 MHz, CDCl$_3$):δ -57.83.

HRMS(ESI): m/z calcd for $C_{29}H_{23}F_3NO_4^+$ $(M+H)^+$ 506.15737, found 506.15753.

5-isopropoxy-1-(4-isopropoxyphenyl)-2-(naphthalen-2-yl)-2-phenylindolin-3-one (**8i**)

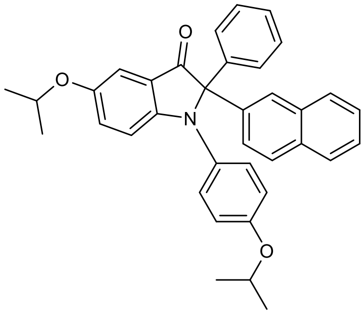

Compound **8i** was synthesized according to general procedure 3.6 starting from **9**, 2-oxo-2-phenylacetaldehyde and naphthalen-2-ylboronic acid. Compound **8i** was obtained in 48% yield (50.2 mg) as a yellow solid.

^{1}H NMR (600 MHz, DMSO-d$_6$):δ 7.85 (d, J = 11.1 Hz, 3H), 7.80 (d, J = 8.8 Hz, 1H), 7.48 (dt, J = 19.5, 7.2 Hz, 2H), 7.30 (s, 3H), 7.24 (d, J = 7.1 Hz, 4H), 7.04 (s, 1H), 6.98 (d, J = 8.9 Hz, 1H), 6.90 (d, J = 8.4 Hz, 2H), 6.65 (d, J = 8.4 Hz, 2H), 4.54 (p, J = 6.1 Hz, 1H), 4.39 (p, J = 6.1 Hz, 1H), 1.25 (d, J = 6.0 Hz, 6H), 1.11 (d, J = 4.2 Hz, 6H).

^{13}C NMR (151 MHz, CDCl$_3$):δ 201.6, 156.6, 155.9, 151.7, 139.0, 136.3, 133.3, 133.1, 133.0, 130.0, 129.4, 128.4, 128.3, 128.3, 128.1, 128.0, 127.9, 127.6, 127.1, 126.4, 126.1, 119.9, 116.4, 113.4, 108.0, 83.7, 71.0, 70.3, 22.2, 22.0.

HRMS (ESI): m/z calcd for $C_{36}H_{34}NO_3^+$ $(M+H)^+$ 528.25332, found 528.25323.

5-methyl-2-(naphthalen-2-yl)-2-phenyl-1-(p-tolyl)indolin-3-one (**8j**)

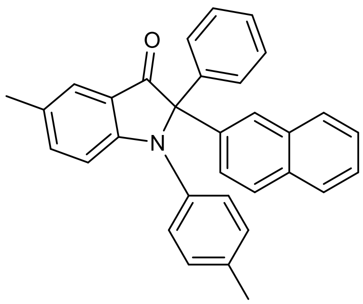

Compound **8j** was synthesized according to general procedure 3.6 starting from di-p-tolylamine, 2-oxo-2-phenylacetaldehyde and naphthalen-2-ylboronic acid. Compound **8j** was obtained in 42% yield (37.0 mg) as a yellow solid.

^{1}H NMR (600 MHz, DMSO-d$_6$): δ 7.88 (s, 1H), 7.85 (d, J = 8.2 Hz, 2H), 7.81 (d, J = 8.7 Hz, 1H), 7.50 (t, J = 7.4 Hz, 1H), 7.46 (dd, J = 8.2, 3.7 Hz, 2H), 7.41 (s, 1H), 7.31 – 7.27 (m, 6H), 7.10 (d, J = 8.5 Hz, 1H), 6.96 (d, J = 7.8 Hz, 2H), 6.92 (d, J = 8.1 Hz, 2H), 2.29 (s, 3H), 2.10 (s, 3H).

^{13}C NMR (151 MHz, CDCl$_3$): δ 201.4, 158.7, 139.1, 138.6, 137.7, 136.0, 135.3, 133.1, 133.0, 129.6, 129.4, 129.2, 128.4, 128.3, 128.1, 128.0, 127.6, 127.0, 126.4, 126.1, 125.6, 125.1, 120.1, 112.1, 83.2, 21.0, 20.6.

HRMS (ESI): m/z calcd for $C_{32}H_{26}NO^+$ $(M+H)^+$ 440.20089, found 440.20084.

2-(4-fluorophenyl)-5-methoxy-1-(4-methoxyphenyl)-2-phenylindolin-3-one (**8k**)

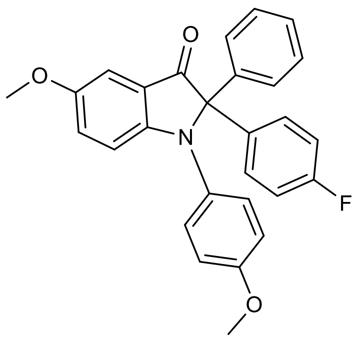

Compound **8k** was synthesized according to general procedure 3.6 starting from bis(4-methoxyphenyl)amine, 2-oxo-2-phenylacetaldehyde and (4-fluorophenyl)boronic acid. Compound **8k** was obtained in 41% yield (36.1 mg) as a yellow solid.

^{1}H NMR (600 MHz, CDCl$_3$): δ 7.30 – 7.29 (m, 2H), 7.25 – 7.24 (m, 5H), 7.15 (dd, J = 9.0, 1.8 Hz, 1H), 7.09 (d, J = 2.7 Hz, 1H), 6.91 (t, J = 8.5 Hz, 2H), 6.87 (dd, J = 8.8, 6.4 Hz, 3H), 6.63 (d, J = 9.3 Hz, 2H), 3.79 (s, 3H), 3.70 (s, 3H).

^{13}C NMR (151 MHz, CDCl$_3$): δ 201.5, 162.6 (d, J = 247.2 Hz), 157.7, 156.8, 153.7, 138.8, 134.78 (d, J = 3.3 Hz), 133.3, 131.1 (d, J = 8.1 Hz), 129.1, 128.6, 128.4, 128.2, 128.0, 119.5, 115.18 (d, J = 21.5 Hz), 114.3, 113.5, 105.1, 83.1, 56.0, 55.4.

^{19}F NMR (565 MHz, CDCl$_3$): δ -114.10 (p, J = 7.2, 6.3 Hz).

HRMS (ESI): m/z calcd for $C_{28}H_{23}FNO_3^+$ $(M+H)^+$ 440.16565, found 440.16541.

2-(4-chlorophenyl)-5-methoxy-1-(4-methoxyphenyl)-2-phenylindolin-3-one (**8l**)

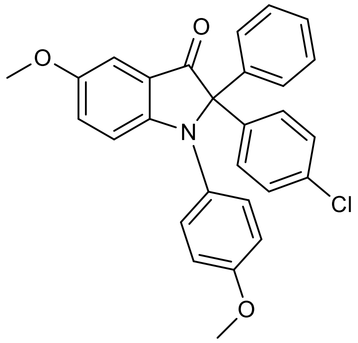

Compound **8l** was synthesized according to general procedure 3.6 starting from bis(4-methoxyphenyl)amine, 2-oxo-2-phenylacetaldehyde and (4-chlorophenyl)boronic acid. Compound **8l** was obtained in 30% yield (27.6 mg) as a yellow solid.

^{1}H NMR (600 MHz, CDCl$_3$):δ 7.31 – 7.27 (m, 9H), 7.21 (dd, J = 9.0, 2.7 Hz, 1H), 7.14 (d, J = 2.7 Hz, 1H), 6.93 (dd, J = 9.0, 3.6 Hz, 3H), 6.69 (d, J = 8.9 Hz, 2H), 3.85 (s, 3H), 3.76 (s, 3H).

^{13}C NMR (151 MHz, CDCl$_3$):δ 200.1, 156.6, 155.6, 152.6, 137.4, 136.4, 133.0, 132.1, 129.5, 128.0, 127.5, 127.3, 127.3, 127.1, 126.9, 118.4, 113.2, 112.3, 103.9, 82.0, 54.8, 54.3.

HRMS (ESI): m/z calcd for $C_{28}H_{23}ClNO_3^+$ $(M+H)^+$ 456.13610, found 456.13586.

5-methoxy-1-(4-methoxyphenyl)-2-phenyl-2-(m-tolyl)indolin-3-one (**8m**)

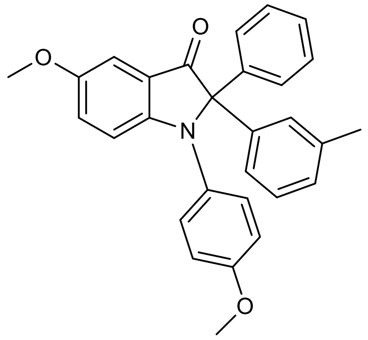

Compound **8m** was synthesized according to general procedure 3.6 starting from bis(4-methoxyphenyl)amine, 2-oxo-2-phenylacetaldehyde and m-tolylboronic acid. Compound **8m** was obtained in 43% yield (37.1 mg) as a yellow solid.

^{1}H NMR (600 MHz, CDCl$_3$):δ 7.30 (dd, J = 7.3, 2.6 Hz, 2H), 7.24 – 7.22 (m, 3H), 7.15 (dd, J = 8.9, 2.7 Hz, 1H), 7.12 (d, J = 7.4 Hz, 4H), 7.06 (d, J = 6.5 Hz, 1H), 6.89 (t, J = 9.0 Hz, 3H), 6.63 (d, J = 8.9 Hz, 2H), 3.80 (s, 3H), 3.70 (s, 3H), 2.25 (s, 3H).

^{13}C NMR (151 MHz, CDCl$_3$):δ 201.8, 157.6, 156.7, 153.5, 139.0, 138.7, 137.9, 133.5, 129.8, 129.3, 128.9, 128.4, 128.3, 128.1, 128.1, 128.0, 126.4, 119.7, 114.2, 113.3, 105.1, 83.7, 56.0, 55.4, 21.6.

HRMS (ESI): m/z calcd for $C_{29}H_{26}NO_3^+$ $(M+H)^+$ 436.19072, found 436.19046.

5-methoxy-1-(4-methoxyphenyl)-2-(4-nitrophenyl)-2-(thiophen-3-yl)indolin-3-one (**8n**)

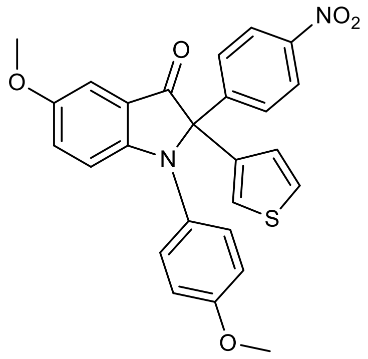

Compound **8n** was synthesized according to general procedure 3.7 starting from bis(4-methoxyphenyl)amine, 4-nitrophenylglyoxal and thiophen-3-ylboronic acid. Compound **8n** was obtained in 40% yield (38.2 mg) as a yellow solid.

^{1}H NMR (600 MHz, CDCl$_3$): δ 8.15 (d, J = 8.8 Hz, 2H), 7.65 (d, J = 8.4 Hz, 2H), 7.22 (d, J = 5.1 Hz, 1H), 7.20 (dd, J = 9.0, 2.8 Hz, 1H), 7.11 (d, J = 2.7 Hz, 1H), 6.92 (s, 1H), 6.91 – 6.90 (m, 2H), 6.87 (dd, J = 9.0, 3.9 Hz, 1H), 6.84 (d, J = 3.6 Hz, 1H), 6.70 (d, J = 8.9 Hz, 2H), 3.81 (s, 3H), 3.74 (s, 3H).

^{13}C NMR (151 MHz, CDCl$_3$): δ 198.5, 158.3, 156.7, 154.3, 147.9, 146.5, 140.3, 132.9, 129.6, 129.3, 129.1, 128.4, 126.9, 126.6, 123.6, 118.5, 114.6, 114.0, 105.1, 79.8, 56.0, 55.5.

HRMS (ESI): m/z calcd for $C_{26}H_{21}N_2O_5S^+$ $(M+H)^+$ 473.11657, found 473.11612.

1-benzyl-2-(4-bromophenyl)-2-(furan-3-yl)-5,6,7-trimethoxyindolin-3-one (**8o**)

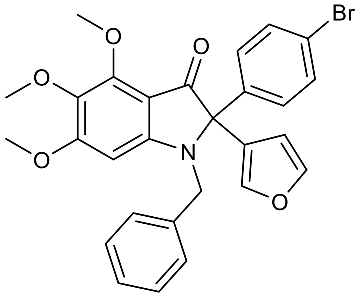

Compound **8o** was synthesized according to general procedure 3.7 starting from N-benzyl-3,4,5-trimethoxyaniline, 2-(4-bromophenyl)-2-oxoacetaldehyde and furan-3-ylboronic acid. Compound **8o** was obtained in 52% yield (55.5 mg) as a yellow solid.

^{1}H NMR (600 MHz, CDCl$_3$):δ 7.46 (d, J = 8.6 Hz, 2H), 7.24 (d, J = 1.8 Hz, 1H), 7.21 – 7.16 (m, 3H), 7.09 (d, J = 8.6 Hz, 2H), 6.89 (d, J = 6.8 Hz, 2H), 6.45 (d, J = 3.3 Hz, 1H), 6.33 (dd, J = 3.4, 1.8 Hz, 1H), 5.71 (s, 1H), 4.74 (d, J = 16.9 Hz, 1H), 4.66 (d, J = 16.9 Hz, 1H), 4.08 (s, 3H), 3.74 (s, 3H), 3.68 (s, 3H).

^{13}C NMR (151 MHz, CDCl$_3$):δ 191.7, 163.2, 158.6, 152.7, 151.1, 143.4, 137.4, 136.1, 133.7, 132.0, 129.1, 128.7, 127.1, 126.3, 122.6, 111.1, 110.5, 103.7, 86.7, 75.9, 61.9, 61.8, 56.2, 48.7.

HRMS (ESI): m/z calcd for $C_{28}H_{25}BrNO_5^+$ $(M+H)^+$ 534.09106, found 534.09119.

5. ^{11}B NMR spectroscopy experiments

Experiment 1: **1a** (0.2 mmol), **2a** (0.4 mmol), **3a** (0.3 mmol), Cu(TFA)$_2$·xH$_2$O (0.04 mmol), Co(salen) (0.04 mmol) and DCE (2.0 mL, *c* 0.1 M) were added to the reaction tube, mixed well by sonication but without heating. Then filtered through a 0.22 µm filter prior to the ^{11}B NMR analysis. The result is shown in the Figure S1.

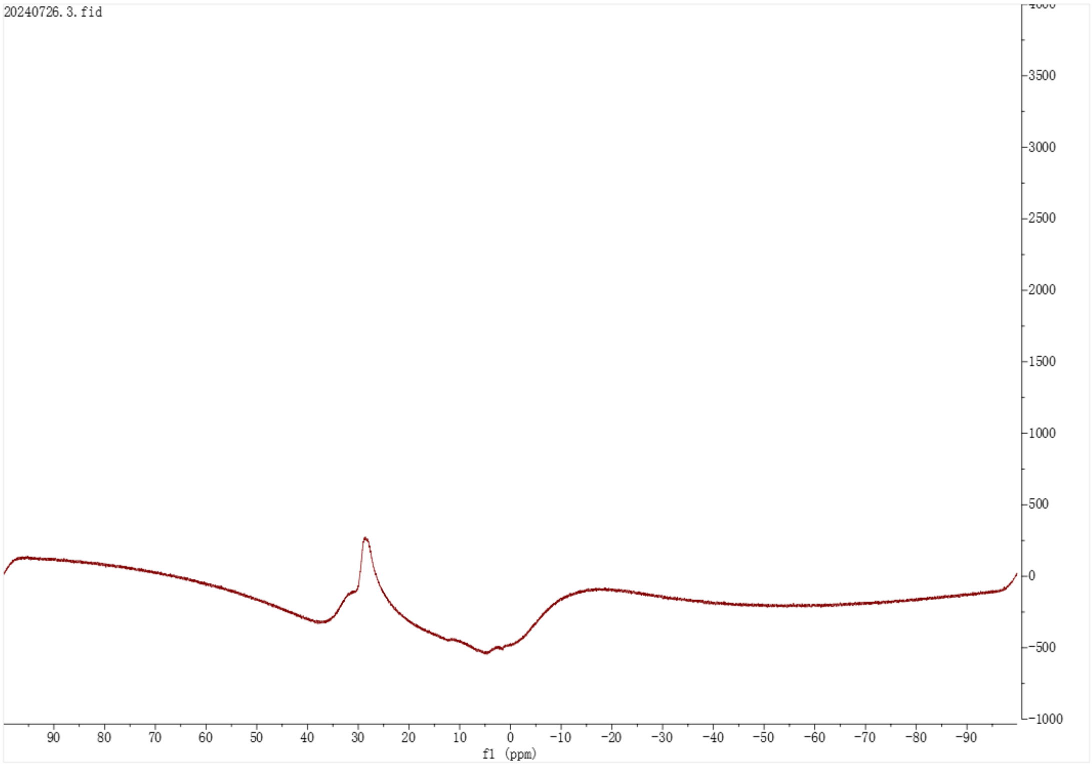

^{11}B NMR of experiment 1

Figure S1. A broad resonance in ^{11}B NMR at 28.6 ppm refers to a phenylboronic acid (**3a**)

Experiment 2: **1a** (0.2 mmol), **2a** (0.4 mmol), **3a** (0.3 mmol), $Cu(TFA)_2 \cdot xH_2O$ (0.04 mmol), Co(salen) (0.04 mmol) and DCE (2.0 mL, *c* 0.1 M) were sealed in a heavy-wall pressure vessel and heated with stirring at 80 °C for 2 h. At the end of the reaction, the reaction solution was filtered through a 0.22 μm filter and analysed by ^{11}B NMR. The result is shown in the Figure S2.

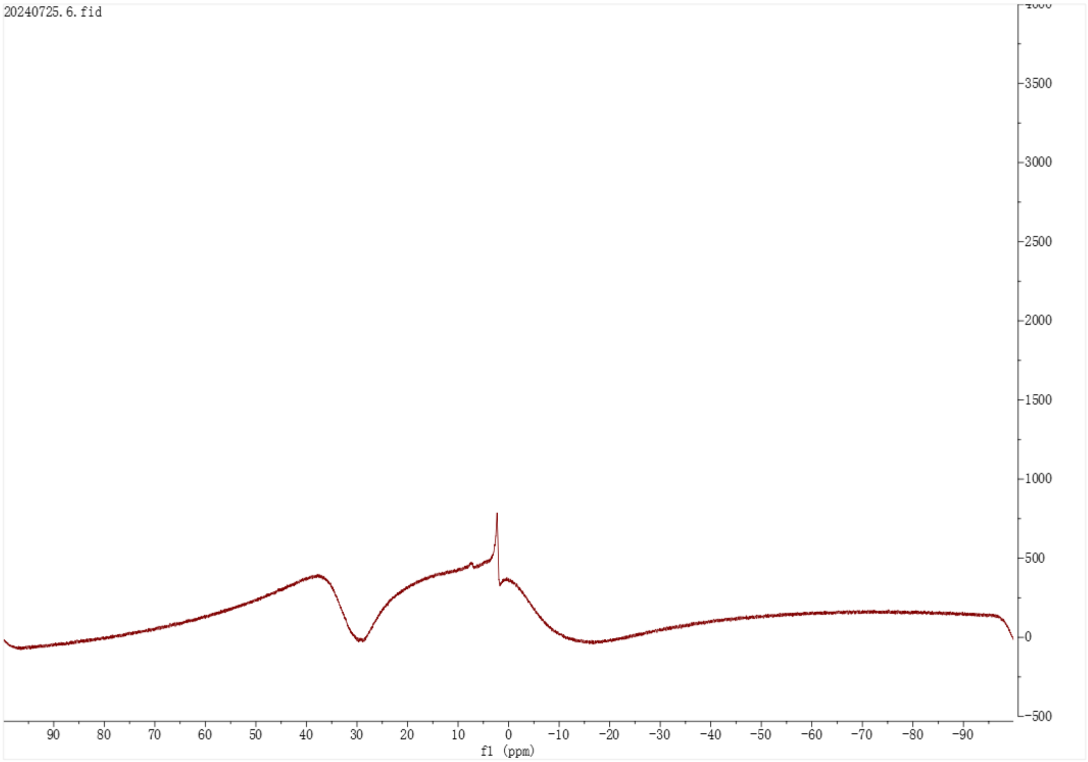

^{11}B NMR of experiment 2

Figure S2. A broad resonance in ^{11}B NMR at 2.1 ppm might refers to a boron-ate complex (**I-B**).

6. EPR experiment

1a (0.2 mmol), **2a** (0.4 mmol), **3a** (0.6 mmol), $Cu(TFA)_2 \cdot xH_2O$ (0.04 mmol), Co(salen) (0.04 mmol) and DCE (2.0 mL, *c* 0.1 M) were sealed in a heavy-wall pressure vessel and heated with stirring at 80 °C for 1 h. After cooling to room temperature, 50 μL of spin-trapping reagent 5,5-dimethyl-1-pyrroline N-oxide (DMPO) was added and stirred for two minutes. The result is shown in the Figure S3.

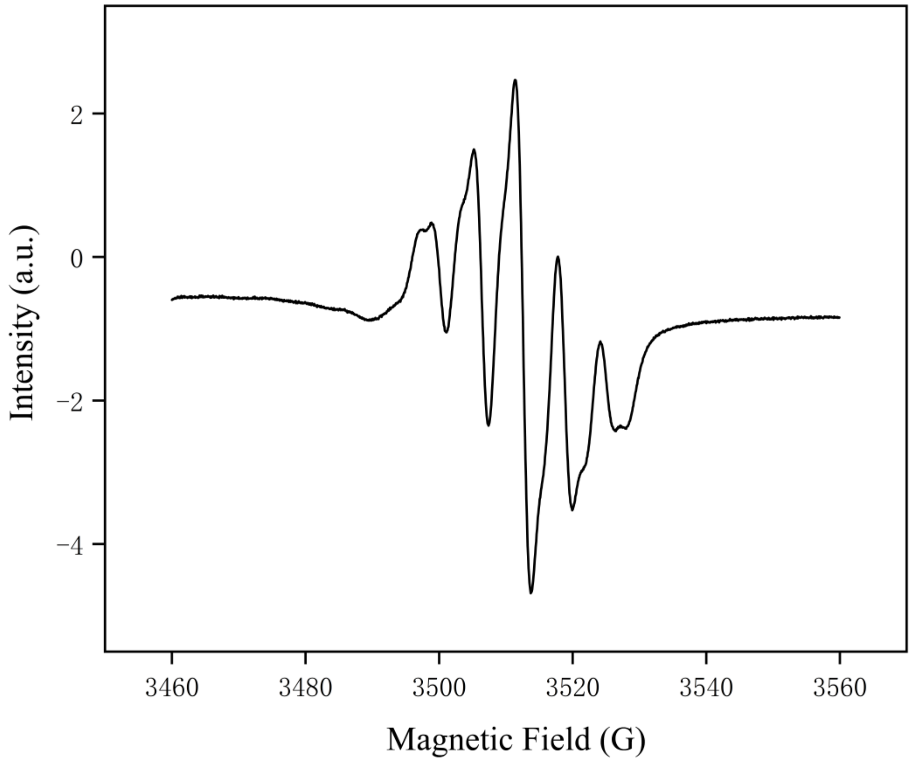

EPR spectrum at room temperature

Figure S3. EPR experiments have demonstrated the presence of radical species in the reaction. We believe that the radicals may be generated from the interaction of Co(salen) with oxygen in the air.

7. UV-vis absorption spectra of different components

1a (0.2 mmol), **2a** (0.4 mmol), **3a** (0.6 mmol), $Cu(TFA)_2 \cdot xH_2O$ (0.04 mmol), Co(salen) (0.04 mmol) and DCE (2.0 mL, *c* 0.1 M) were sealed in a heavy-wall pressure vessel and heated with stirring at 80 °C for 2 h. The reaction solution was used for UV-vis spectroscopic analysis. The result is shown in the Figure S4.

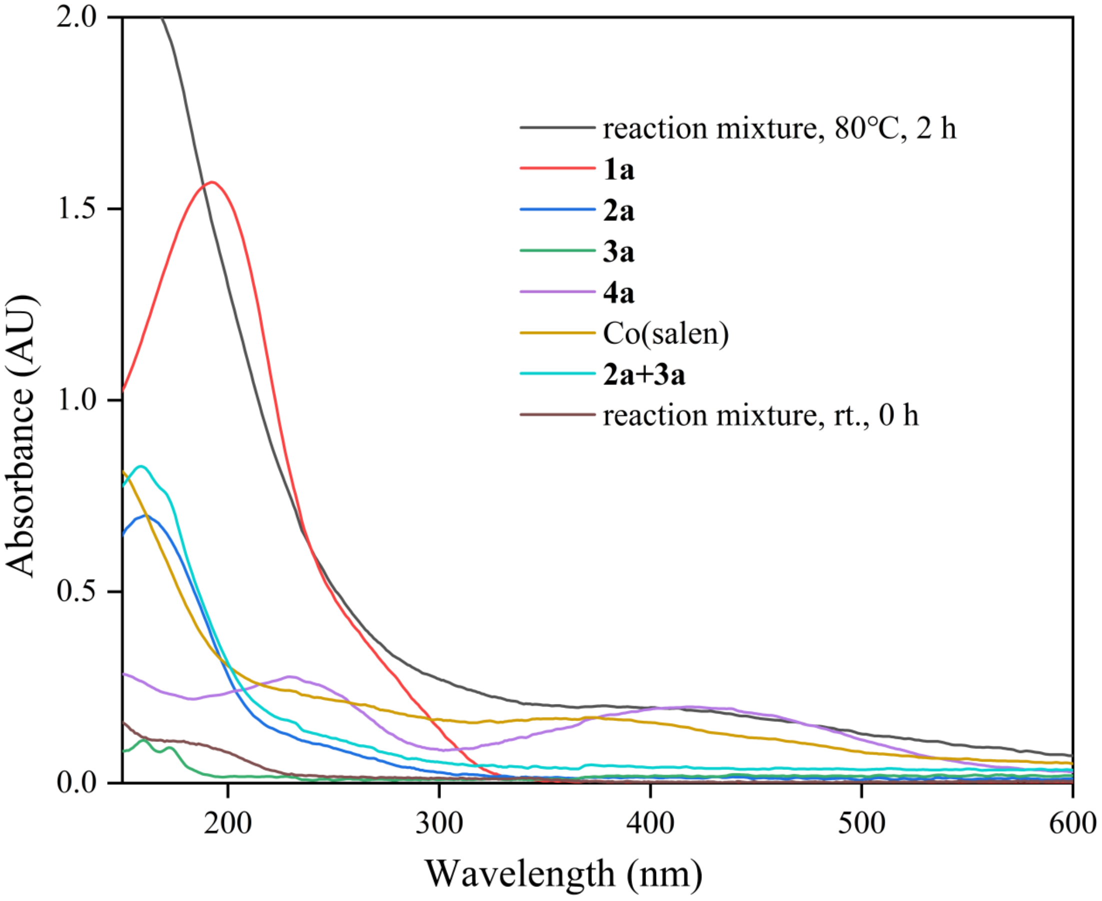

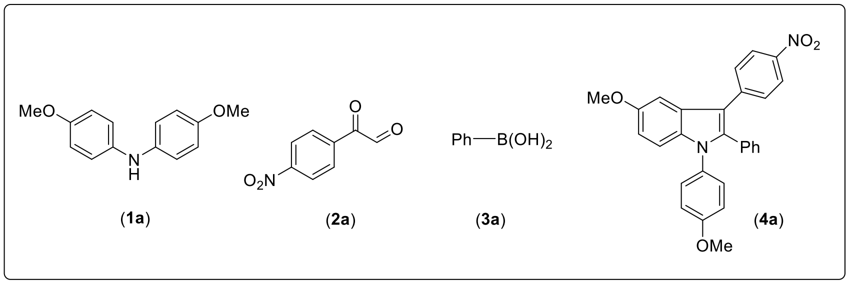

Figure S4. UV-vis absorption spectra of different components

8. Measurement of the H_2O_2 in the reaction mixture

25 μL of the reaction mixture was added into 10 ml of MeCN solution of NaI (0.1 M) and the absorption spectrum of the I^{3-} species resulting from the reaction of I^- with H_2O_2 was measured. The result is shown in the Figure S5.

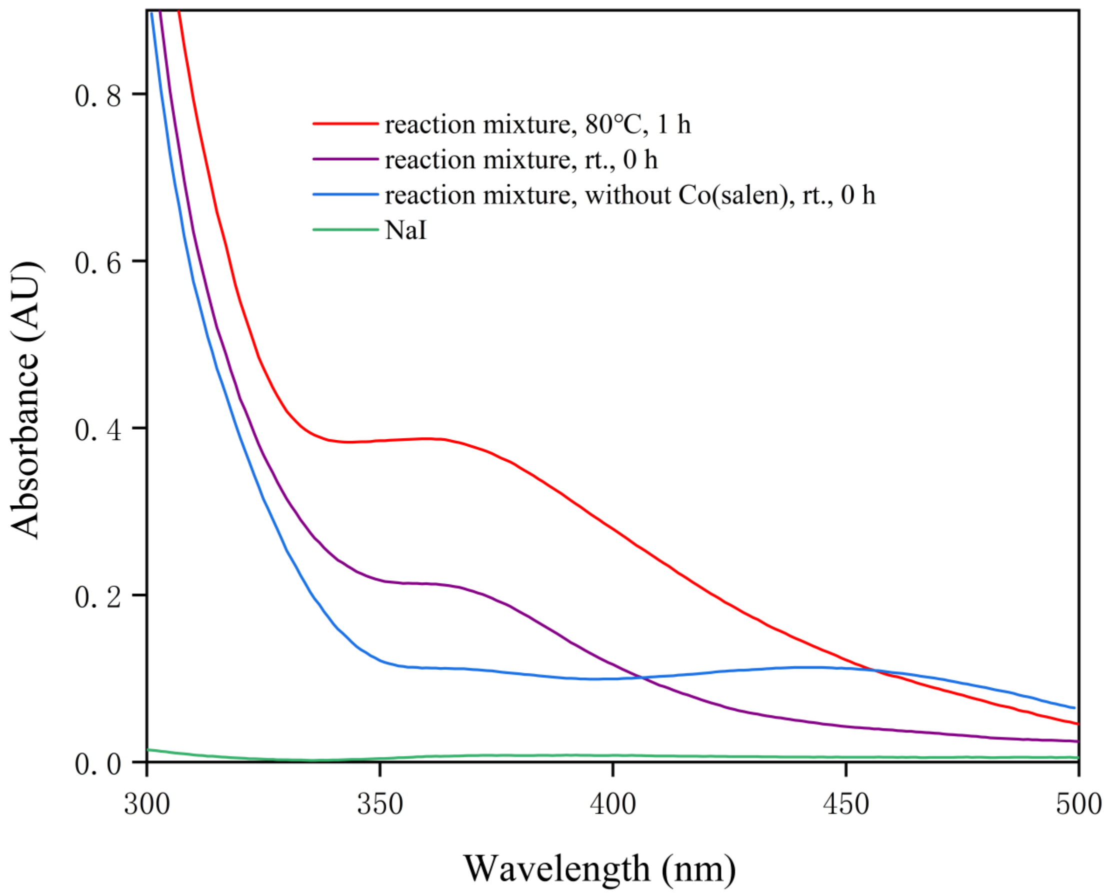

Figure S5. UV-vis absorption spectra of different reaction mixture**:** This experiment further demonstrates that cobalt interacts with oxygen, possibly creating cobalt-oxygen adducts and thus indirectly produces the hydrogen peroxide.

9. Computational Details

All DFT calculations were performed with Gaussian09 package.[1] A careful conformational searching was conducted by CREST[2] to capture the lowest energy conformer of intermediates and key transition states during the catalytic cycle. The ensemble of structures created by CREST was optimized using the B3LYP-D3[3, 4] functional in combination with the def2-SVP[5] basis set. The reaction conditions of liquid phase were simulated in the optimization of all structures by introducing the SMD[6] solvation model (in dichloroethane). Vibrational frequency analysis was used to identify the nature of each optimized structure, as the intermediates show no imaginary frequency while the transition states show only one imaginary frequency. Thermal correction of free energy (G_{therm}) was performed under experimental conditions (353.15 K and 1.0 atm). The single-point energy (E) of each optimized structure was further refined using the B3LYP-D3 functional in combination with a triple-ζ basis set def2-TZVPP[5]. Solvation effects (G_{sol}) were incorporated into single-point energy calculations by using the SMD model (dichloroethane as the solvent).[7, 8] The contribution (G_{std}) originating from the change of the standard state from gas phase to solvent environment (1 mol/L) was also included.[9] As such, the relative Gibbs free energy (ΔG) is given by $\Delta G = \Delta E + \Delta G_{\text{therm}} + \Delta G_{\text{sol}} + \Delta G_{\text{std}}$. Accurate Gibbs free energy calculation of solvated proton is still a challenge in computational chemistry. Herein, the gas-phase Gibbs free energy of proton calculated by Fifen et al. (-6.3 kcal/mol in 298.15 K and 1 atm) and the solvation free energy of -265.9 kcal/mol (298.15 K and 1 atm) provided by Cramer and Truhlar were employed to obtain an approximate solvated Gibbs free energy of proton.[10, 11] The 3D images of DFT-derived structure were created with CYLview.[12]

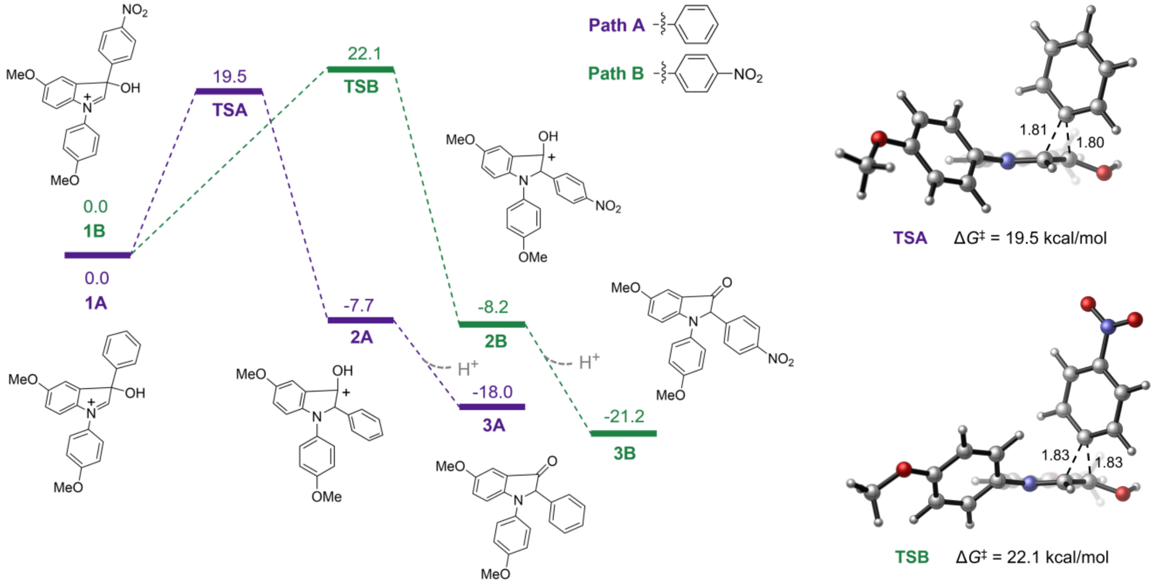

Figure S6. Energy profiles for the 1,2-aryl migration processes. The relative Gibbs free energies are given in kcal/mol at the calculation level of B3LYP-D3/def2-TZVPP//B3LYP-D3/def2-SVP. The selected distances are in Å.

Note: Energy profiles for the proposed 1,2-aryl migration processes were then calculated at the level of B3LYP-D3/def2-TZVPP//B3LYP-D3/def2-SVP (Figure S6). The difference between the relative Gibbs free energies of TSA and TSB is 2.6 kcal/mol, showcasing that the transition states of 1,2-aryl migration with phenyl group (TSA) in-deed favour the one bearing p-nitrophenyl (TSB) migrative unit. Based on the half-life equation for primary reac-tions, the rate of α-iminol rearrangement with a p-nitrophenyl unit should be approximately 40 times slower than that with a phenyl group. This rate gap represents a crucial factor in the successful intercepting intramolecular rearrangements with intermolecular reactions.

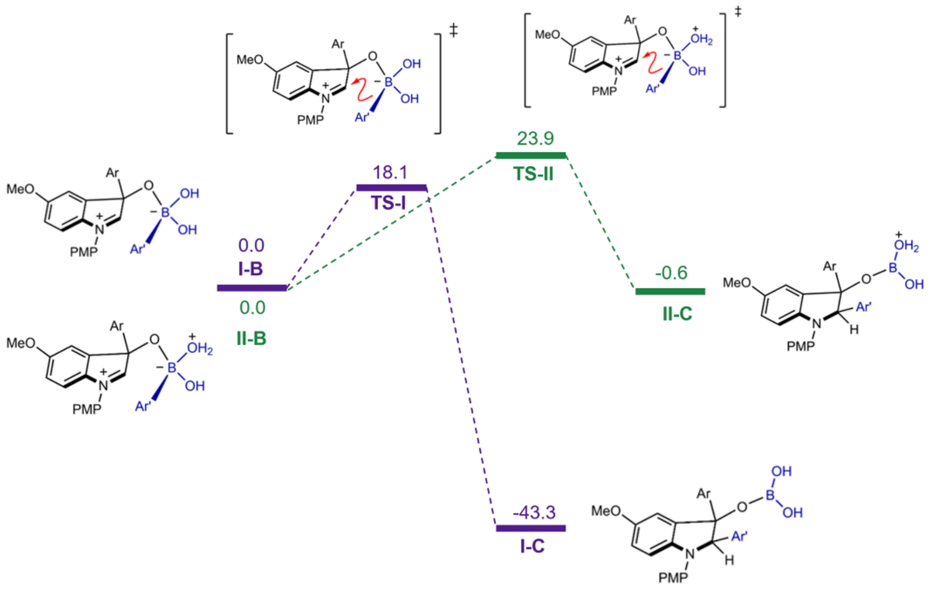

Figure S7. Energy profiles for Petasis reaction. The route involving the deprotonated ate complex is illustrated in purple while the counterpart non-protonation path is shown in green. The relative Gibbs free energies are given in kcal/mol at the calculation level of B3LYP-D3/def2-TZVPP//B3LYP-D3/def2-SVP.

Table S2. Various conformers involved in Petasis reaction.

Structure	Number of conformers [a]
**I-B**	12
**TS-I**	10
**I-C**	26
**II-B**	12
**TS-II**	27
**II-C**	18

[a]CREST was utilized to generate an ensemble of conformers. It is worth noting that the atoms involving in the reaction coordinate of a structure must remain frozen when applying CREST.

List of Cartesian Coordinates

```
1A
Geometry with  46 atoms:
 N  -0.652577  -0.070469  -0.562299
 C  -2.077117  -0.075192  -0.393147
 C  -2.898291  -0.437187  -1.471905
 C  -2.631381   0.254819   0.846939
 C  -4.274397  -0.467744  -1.300893
 H  -2.457910  -0.672323  -2.443085
 C  -4.014623   0.221567   1.020646
 H  -1.986351   0.517585   1.687346
 C  -4.848823  -0.140463  -0.054769
 H  -4.935676  -0.734531  -2.127400
 H  -4.430591   0.472257   1.995752
 C   0.202467   1.036481  -0.215045
 C   1.516869   0.677825  -0.510800
 C  -0.134306   2.296746   0.274878
```

C	2.563914	1.562611	-0.309406
C	0.910898	3.193706	0.480920
H	-1.164570	2.583137	0.485728
C	2.251607	2.844897	0.198644
H	3.587568	1.267296	-0.538639
H	0.713189	4.196501	0.864058
O	-6.188770	-0.198850	0.010280
C	-6.851966	0.117462	1.228877
H	-6.552766	-0.566991	2.041033
H	-6.655730	1.158766	1.537073
H	-7.925850	-0.003514	1.034395
O	3.165028	3.797727	0.442287
C	4.539913	3.548287	0.171686
H	4.707246	3.335322	-0.898048
H	5.077930	4.465622	0.444462
H	4.925148	2.709939	0.776825
C	1.526241	-0.743126	-1.061652
C	0.040236	-1.044334	-1.075340
H	-0.418966	-1.977672	-1.403449
C	2.161354	-1.773851	-0.111010
C	2.285103	-1.535925	1.266873
C	2.586776	-3.001620	-0.644484
C	2.840485	-2.512693	2.098245
H	1.955822	-0.586448	1.693373
C	3.148252	-3.971013	0.189727
H	2.485769	-3.187579	-1.715419
C	3.275116	-3.729643	1.562421
H	2.939273	-2.317828	3.169060
H	3.486387	-4.919818	-0.234488
H	3.713496	-4.489653	2.214201
O	2.002547	-0.846905	-2.381462
H	2.964215	-0.702218	-2.363967

1B

Geometry with 48 atoms:

N	1.251511	0.027911	0.522287
C	2.526861	-0.515107	0.155193
C	2.934103	-0.485174	-1.188184
C	3.337113	-1.092891	1.135793
C	4.154592	-1.041585	-1.539340
H	2.286538	-0.050064	-1.951513
C	4.567431	-1.647400	0.784301
H	3.016381	-1.092389	2.179720
C	4.985752	-1.627383	-0.560286
H	4.490683	-1.043872	-2.577791
H	5.193077	-2.082378	1.563037
C	0.804290	1.351440	0.167170
C	-0.475995	1.536804	0.701444
C	1.472595	2.356406	-0.517041
C	-1.135097	2.738255	0.557352
C	0.809419	3.579649	-0.679233
H	2.478484	2.219010	-0.913580
C	-0.484231	3.778294	-0.152894
H	-2.130916	2.908188	0.970098
H	1.317374	4.379406	-1.216636
O	6.147280	-2.133780	-1.002720
C	7.047201	-2.748716	-0.087410
H	7.906937	-3.085321	-0.681641
H	7.394621	-2.033750	0.678099
H	6.586368	-3.620430	0.407939
O	-1.179240	4.919225	-0.267130
C	-0.618107	6.031509	-0.957201
H	0.308194	6.381006	-0.470281
H	-0.408725	5.786627	-2.012569
H	-1.371252	6.829212	-0.914729
C	-0.892221	0.270932	1.437652
C	0.355300	-0.585899	1.237982
H	0.495021	-1.604414	1.602840
C	-2.059383	-0.476060	0.782285
C	-2.268374	-0.409574	-0.604642
C	-2.901933	-1.264565	1.580498
C	-3.315726	-1.115495	-1.192581
H	-1.619799	0.202826	-1.233301
C	-3.957287	-1.969040	1.003647
H	-2.738934	-1.317996	2.656779
C	-4.147780	-1.883165	-0.376250
H	-3.495098	-1.069288	-2.266021
H	-4.626503	-2.578337	1.610392
O	-1.188771	0.460765	2.798353
H	-0.488188	1.002246	3.200345
N	-5.265372	-2.625325	-0.992445
O	-5.992282	-3.277029	-0.258027

```
 O  -5.403033  -2.548653  -2.204179

2A
Geometry with  46 atoms:
 N  -0.103208  -0.188224  -0.321978
 C   1.168130  -0.835748  -0.254804
 C   1.454325  -1.908480  -1.115902
 C   2.137998  -0.394892   0.651011
 C   2.695886  -2.529657  -1.065424
 H   0.696708  -2.248507  -1.825757
 C   3.392627  -1.007763   0.698938
 H   1.912914   0.431342   1.326681
 C   3.681084  -2.081374  -0.162358
 H   2.935208  -3.364676  -1.727127
 H   4.131885  -0.645038   1.412448
 C  -1.298839  -0.742984  -0.048088
 C  -2.385120   0.132665  -0.401822
 C  -1.581049  -2.011465   0.524484
 C  -3.748329  -0.226919  -0.172375
 C  -2.898481  -2.335082   0.733624
 H  -0.776085  -2.695638   0.793140
 C  -3.994725  -1.459505   0.395153
 H  -4.547610   0.461535  -0.447570
 H  -3.161474  -3.296963   1.180827
 O   4.858491  -2.737065  -0.195949
 C   5.913588  -2.326109   0.662236
 H   5.637136  -2.427630   1.726267
 H   6.762126  -2.989987   0.448705
 H   6.214734  -1.282384   0.464656
 O  -5.207823  -1.965274   0.683731
 C  -6.359652  -1.183278   0.399473
 H  -6.347548  -0.232042   0.959958
 H  -6.442544  -0.967975  -0.680247
 H  -7.226258  -1.778372   0.716223
 C  -1.800702   1.269552  -0.943283
 C  -0.303863   1.127172  -0.959034
 H   0.011470   1.044436  -2.015150
 C   0.450869   2.265245  -0.295626
 C   0.122483   2.656246   1.011933
 C   1.499523   2.901007  -0.969274
 C   0.840705   3.675808   1.638098
 H  -0.690626   2.153960   1.543389
 C   2.217531   3.924433  -0.340373
 H   1.760166   2.591977  -1.984816
 C   1.890344   4.311660   0.961925
 H   0.582357   3.976218   2.656637
 H   3.036478   4.416894  -0.870580
 H   2.452174   5.110261   1.453118
 O  -2.335031   2.373798  -1.393820
 H  -3.309858   2.365297  -1.319727

2B
Geometry with  48 atoms:
 N  -0.774608   0.687386   0.505953
 C   0.184415   1.707213   0.257590
 C   0.339123   2.243733  -1.032682
 C   1.023374   2.142909   1.289624
 C   1.293534   3.224158  -1.269553
 H  -0.279727   1.879802  -1.854578
 C   1.997665   3.115012   1.052275
 H   0.919676   1.724099   2.292716
 C   2.134832   3.671803  -0.231752
 H   1.422951   3.649489  -2.266859
 H   2.636377   3.434899   1.875255
 C  -2.065833   0.637499   0.111399
 C  -2.686972  -0.602960   0.500958
 C  -2.839818   1.617654  -0.563877
 C  -4.048068  -0.899867   0.187045
 C  -4.149196   1.314207  -0.844787
 H  -2.414967   2.581527  -0.841552
 C  -4.768432   0.059947  -0.492773
 H  -4.480544  -1.855772   0.481787
 H  -4.780067   2.044141  -1.357788
 O   3.034564   4.618941  -0.563799
 C   3.939655   5.101965   0.418684
 H   3.409965   5.583989   1.258937
 H   4.582623   4.294341   0.810331
 H   4.569437   5.849644  -0.082225
 O  -6.052876  -0.042828  -0.876787
 C  -6.764384  -1.237042  -0.580234
 H  -7.776486  -1.104853  -0.984374
 H  -6.293349  -2.114013  -1.058104
```

H	-6.827395	-1.407205	0.508897
C	-1.722908	-1.316351	1.198325
C	-0.440565	-0.532176	1.248620
H	-0.252870	-0.275985	2.307009
C	0.750669	-1.288754	0.684962
C	0.882494	-1.461014	-0.701387
C	1.698692	-1.839616	1.555507
C	1.960976	-2.172377	-1.219986
H	0.144091	-1.031972	-1.381689
C	2.781826	-2.558693	1.049564
H	1.596407	-1.702253	2.634071
C	2.896076	-2.710511	-0.332251
H	2.082859	-2.312429	-2.293553
H	3.532720	-2.990134	1.710650
O	-1.768458	-2.481353	1.787608
H	-2.648608	-2.900510	1.709363
N	4.046440	-3.459959	-0.875328
O	4.134390	-3.570592	-2.088857
O	4.848774	-3.929071	-0.082453

3A

Geometry with 45 atoms:

N	0.056033	-0.141091	-0.105171
C	1.340941	-0.747765	-0.113628
C	1.529917	-2.050032	-0.617525
C	2.462304	-0.043752	0.347247
C	2.793685	-2.630078	-0.634455
H	0.677647	-2.612049	-1.004352
C	3.740643	-0.611170	0.310393
H	2.340948	0.964517	0.743999
C	3.916093	-1.917456	-0.173715
H	2.940295	-3.641556	-1.020364
H	4.586457	-0.027314	0.674012
C	-1.132726	-0.822503	0.115917
C	-2.236262	-0.157195	-0.450510
C	-1.350623	-2.013884	0.838639
C	-3.554509	-0.624426	-0.317743
C	-2.652622	-2.479433	0.965899
H	-0.523689	-2.557650	1.296629
C	-3.764222	-1.803422	0.397902
H	-4.365909	-0.056904	-0.773672
H	-2.853688	-3.396478	1.525450
O	5.105139	-2.564151	-0.238088
C	6.275524	-1.907292	0.216626
H	6.209568	-1.646624	1.288260
H	7.105187	-2.613436	0.073256
H	6.483572	-0.989760	-0.362818
O	-4.973867	-2.381712	0.613538
C	-6.127037	-1.757271	0.079418
H	-6.270888	-0.741302	0.490123
H	-6.084632	-1.691870	-1.023042
H	-6.984278	-2.382095	0.366334
C	-1.750326	1.039996	-1.130209
C	-0.214297	1.075259	-0.883269
H	0.282035	1.009943	-1.866416
C	0.162580	2.378803	-0.205405
C	0.132763	2.505991	1.191461
C	0.458842	3.502943	-0.988819
C	0.410172	3.735811	1.794985
H	-0.096828	1.631525	1.804883
C	0.733193	4.734354	-0.384925
H	0.477911	3.411629	-2.077857
C	0.711393	4.854290	1.008658
H	0.390632	3.821306	2.884840
H	0.969967	5.602328	-1.006224
H	0.930856	5.815274	1.481458
O	-2.371266	1.885557	-1.745444

3B

Geometry with 47 atoms:

N	-0.756589	0.619676	0.403226
C	0.079483	1.744256	0.214437
C	0.133475	2.405575	-1.028650
C	0.917723	2.192562	1.244716
C	0.971656	3.499107	-1.215098
H	-0.476068	2.048400	-1.860375
C	1.780364	3.277694	1.056467
H	0.905578	1.690961	2.214092
C	1.806947	3.949349	-0.175991
H	1.011782	4.014634	-2.177299
H	2.418946	3.592297	1.881900
C	-2.089429	0.499133	0.045922

C	-2.620007	-0.746333	0.442162
C	-2.938729	1.426541	-0.593333
C	-3.950584	-1.120819	0.190952
C	-4.255621	1.057257	-0.835753
H	-2.587877	2.415506	-0.886536
C	-4.777844	-0.208672	-0.463970
H	-4.295388	-2.100148	0.522790
H	-4.935380	1.756692	-1.328807
O	2.594725	5.015224	-0.457821
C	3.463964	5.516881	0.542859
H	2.908245	5.866139	1.431656
H	4.205866	4.761390	0.858423
H	3.993609	6.370250	0.097024
O	-6.081116	-0.421480	-0.779406
C	-6.667896	-1.662361	-0.430565
H	-7.710625	-1.624830	-0.775156
H	-6.157907	-2.509966	-0.923499
H	-6.657543	-1.828113	0.662093
C	-1.580753	-1.490929	1.141872
C	-0.336686	-0.552357	1.166029
H	-0.184217	-0.295102	2.229274
C	0.888235	-1.258199	0.636594
C	1.250929	-1.176617	-0.717404
C	1.632479	-2.075067	1.503209
C	2.346069	-1.888466	-1.201194
H	0.674637	-0.547773	-1.397387
C	2.726959	-2.797082	1.033494
H	1.352679	-2.144448	2.556349
C	3.071360	-2.690516	-0.316413
H	2.640405	-1.829969	-2.248609
H	3.313873	-3.432127	1.696405
O	-1.614370	-2.600508	1.637541
N	4.231141	-3.442663	-0.820196
O	4.515923	-3.335246	-2.005155
O	4.852957	-4.139053	-0.029609

TSA

Geometry with 46 atoms:

N	-0.333002	-0.271895	0.747789
C	0.747148	-1.046371	0.299958
C	1.955067	-0.423432	0.665058
C	0.751907	-2.256073	-0.409546
C	3.200334	-0.983238	0.359355
C	1.984491	-2.814556	-0.713894
H	-0.175920	-2.743965	-0.708892
C	3.208126	-2.197055	-0.340150
H	4.120840	-0.484421	0.661583
H	2.042889	-3.760223	-1.256940
C	1.593467	0.817518	1.356508
C	0.149239	0.860101	1.356598
H	-0.491126	1.461985	1.999487
C	0.921968	2.011423	0.188484
C	0.983133	3.337756	0.650766
C	0.820993	1.735770	-1.187678
C	0.944760	4.387121	-0.266452
H	1.066743	3.540361	1.720115
C	0.784148	2.792507	-2.092641
H	0.772922	0.707668	-1.549917
C	0.846913	4.117365	-1.636109
H	0.989697	5.418332	0.091056
H	0.710298	2.582726	-3.162098
H	0.820035	4.941304	-2.353336
O	2.331144	1.429620	2.297096
H	3.263541	1.466984	2.015843
O	4.323864	-2.859354	-0.706788
C	5.593256	-2.306896	-0.391035
H	5.736088	-2.215903	0.700194
H	6.342924	-3.001188	-0.793398
H	5.732545	-1.316071	-0.858671
C	-1.703767	-0.457347	0.387314
C	-2.076350	-0.541942	-0.964573
C	-2.678271	-0.531599	1.385607
C	-3.413415	-0.706387	-1.303692
H	-1.316443	-0.475213	-1.745536
C	-4.025694	-0.683996	1.050071
H	-2.380702	-0.474548	2.435159
C	-4.402472	-0.777217	-0.302022
H	-3.723400	-0.776360	-2.348302
H	-4.768026	-0.741235	1.845812
O	-5.670335	-0.935900	-0.731288
C	-6.724710	-1.025005	0.216624
H	-6.804573	-0.106531	0.824235

H	-7.649872	-1.153207	-0.361338
H	-6.596449	-1.892838	0.886944

TSB

Geometry with 48 atoms:

N	0.849003	0.762427	0.823723
C	0.157416	1.800658	0.178283
C	-1.151812	1.881457	0.694809
C	0.562228	2.642939	-0.868675
C	-2.081937	2.815996	0.226608
C	-0.354509	3.573240	-1.331327
H	1.555611	2.569384	-1.310379
C	-1.666826	3.681335	-0.792843
H	-3.086383	2.853128	0.647237
H	-0.085092	4.257624	-2.138449
C	-1.272411	0.818191	1.688577
C	0.003961	0.140293	1.699664
H	0.390274	-0.512688	2.481102
C	-1.316059	-0.805306	0.849319
C	-1.883597	-1.818026	1.638532
C	-1.263929	-0.921551	-0.550050
C	-2.403475	-2.956689	1.029374
H	-1.923575	-1.713345	2.723908
C	-1.780371	-2.057518	-1.161073
H	-0.826664	-0.135471	-1.166754
C	-2.343044	-3.058004	-0.361391
H	-2.847036	-3.757676	1.619825
H	-1.754731	-2.172216	-2.244168
O	-2.092601	0.809981	2.745521
H	-2.970722	1.151644	2.494341
O	-2.433689	4.642115	-1.339408
C	-3.765635	4.820138	-0.875730
H	-4.376599	3.916857	-1.047153
H	-3.788423	5.077373	0.197609
H	-4.185671	5.653444	-1.454286
C	2.140121	0.258003	0.478853
C	2.319611	-1.117433	0.259304
C	3.230579	1.128207	0.382552
C	3.581256	-1.609241	-0.052445
H	1.470938	-1.800653	0.323884
C	4.496919	0.638607	0.056025
H	3.099999	2.193902	0.579432
C	4.682964	-0.738554	-0.162736
H	3.738813	-2.675611	-0.226374
H	5.331074	1.336374	-0.010566
O	5.863027	-1.309508	-0.474924
C	7.022685	-0.498368	-0.601273
H	7.848657	-1.177325	-0.852526
H	6.911651	0.246749	-1.408536
H	7.259915	0.022330	0.342940
N	-2.891967	-4.264636	-1.012957
O	-3.402375	-5.114050	-0.299489
O	-2.802624	-4.344614	-2.228273

I-B

Geometry with 63 atoms:

C	-0.494128	-0.773234	-1.290626
C	-1.054713	-1.151988	-2.508595
C	-0.431828	-2.192008	-3.195974
C	0.719711	-2.831122	-2.681514
C	1.281637	-2.410083	-1.456948
C	0.656335	-1.373073	-0.782702
H	-1.942173	-0.665389	-2.914086
H	-0.827295	-2.541026	-4.151749
H	2.174137	-2.872070	-1.036897
N	-0.929416	0.226412	-0.353530
C	1.048061	-0.687842	0.524578
C	-0.155907	0.240219	0.697883
H	-0.316213	0.914573	1.536796
O	1.325666	-1.533101	1.565656
B	0.389574	-1.564717	2.793339
O	0.895443	-2.614368	3.650276
H	0.956446	-3.440848	3.154783
O	0.432150	-0.289482	3.500229
H	1.134291	-0.336331	4.162093
O	1.215732	-3.832945	-3.432649
C	2.367482	-4.537335	-2.987899
H	3.242340	-3.870023	-2.899720
H	2.189834	-5.032653	-2.017594
H	2.574423	-5.301879	-3.748710
C	-2.060650	1.086774	-0.538983
C	-3.325011	0.536075	-0.795701

C -1.895698 2.468402 -0.428761
C -4.416992 1.379178 -0.945509
H -3.449974 -0.546136 -0.844758
C -2.993017 3.319039 -0.577401
H -0.903186 2.882284 -0.237267
C -4.264692 2.777505 -0.839770
H -5.413638 0.975757 -1.134499
H -2.843213 4.394997 -0.494231
O -5.384649 3.506421 -1.003308
C -5.322321 4.922772 -0.904446
H -6.344423 5.288948 -1.070405
H -4.981898 5.245432 0.094861
H -4.656366 5.353972 -1.672042
C -1.142506 -1.825836 2.238713
C -1.409229 -2.834978 1.288205
C -2.256230 -1.122108 2.742400
C -2.709534 -3.132842 0.865872
H -0.571481 -3.398191 0.865502
C -3.562815 -1.404692 2.326751
H -2.079205 -0.331384 3.477377
C -3.794800 -2.414721 1.384985
H -2.879705 -3.921862 0.126724
H -4.404336 -0.835173 2.733332
H -4.813359 -2.637891 1.054209
C 2.214981 0.321989 0.311006
C 2.646379 0.748158 -0.954721
C 2.838682 0.828263 1.464075
C 3.698639 1.656628 -1.072670
H 2.173456 0.366260 -1.860115
C 3.886620 1.737606 1.360855
H 2.473478 0.501259 2.437313
C 4.305782 2.137408 0.088179
H 4.051260 1.987431 -2.049017
H 4.377410 2.138364 2.247355
N 5.417122 3.096506 -0.031925
O 5.764070 3.437133 -1.154289
O 5.935608 3.505819 0.997152

TS-I
Geometry with 63 atoms:
C 0.438225 1.046595 -1.125173
C 0.991510 1.580185 -2.281198
C 0.385242 2.717485 -2.837865
C -0.750483 3.295897 -2.240000
C -1.308352 2.722820 -1.073590
C -0.707142 1.603426 -0.535727
H 1.871269 1.140712 -2.752588
H 0.814984 3.147221 -3.742104
H -2.196176 3.171672 -0.624285
N 0.849666 -0.075855 -0.347998
C -1.097565 0.768908 0.685220
C 0.157913 -0.122973 0.799191
H 0.174713 -1.026828 1.405079
O -1.337476 1.495953 1.824433
B -0.471805 1.112843 2.975311
O -0.520958 2.094657 4.012553
H -0.250742 2.957462 3.672680
O -0.820354 -0.196486 3.469312
H -0.539606 -0.275440 4.389555
O -1.380699 4.394718 -2.710716
C -0.889906 5.039633 -3.875350
H 0.140826 5.410043 -3.733245
H -0.914803 4.370963 -4.754005
H -1.554333 5.895030 -4.059188
C 1.934999 -0.948786 -0.657806
C 3.223783 -0.431786 -0.862433
C 1.717436 -2.327349 -0.718501
C 4.277944 -1.293882 -1.134515
H 3.396468 0.642035 -0.779908
C 2.775856 -3.200530 -0.982921
H 0.710618 -2.721354 -0.562311
C 4.066779 -2.686165 -1.198202
H 5.289245 -0.912476 -1.289296
H 2.580914 -4.271803 -1.024551
O 5.154457 -3.437788 -1.466700
C 5.027796 -4.849736 -1.547523
H 4.684515 -5.283833 -0.592052
H 4.334032 -5.151309 -2.351840
H 6.029661 -5.237730 -1.776369
C 1.145687 1.072746 2.283854
C 1.624036 2.266297 1.679620
C 2.120899 0.185197 2.799502

C	2.986455	2.549592	1.593258
H	0.899858	2.983942	1.284356
C	3.487553	0.458795	2.719124
H	1.779139	-0.742083	3.270375
C	3.924004	1.643082	2.111799
H	3.327383	3.478815	1.127483
H	4.216995	-0.248563	3.123790
H	4.993359	1.860686	2.040607
C	-2.265317	-0.194479	0.355517
C	-2.407036	-0.782364	-0.913166
C	-3.184521	-0.509085	1.367625
C	-3.451682	-1.666203	-1.174599
H	-1.702596	-0.550143	-1.713428
C	-4.235771	-1.389679	1.119738
H	-3.052343	-0.065336	2.352914
C	-4.356995	-1.956484	-0.151091
H	-3.572342	-2.123820	-2.155920
H	-4.956691	-1.639956	1.897687
N	-5.465699	-2.885653	-0.420314
O	-6.246822	-3.128797	0.489001
O	-5.550794	-3.370822	-1.540451

I-C

Geometry with 63 atoms:

C	0.299316	-0.687432	1.512598
C	0.587440	-0.976446	2.856425
C	-0.454142	-1.374140	3.696101
C	-1.777871	-1.498201	3.230463
C	-2.062870	-1.217069	1.882343
C	-1.014800	-0.826323	1.051682
H	1.603941	-0.911506	3.244672
H	-0.255512	-1.611235	4.743830
H	-3.068665	-1.306722	1.472119
N	1.153464	-0.308670	0.470015
C	-1.078061	-0.470466	-0.429550
C	0.476384	-0.481988	-0.820263
H	0.689432	0.383195	-1.463777
O	-1.842872	-1.342175	-1.216809
B	-1.976758	-2.713584	-1.161591
O	-1.498620	-3.536892	-0.184204
H	-1.000734	-3.084385	0.511041
O	-2.700138	-3.246528	-2.190728
H	-2.781008	-4.207913	-2.108884
O	-2.700549	-1.898627	4.142724
C	-4.042804	-2.073167	3.726651
H	-4.603548	-2.409352	4.609918
H	-4.486702	-1.129665	3.360609
H	-4.130560	-2.839367	2.934939
C	2.390570	0.347110	0.580234
C	2.714006	1.137186	1.705838
C	3.330386	0.279891	-0.463688
C	3.934123	1.799826	1.787203
H	1.998391	1.250961	2.520486
C	4.551315	0.960423	-0.391516
H	3.118081	-0.317580	-1.350719
C	4.871956	1.720707	0.742480
H	4.176935	2.408696	2.661353
H	5.244982	0.878205	-1.228544
O	6.032120	2.405556	0.917091
C	7.014379	2.362770	-0.101377
H	6.640101	2.782252	-1.053046
H	7.856240	2.976433	0.248954
H	7.375111	1.333976	-0.283210
C	0.883926	-1.744486	-1.562982
C	1.525372	-2.806833	-0.913055
C	0.584258	-1.862987	-2.929127
C	1.854568	-3.971854	-1.612581
H	1.772637	-2.717550	0.146727
C	0.904901	-3.029214	-3.628180
H	0.083590	-1.038310	-3.442907
C	1.540699	-4.088858	-2.970338
H	2.356330	-4.792499	-1.093022
H	0.660090	-3.110593	-4.690525
H	1.795101	-5.000879	-3.516914
C	-1.651452	0.940612	-0.610851
C	-1.069146	2.029067	0.065650
C	-2.751905	1.169589	-1.449141
C	-1.570120	3.317293	-0.090741
H	-0.212968	1.875868	0.723653
C	-3.269611	2.455070	-1.611559
H	-3.206109	0.332885	-1.978139
C	-2.670555	3.514563	-0.930131

H	-1.121507	4.163245	0.428935
H	-4.126938	2.639096	-2.258547
N	-3.209576	4.873046	-1.097855
O	-4.173556	5.022783	-1.836009
O	-2.666597	5.786156	-0.491026

II-B

Geometry with 64 atoms:

C	-0.519340	-0.424386	-1.158391
C	-1.125776	-0.767091	-2.364201
C	-0.535060	-1.793739	-3.097885
C	0.634276	-2.448015	-2.645822
C	1.265197	-2.035969	-1.450483
C	0.655546	-1.034691	-0.719091
H	-2.025431	-0.267553	-2.723197
H	-0.974714	-2.124205	-4.040646
H	2.183295	-2.497566	-1.096503
N	-0.921908	0.539207	-0.170275
C	1.083186	-0.387543	0.588724
C	-0.102362	0.536415	0.840952
H	-0.244979	1.175070	1.713164
O	1.347913	-1.276228	1.639966
B	0.411054	-2.058700	2.295129
O	1.327593	-4.256234	0.685892
H	2.104967	-3.745015	0.960589
O	0.939302	-2.747673	3.340227
H	0.278533	-3.284658	3.803060
O	1.082468	-3.453062	-3.412708
C	2.194677	-4.227990	-2.968894
H	2.007189	-4.655747	-1.969349
H	2.315056	-5.038058	-3.700010
H	3.118494	-3.624917	-2.942851
C	-2.058613	1.404753	-0.286365
C	-3.322114	0.868375	-0.547196
C	-1.886915	2.784621	-0.100832
C	-4.430136	1.711151	-0.623365
H	-3.447601	-0.209328	-0.652654
C	-2.987881	3.624550	-0.179953
H	-0.889966	3.190425	0.083847
C	-4.271024	3.099173	-0.440417
H	-5.410619	1.275353	-0.811894
H	-2.881605	4.703252	-0.050564
O	-5.271962	3.992484	-0.495165
C	-6.600156	3.552496	-0.754113
H	-6.678135	3.061826	-1.739458
H	-6.955355	2.859898	0.028203
H	-7.228505	4.452959	-0.749869
C	-1.125626	-2.074075	1.945536
C	-1.635197	-2.787498	0.841922
C	-2.038721	-1.393000	2.778724
C	-3.008629	-2.817453	0.580972
H	-0.944313	-3.333203	0.196646
C	-3.411574	-1.413537	2.512663
H	-1.672370	-0.837692	3.647774
C	-3.898798	-2.129210	1.413562
H	-3.385146	-3.379365	-0.277992
H	-4.102406	-0.873616	3.165626
H	-4.971765	-2.148446	1.204656
C	2.297249	0.550777	0.428778
C	2.599485	1.153744	-0.802742
C	3.078061	0.841570	1.558738
C	3.673660	2.035264	-0.909716
H	2.004304	0.934310	-1.690185
C	4.158067	1.716077	1.461602
H	2.847398	0.372297	2.515116
C	4.438588	2.302075	0.226317
H	3.923105	2.506832	-1.859679
H	4.777470	1.945621	2.328100
N	5.579614	3.232265	0.118789
O	6.231289	3.455455	1.127822
O	5.809558	3.730450	-0.972807
H	1.144190	-4.807858	1.462850

TS-II

Geometry with 64 atoms:

C	0.368538	1.080195	-1.053514
C	0.875803	1.759831	-2.157241
C	0.232981	2.936833	-2.570845
C	-0.898870	3.422555	-1.889156
C	-1.419293	2.705639	-0.787060
C	-0.782497	1.547094	-0.393362
H	1.748551	1.396490	-2.700259

H	0.630575	3.470412	-3.433763
H	-2.311096	3.075273	-0.277499
N	0.808709	-0.115308	-0.434195
C	-1.146168	0.564590	0.714643
C	0.155120	-0.285926	0.764988
H	0.080707	-1.311237	1.131719
O	-1.359687	1.155729	1.963659
B	-0.432204	0.628591	2.923862
O	-0.356765	1.878644	4.006172
H	-0.444170	1.576977	4.932312
O	-0.754896	-0.529159	3.636318
H	-1.712833	-0.644131	3.727623
O	-1.561321	4.553018	-2.215382
C	-1.114926	5.336962	-3.311474
H	-1.156400	4.772943	-4.259950
H	-1.797641	6.194964	-3.376620
H	-0.086116	5.706757	-3.155358
C	1.953963	-0.881875	-0.783886
C	3.189598	-0.269330	-1.020487
C	1.855743	-2.283061	-0.832147
C	4.318560	-1.037379	-1.312958
H	3.284157	0.814119	-0.942978
C	2.976100	-3.053486	-1.114276
H	0.891607	-2.763632	-0.651289
C	4.219803	-2.439925	-1.363002
H	5.270044	-0.535052	-1.485950
H	2.913153	-4.142707	-1.156824
O	5.247315	-3.268878	-1.636416
C	6.534566	-2.727130	-1.894334
H	7.193862	-3.581171	-2.100147
H	6.527020	-2.058990	-2.773372
H	6.925027	-2.174171	-1.022118
C	1.062670	0.520527	2.192860
C	1.612413	1.796316	1.809930
C	1.996106	-0.458582	2.669869
C	2.974573	2.039747	1.855179
H	0.933857	2.591433	1.494706
C	3.359711	-0.206831	2.725511
H	1.605396	-1.427622	2.989929
C	3.851240	1.040089	2.313677
H	3.369334	3.013764	1.556576
H	4.047649	-0.974424	3.086862
H	4.925324	1.239558	2.352614
C	-2.329946	-0.325204	0.305215
C	-3.444417	-0.439439	1.147352
C	-2.307097	-1.033794	-0.909132
C	-4.520708	-1.254341	0.794619
H	-3.472120	0.120000	2.082108
C	-3.370862	-1.854674	-1.271358
H	-1.453377	-0.948990	-1.583232
C	-4.466923	-1.954474	-0.410183
H	-5.393305	-1.347995	1.440731
H	-3.360234	-2.411884	-2.207419
N	-5.596439	-2.822216	-0.786809
O	-6.546310	-2.892336	-0.020368
O	-5.524590	-3.428446	-1.846071
H	-1.048496	2.550888	3.839437

II-C

Geometry with 64 atoms:

C	0.401644	-0.670807	1.503262
C	0.735835	-0.997474	2.831530
C	-0.254004	-1.490145	3.676537
C	-1.586101	-1.669821	3.244379
C	-1.923301	-1.353202	1.912212
C	-0.914195	-0.858880	1.071911
H	1.756921	-0.888913	3.197362
H	-0.013141	-1.754751	4.708434
H	-2.946682	-1.427707	1.540110
N	1.209193	-0.215024	0.465362
C	-1.030078	-0.476355	-0.395372
C	0.508969	-0.338572	-0.819322
H	0.638107	0.586878	-1.396269
O	-1.696215	-1.460056	-1.184093
B	-1.649593	-2.794990	-1.183722
O	-1.276447	-3.455116	0.075843
H	-1.293642	-2.884698	0.913480
O	-2.000888	-3.533997	-2.232367
H	-2.010810	-4.500613	-2.146726
O	-2.454651	-2.146732	4.160442
C	-3.805912	-2.364209	3.784780
H	-4.317013	-2.759112	4.672965

H	-4.298491	-1.426366	3.473531
H	-3.885120	-3.102592	2.966418
C	2.418917	0.499500	0.588430
C	2.687640	1.297447	1.720761
C	3.369577	0.476651	-0.444531
C	3.875048	2.013430	1.821111
H	1.956095	1.373896	2.526187
C	4.556504	1.213085	-0.355926
H	3.192629	-0.125546	-1.336233
C	4.827105	1.982033	0.785948
H	4.080218	2.628769	2.700088
H	5.263336	1.168047	-1.184623
O	5.948866	2.719001	0.977434
C	6.944279	2.735799	-0.030665
H	6.557620	3.141109	-0.983179
H	7.747218	3.391462	0.334020
H	7.361223	1.728902	-0.213210
C	0.974235	-1.510041	-1.669406
C	1.546739	-2.655171	-1.096209
C	0.783125	-1.464191	-3.059400
C	1.906934	-3.743080	-1.899035
H	1.718540	-2.691050	-0.018370
C	1.143956	-2.549005	-3.860995
H	0.340639	-0.572541	-3.511826
C	1.703947	-3.693917	-3.281819
H	2.356002	-4.628465	-1.441391
H	0.989196	-2.500972	-4.941889
H	1.988945	-4.542485	-3.909145
C	-1.772226	0.843827	-0.579484
C	-1.351879	1.965540	0.157337
C	-2.845331	0.967915	-1.472097
C	-1.991791	3.191914	0.010173
H	-0.515484	1.886473	0.853806
C	-3.499234	2.191277	-1.625828
H	-3.175783	0.107342	-2.052590
C	-3.063932	3.286661	-0.881015
H	-1.673158	4.066384	0.576453
H	-4.337131	2.298724	-2.314127
N	-3.753990	4.579323	-1.035938
O	-4.689926	4.639290	-1.820066
O	-3.354969	5.524652	-0.371413
H	-1.303659	-4.424913	0.229531

References

(1) Gaussian 09, Revision E.01, M. J. Frisch, G. W. Trucks, H. B. Schlegel, G. E. Scuseria, M. A. Robb, J. R. Cheeseman, G. Scalmani, V. Barone, G. A. Petersson, H. Nakatsuji, X. Li, M. Caricato, A. Marenich, J. Bloino, B. G. Janesko, R. Gomperts, B. Mennucci, H. P. Hratchian, J. V. Ortiz, A. F. Izmaylov, J. L. Sonnenberg, D. Williams-Young, F. Ding, F. Lipparini, F. Egidi, J. Goings, B. Peng, A. Petrone, T. Henderson, D. Ranasinghe, V. G. Zakrzewski, J. Gao, N. Rega, G. Zheng, W. Liang, M. Hada, M. Ehara, K. Toyota, R. Fukuda, J. Hasegawa, M. Ishida, T. Nakajima, Y. Honda, O. Kitao, H. Nakai, T. Vreven, K. Throssell, J. A. Montgomery, Jr., J. E. Peralta, F. Ogliaro, M. Bearpark, J. J. Heyd, E. Brothers, K. N. Kudin, V. N. Staroverov, T. Keith, R. Kobayashi, J. Normand, K. Raghavachari, A. Rendell, J. C. Burant, S. S. Iyengar, J. Tomasi, M. Cossi, J. M. Millam, M. Klene, C. Adamo, R. Cammi, J. W. Ochterski, R. L. Martin, K. Morokuma, O. Farkas, J. B. Foresman, and D. J. Fox, Gaussian, Inc., Wallingford CT, 2016.

(2) Pracht, P.; Bohle, F.; Grimme, S. Automated exploration of the low-energy chemical space with fast quantum chemical methods. *Phys. Chem. Chem. Phys.* **2020**, *22*, 7169-7192.

(3) Stephens, P. J.; Devlin, F. J.; Chabalowski, C. F.; Frisch, M. J. Ab Initio Calculation of Vibrational Absorption and Circular Dichroism Spectra Using Density Functional Force Fields. *J. Phys. Chem.* **1994**, *98*, 11623–11627.

(4) Grimme, S.; Antony, J.; Ehrlich, S.; Krieg, H. A consistent and accurate ab initio parametrization of density functional dispersion correction (DFT-D) for the 94 elements H-Pu. *J. Chem. Phys.* **2010**, *132*, 154104.

(5) Weigend, F.; Ahlrichs, R. Balanced basis sets of split valence, triple zeta valence and quadruple zeta valence quality for H to Rn: Design and assessment of accuracy. *Phys. Chem. Chem. Phys.* **2005**, *7*, 3297-3305.

(6) Marenich, A. V.; Cramer, C. J.; Truhlar, D. G. Universal Solvation Model Based on Solute Electron Density and on a Continuum Model of the Solvent Defined by the Bulk Dielectric Constant and Atomic Surface Tensions. *J. Phys. Chem. B* **2009**, *113*, 6378-6396.

(7) Shao, H.; Pandharkar, R.; Cramer, C. J. Factors Affecting the Mechanism of 1,3-Butadiene Polymerization at Open Metal Sites in Co-MFU-4l. *Organometallics* **2022**, *41*, 169-177.

(8) Greaves, M. E.; Ronson, T. O.; Maseras, F.; Nelson, D. J. The Effect of Added Ligands on the Reactions of [Ni(COD)(dppf)] with Alkyl Halides: Halide Abstraction May Be Reversible. *Organometallics* **2021**, *40*, 1997-2007.

(9) Kozuch, S.; Martin, J. M. What Makes for a Bad Catalytic Cycle? A Theoretical Study on the Suzuki−Miyaura Reaction within the Energetic Span Model. *ACS Catal.* **2011**, *1*, 246-253.

(10) Kelly, C. P.; Cramer, C. J.; Truhlar, D. G. Aqueous Solvation Free Energies of Ions and Ion-Water Clusters Based on an Accurate Value for the Absolute Aqueous Solvation Free Energy of the Proton. *J. Phys. Chem. B* **2006**, *110*, 16066-16081.

(11) Fifen, J. J.; Dhaouadi, Z.; Nsangou, M. Revision of the thermodynamics of the proton in gas phase. *J. Phys. Chem. A* **2014**, *118*, 11090-11097.

(12) CYLview, 1.0b; Legault, C. Y., Université de Sherbrooke, 2009 (http://www.cylview.org).

10. Discussion of the possible mechanistic details

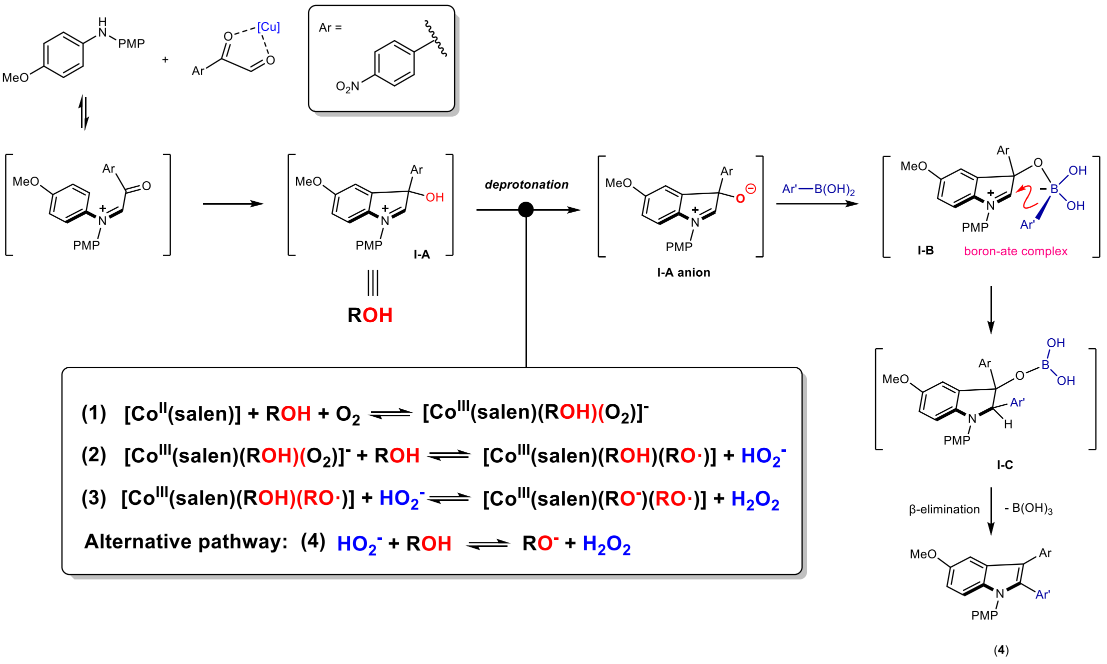

Figure S8. Supplementary discussion for possible mechanism

Supplementary Discussion: Based on previous reports (J. Chem. Soc., Dalton Trans.,24, 4695−4700 (1997).) and our own DFT calculations, we believe that the intermediate **I-A**, if preferentially deprotonated, will be favorable for the subsequent reaction with boric acid to generate the boron-ate complex **I-B**. The whole reaction will be more likely to take place through such a mechanistic process. Therefore, we believe that cobalt may be involved in the synergistic deprotonation process in the reaction system. Combined with the results of our EPR experiments that detected the possible generation of free radicals in the reaction system and the iodometry experiments that revealed the presence of hydrogen peroxide in the system, we propose a mechanistic process as shown in Fig. S8. The interaction of cobalt with the intermediate **I-A** in the presence of oxygen led to the generation of the HO_2^- ion, which may have acted as a base to carry out deprotonation process of **I-A**. Meanwhile, metallic species of copper and cobalt may coordinate to the *N* atom of the indoline intermediate (**I-C**), thus enhancing the acidity of the C-H bond adjacent to nitrogen and therefore facilitating the final elimination step. Given that copper and cobalt act primarily as a reaction accelerator rather than an essential additive, we believe that neither of these metals is

involved in the formation of carbon-metal bonds. At this stage we believe that the mechanism still has a lot of unknowns, we give a possible way of mechanism here. For the mechanism of this reaction, especially the role of copper/cobalt reagent, we will further study in the future.

11. Copies of the ^{1}H, ^{13}C and ^{19}F spectra

^{1}H NMR of compound **4a** (in $CDCl_3$)

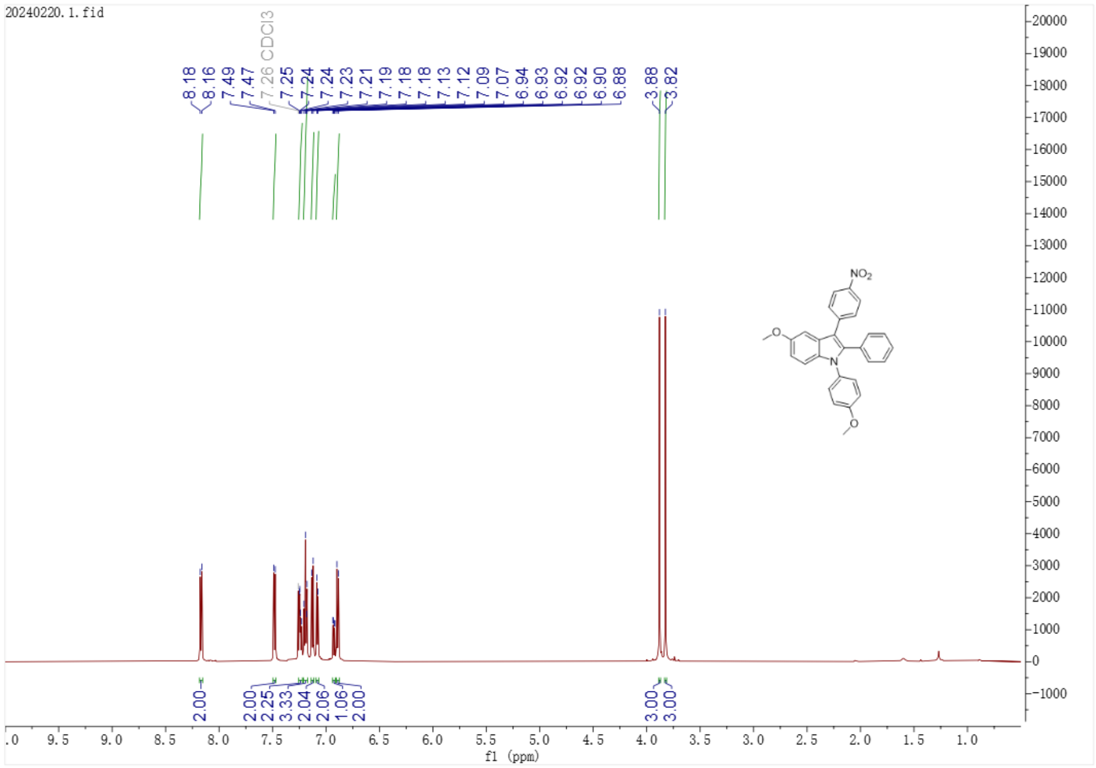

^{13}C NMR of compound **4a** (in $CDCl_3$)

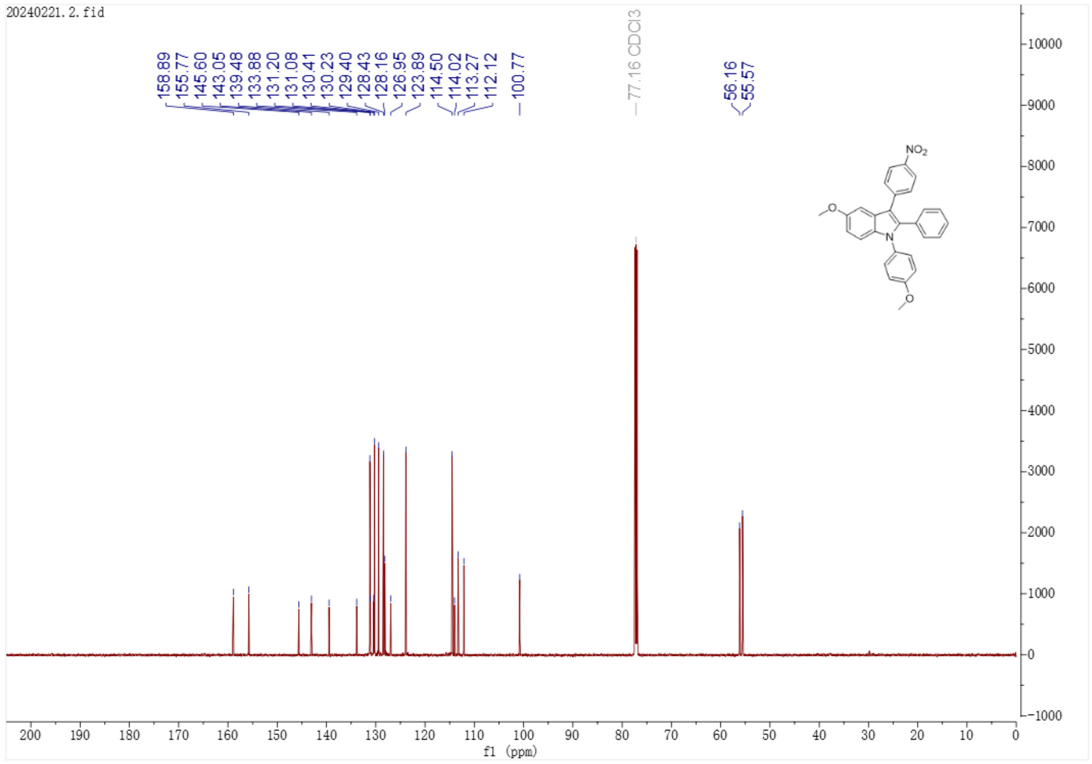

^{1}H NMR of compound **4b** (in $CDCl_3$)

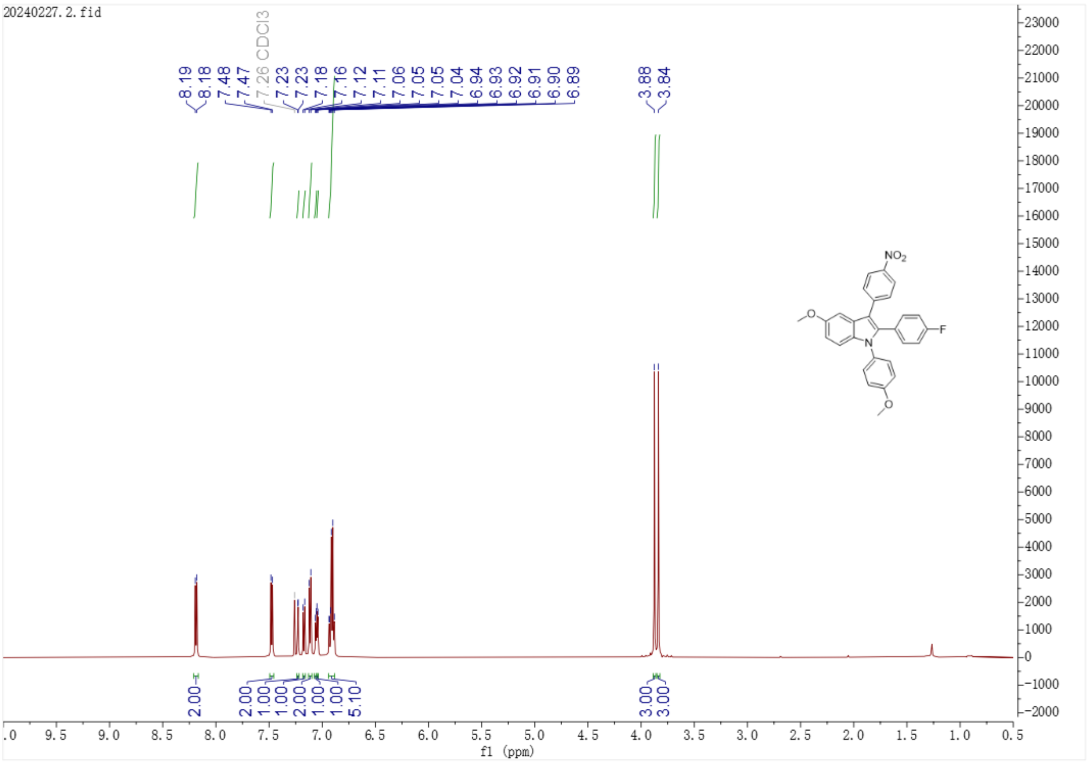

^{13}C NMR of compound **4b** (in $CDCl_3$)

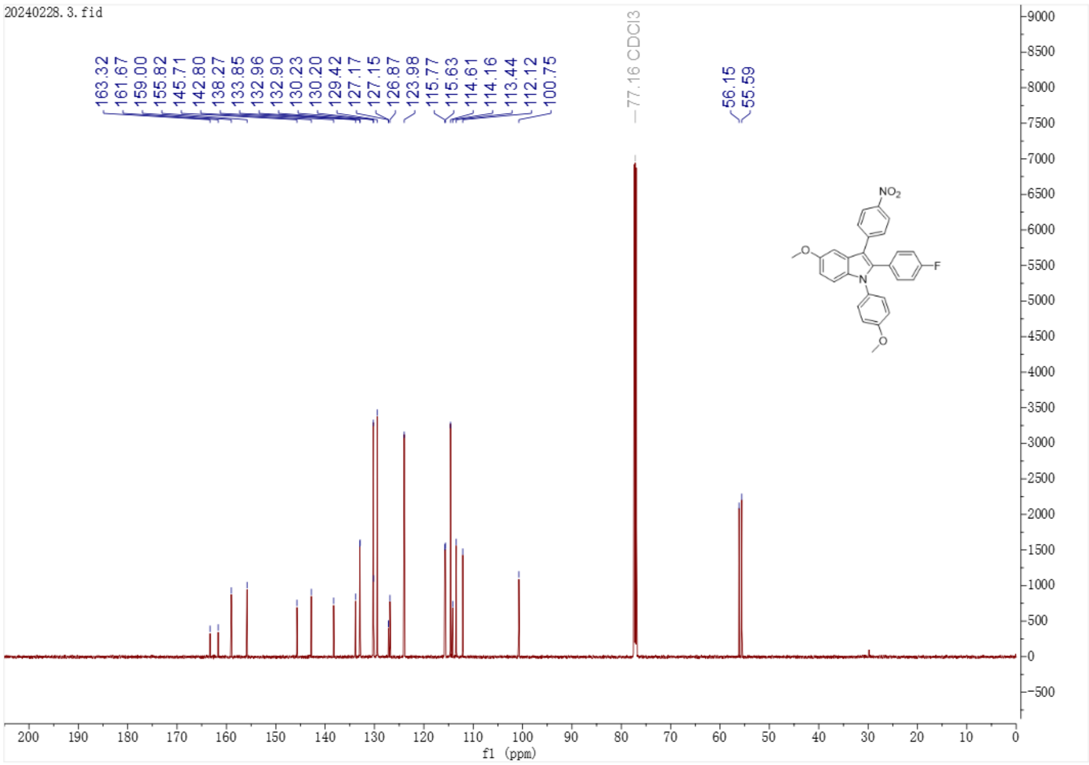

^{19}F NMR of compound **4b** (in $CDCl_3$)

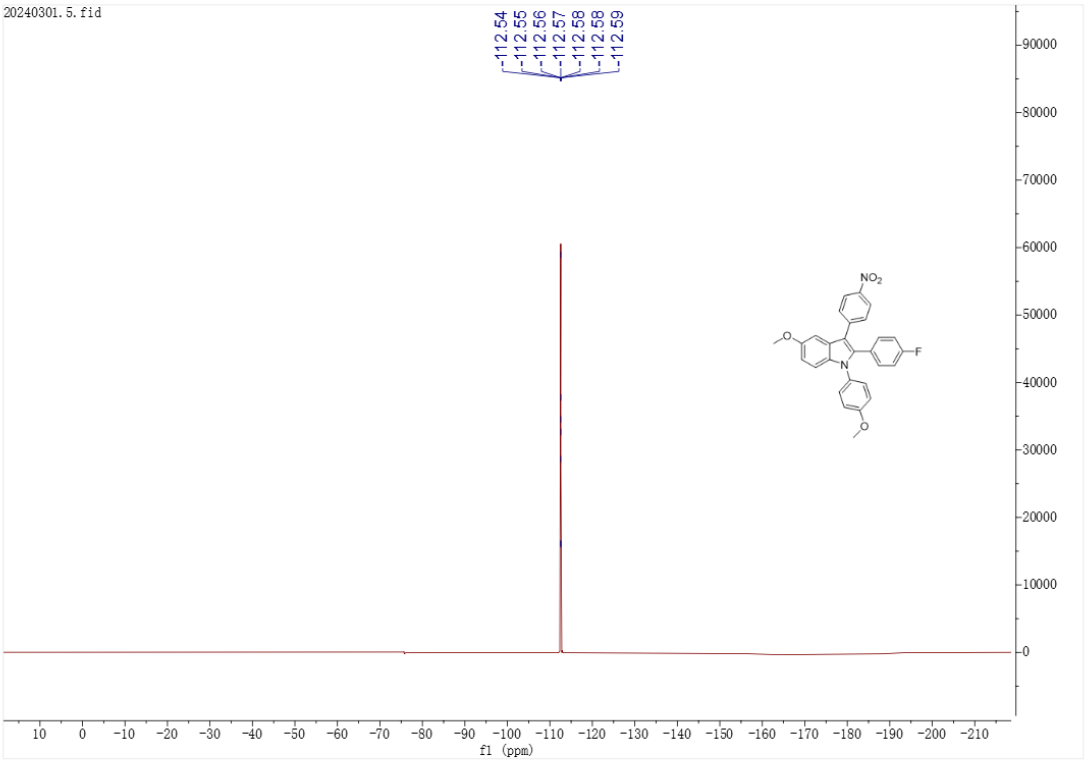

^{1}H NMR of compound **4c** (in $CDCl_3$)

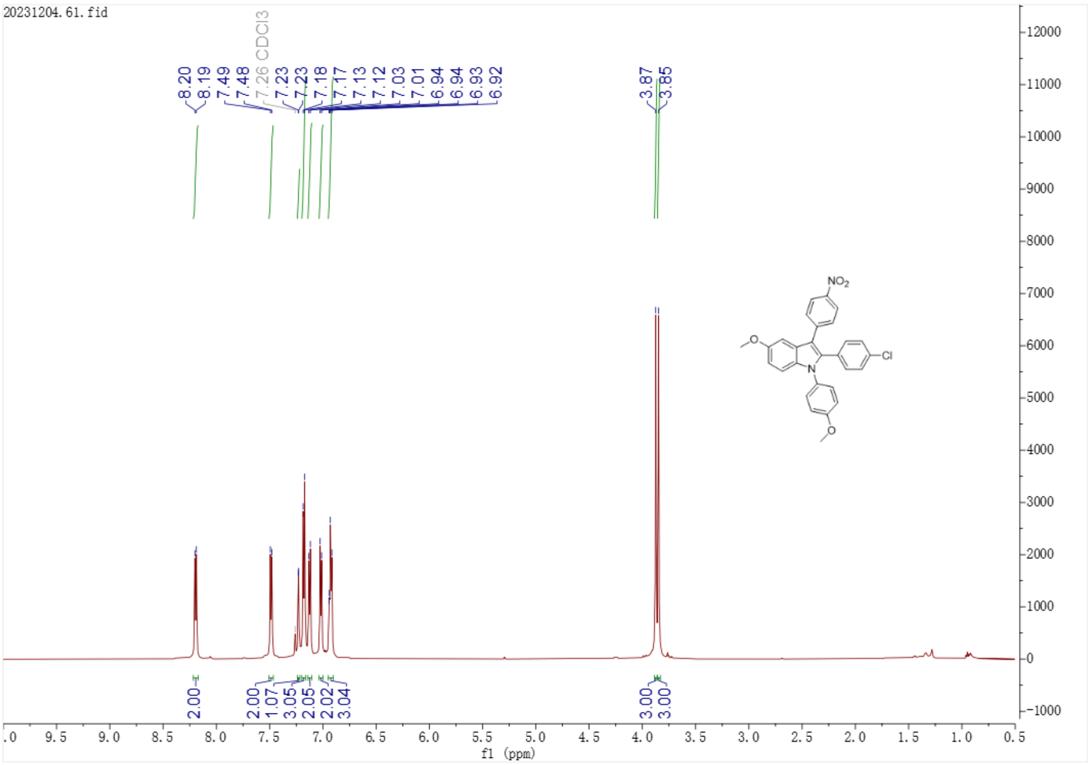

^{13}C NMR of compound **4c** (in $CDCl_3$)

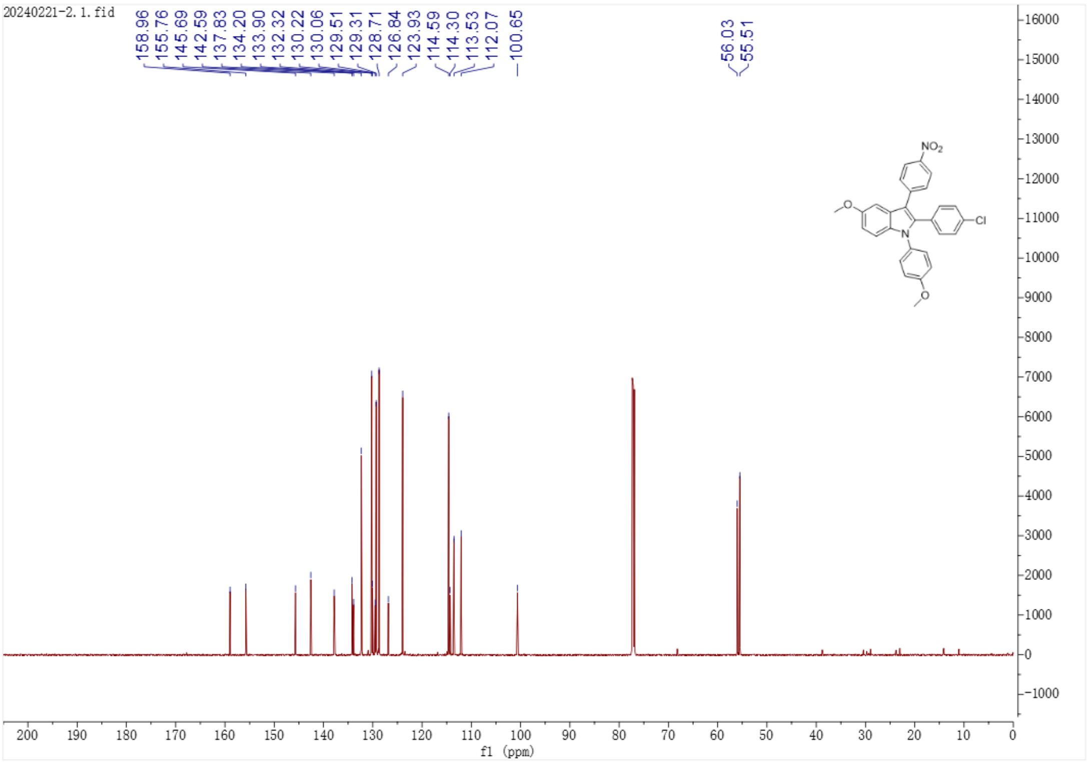

^{1}H NMR of compound **4d** (in $CDCl_3$)

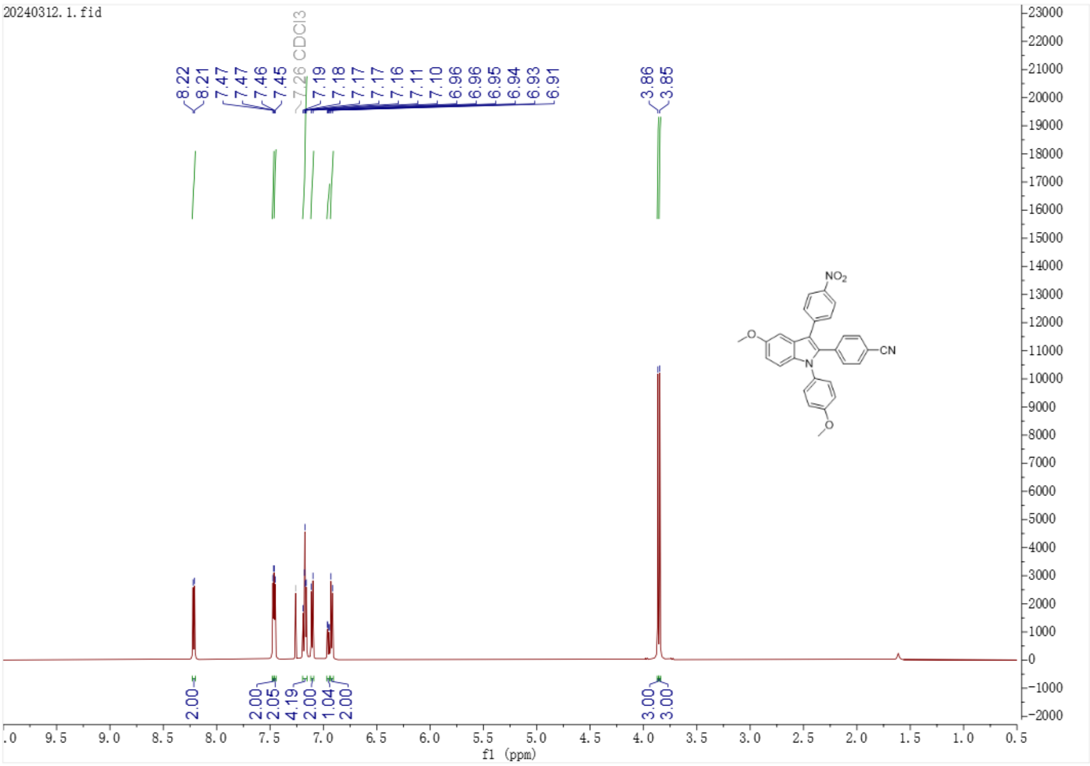

^{13}C NMR of compound **4d** (in $CDCl_3$)

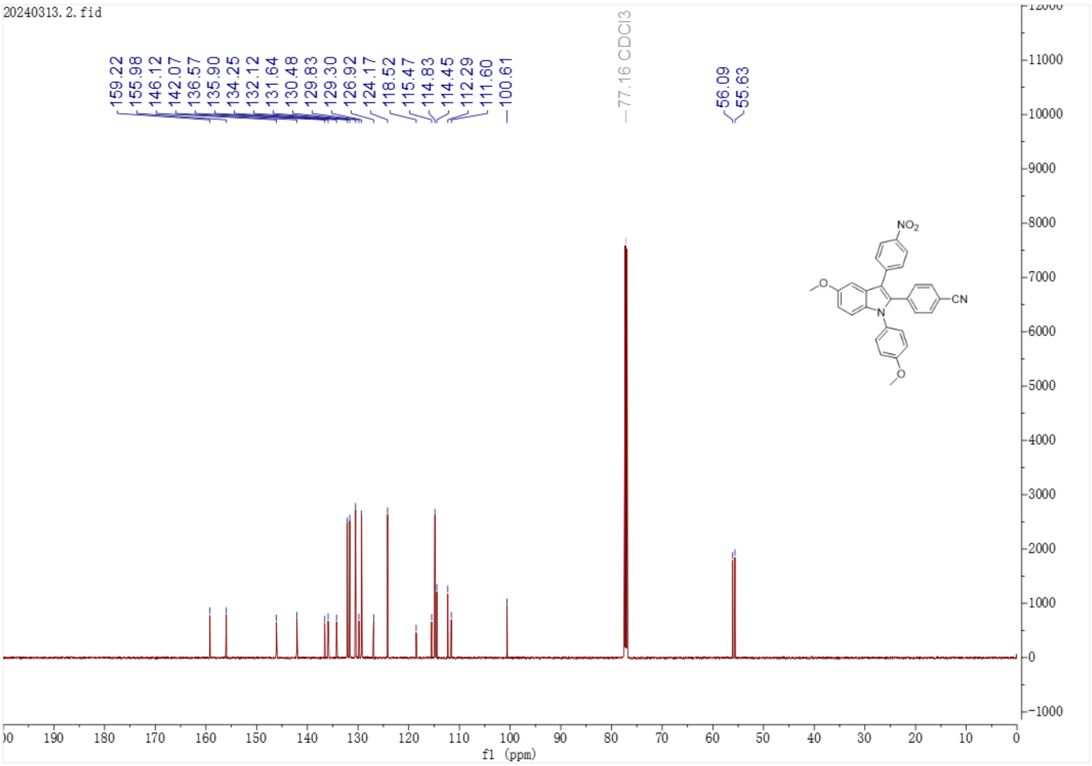

^{1}H NMR of compound **4e** (in $CDCl_3$)

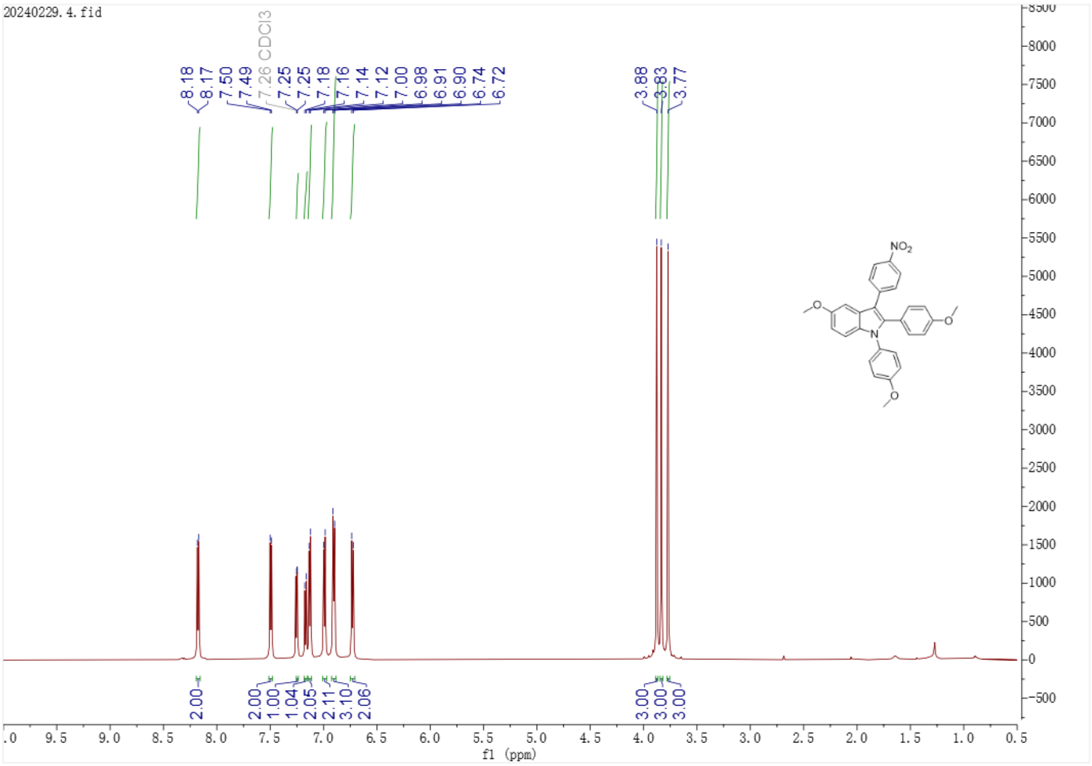

^{13}C NMR of compound **4e** (in $CDCl_3$)

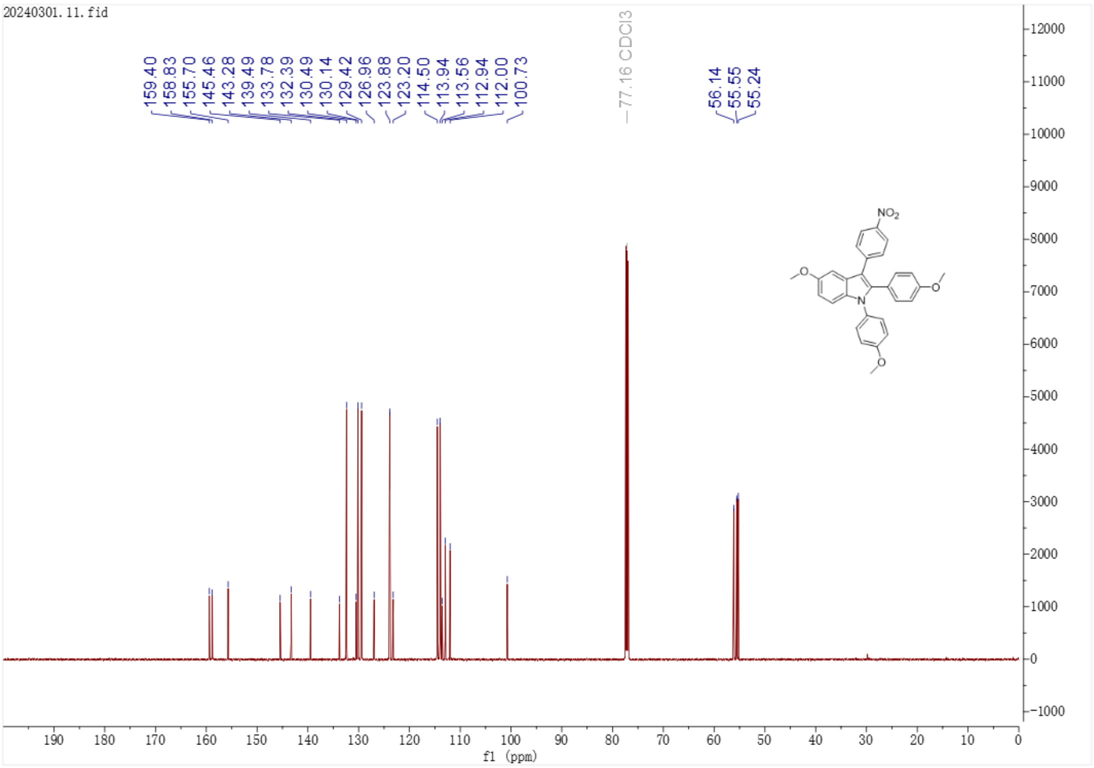

^{1}H NMR of compound **4f** (in $CDCl_3$)

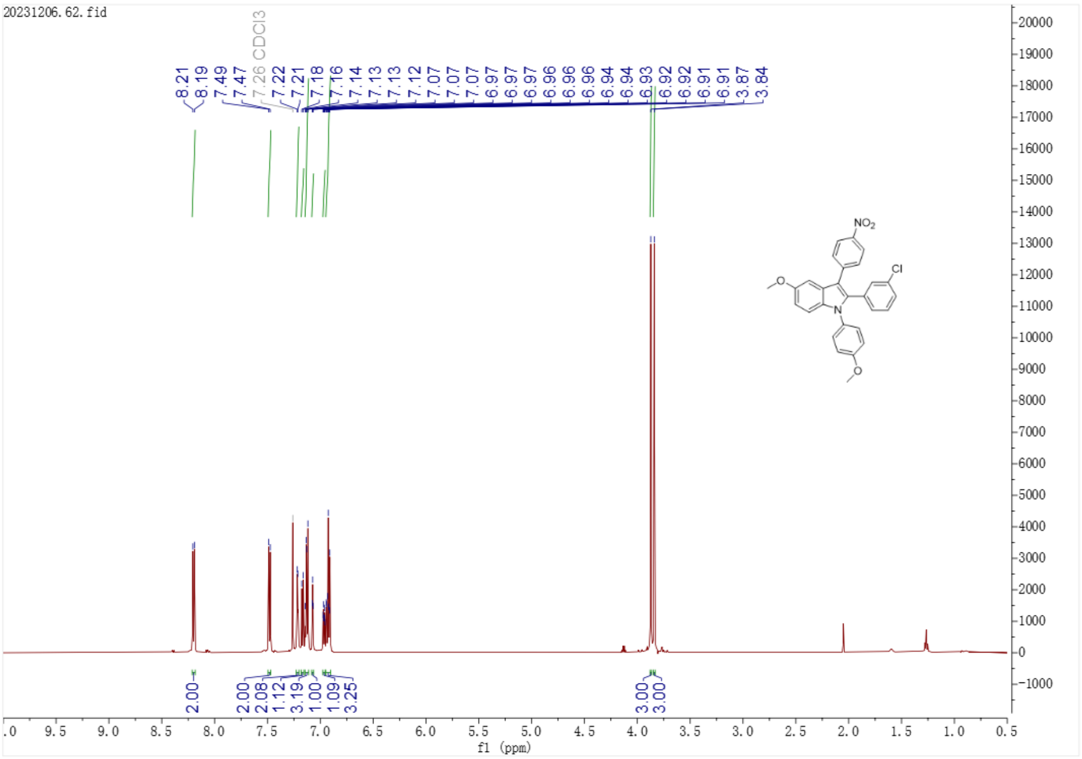

^{13}C NMR of compound **4f** (in $CDCl_3$)

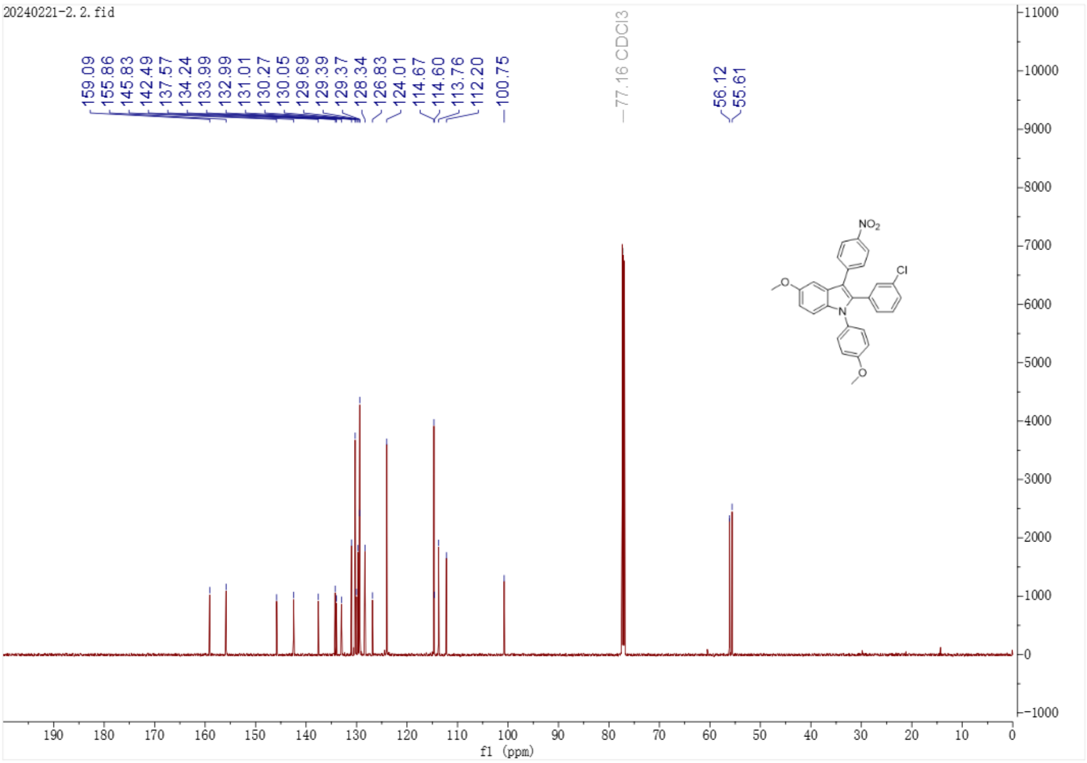

^{1}H NMR of compound **4g** (in $CDCl_3$)

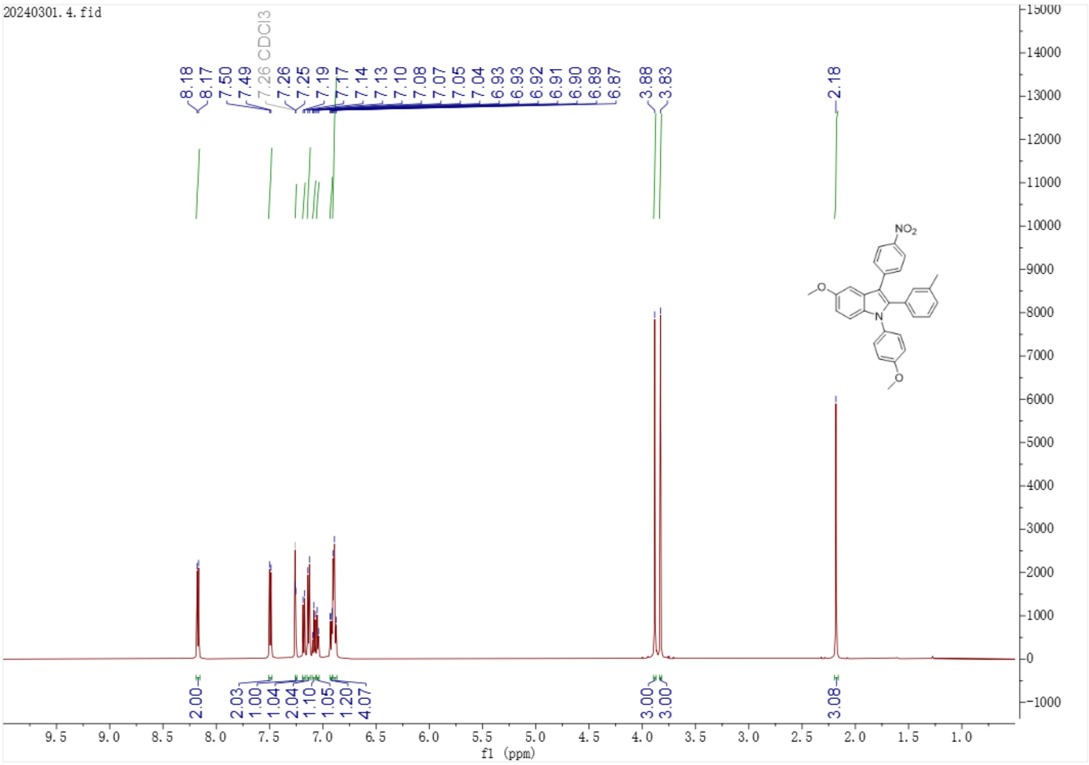

^{13}C NMR of compound **4g** (in $CDCl_3$)

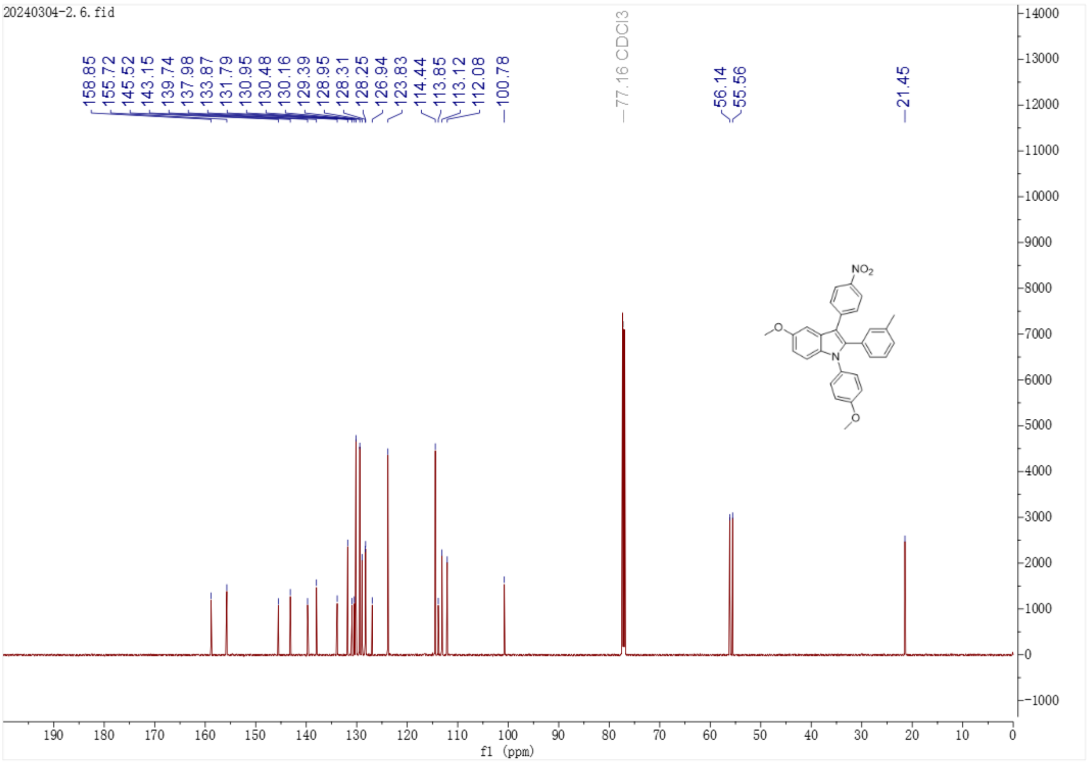

^{1}H NMR of compound **4h** (in $CDCl_3$)

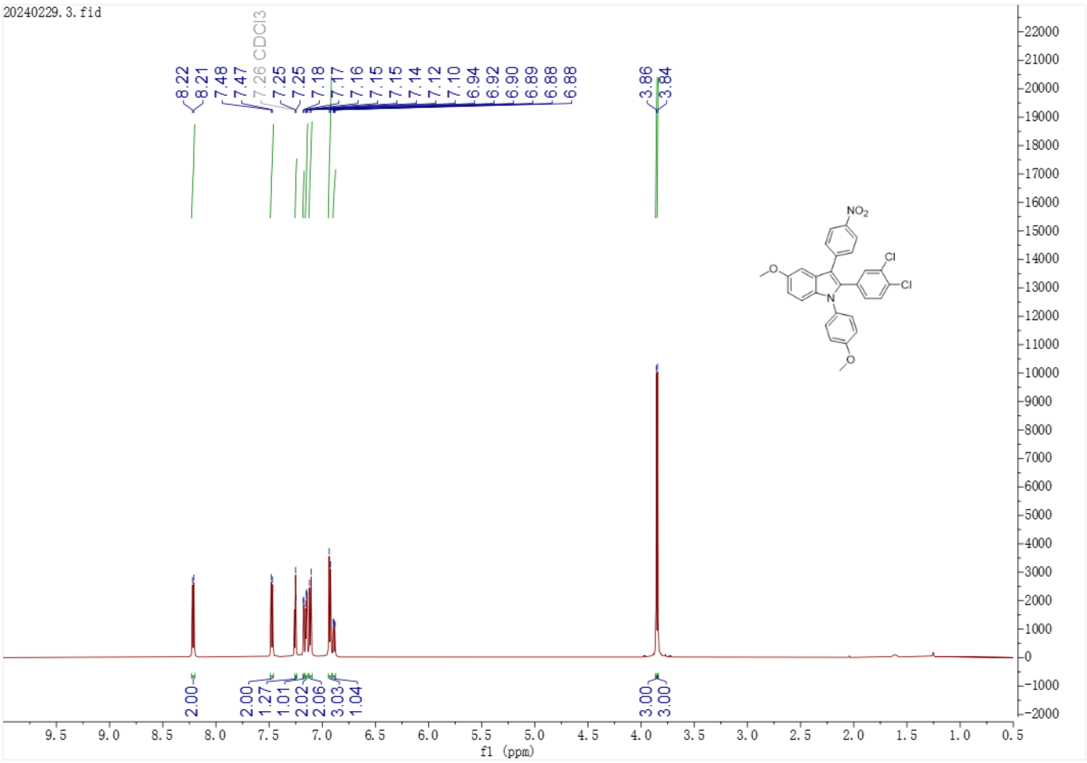

^{13}C NMR of compound **4h** (in $CDCl_3$)

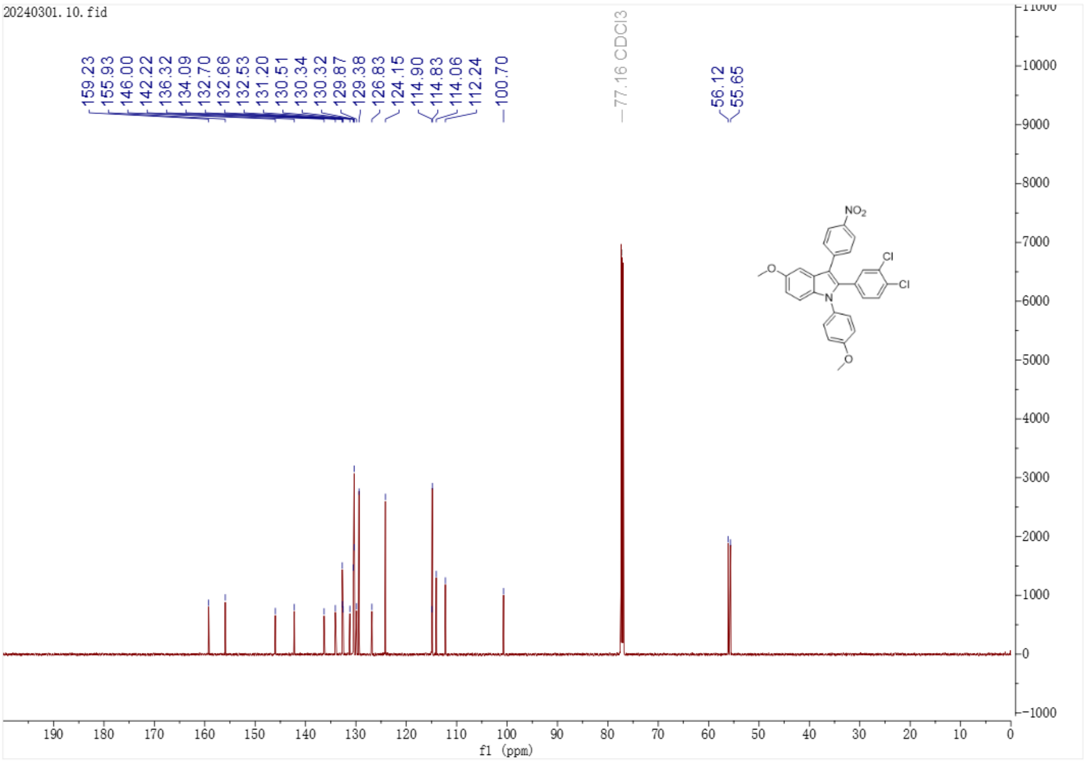

^{1}H NMR of compound **4i** (in $CDCl_3$)

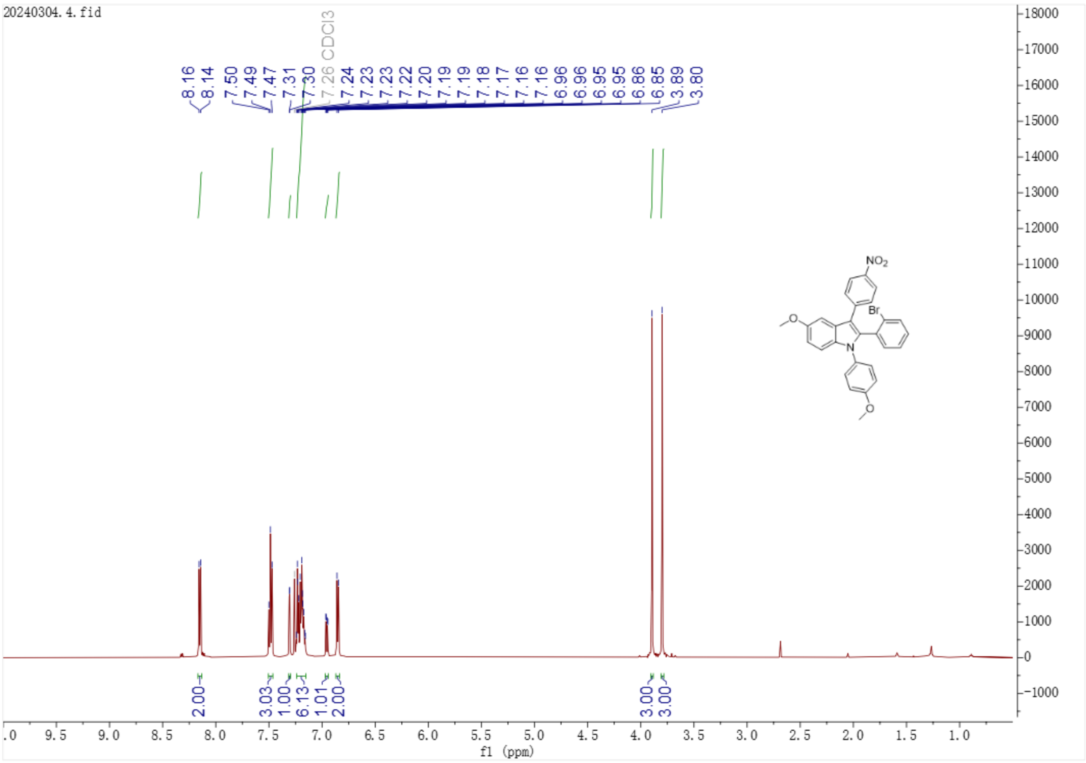

^{13}C NMR of compound **4i** (in $CDCl_3$)

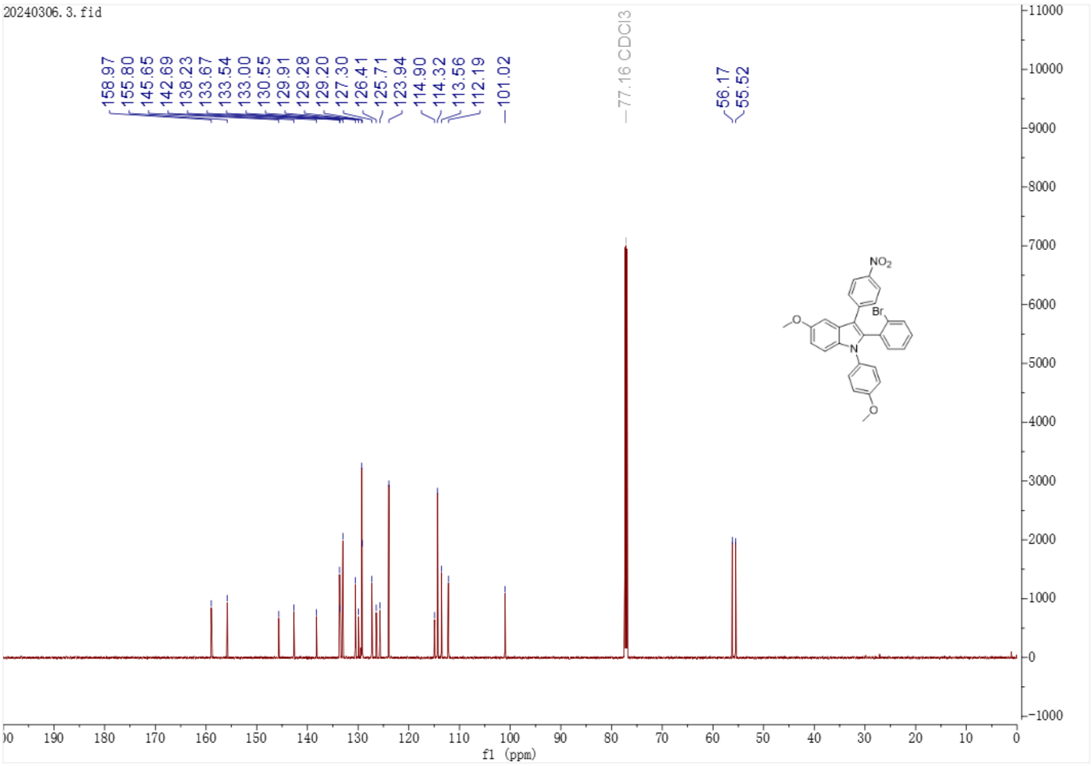

^{1}H NMR of compound **4j** (in $CDCl_3$)

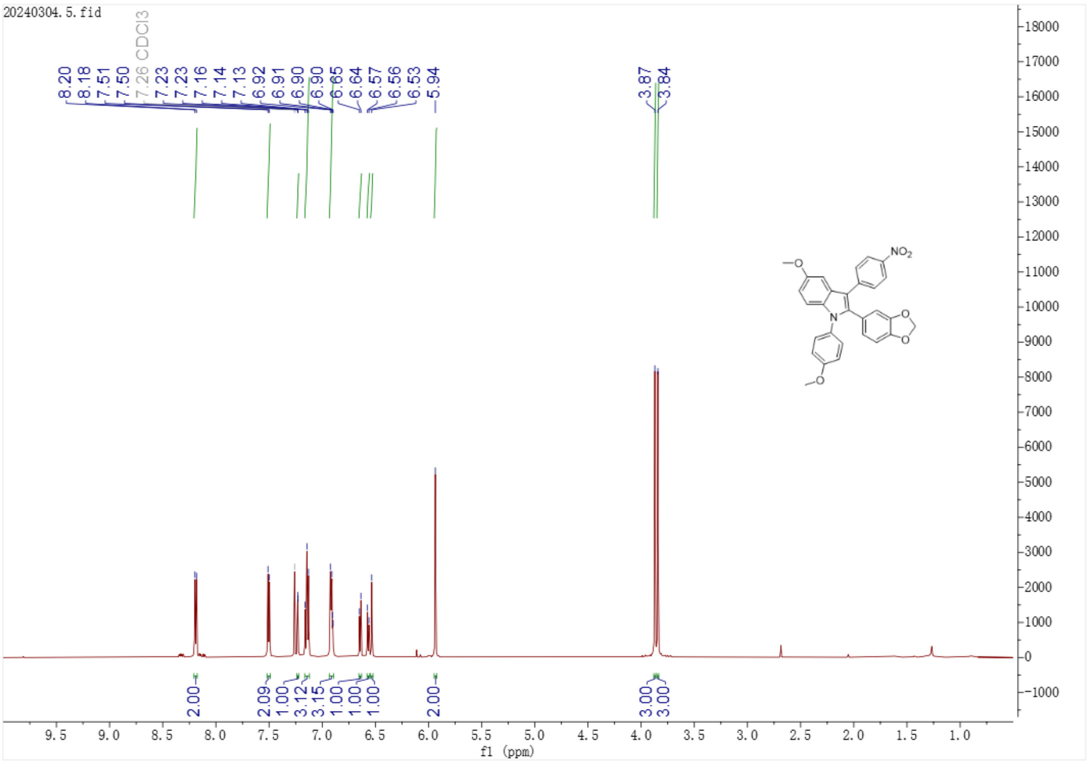

^{13}C NMR of compound **4j** (in $CDCl_3$)

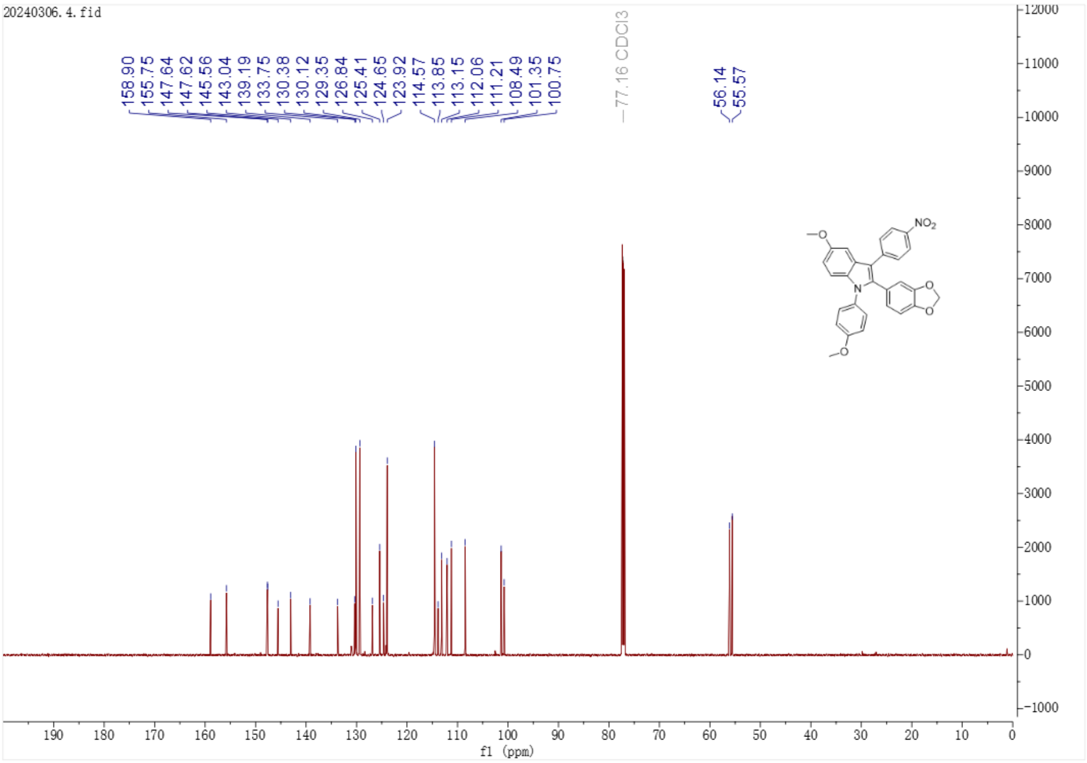

^{1}H NMR of compound **4k** (in $CDCl_3$)

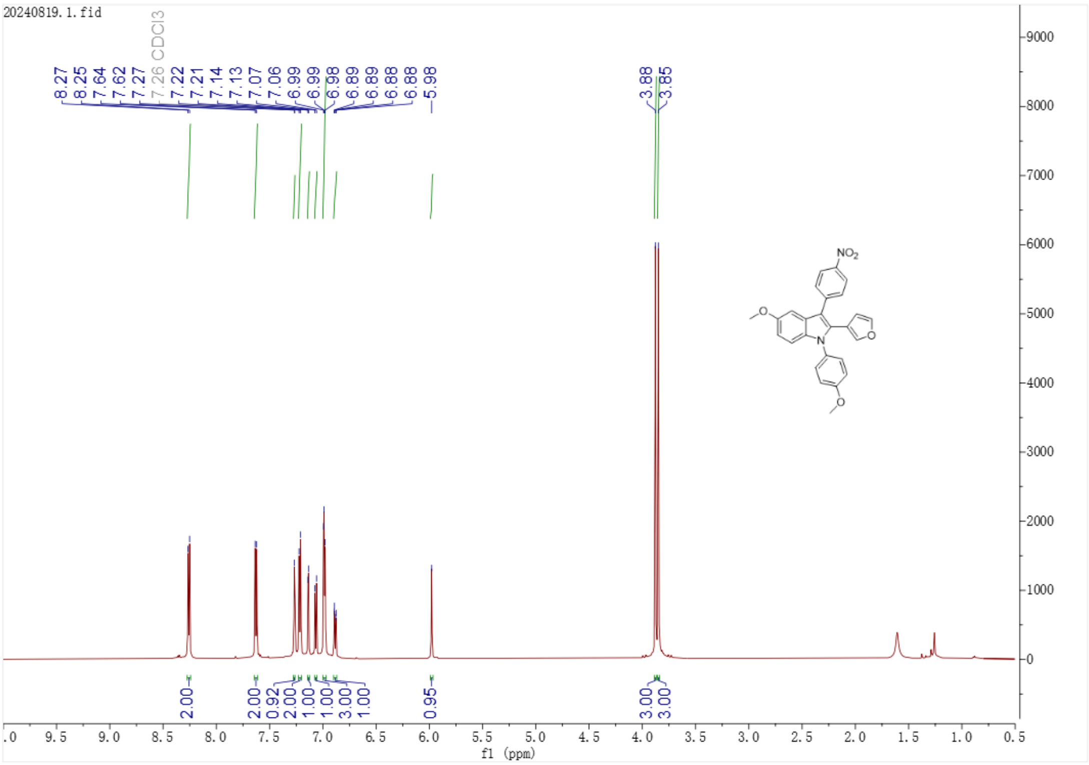

^{13}C NMR of compound **4k** (in $CDCl_3$)

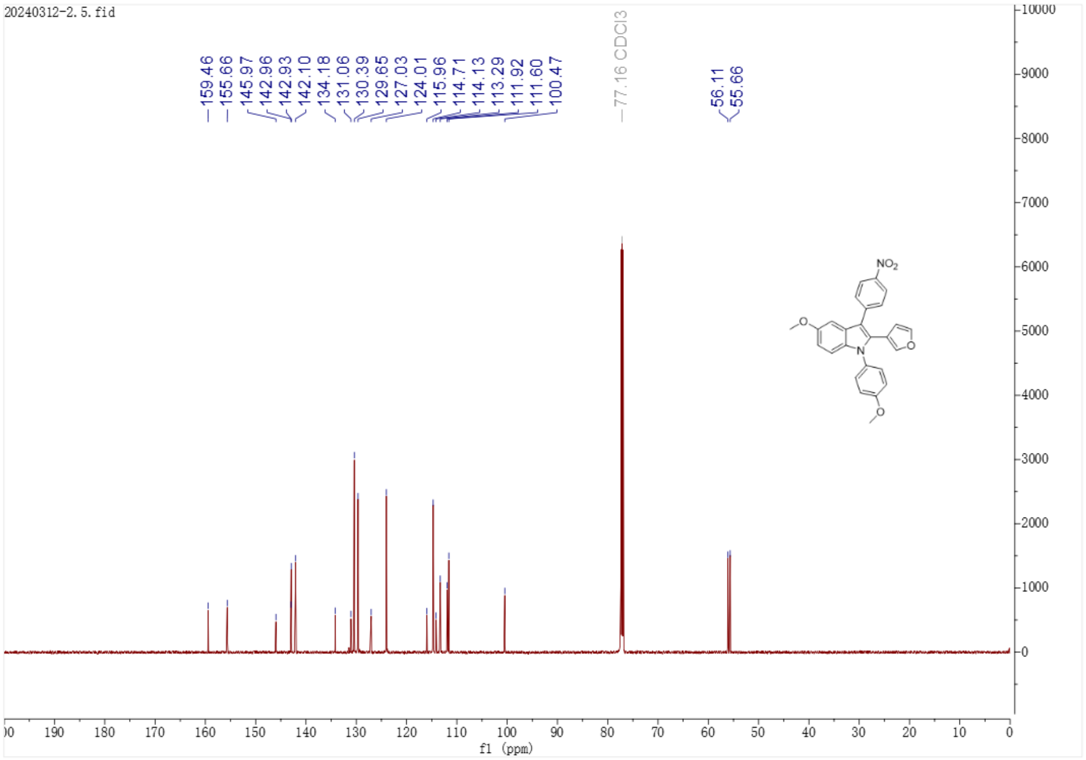

^{1}H NMR of compound **4l** (in $CDCl_3$)

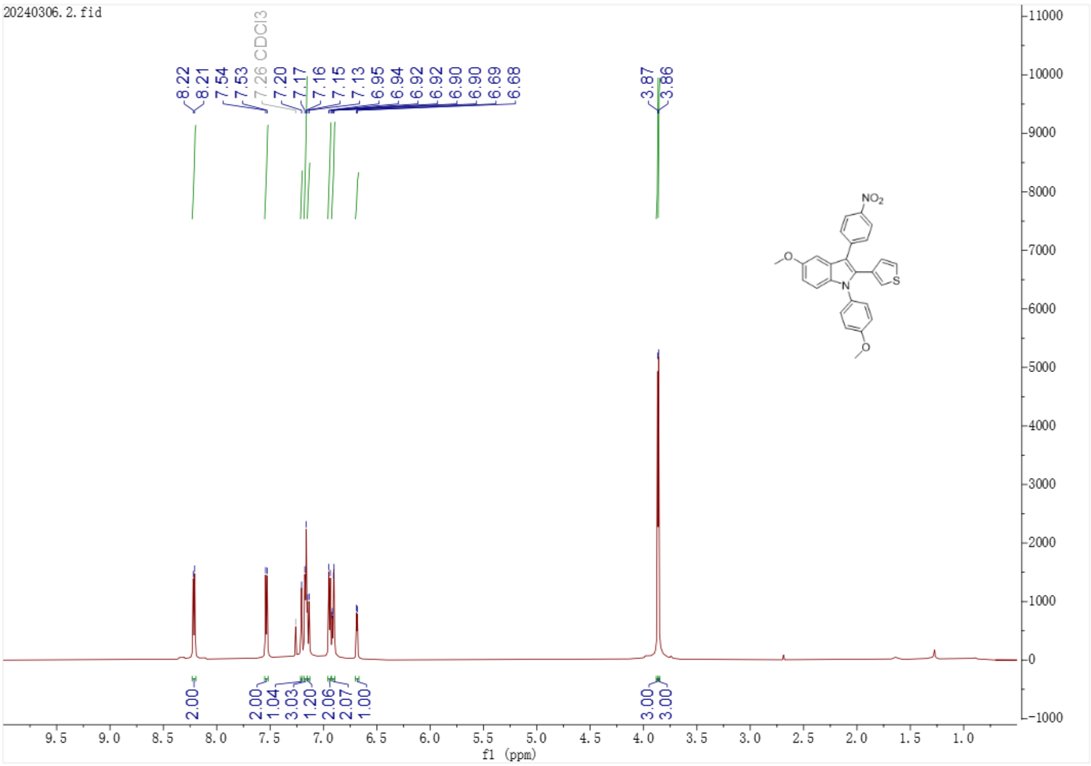

^{13}C NMR of compound **4l** (in $CDCl_3$)

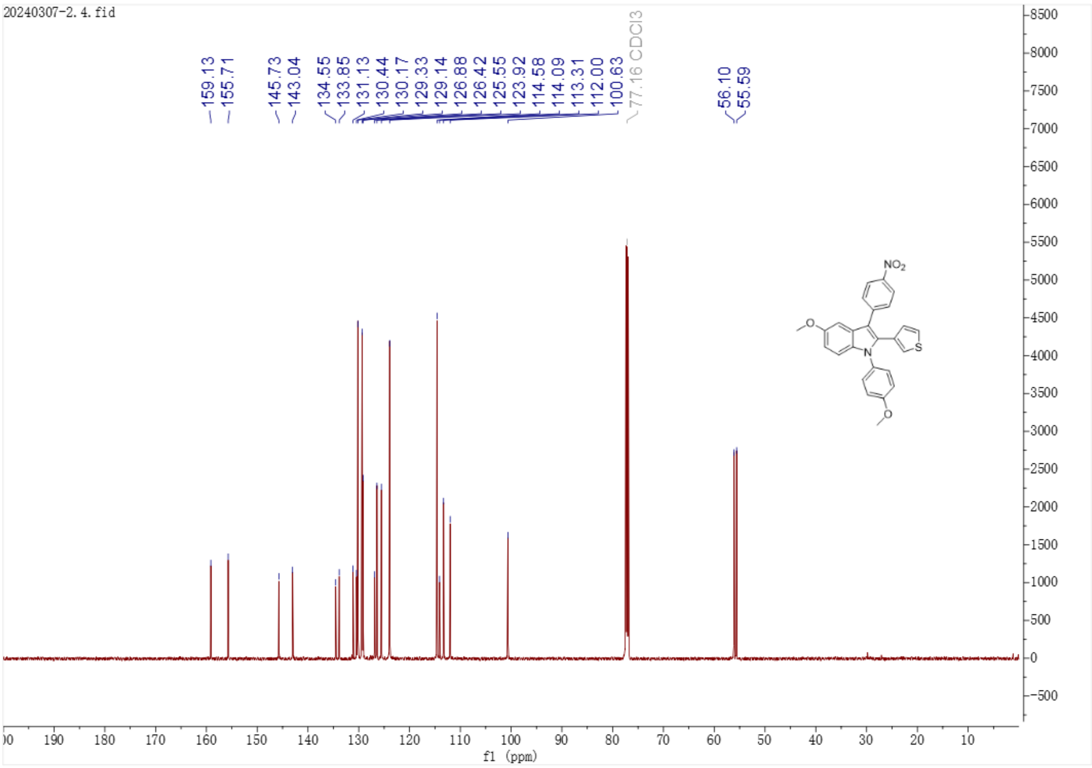

^{1}H NMR of compound **4m** (in $CDCl_3$)

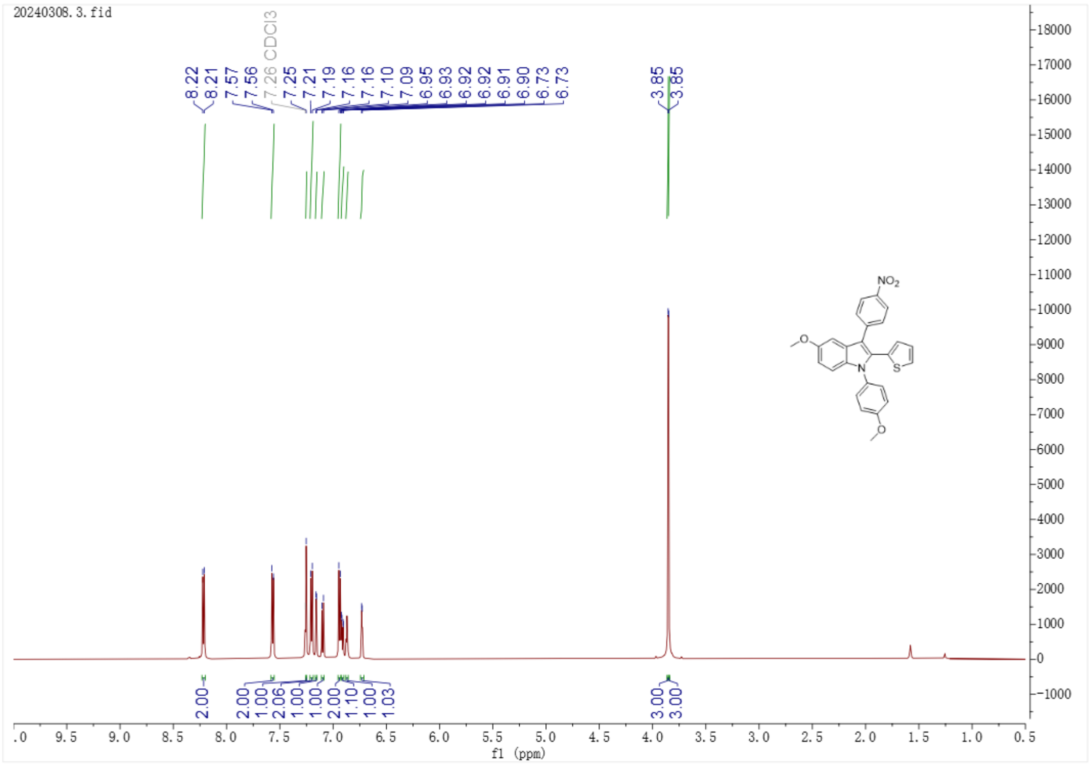

^{13}C NMR of compound **4m** (in $CDCl_3$)

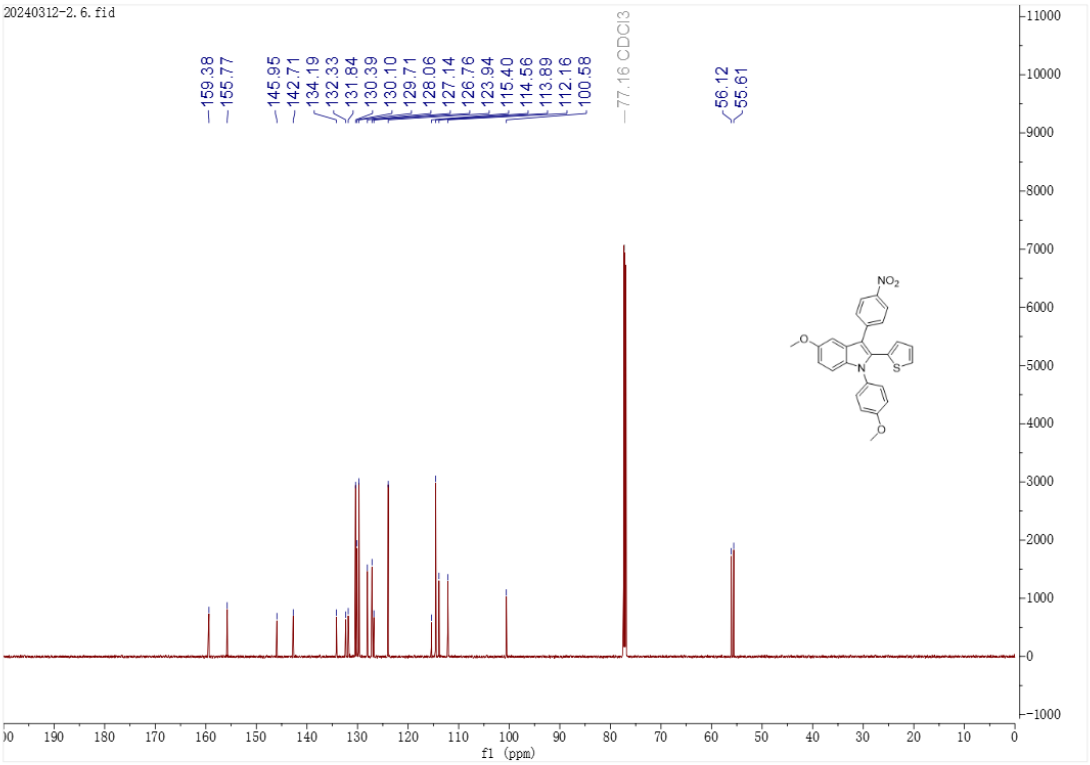

^{1}H NMR of compound **4n** (in $CDCl_3$)

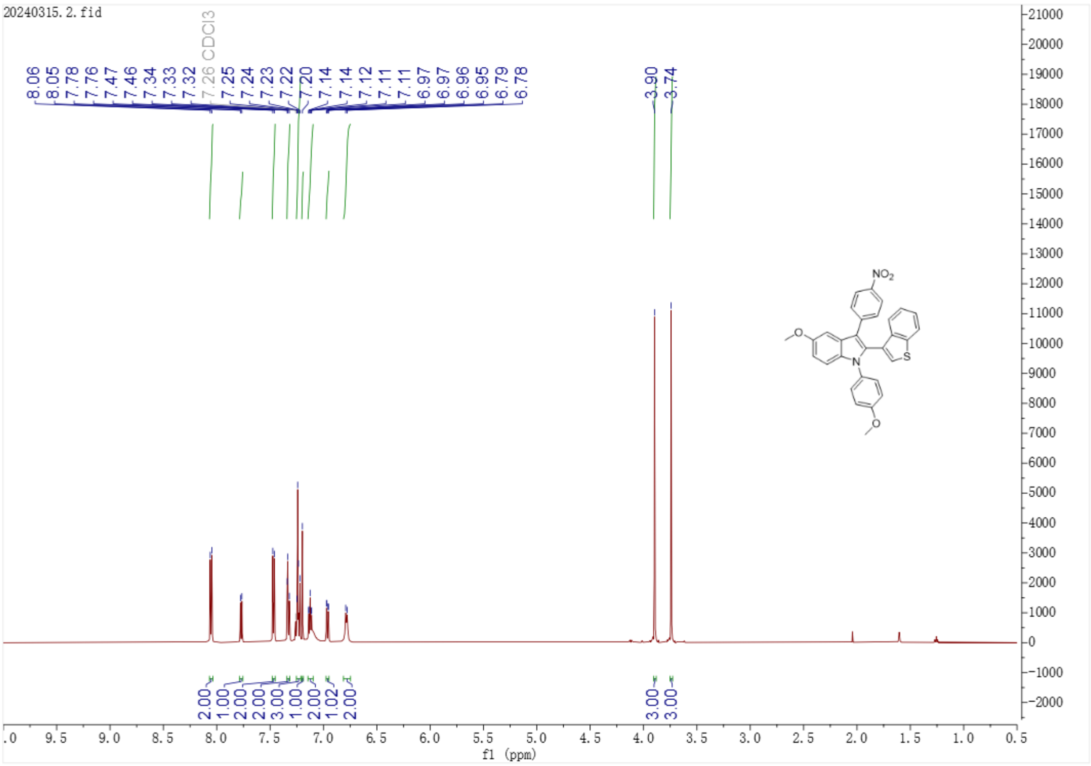

^{13}C NMR of compound **4n** (in $CDCl_3$)

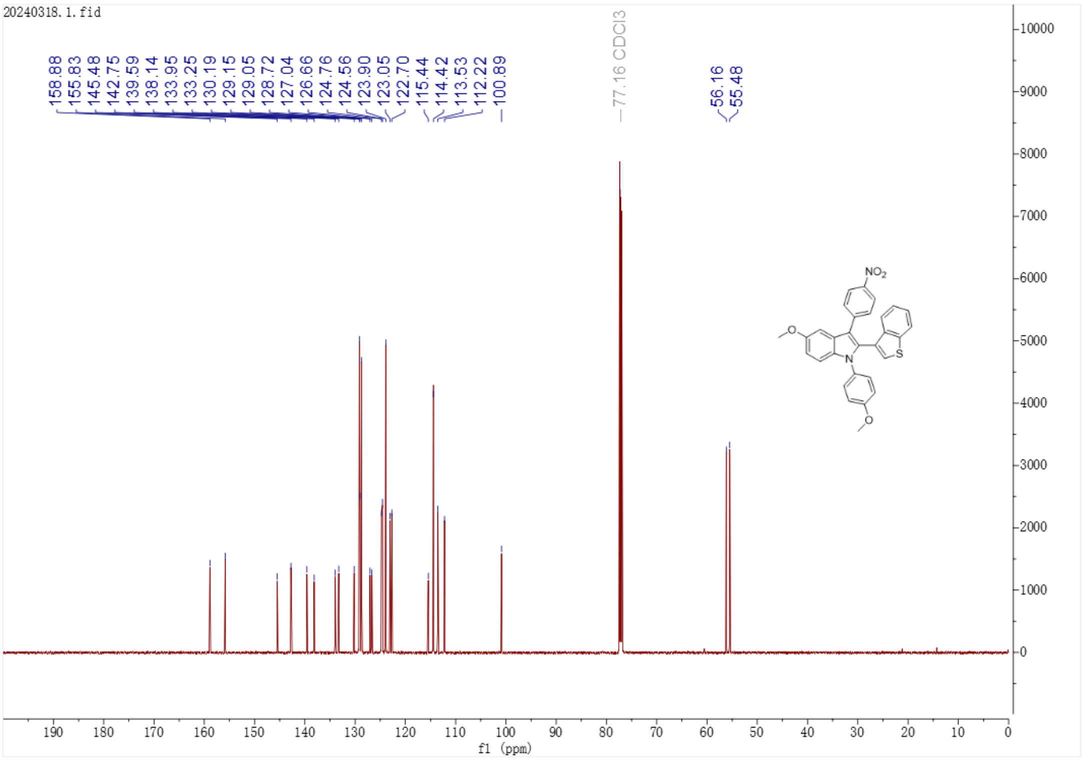

^{1}H NMR of compound **4o** (in $CDCl_3$)

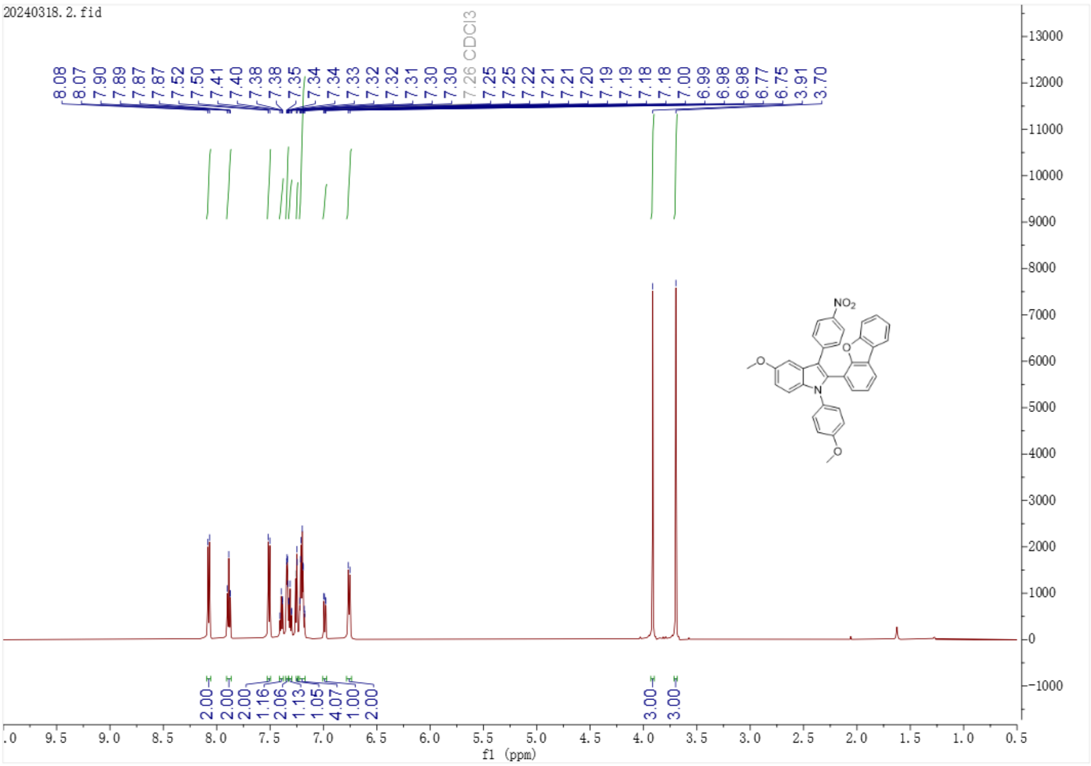

^{13}C NMR of compound **4o** (in $CDCl_3$)

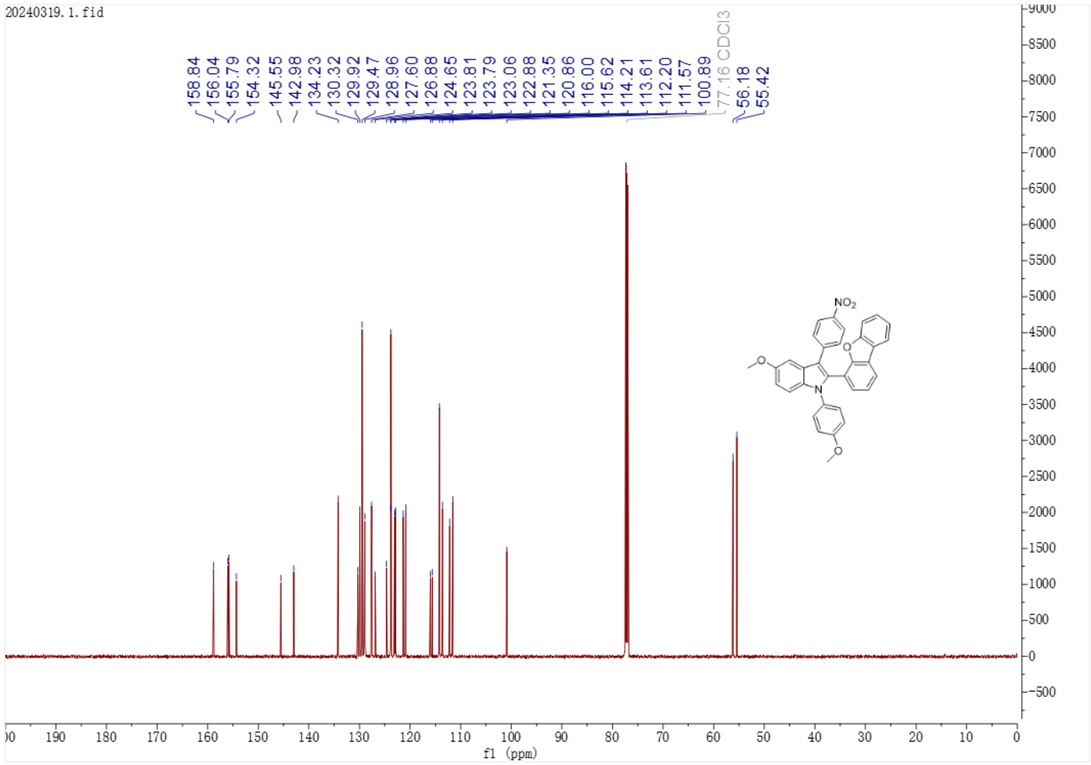

^{1}H NMR of compound **4p** (in $CDCl_3$)

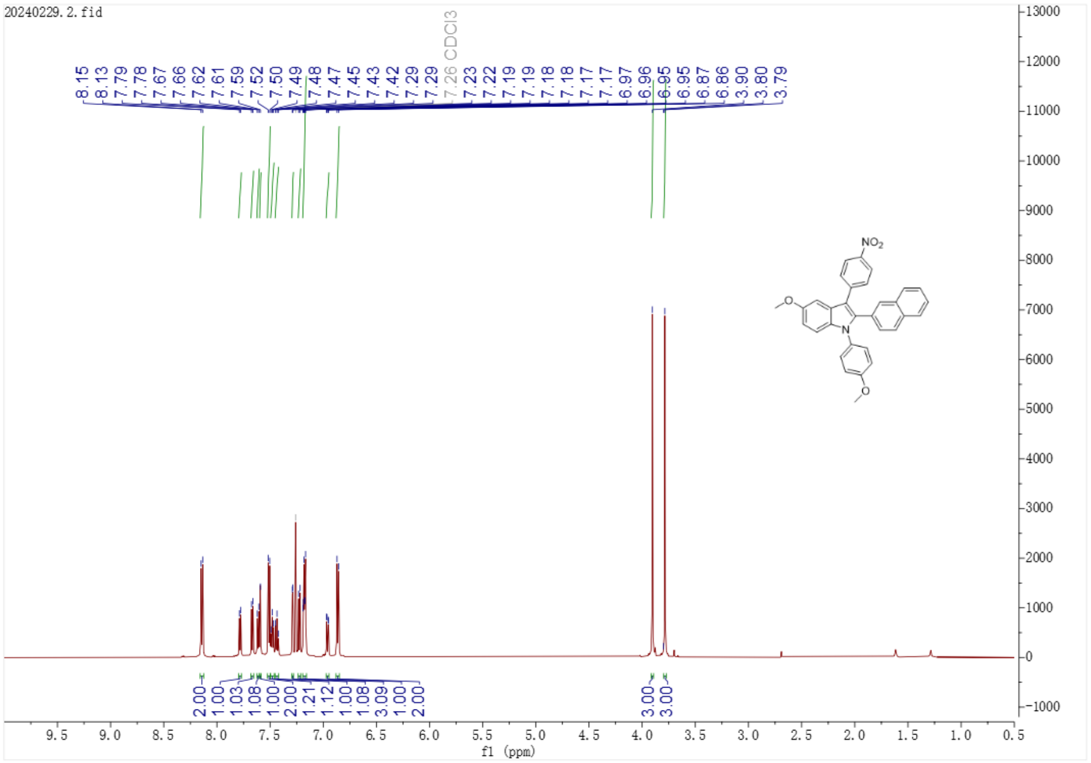

^{13}C NMR of compound **4p** (in $CDCl_3$)

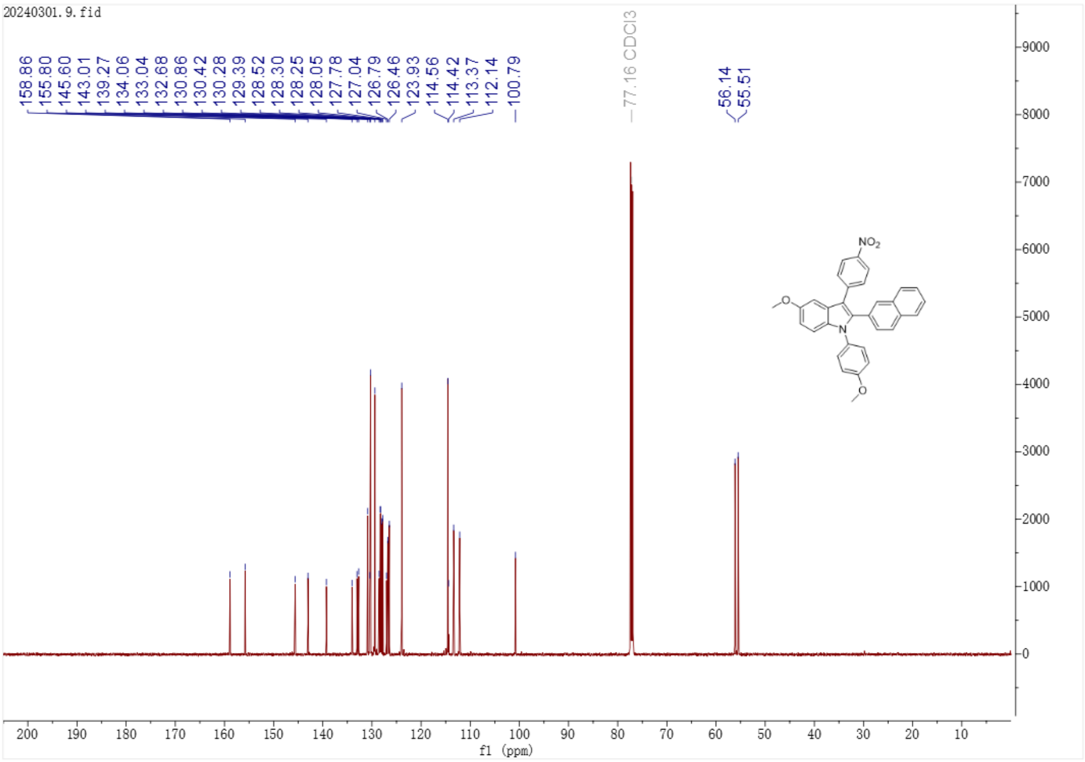

^{1}H NMR of compound **4q** (in $CDCl_3$)

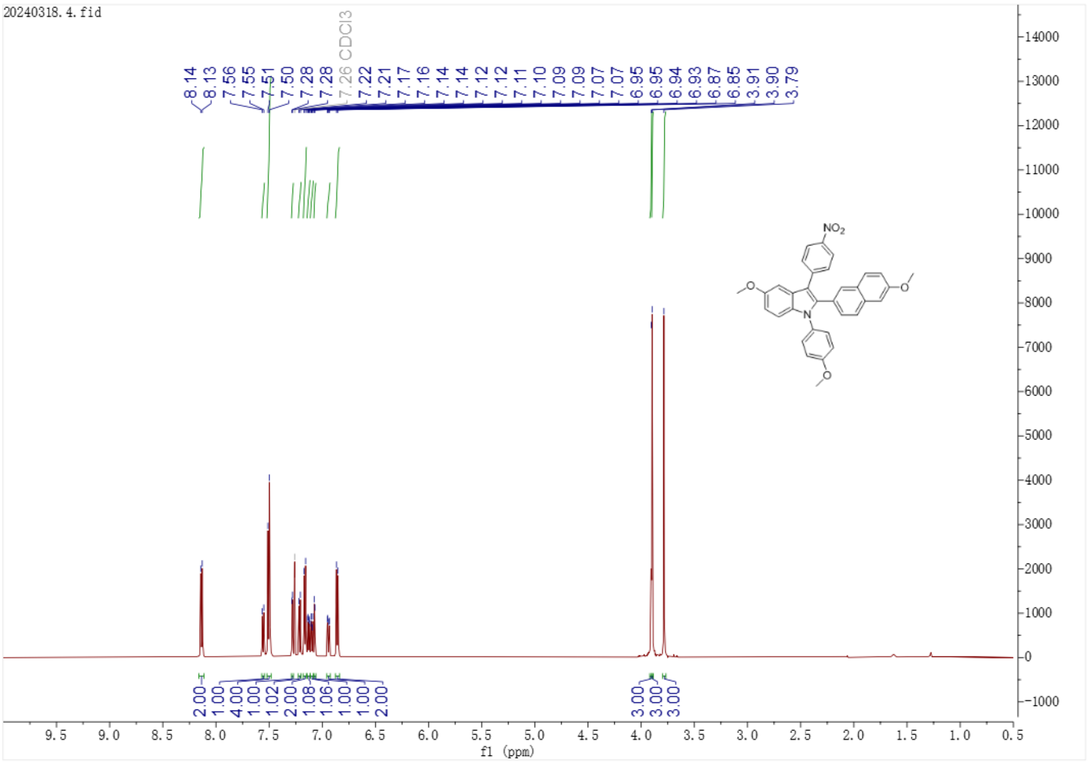

^{13}C NMR of compound **4q** (in $CDCl_3$)

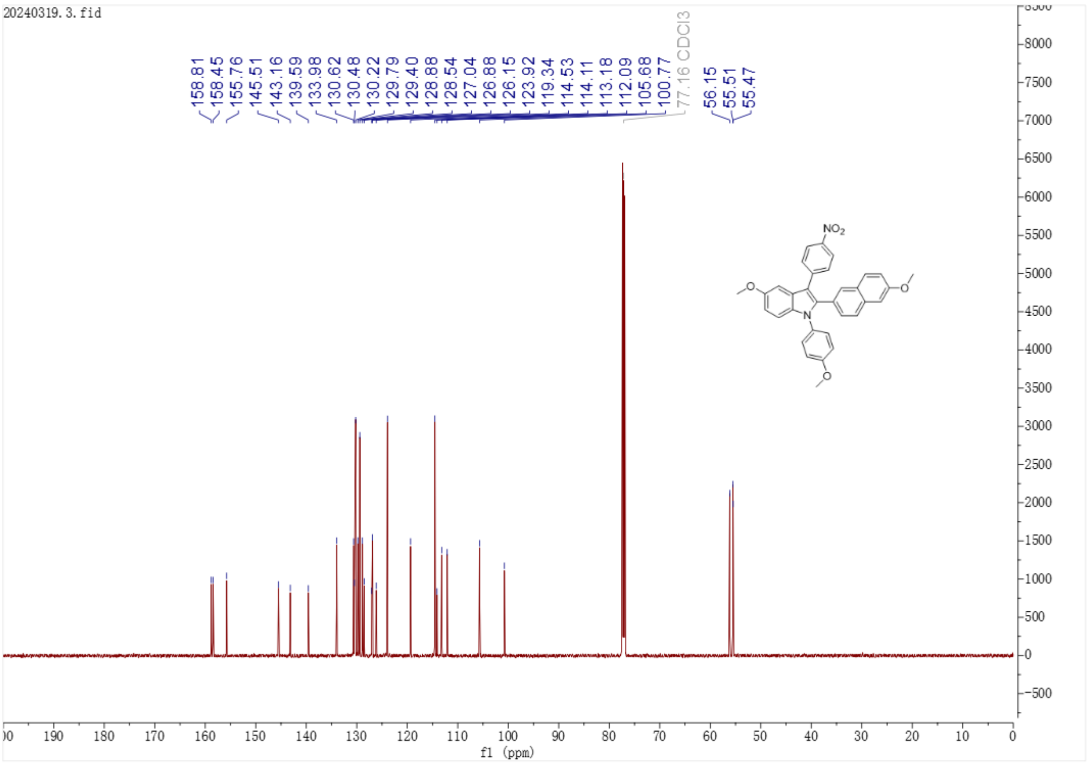

^{1}H NMR of compound **4r** (in $CDCl_3$)

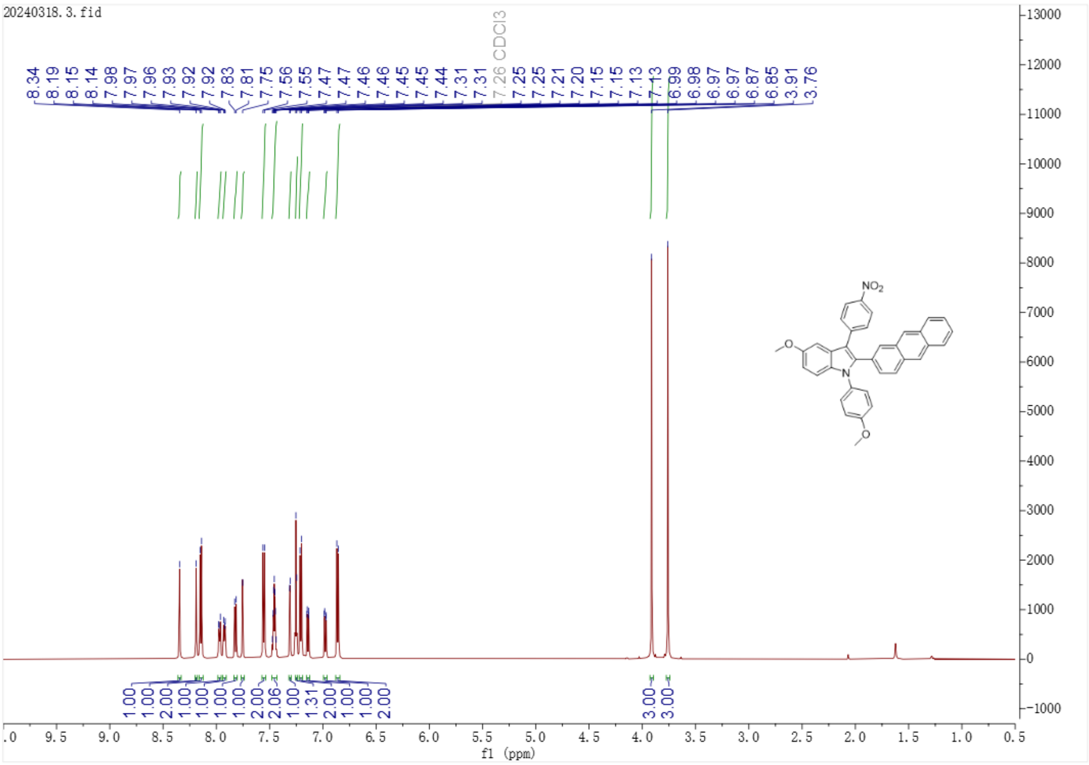

^{13}C NMR of compound **4r** (in $CDCl_3$)

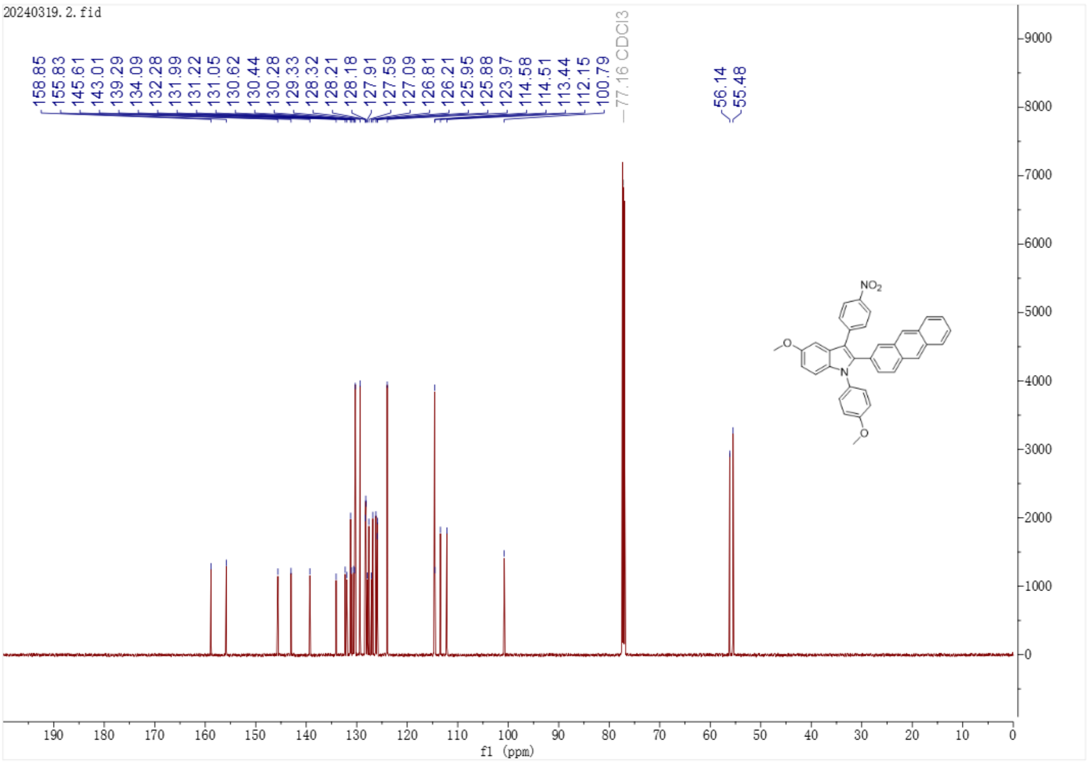

^{1}H NMR of compound **4s** (in $CDCl_3$)

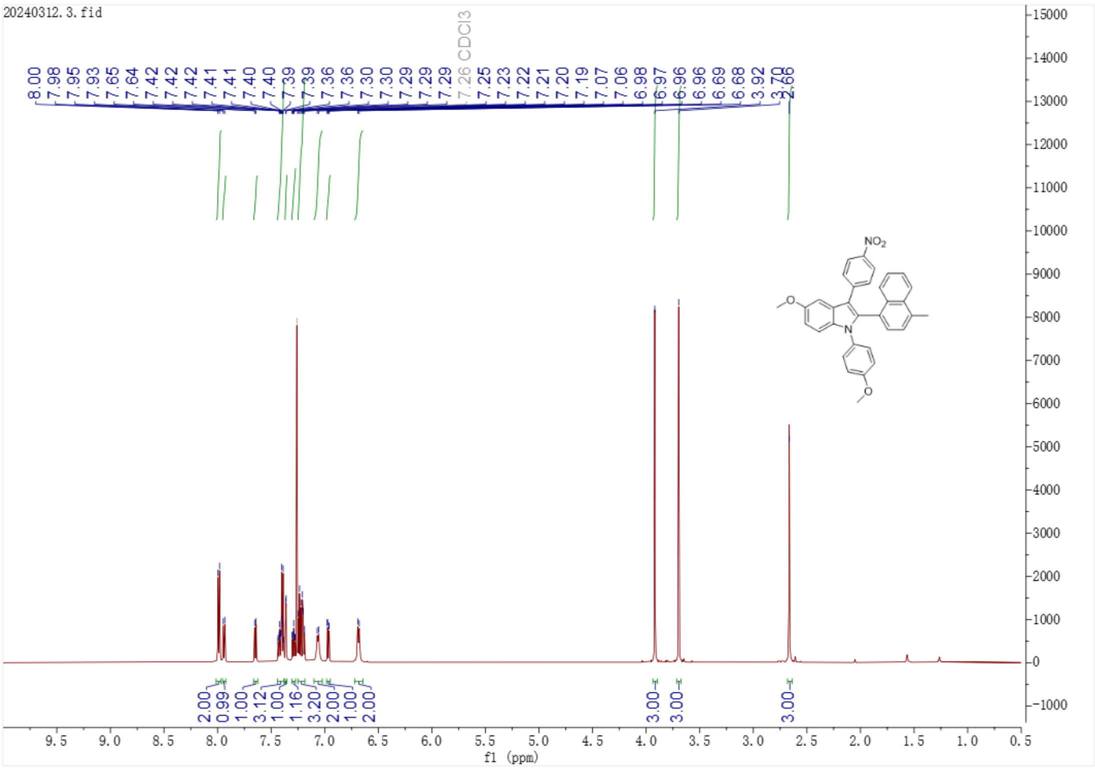

^{13}C NMR of compound **4s** (in $CDCl_3$)

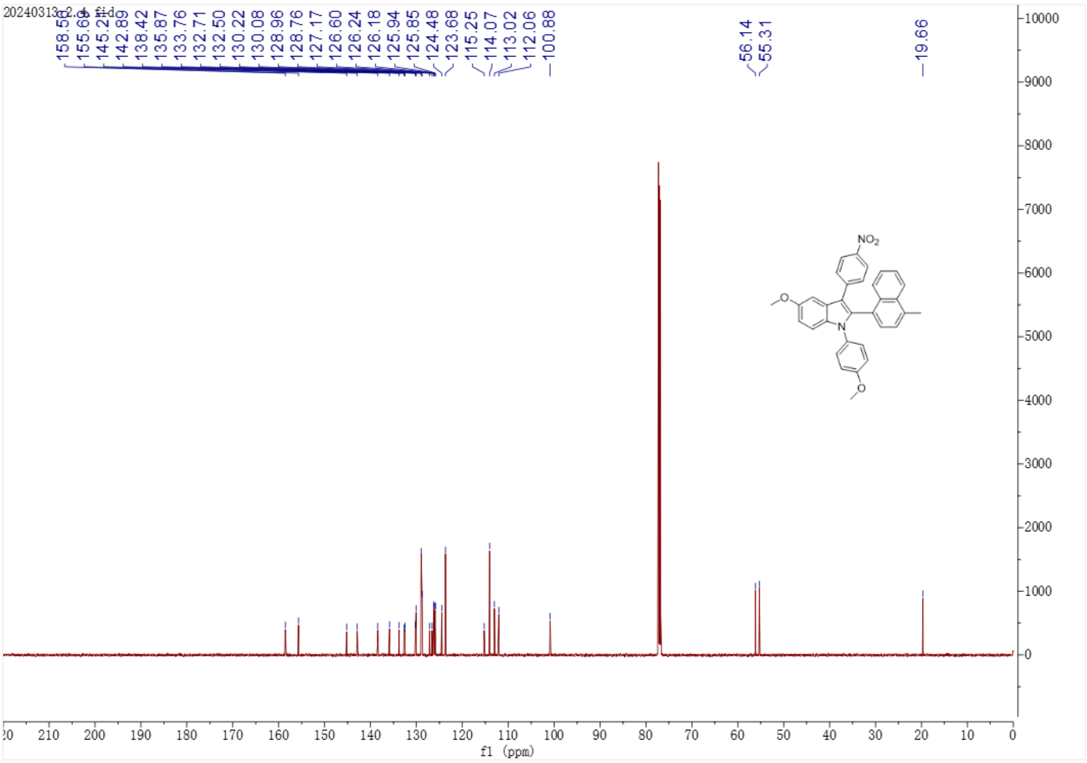

^{1}H NMR of compound **4t** (in $CDCl_3$)

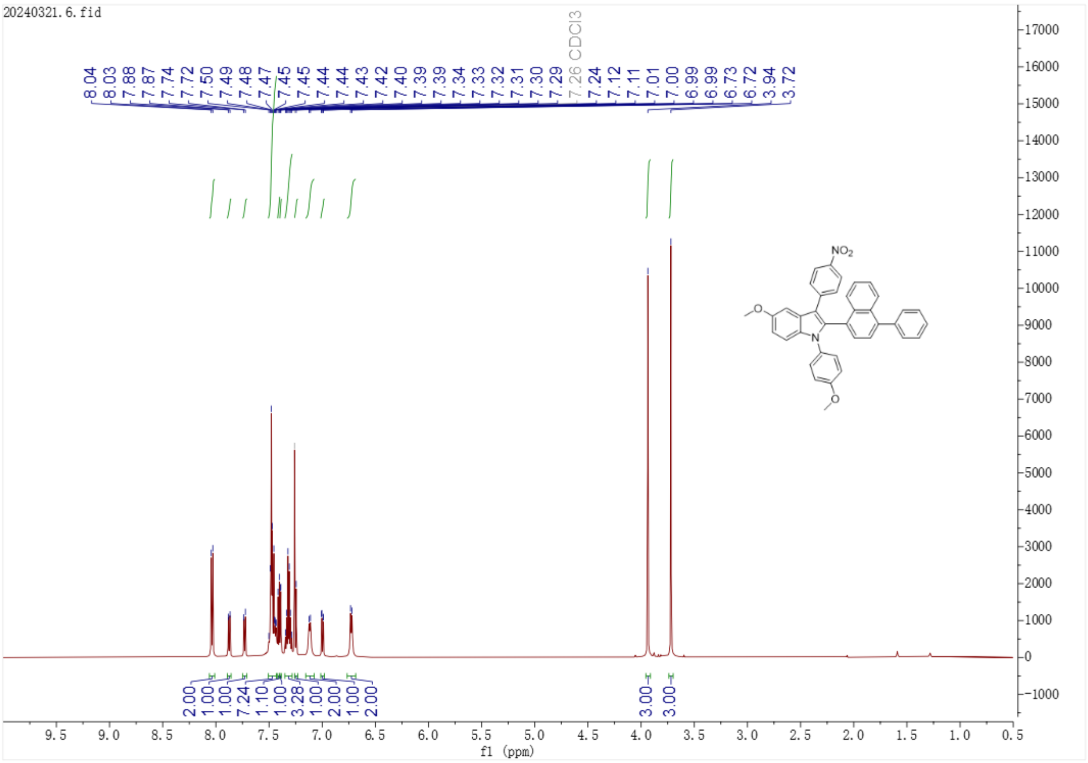

^{13}C NMR of compound **4t** (in $CDCl_3$)

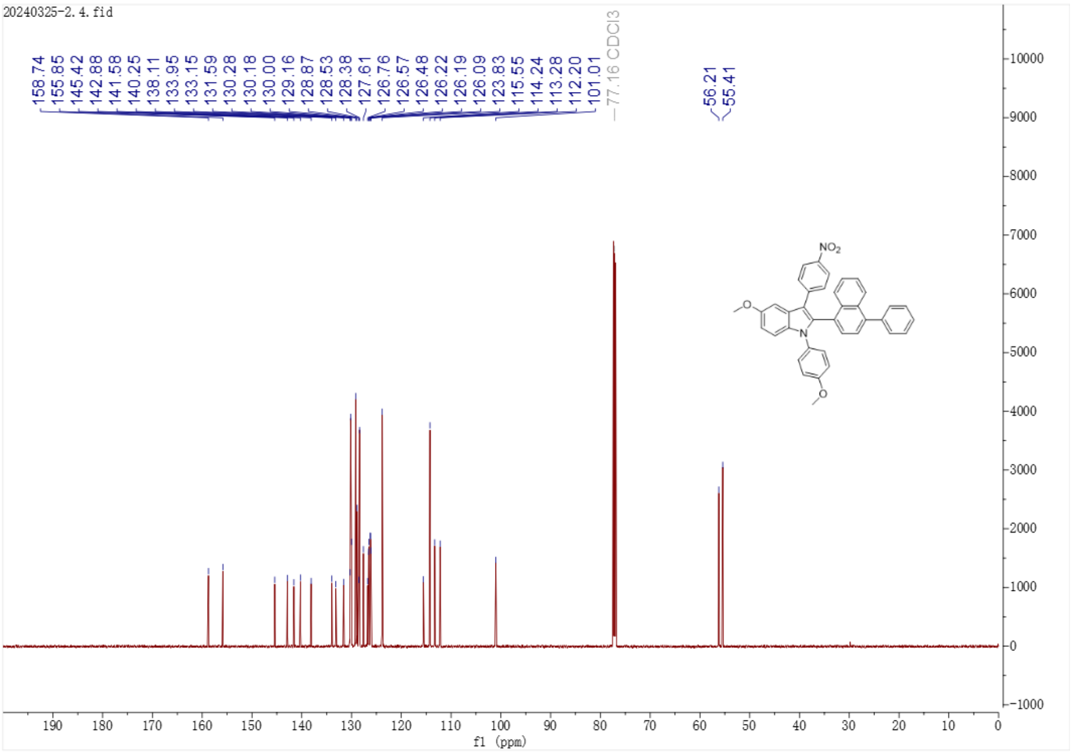

^{1}H NMR of compound **4u** (in $CDCl_3$)

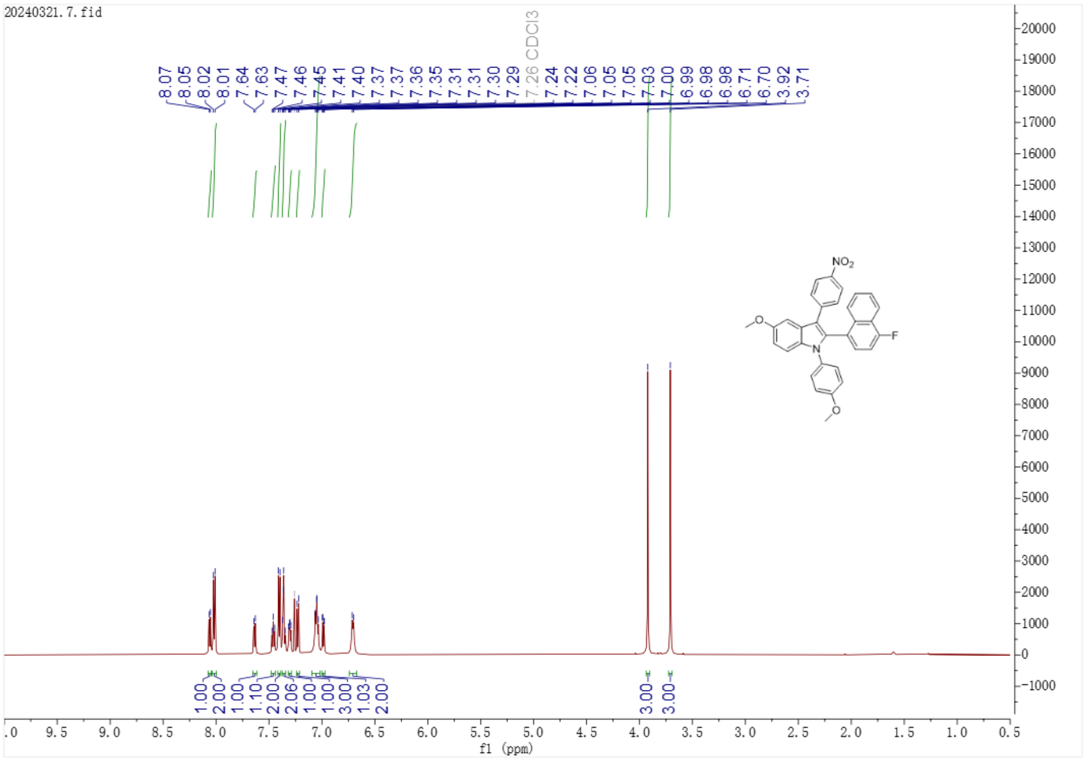

^{13}C NMR of compound **4u** (in $CDCl_3$)

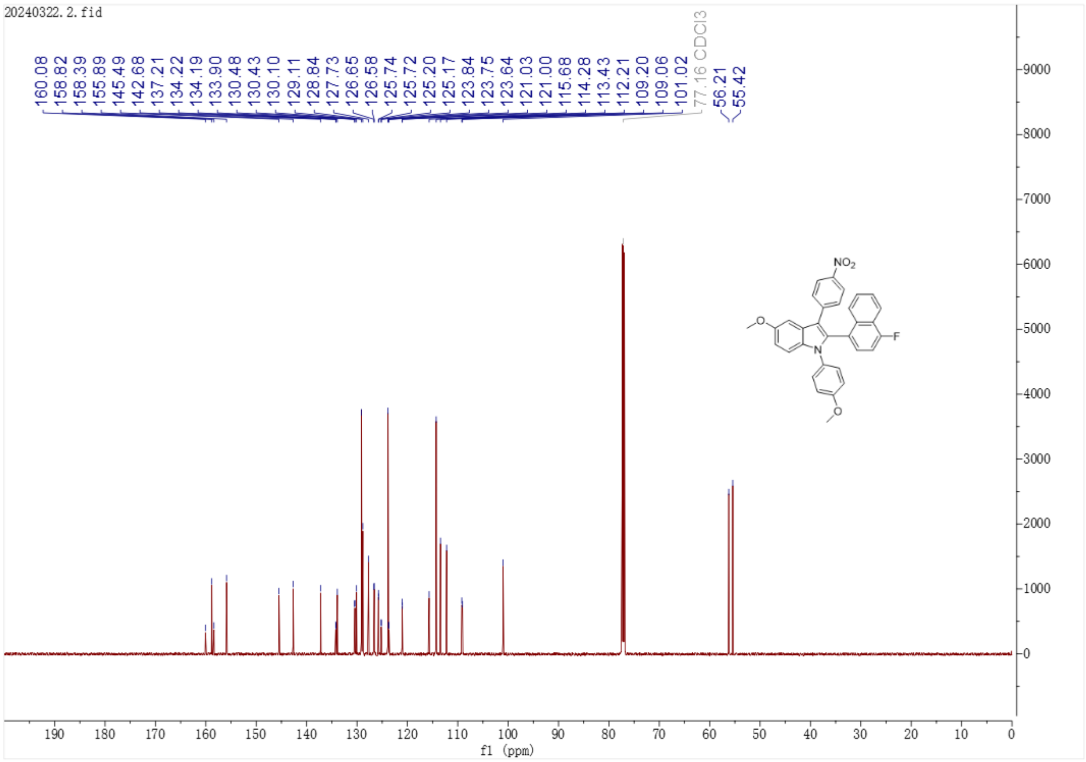

^{19}F NMR of compound **4u** (in $CDCl_3$)

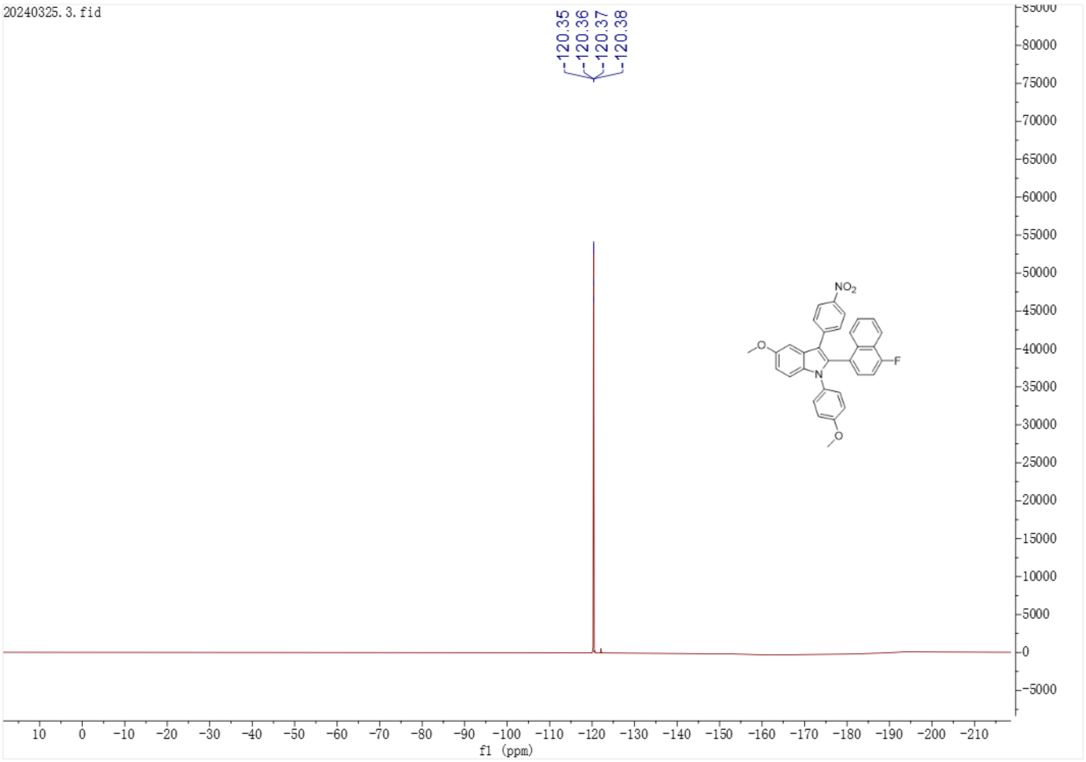

^{1}H NMR of compound **4v** (in $CDCl_3$)

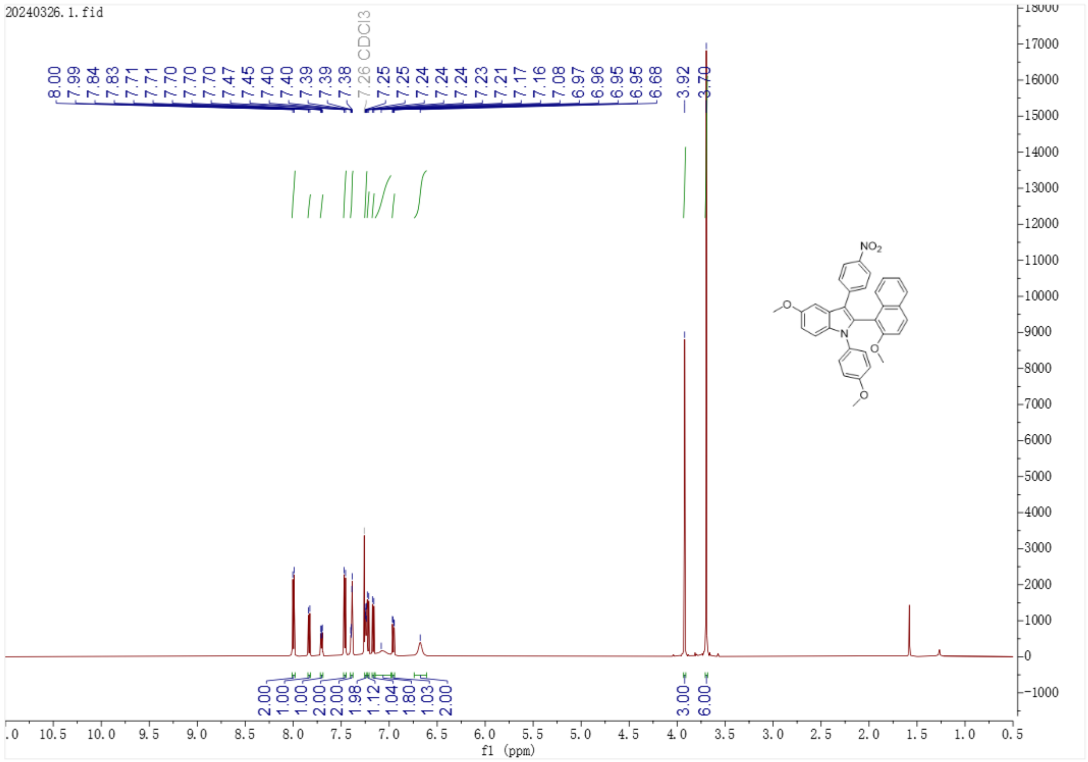

^{13}C NMR of compound **4v** (in $CDCl_3$)

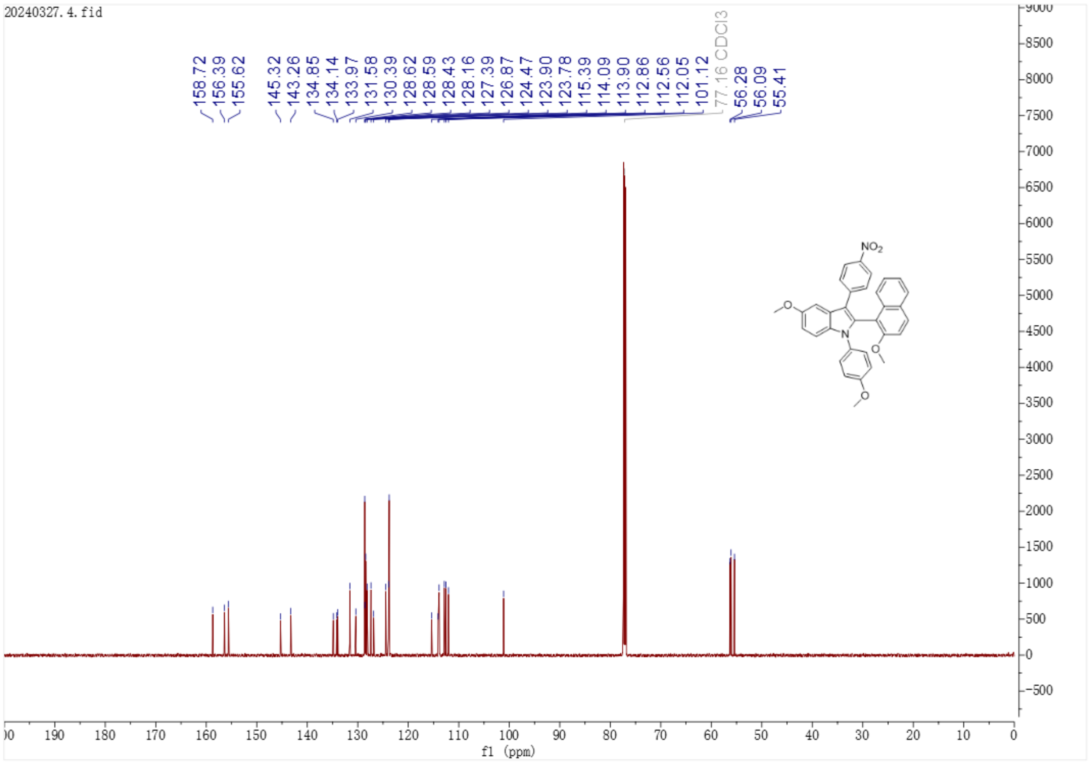

^{1}H NMR of compound **4w** (in $CDCl_3$)

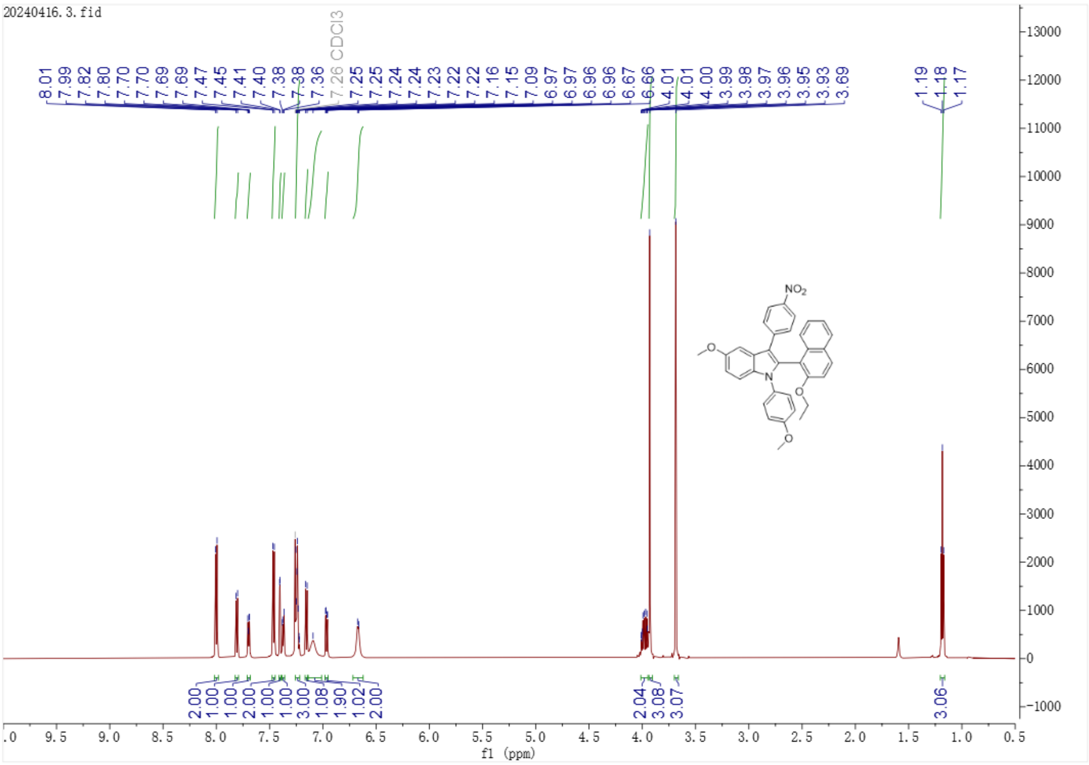

^{13}C NMR of compound **4w** (in $CDCl_3$)

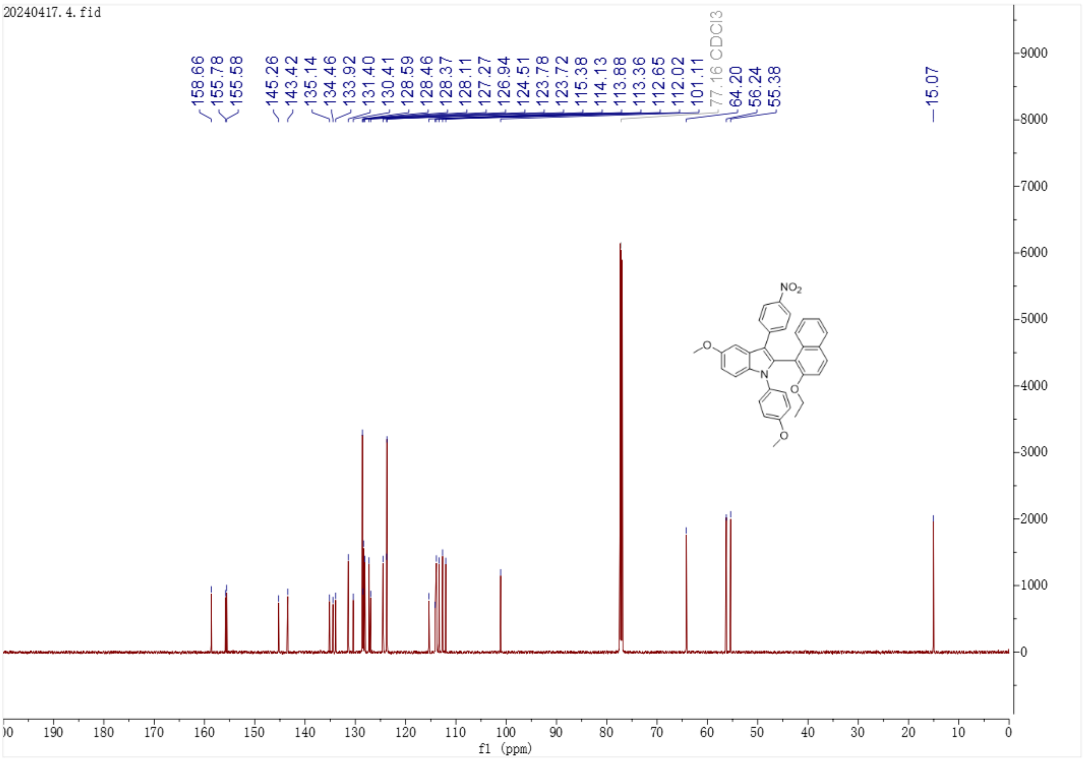

^{1}H NMR of compound **4x** (in $CDCl_3$)

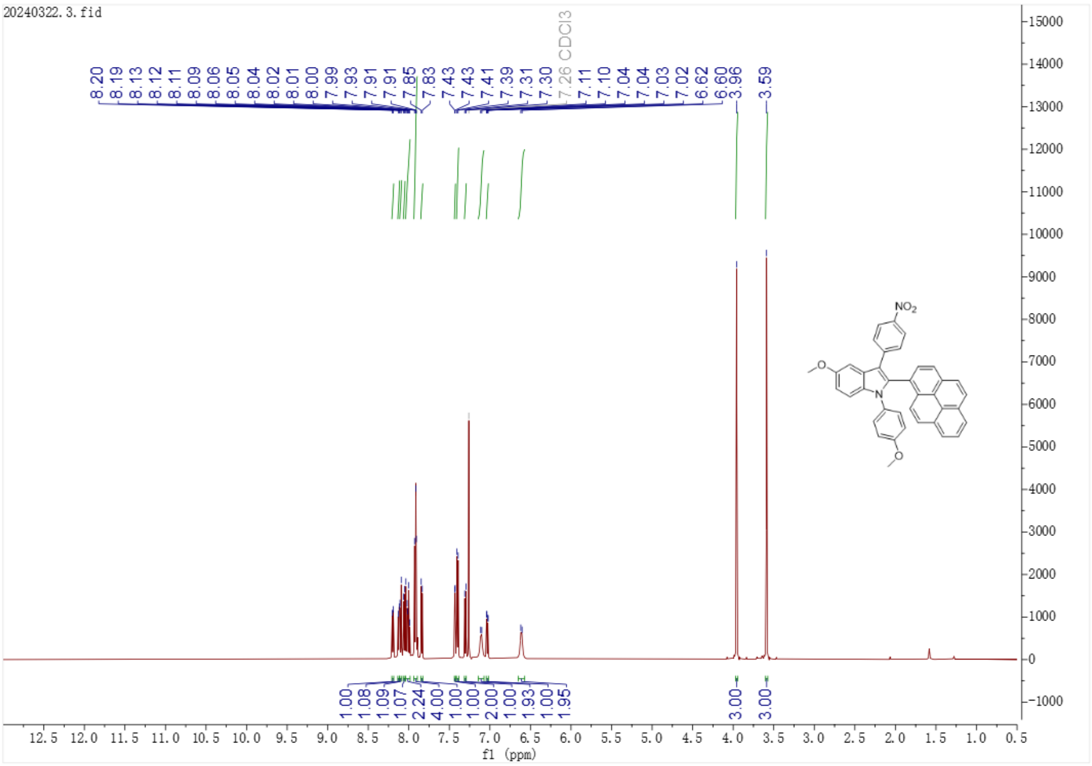

^{13}C NMR of compound **4x** (in $CDCl_3$)

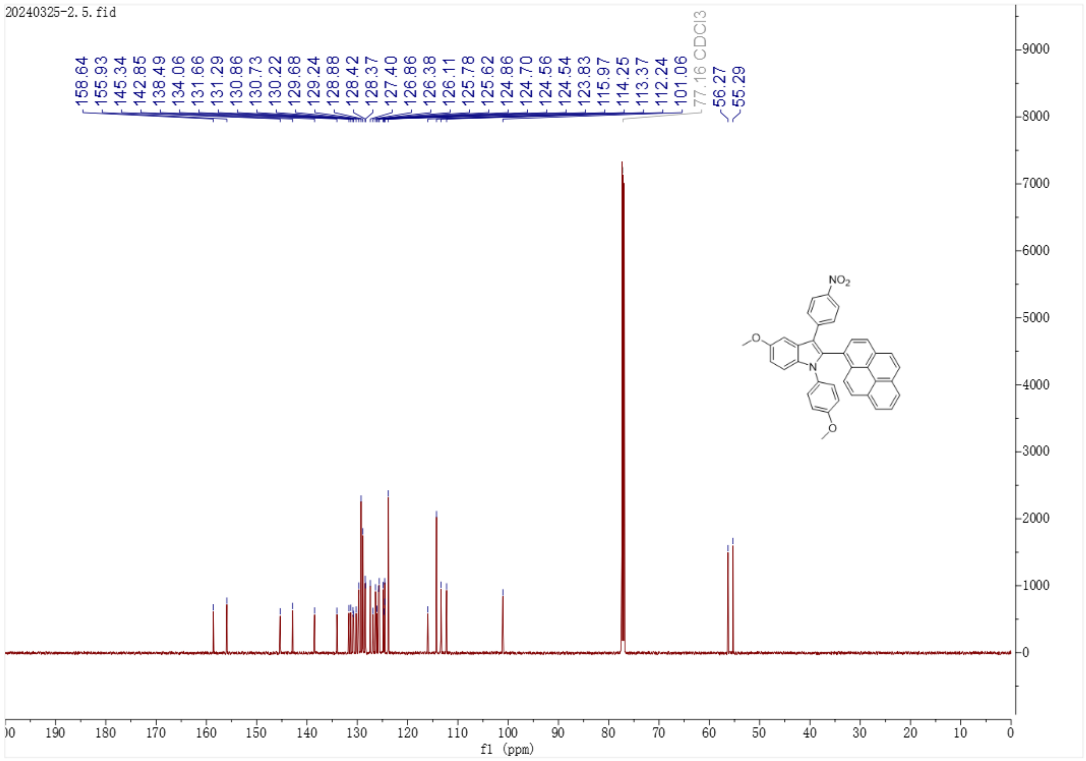

^{1}H NMR of compound **4y** (in $CDCl_3$)

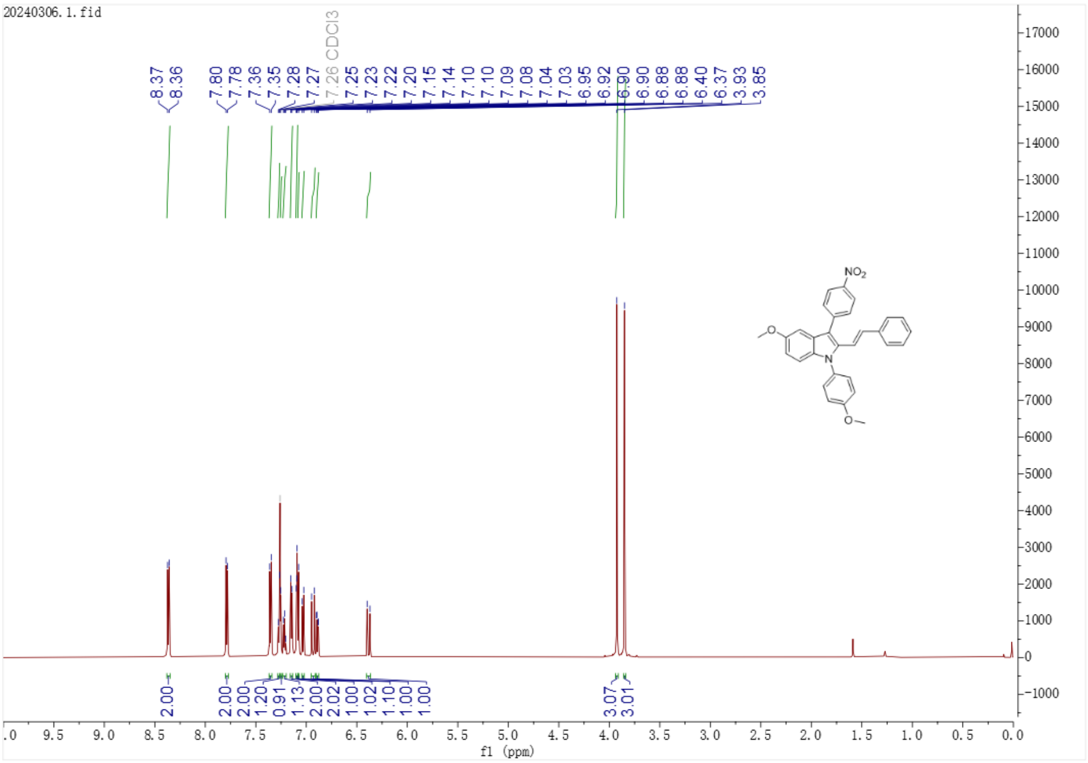

^{13}C NMR of compound **4y** (in $CDCl_3$)

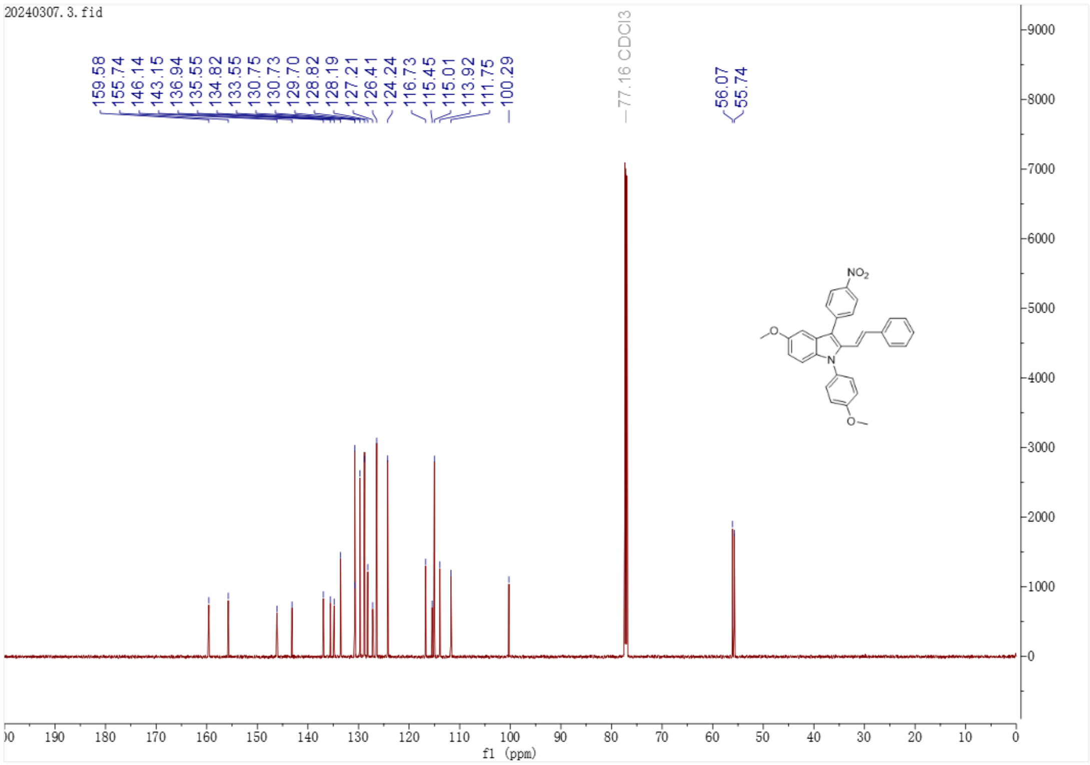

^{1}H NMR of compound **4z** (in $CDCl_3$)

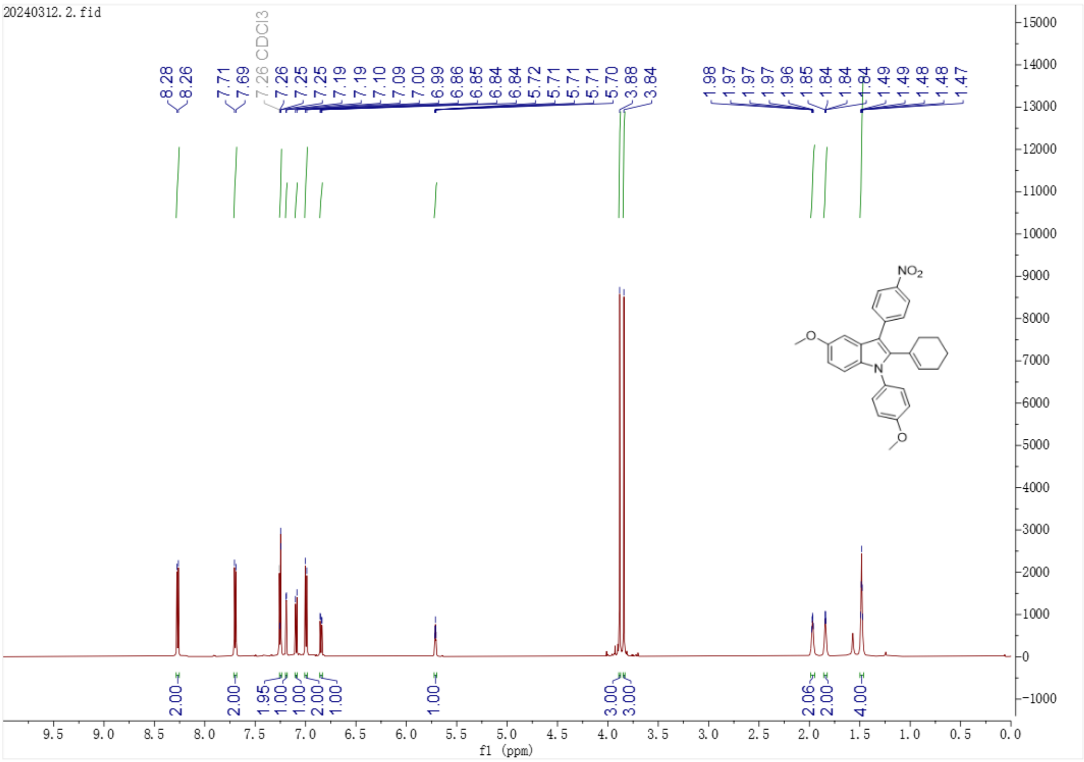

^{13}C NMR of compound **4z** (in $CDCl_3$)

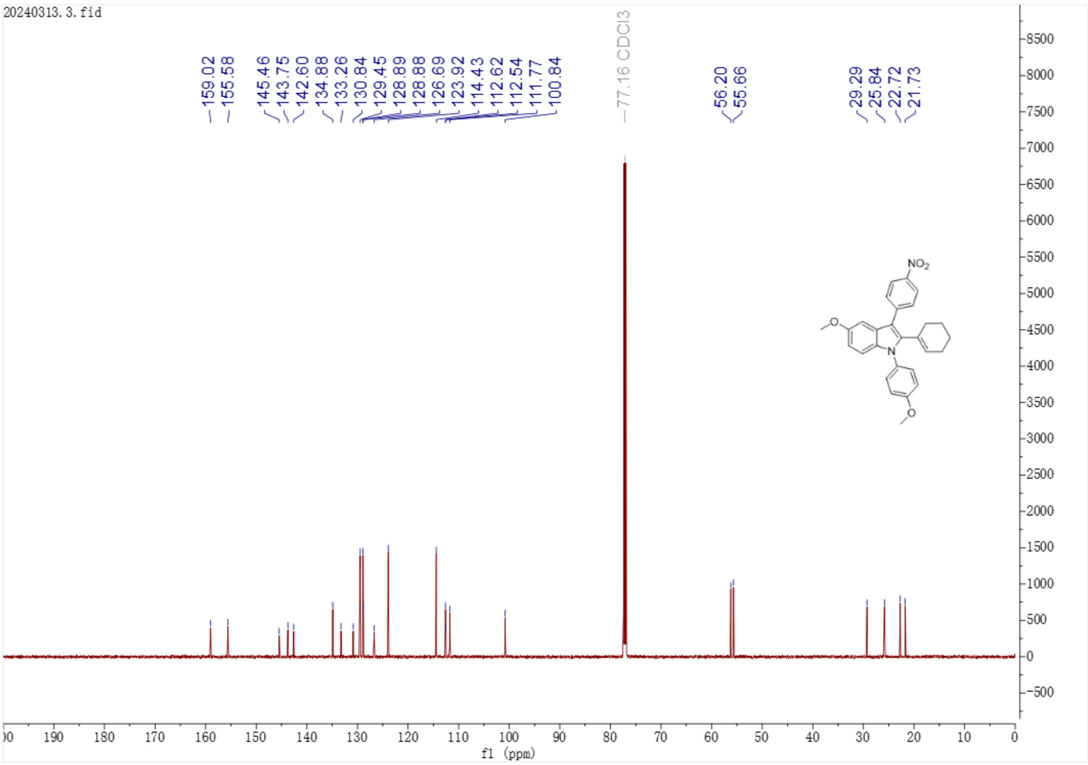

^{1}H NMR of compound **5a** (in $CDCl_3$)

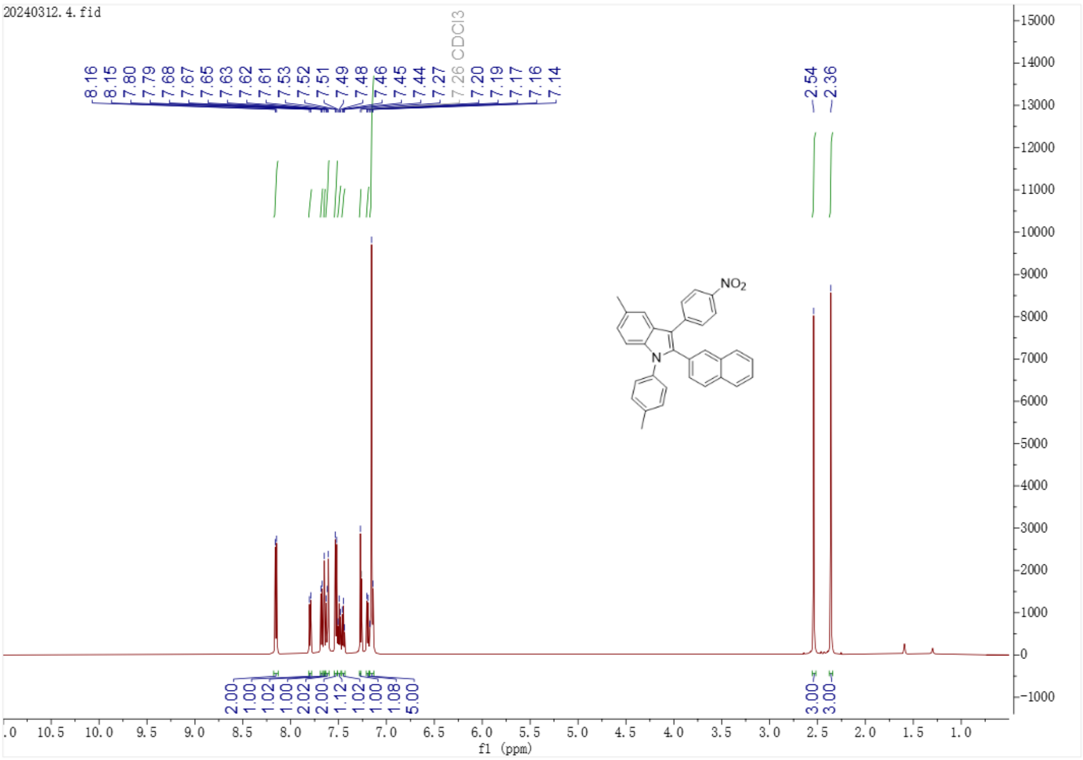

^{13}C NMR of compound **5a** (in $CDCl_3$)

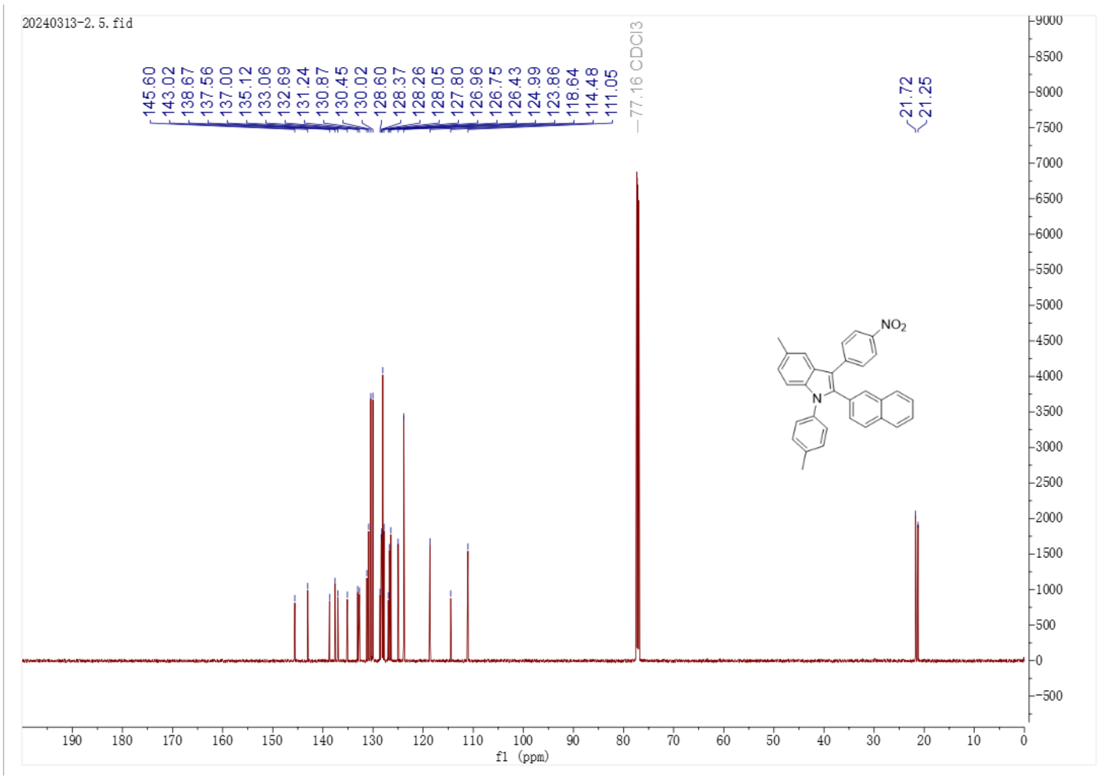

^{1}H NMR of compound **5b** (in $CDCl_3$)

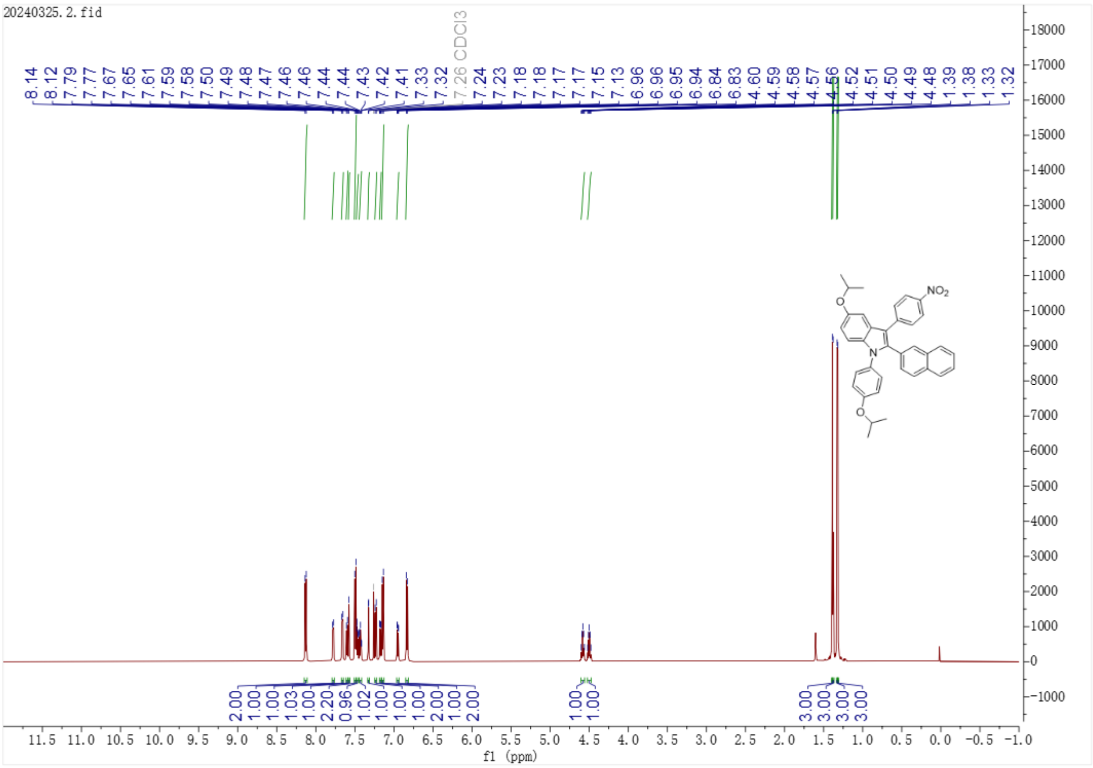

^{13}C NMR of compound **5b** (in $CDCl_3$)

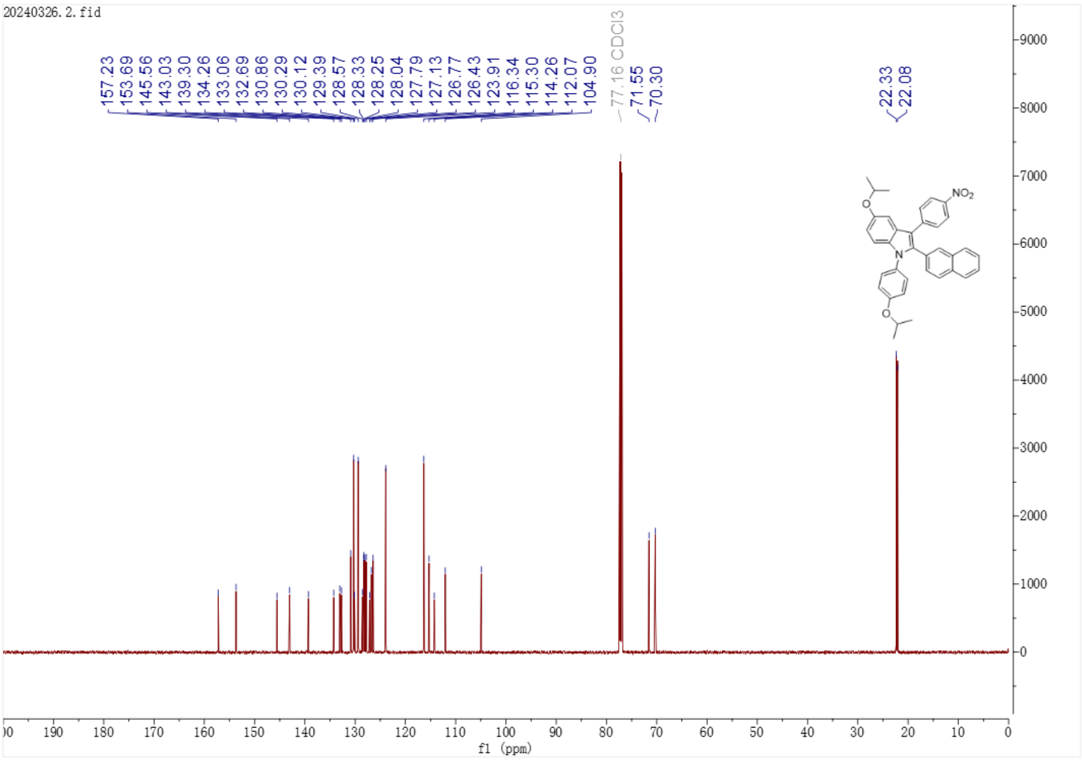

^{1}H NMR of compound **5c** (in $CDCl_3$)

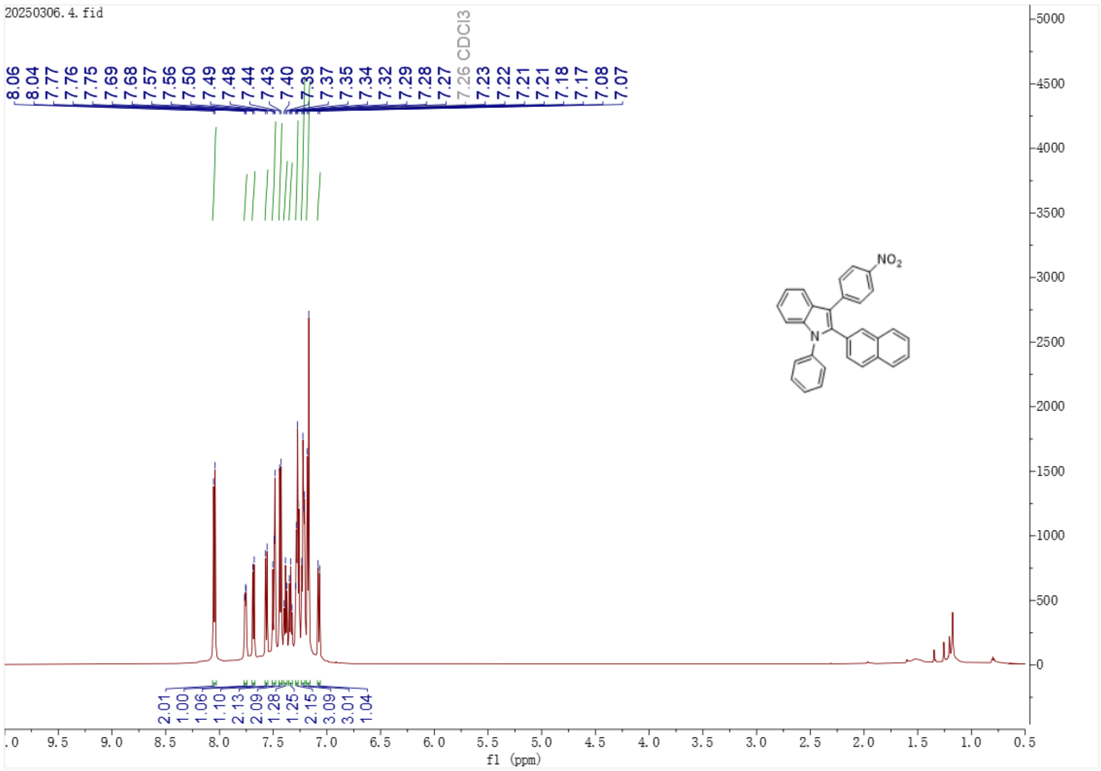

^{13}C NMR of compound **5c** (in $CDCl_3$)

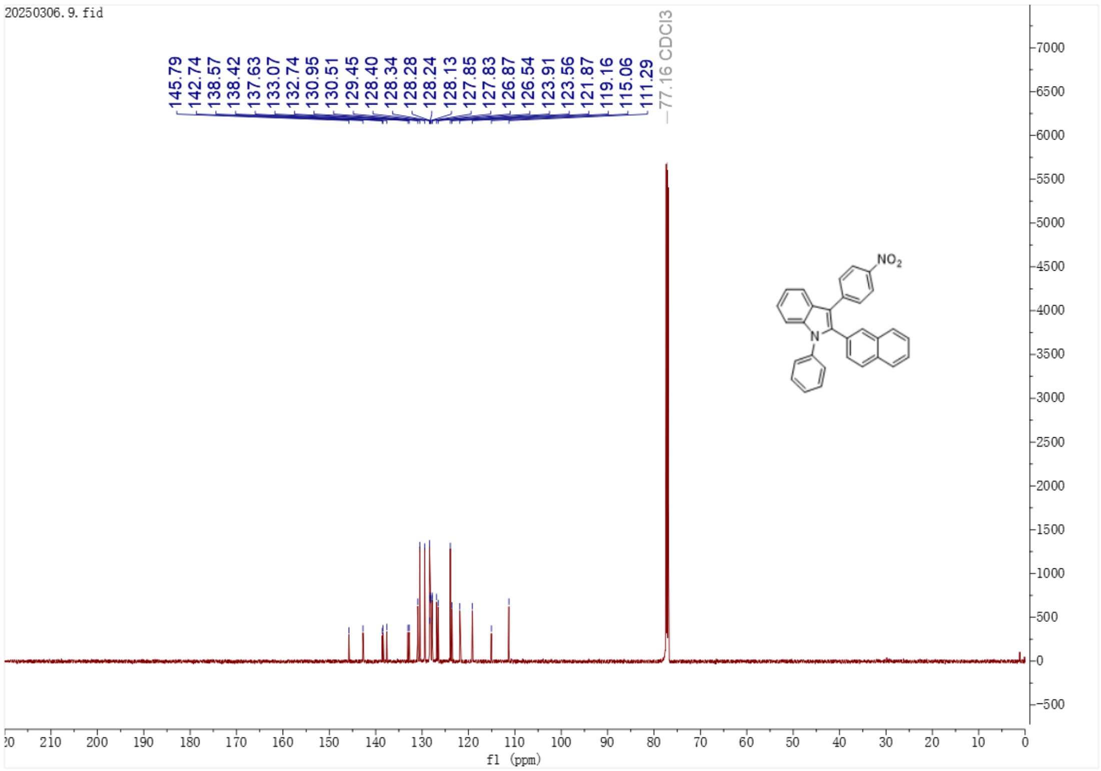

^{1}H NMR of compound **5d** (in $CDCl_3$)

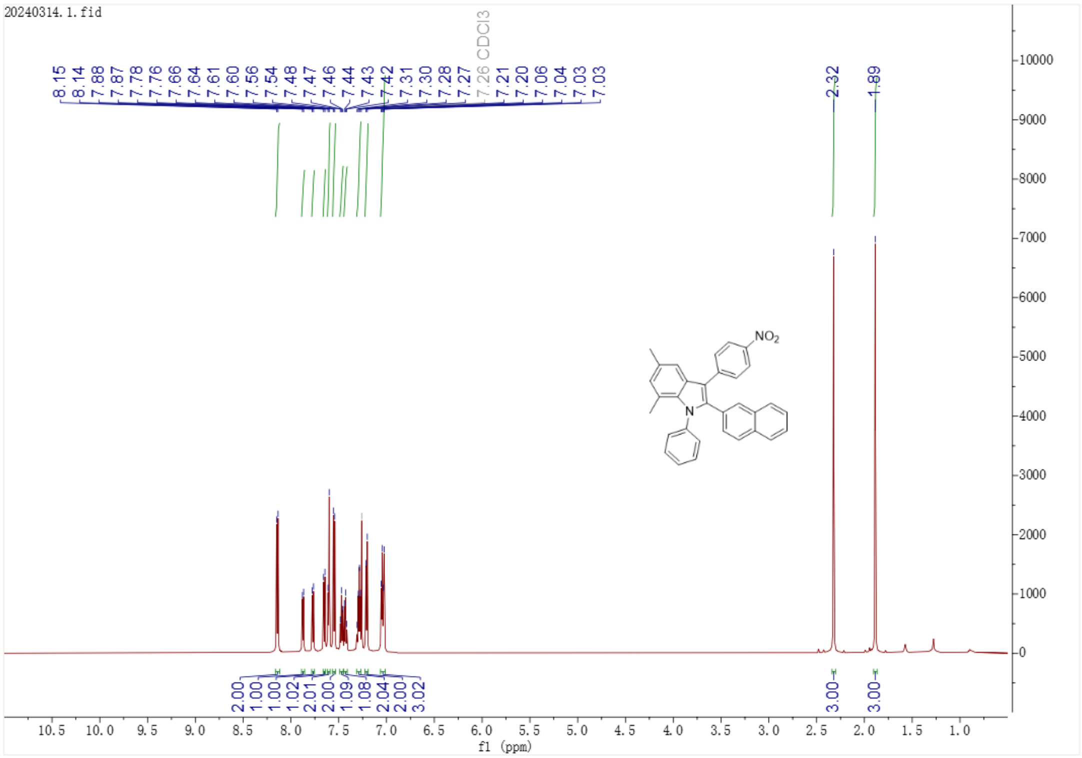

^{13}C NMR of compound **5d** (in $CDCl_3$)

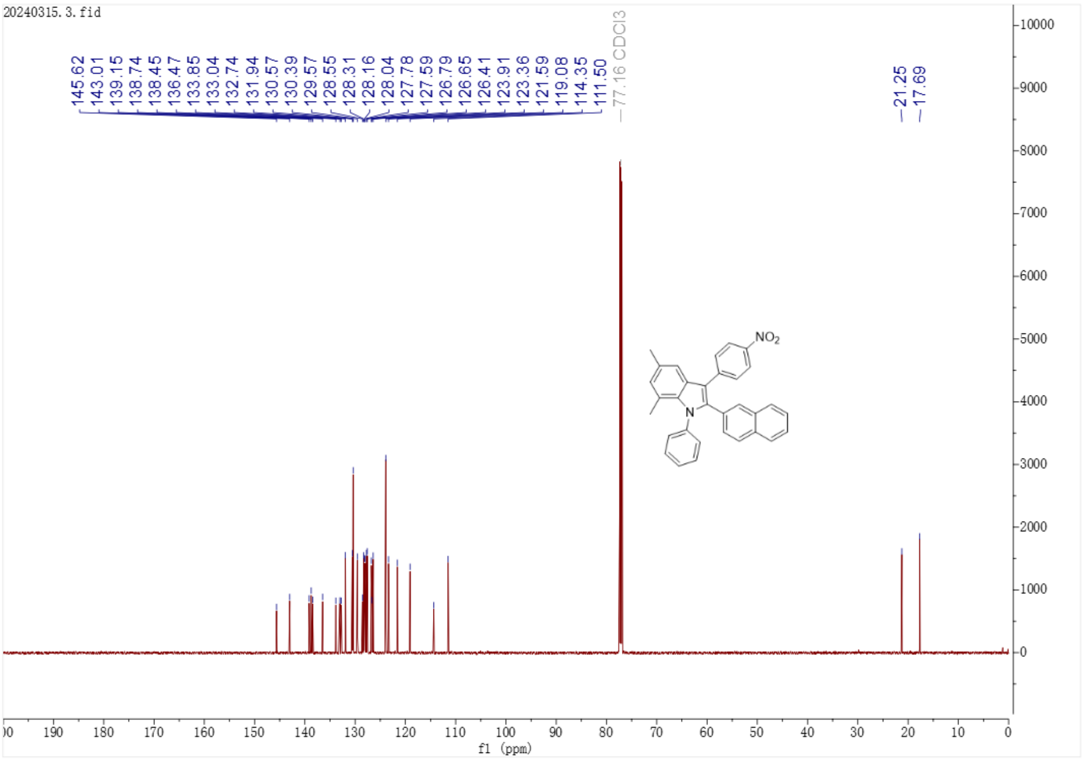

^{1}H NMR of compound **5e** (in $CDCl_3$)

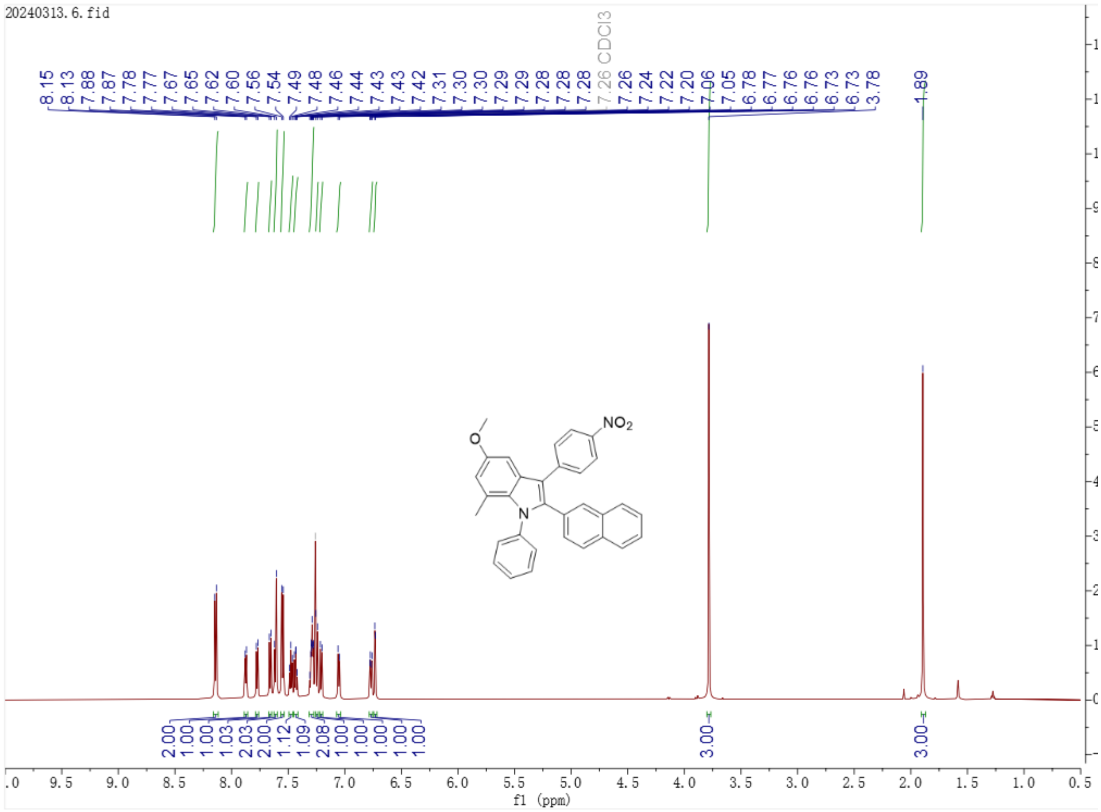

^{13}C NMR of compound **5d** (in $CDCl_3$)

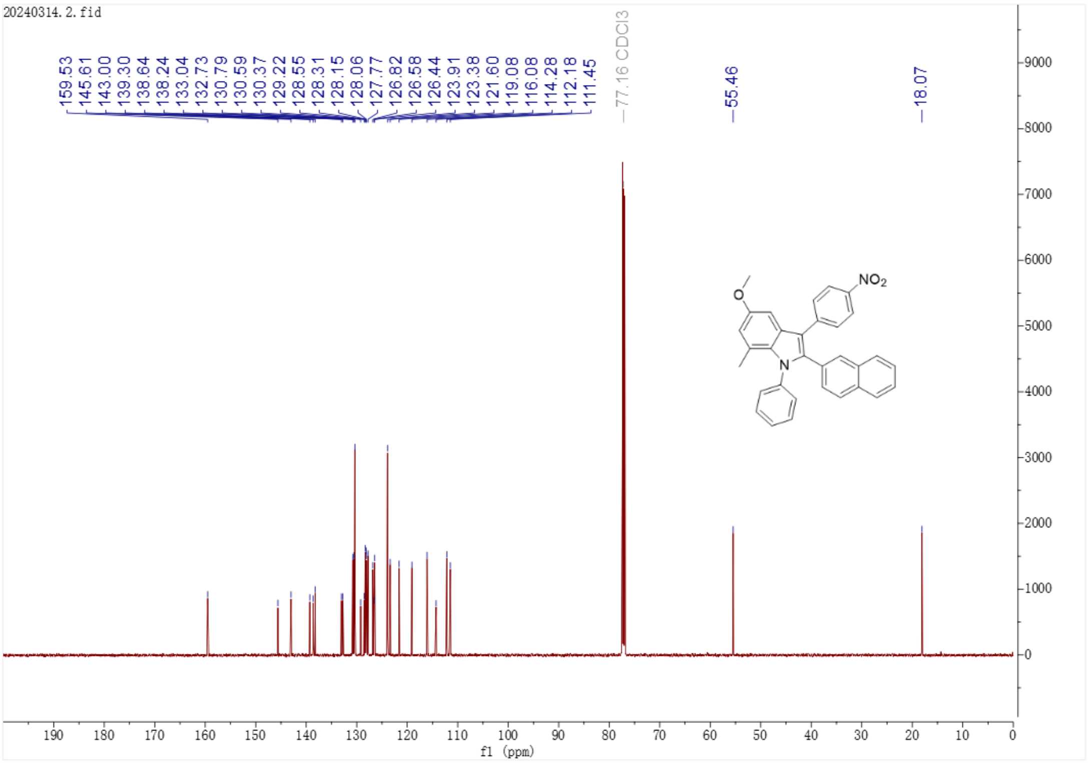

^{1}H NMR of compound **5f** (in $CDCl_3$)

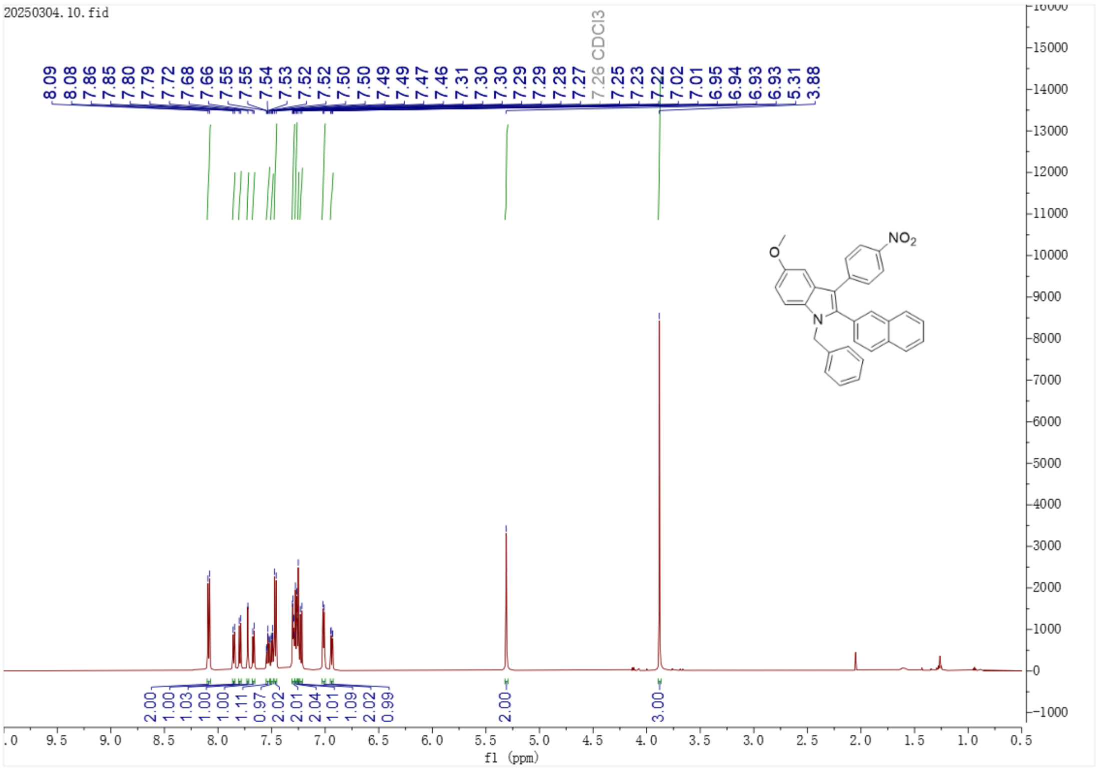

^{13}C NMR of compound **5f** (in $CDCl_3$)

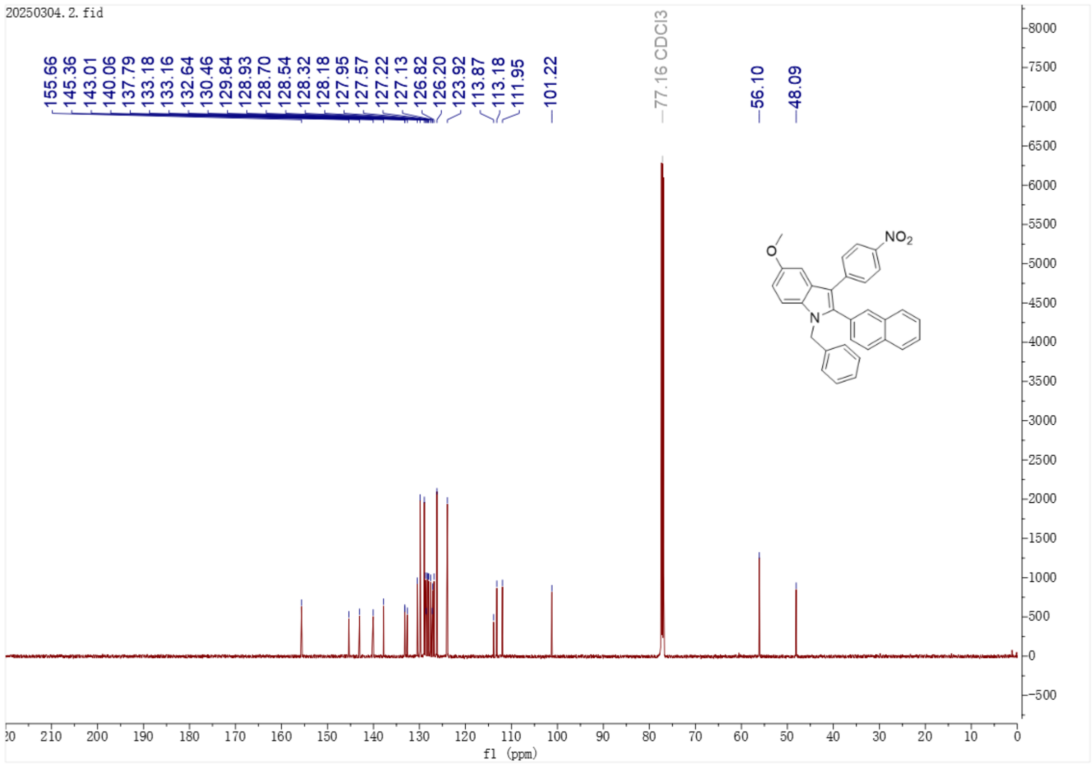

^{1}H NMR of compound **5g** (in $CDCl_3$)

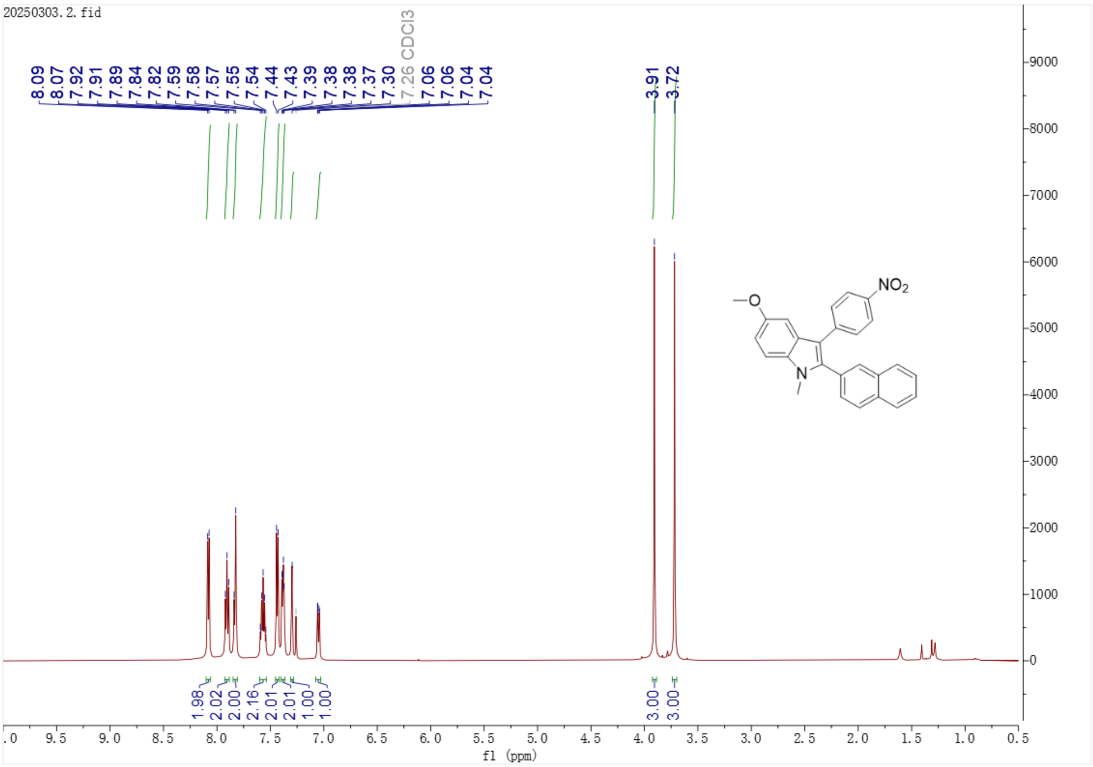

^{13}C NMR of compound **5g** (in $CDCl_3$)

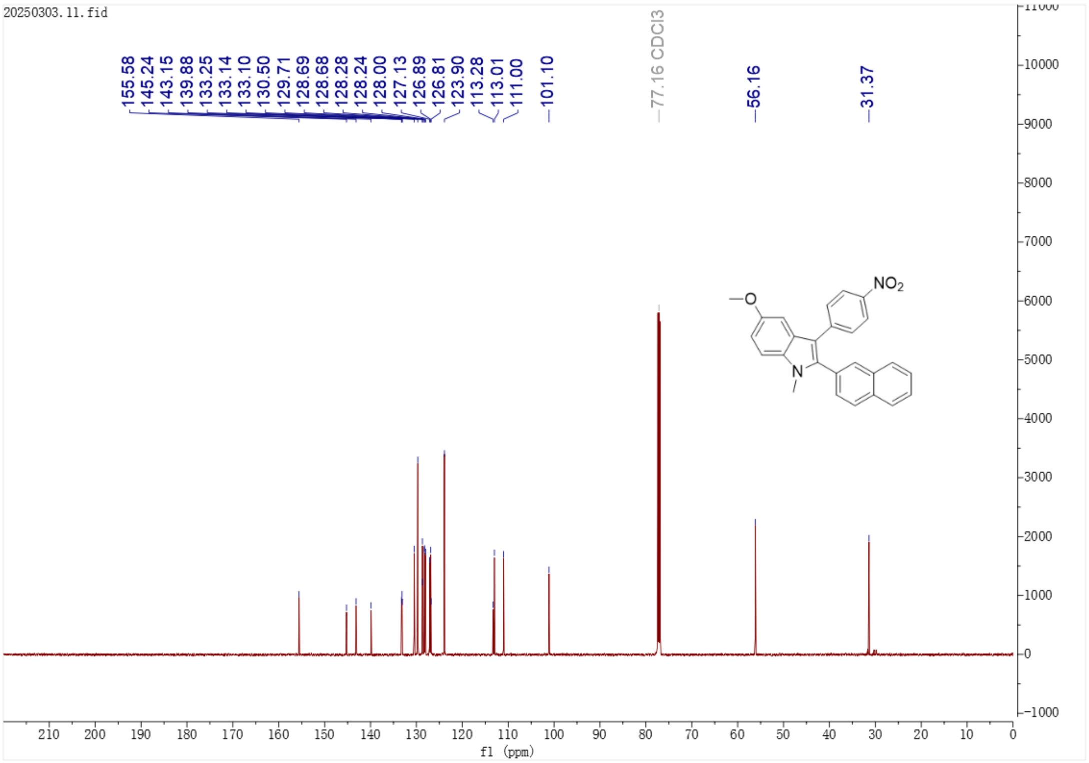

^{1}H NMR of compound **5h** (in $CDCl_3$)

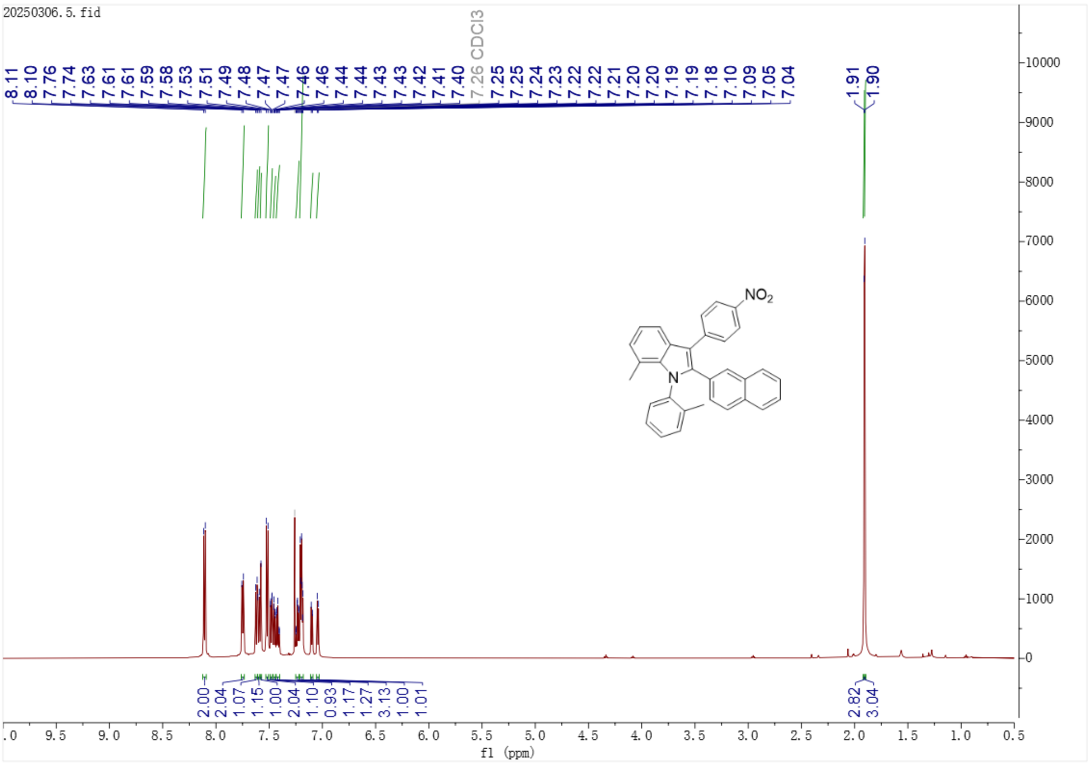

^{13}C NMR of compound **5h** (in $CDCl_3$)

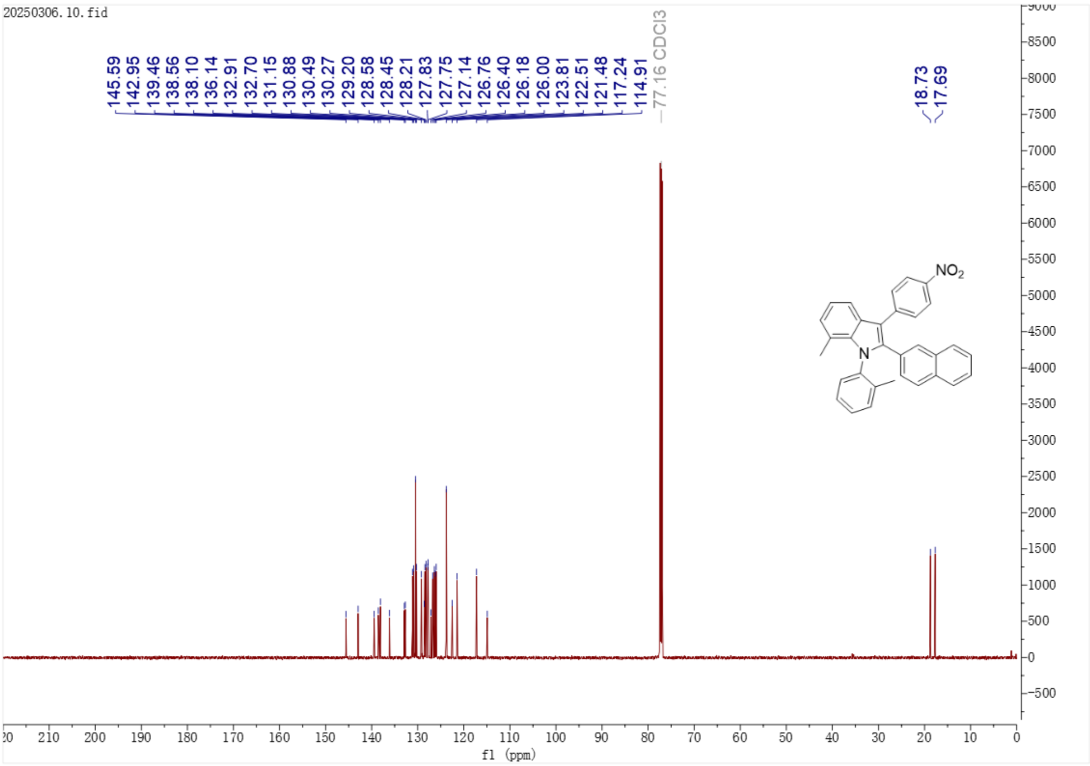

^{1}H NMR of compound **5i** (in $CDCl_3$)

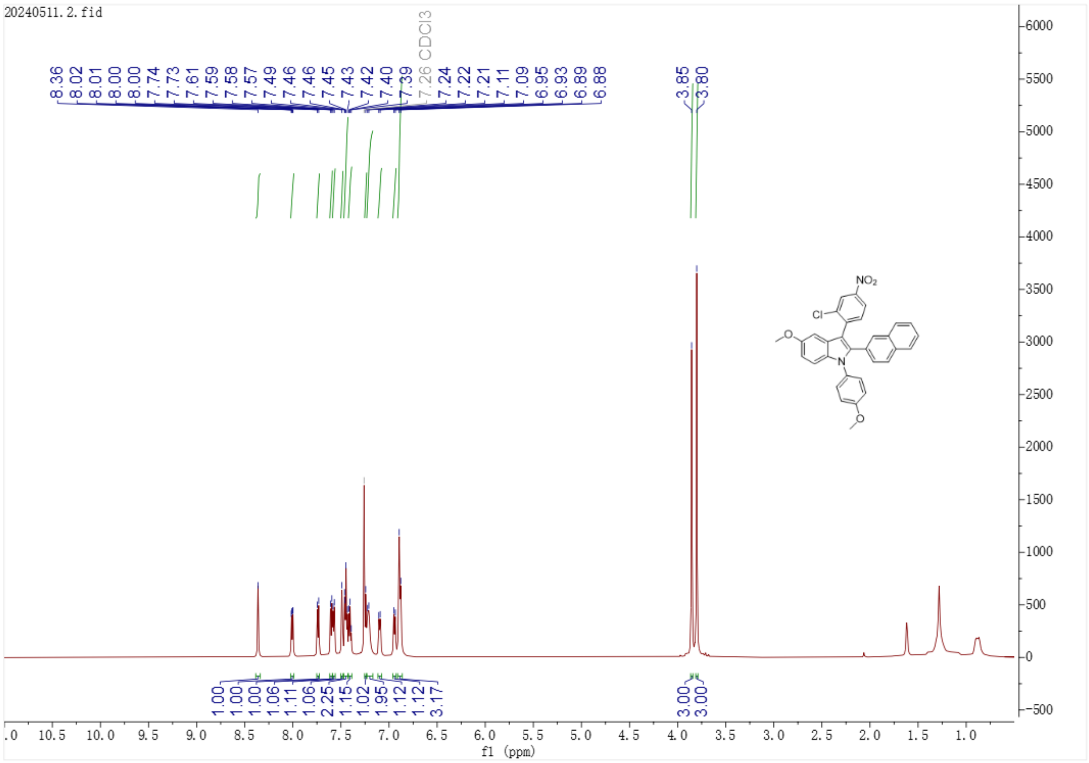

^{13}C NMR of compound **5i** (in $CDCl_3$)

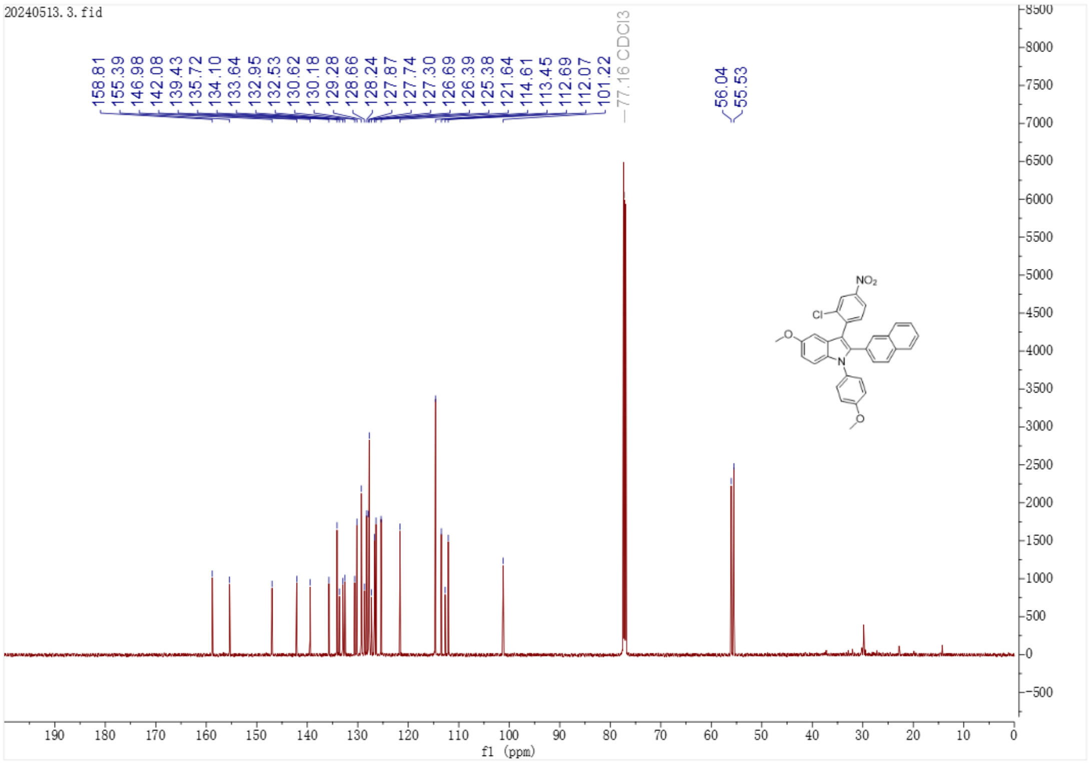

^{1}H NMR of compound **5j** (in $CDCl_3$)

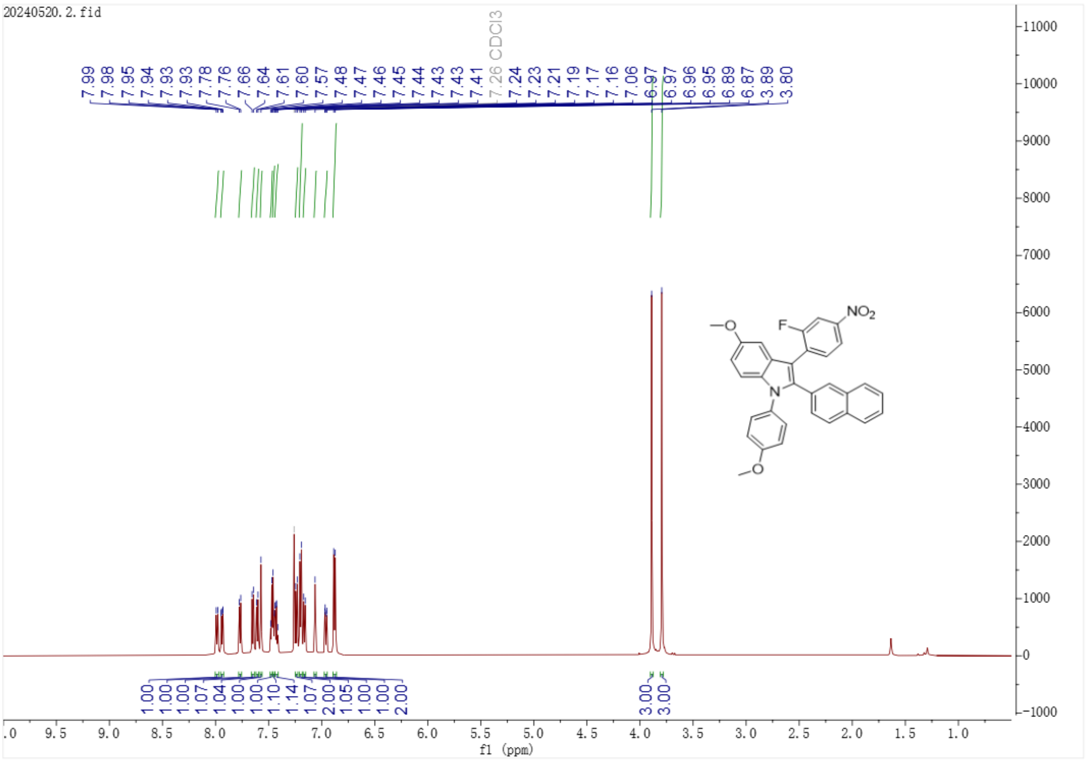

^{13}C NMR of compound **5j** (in $CDCl_3$)

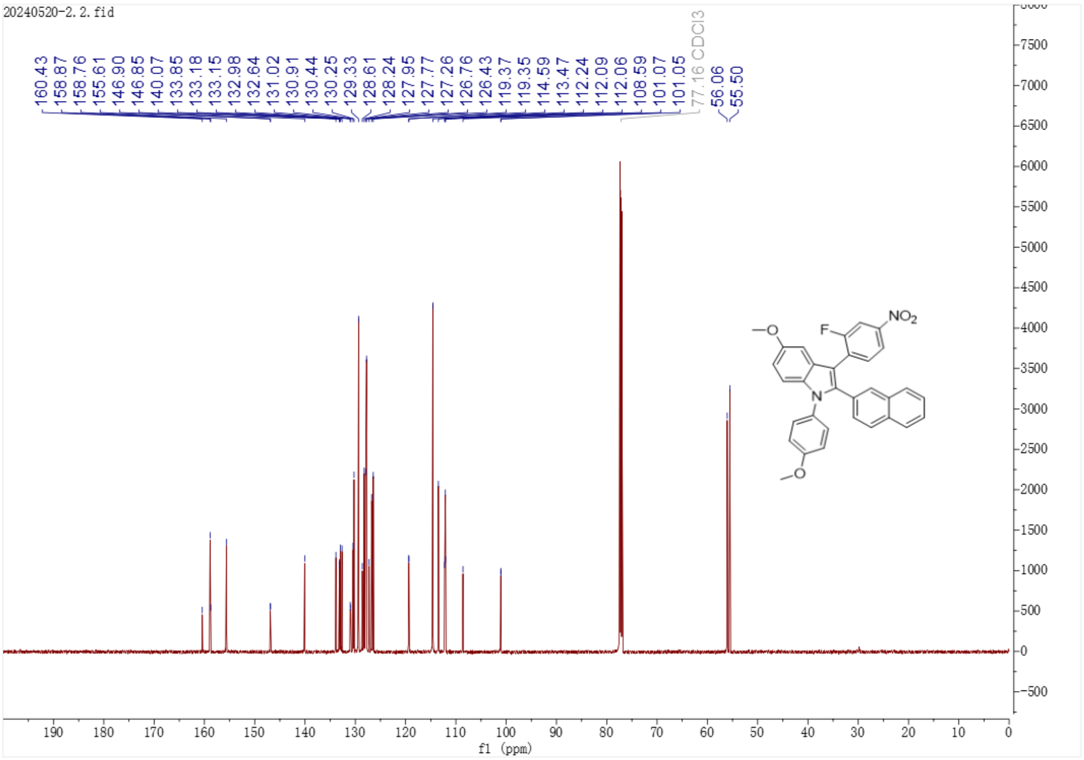

^{19}F NMR of compound **5j** (in $CDCl_3$)

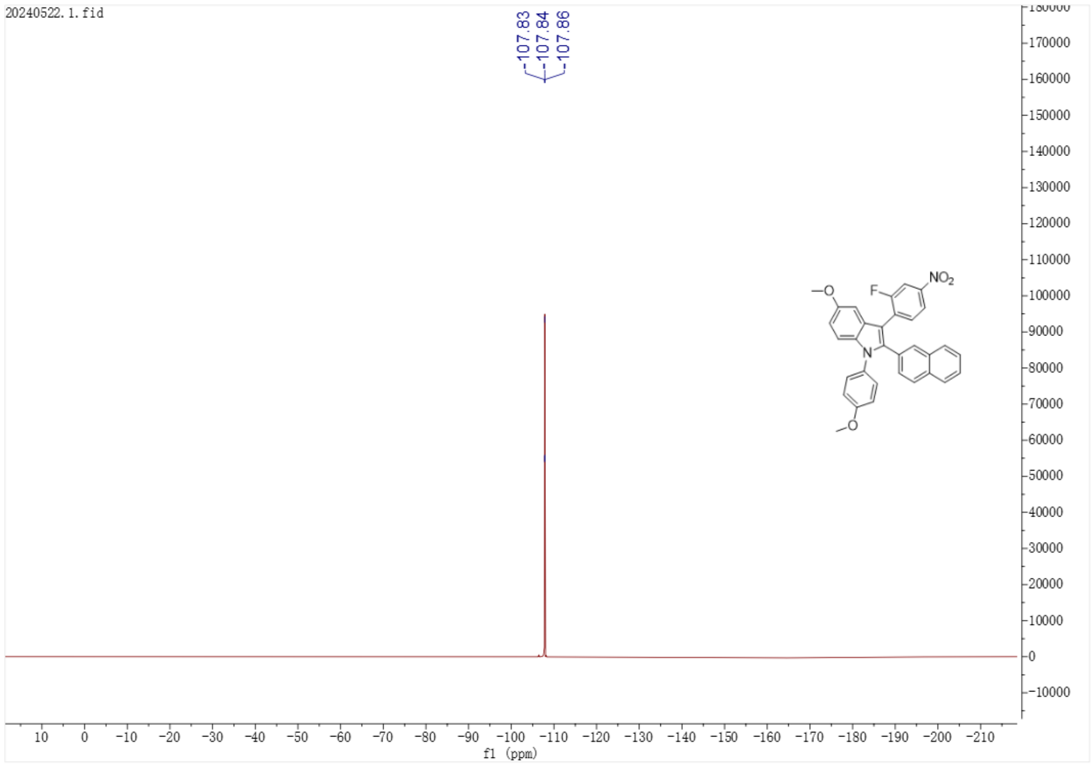

^{1}H NMR of compound **5k** (in $CDCl_3$)

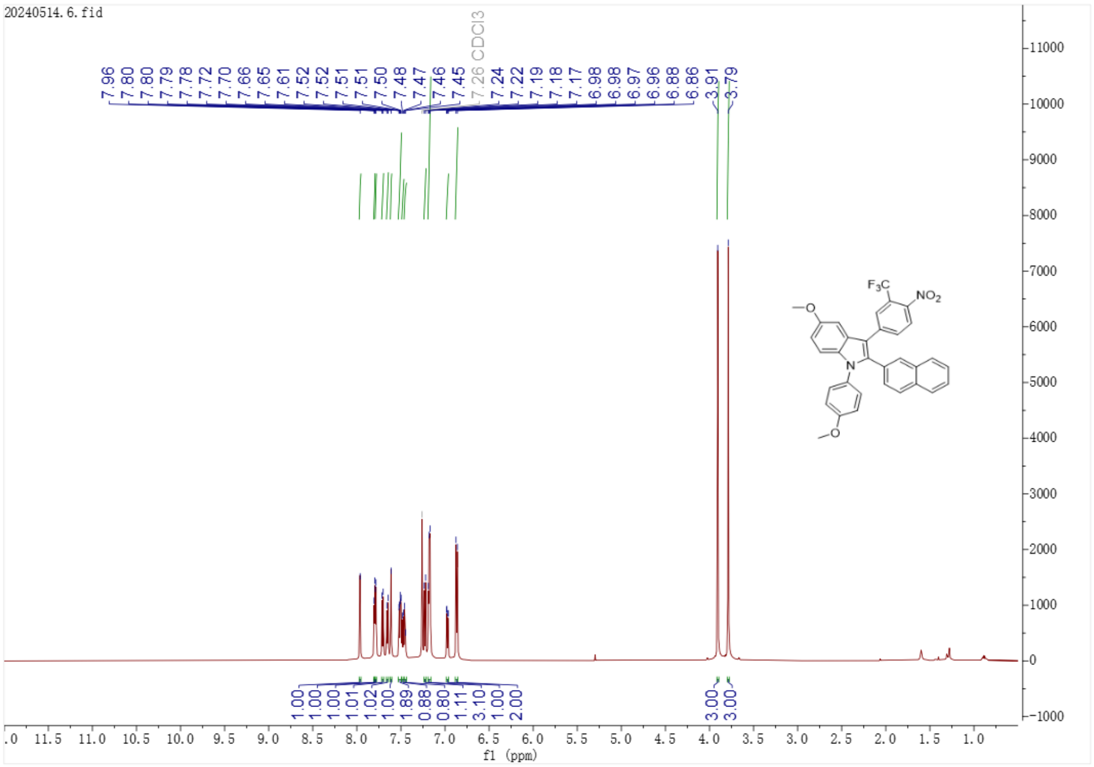

^{13}C NMR of compound **5k** (in $CDCl_3$)

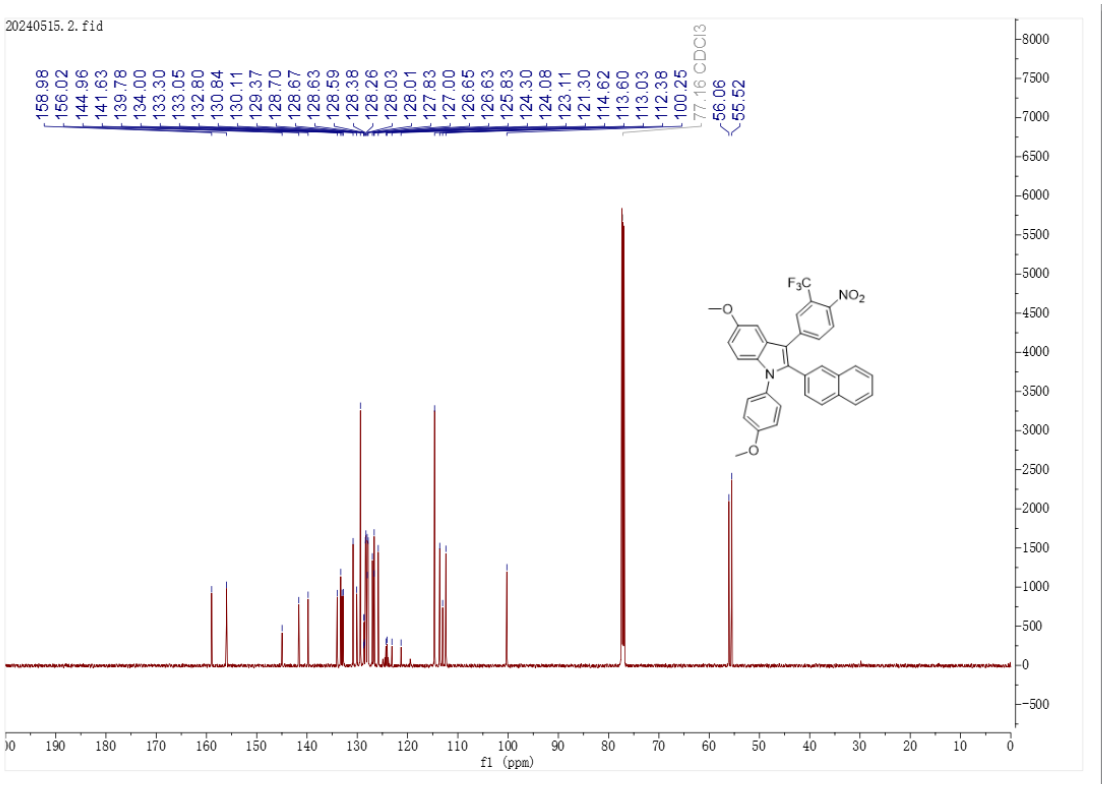

^{19}F NMR of compound **5k** (in $CDCl_3$)

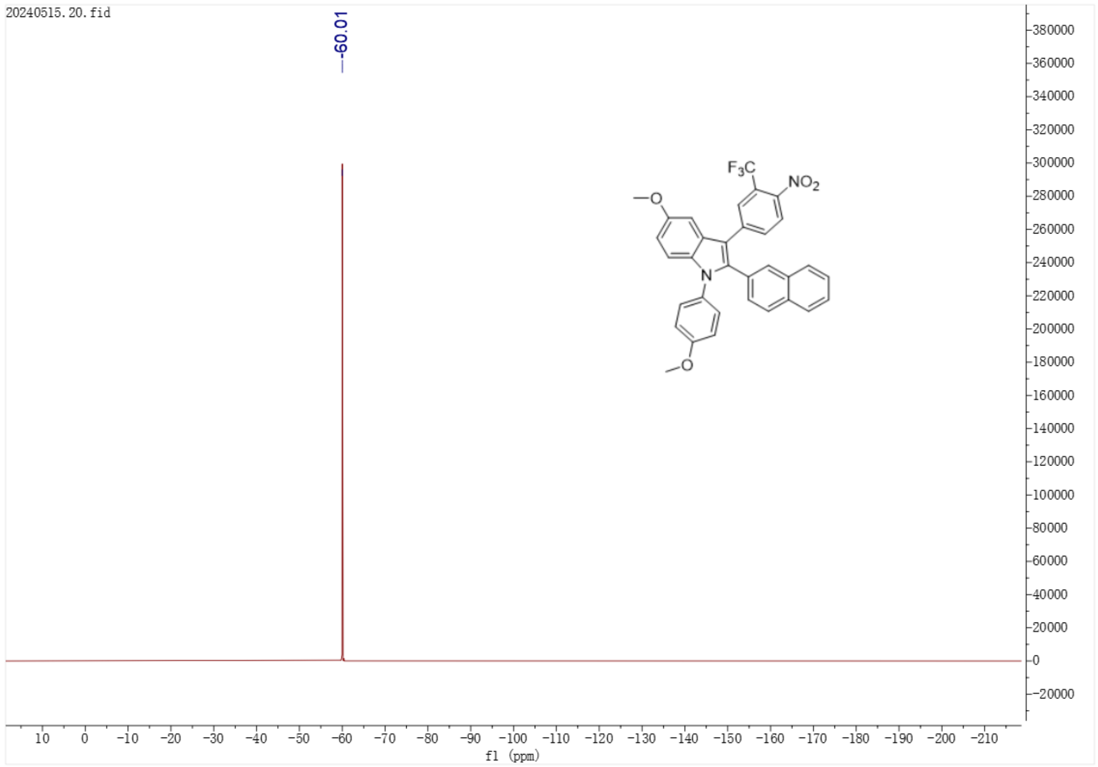

^{1}H NMR of compound **5l** (in $CDCl_3$)

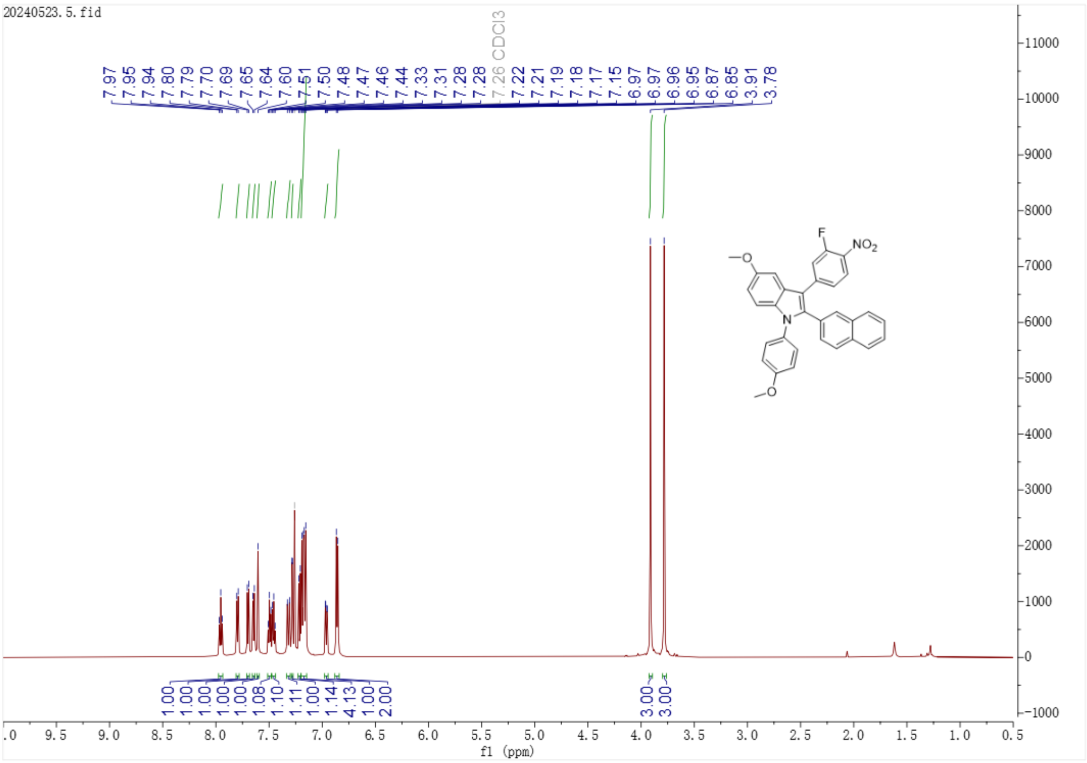

^{13}C NMR of compound **5l** (in $CDCl_3$)

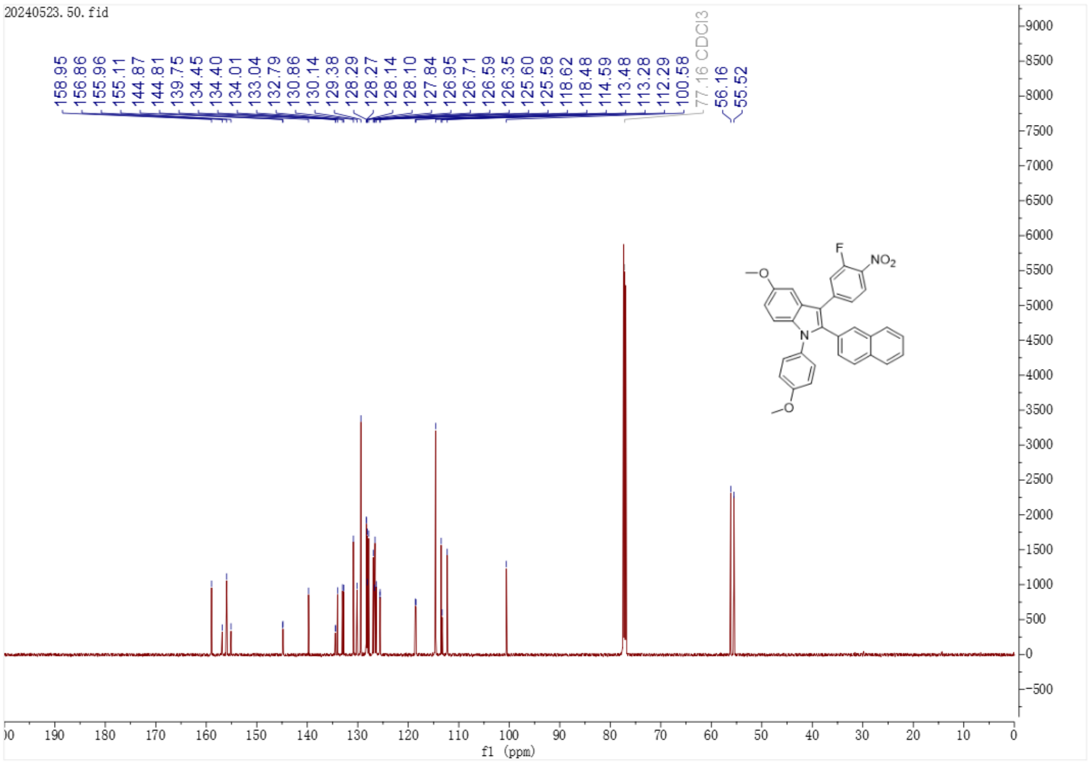

^{19}F NMR of compound **5l** (in $CDCl_3$)

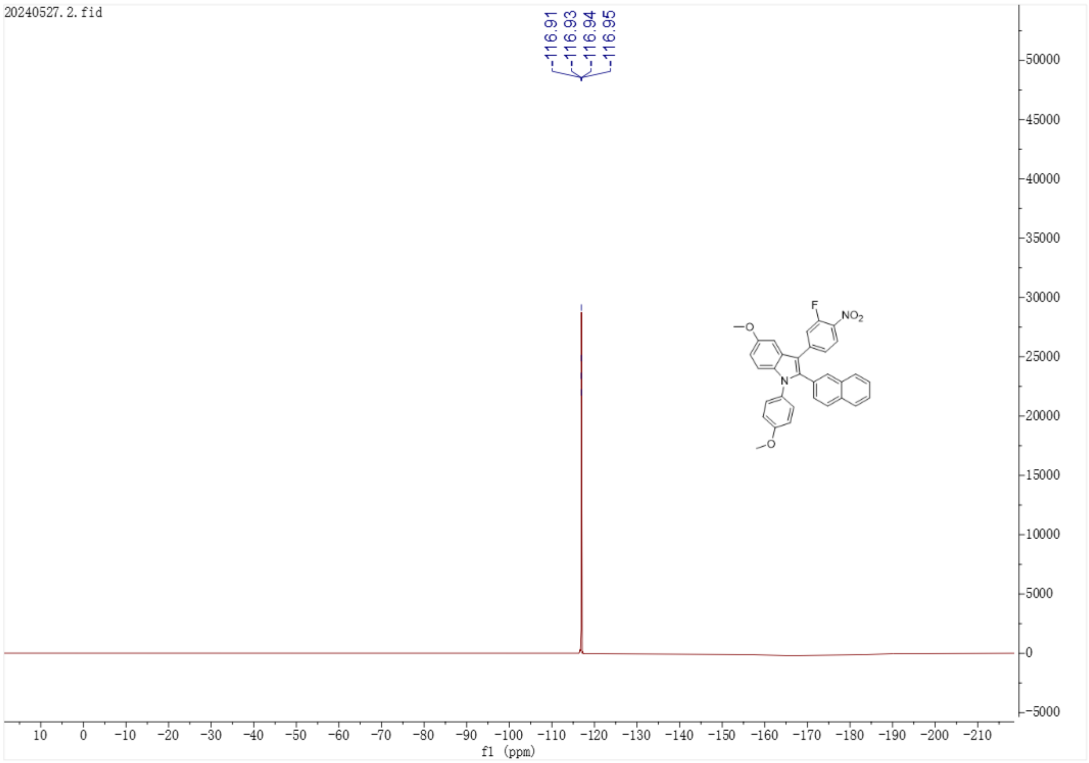

^{1}H NMR of compound **5m** (in $CDCl_3$)

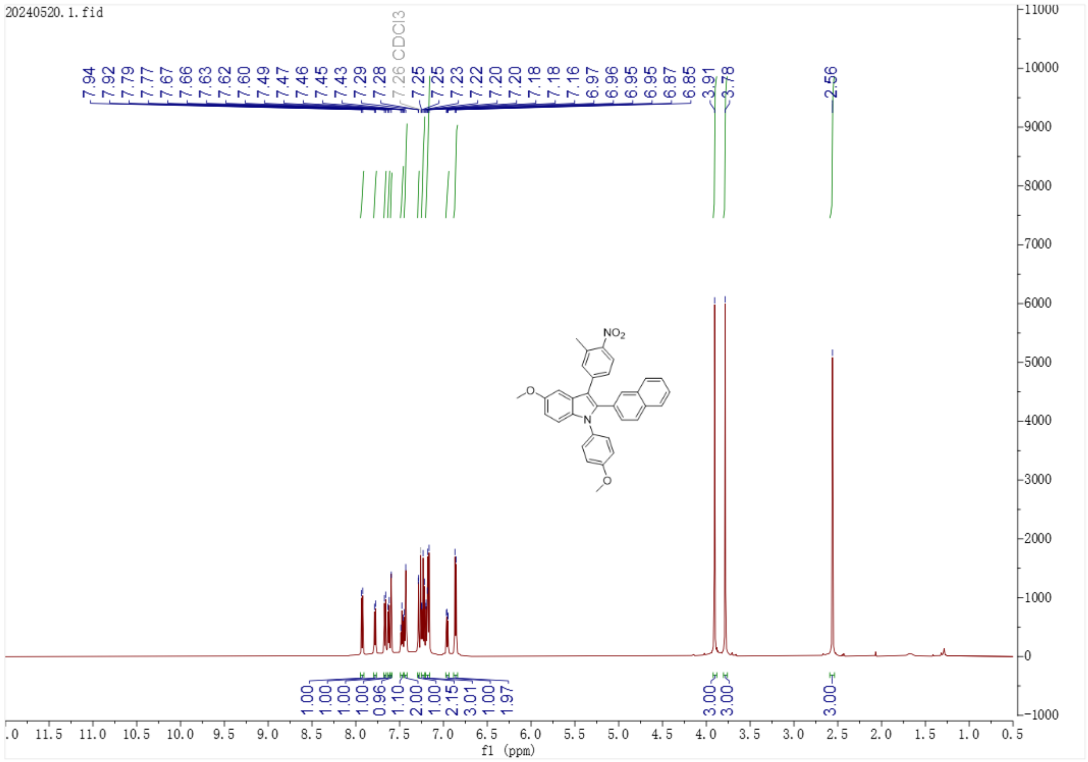

^{13}C NMR of compound **5m** (in $CDCl_3$)

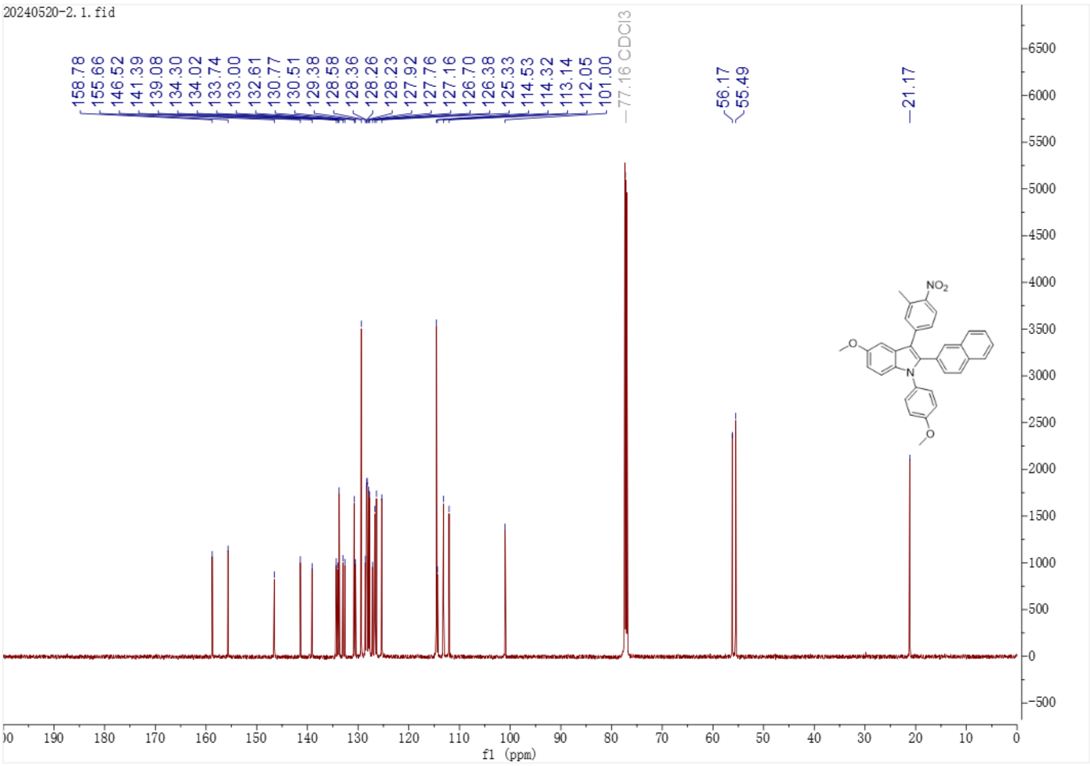

^{1}H NMR of compound **6** (in $CDCl_3$)

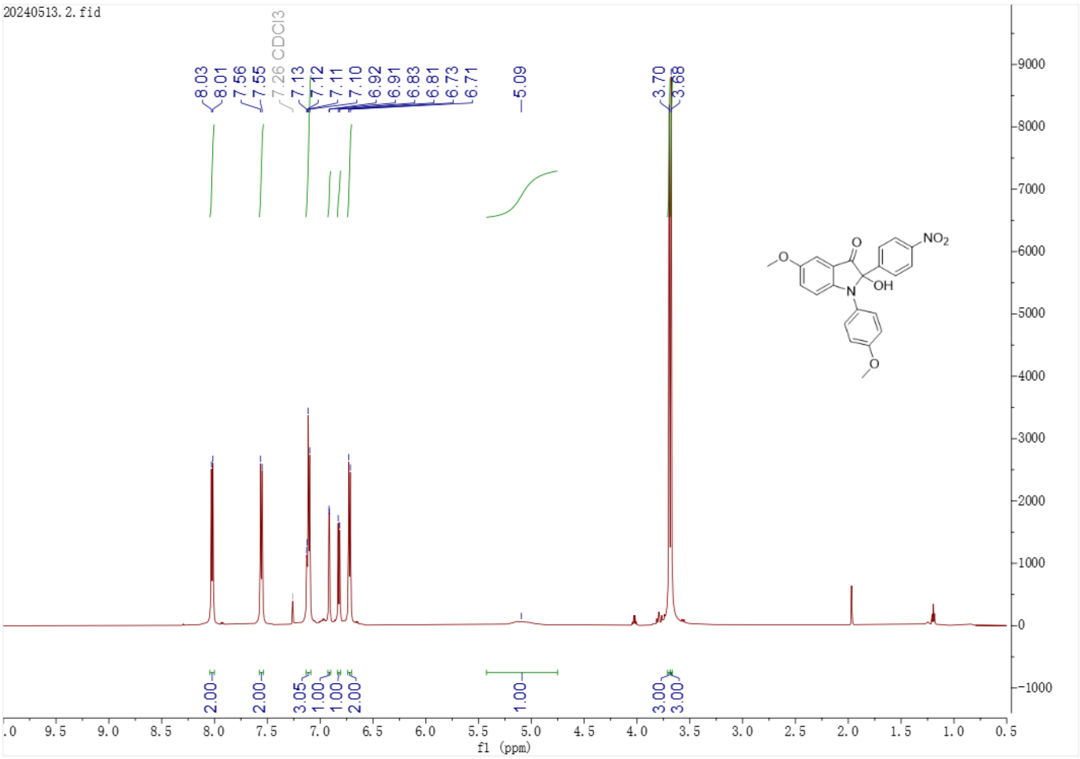

^{13}C NMR of compound **6** (in $CDCl_3$)

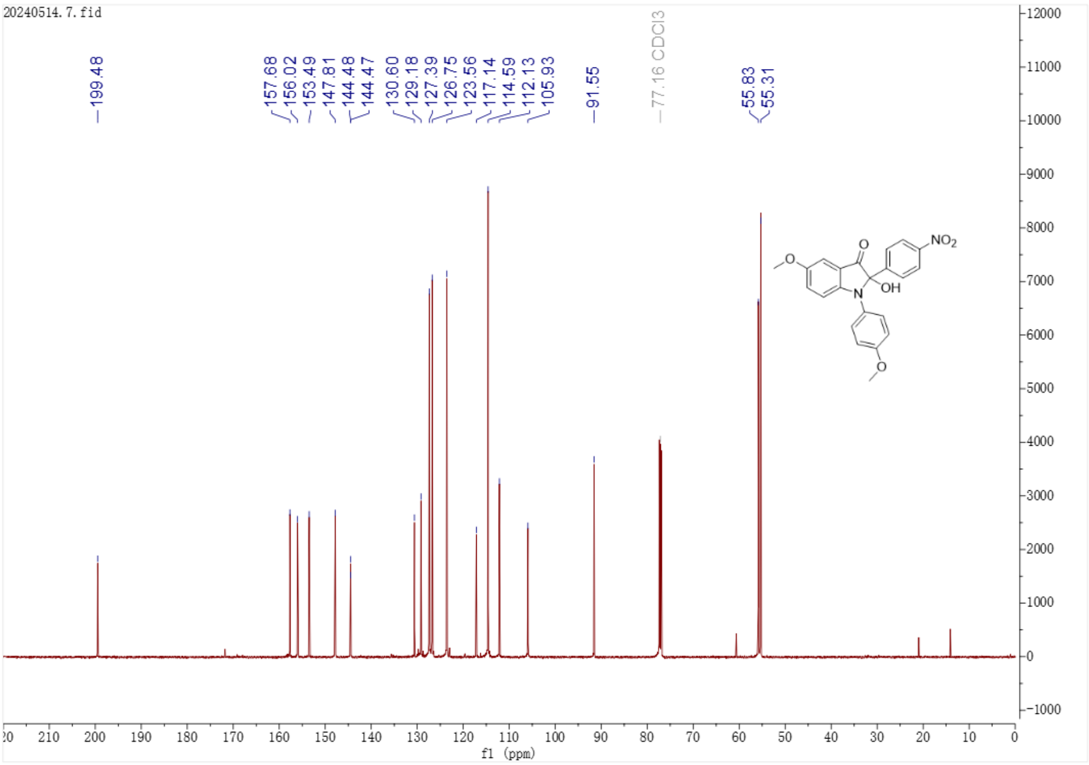

^{1}H NMR of compound **7** (in DMSO-d_6)

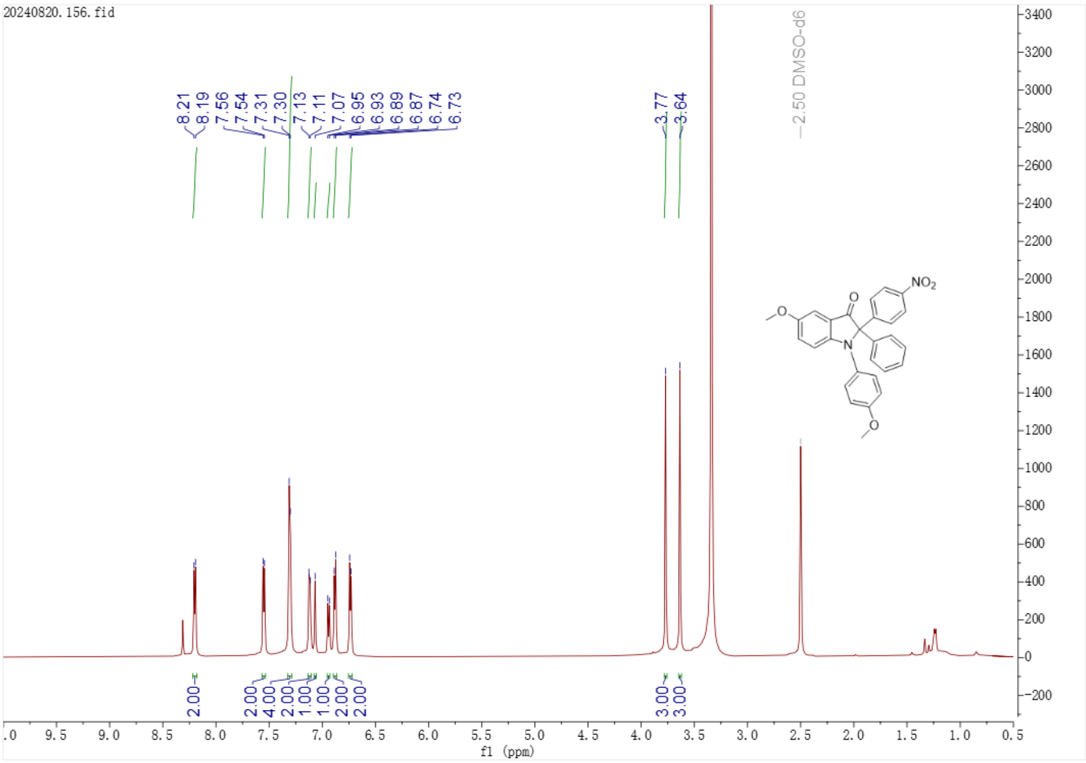

^{13}C NMR of compound **7** (in $CDCl_3$)

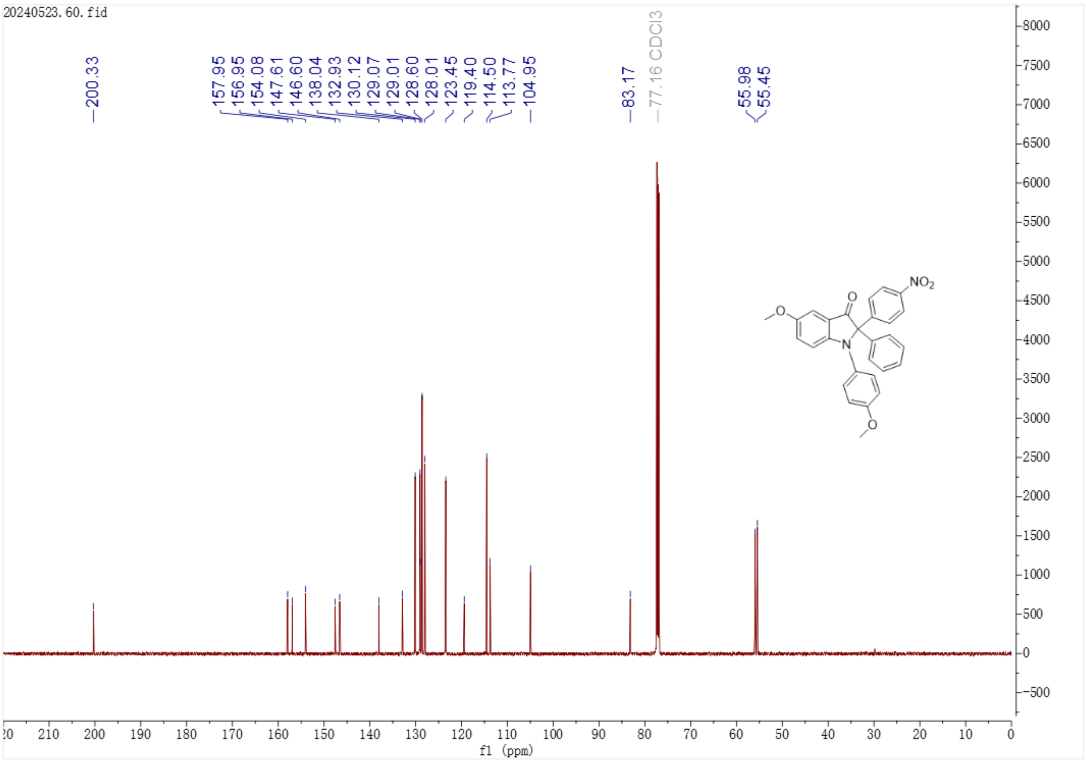

^{1}H NMR of compound **8a** (in $CDCl_3$)

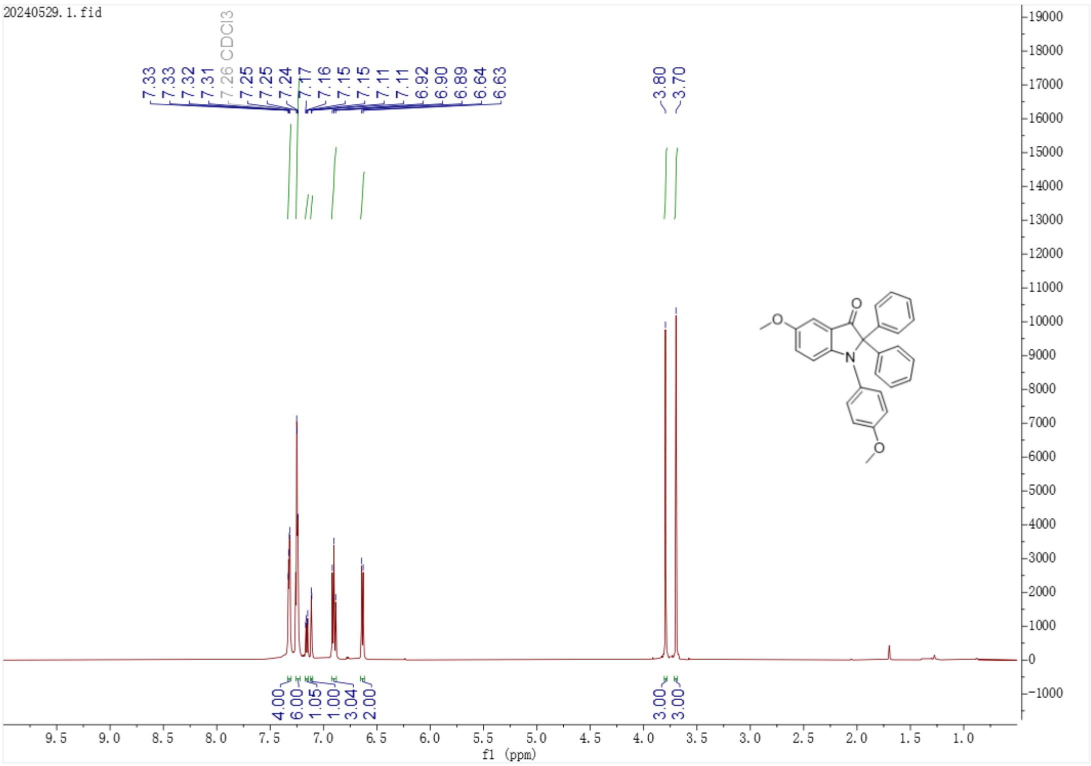

^{13}C NMR of compound **8a** (in $CDCl_3$)

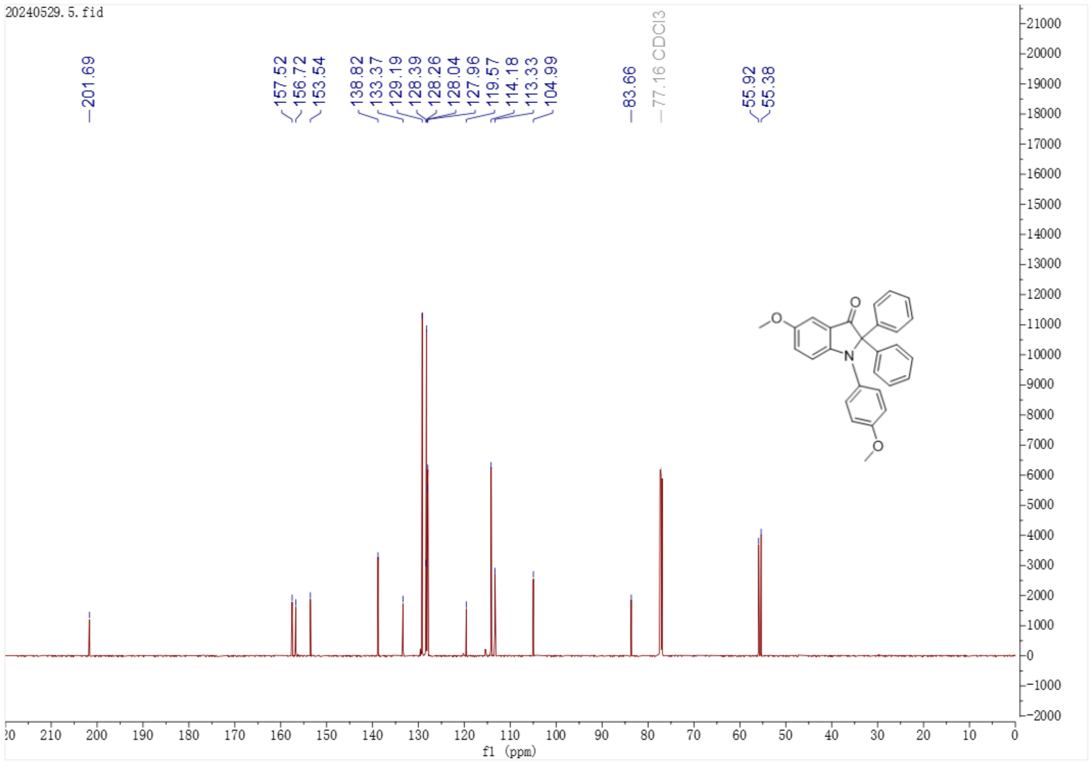

^{1}H NMR of compound **8b** (in $CDCl_3$)

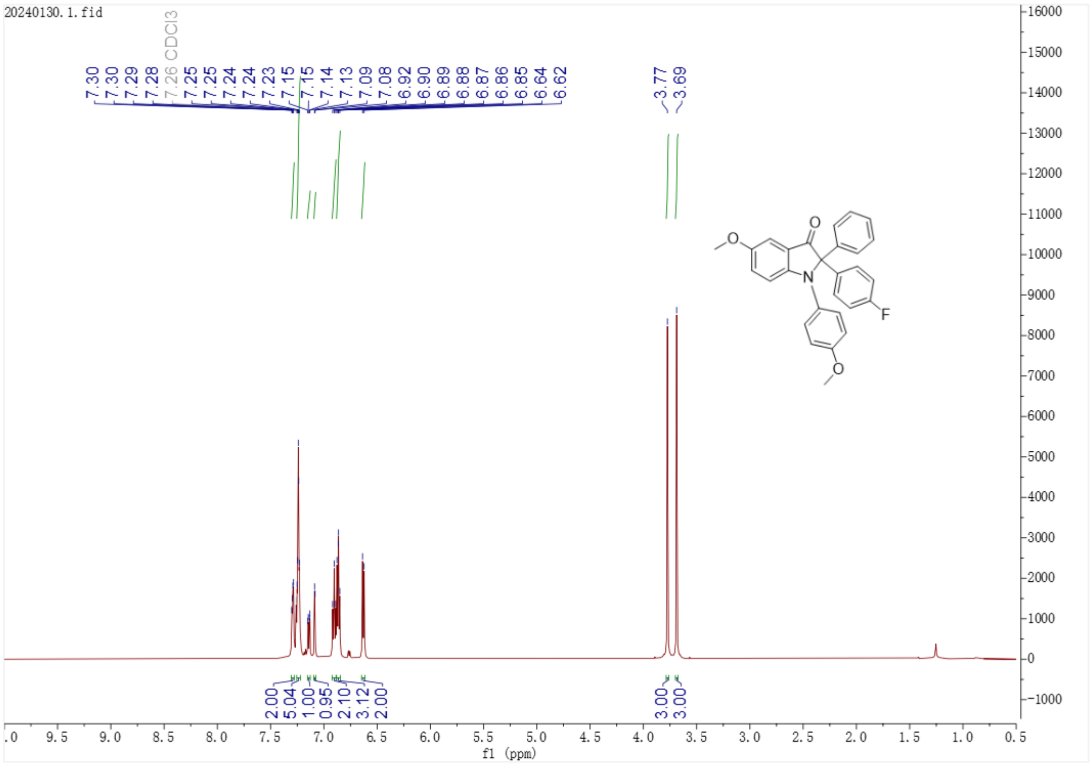

^{13}C NMR of compound **8b** (in $CDCl_3$)

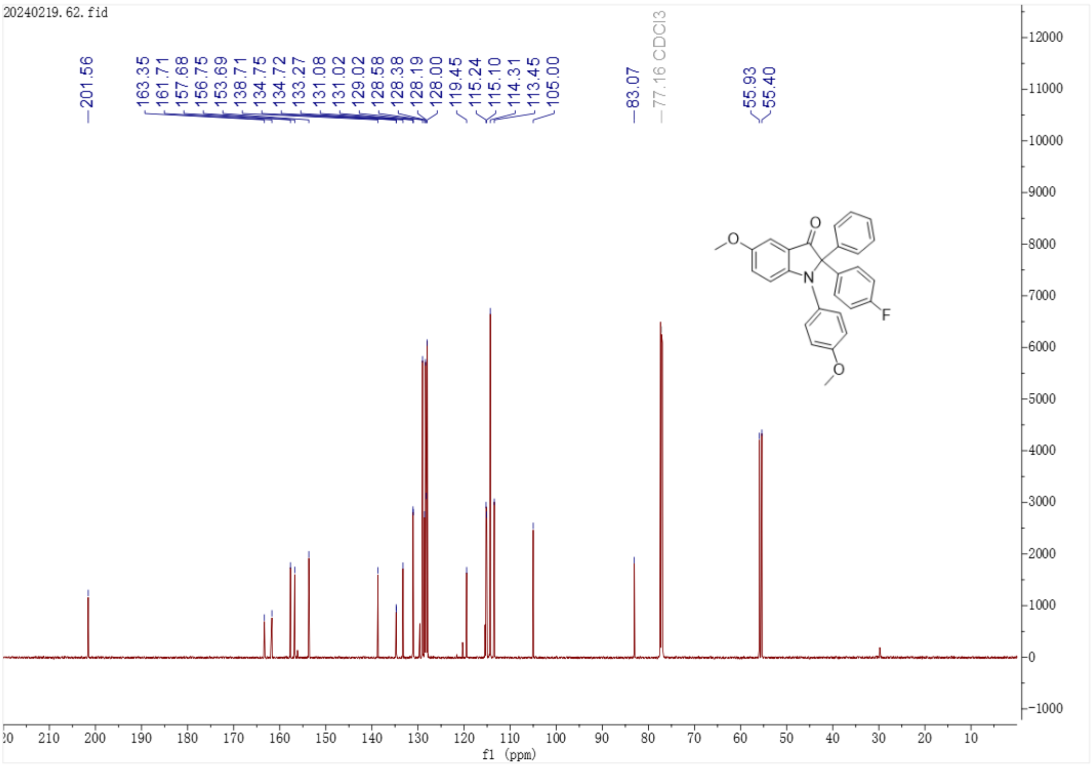

^{19}F NMR of compound **8b** (in $CDCl_3$)

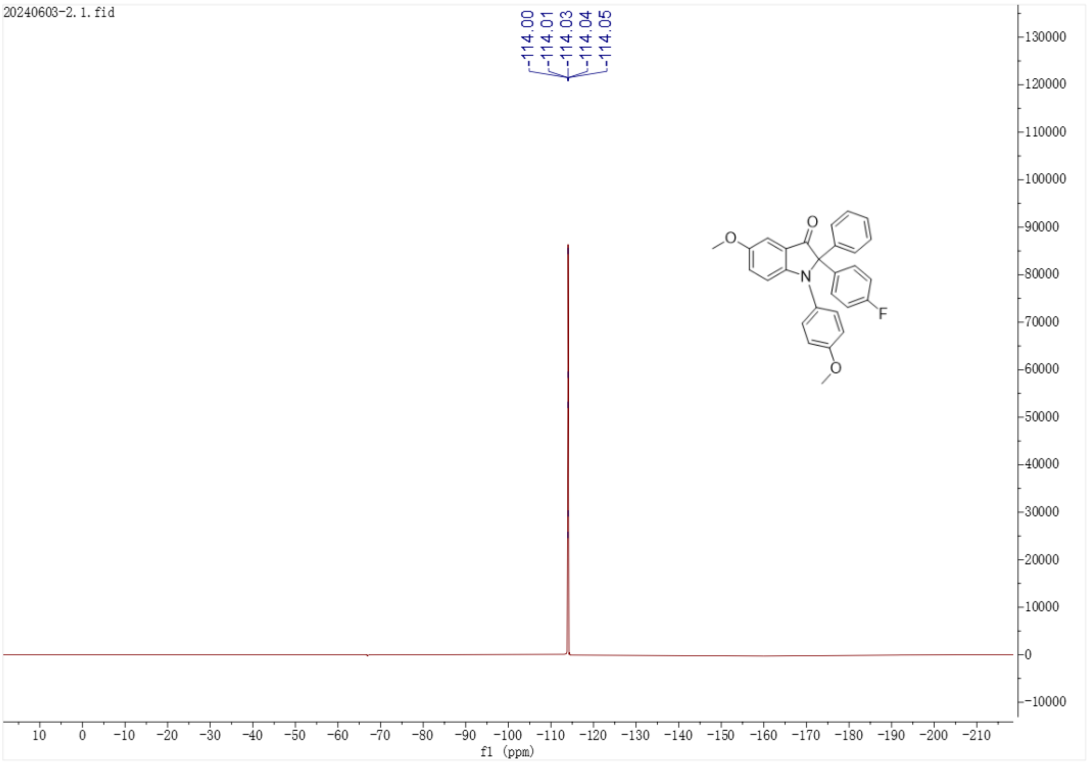

^{1}H NMR of compound **8c** (in $CDCl_3$)

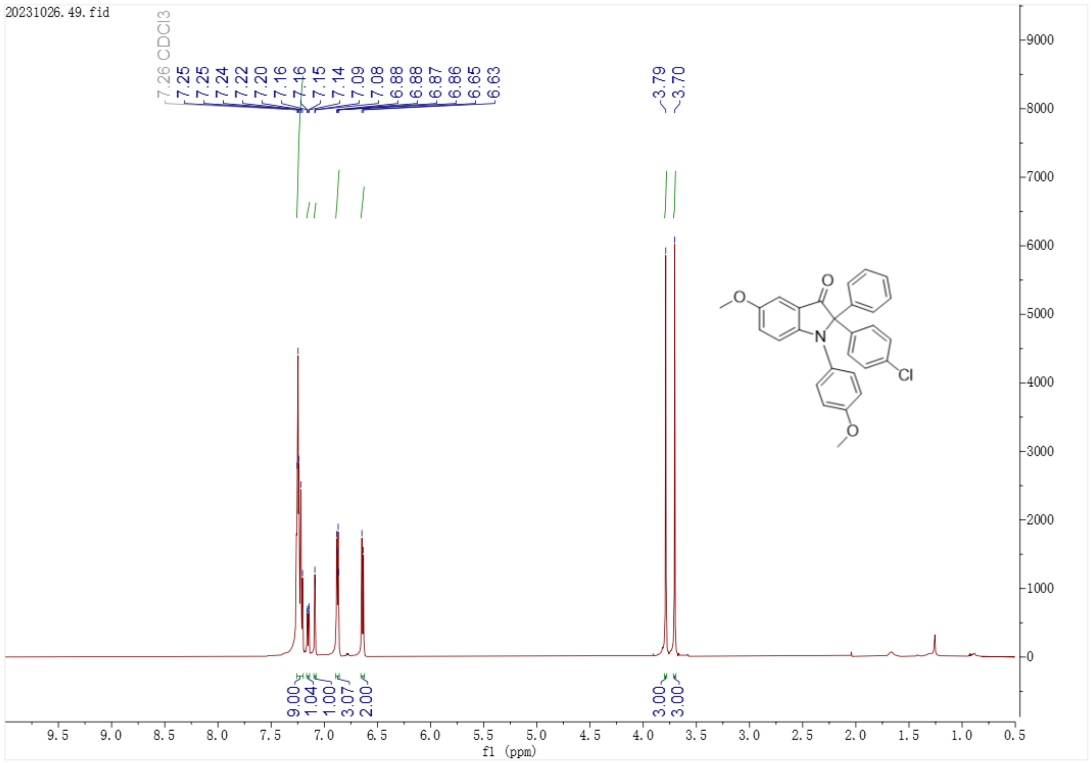

^{13}C NMR of compound **8c** (in $CDCl_3$)

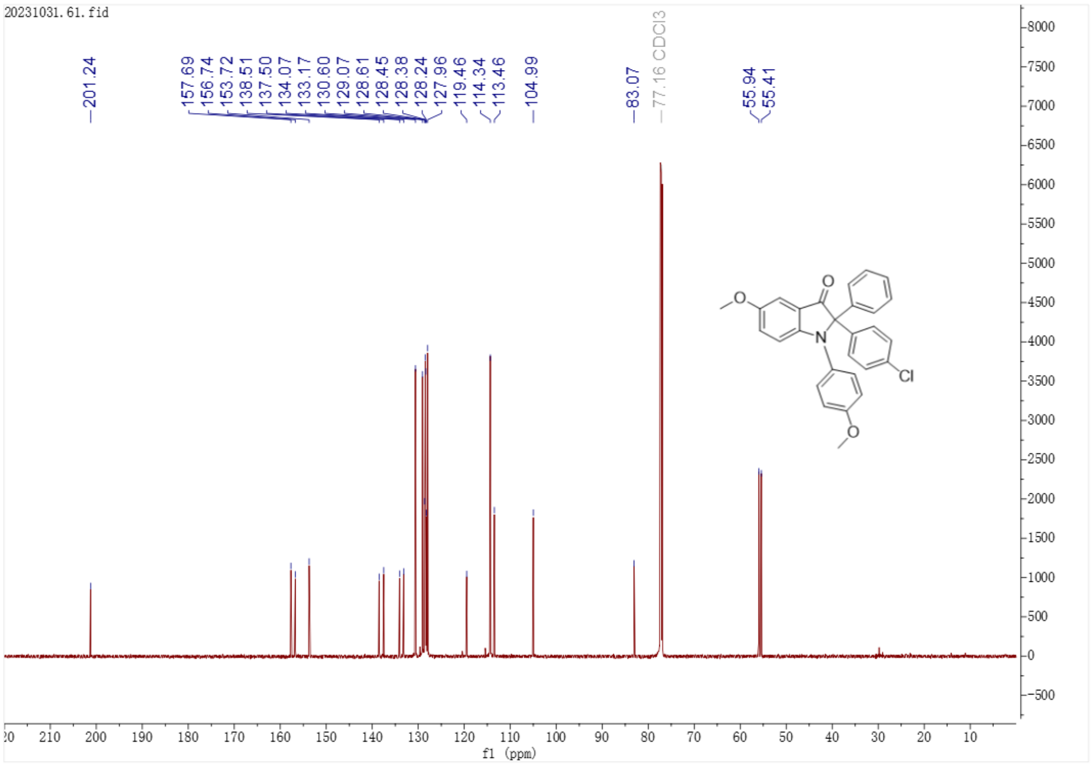

^{1}H NMR of compound **8d** (in CDCl3)

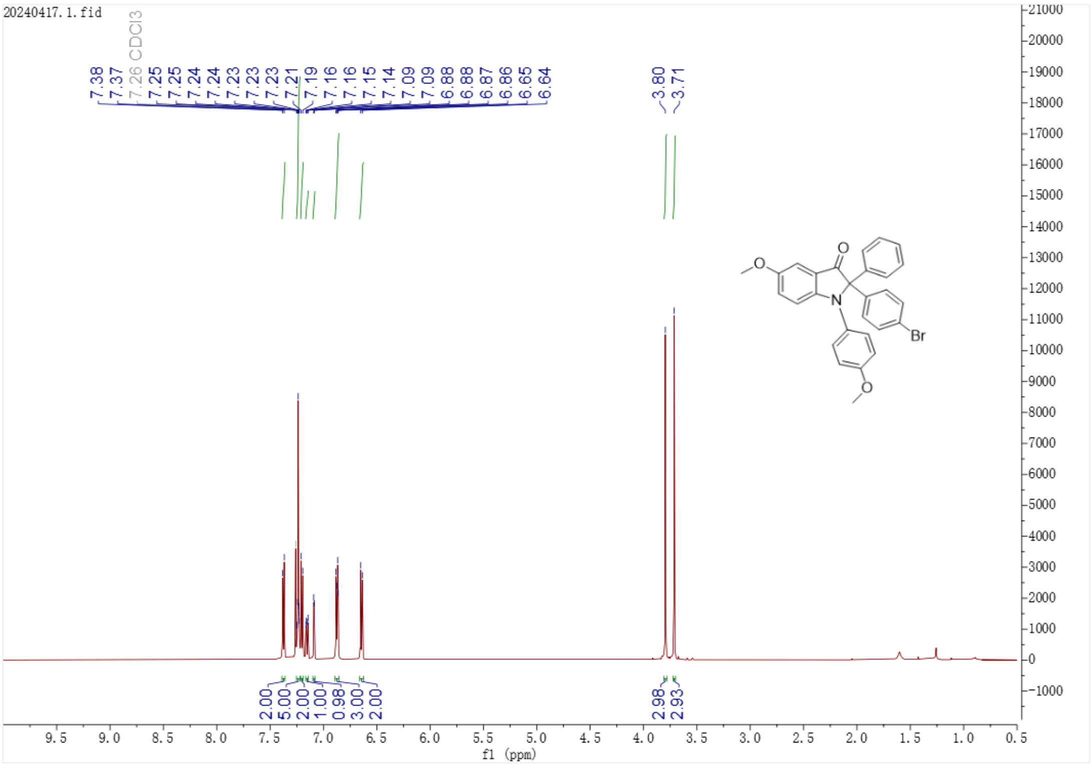

^{13}C NMR of compound **8d** (in CDCl3)

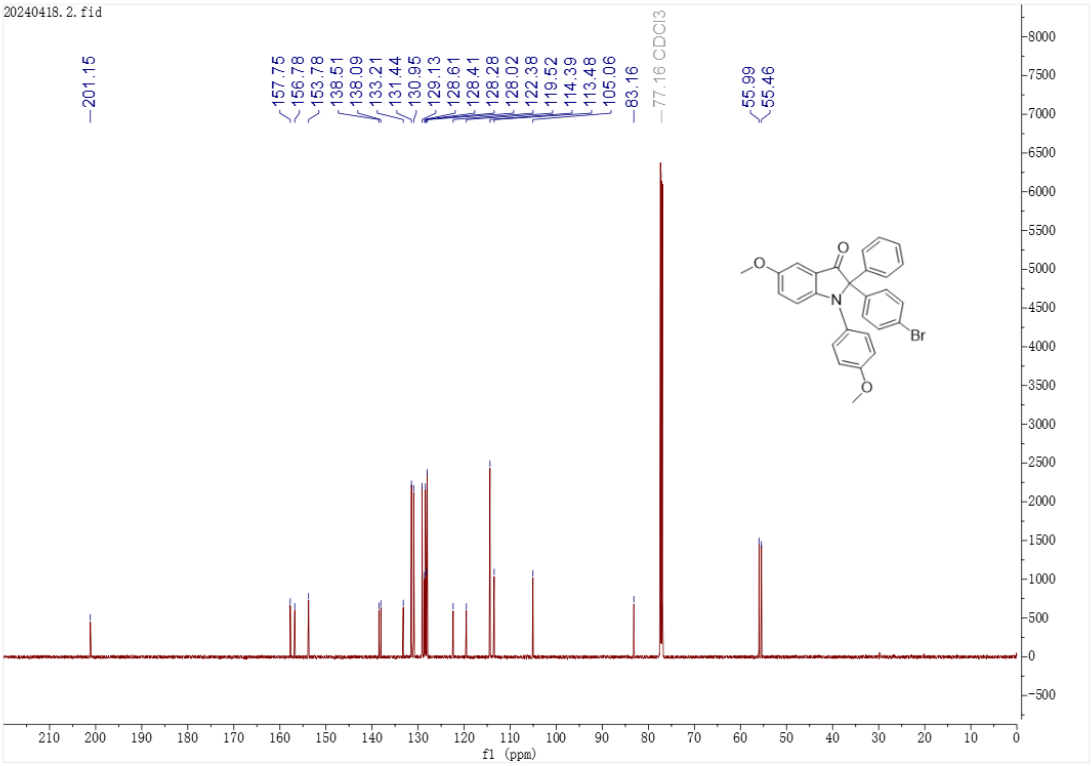

^{1}H NMR of compound **8e** (in $CDCl_3$)

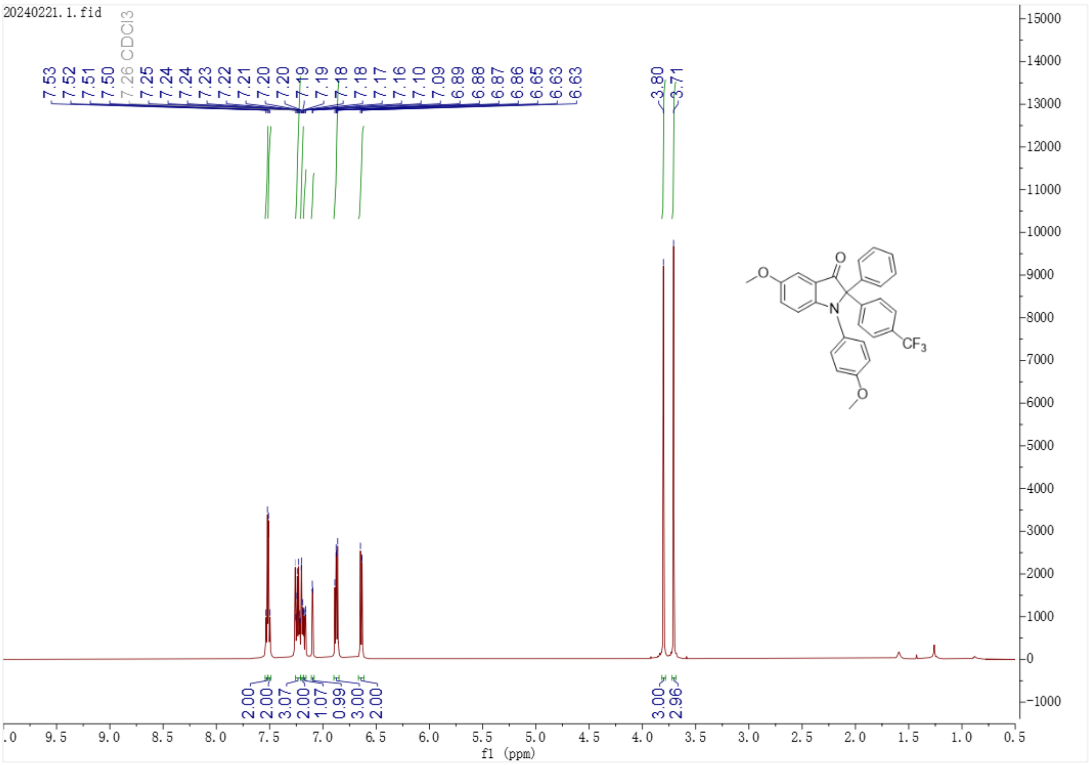

^{13}C NMR of compound **8e** (in $CDCl_3$)

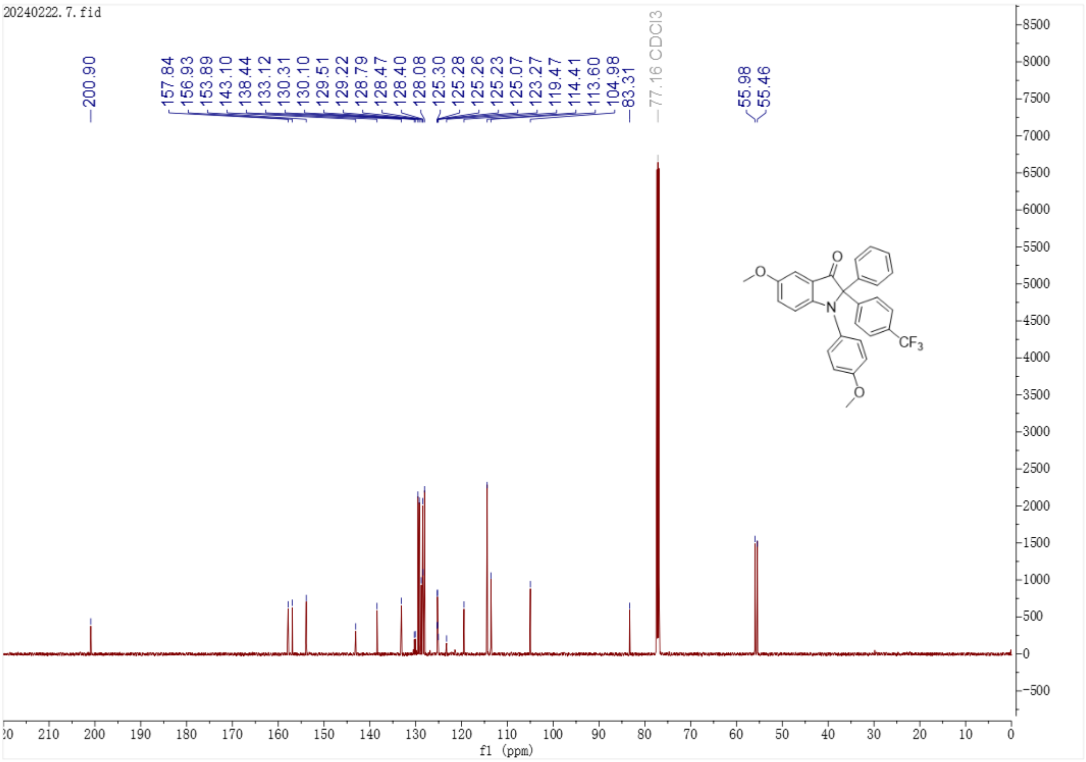

^{19}F NMR of compound **8e** (in $CDCl_3$)

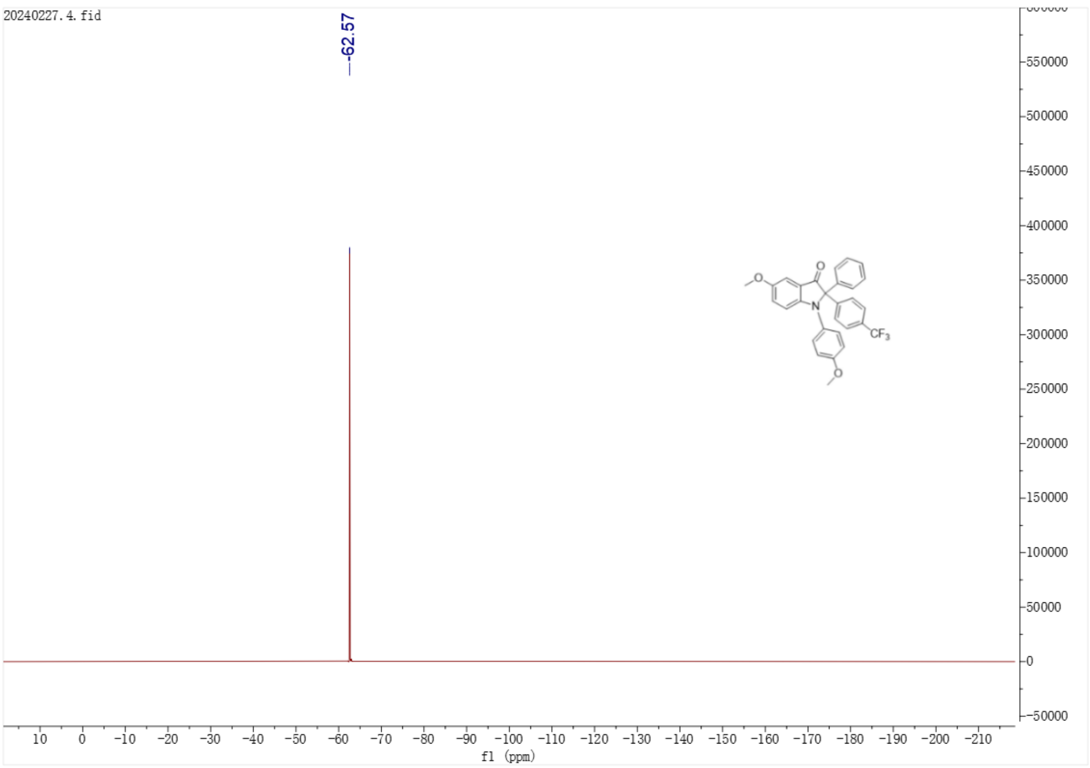

^{1}H NMR of compound **8f** (in $CDCl_3$)

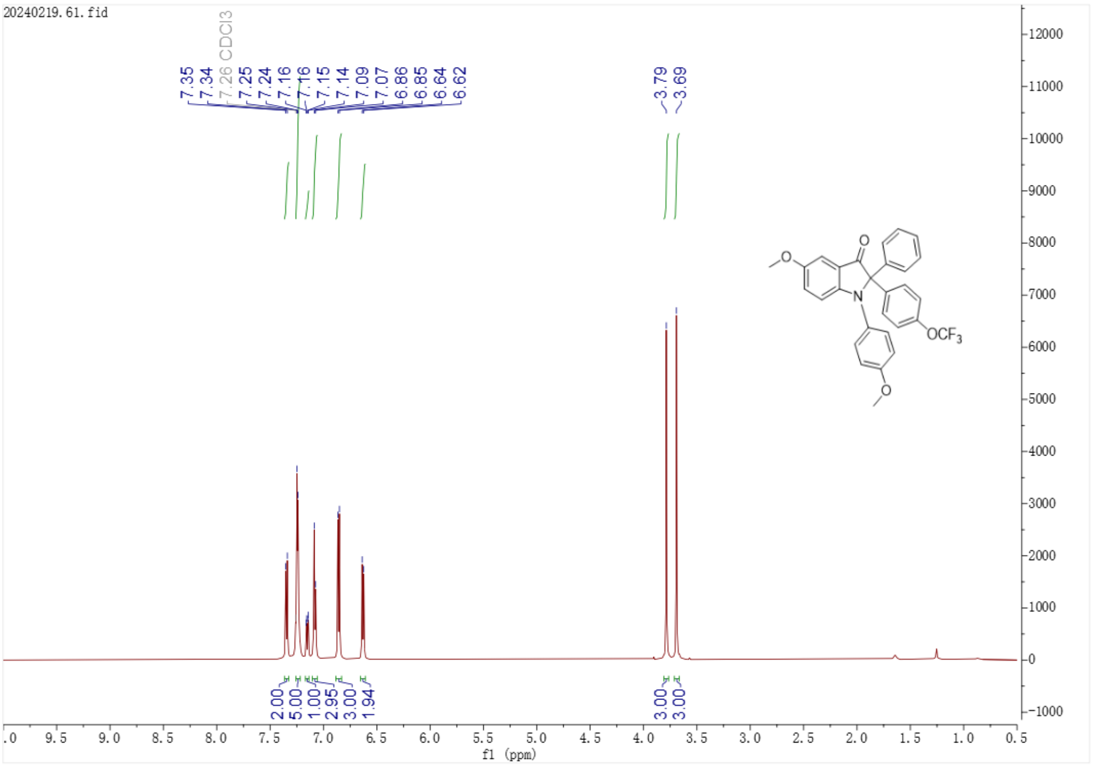

^{13}C NMR of compound **8f** (in $CDCl_3$)

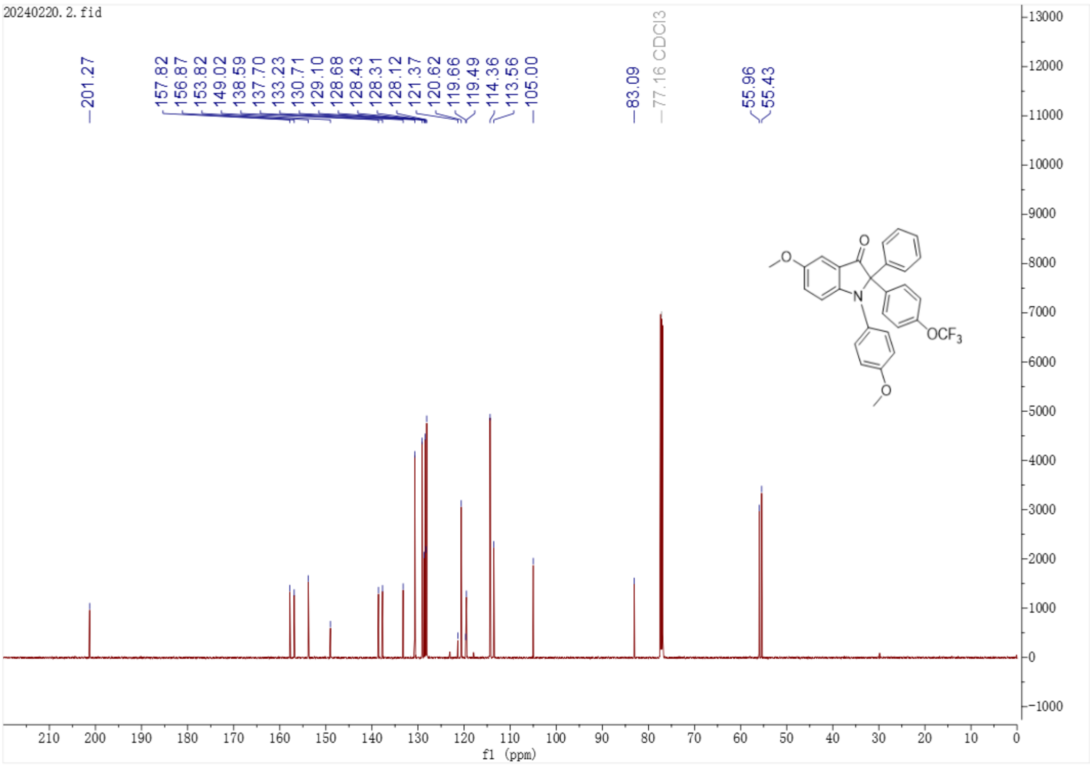

^{19}F NMR of compound **8f** (in $CDCl_3$)

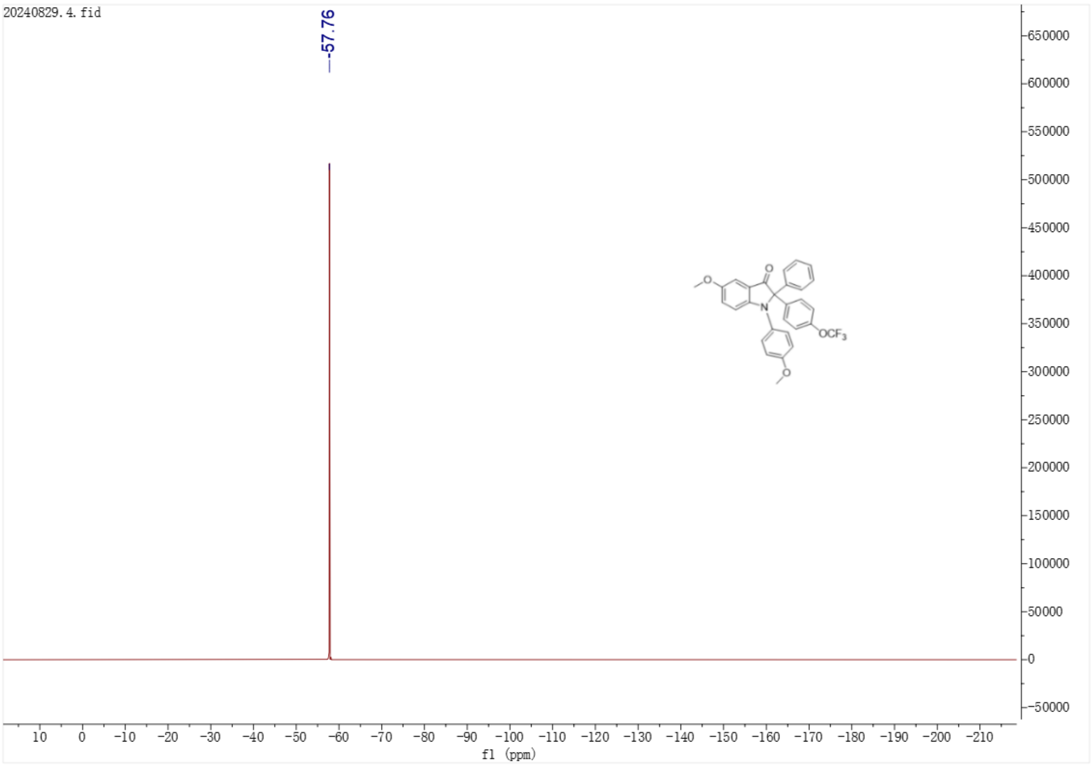

^{1}H NMR of compound **8g** (in $CDCl_3$)

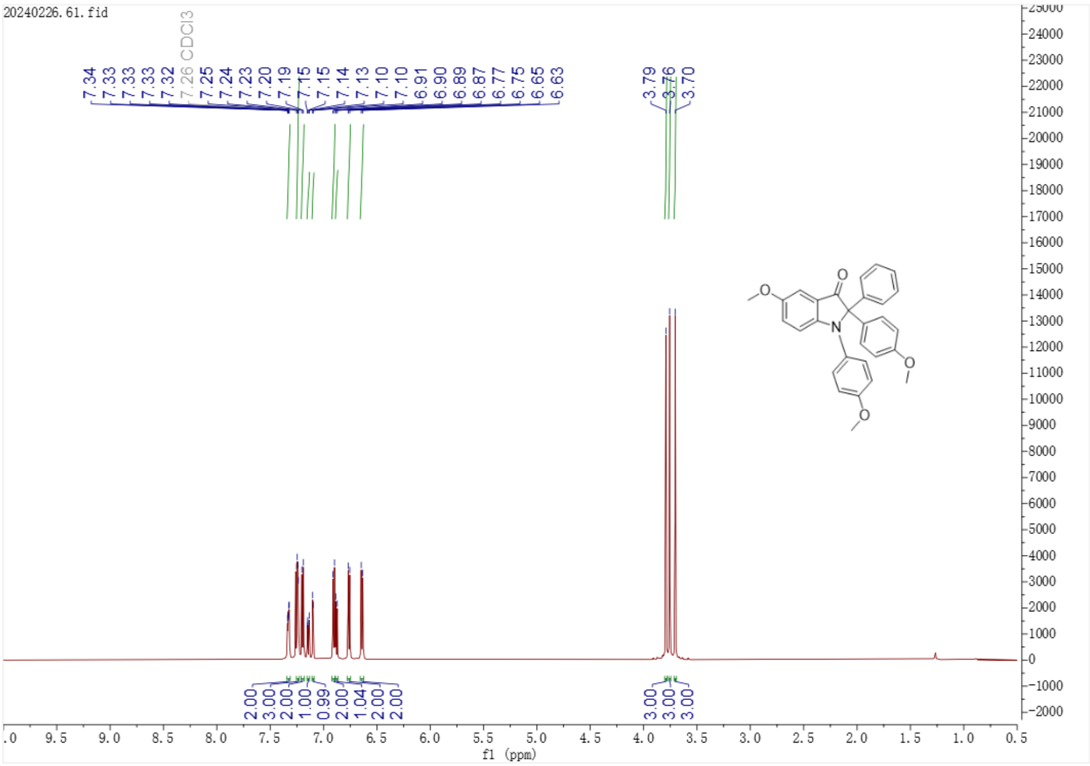

^{13}C NMR of compound **8g** (in $CDCl_3$)

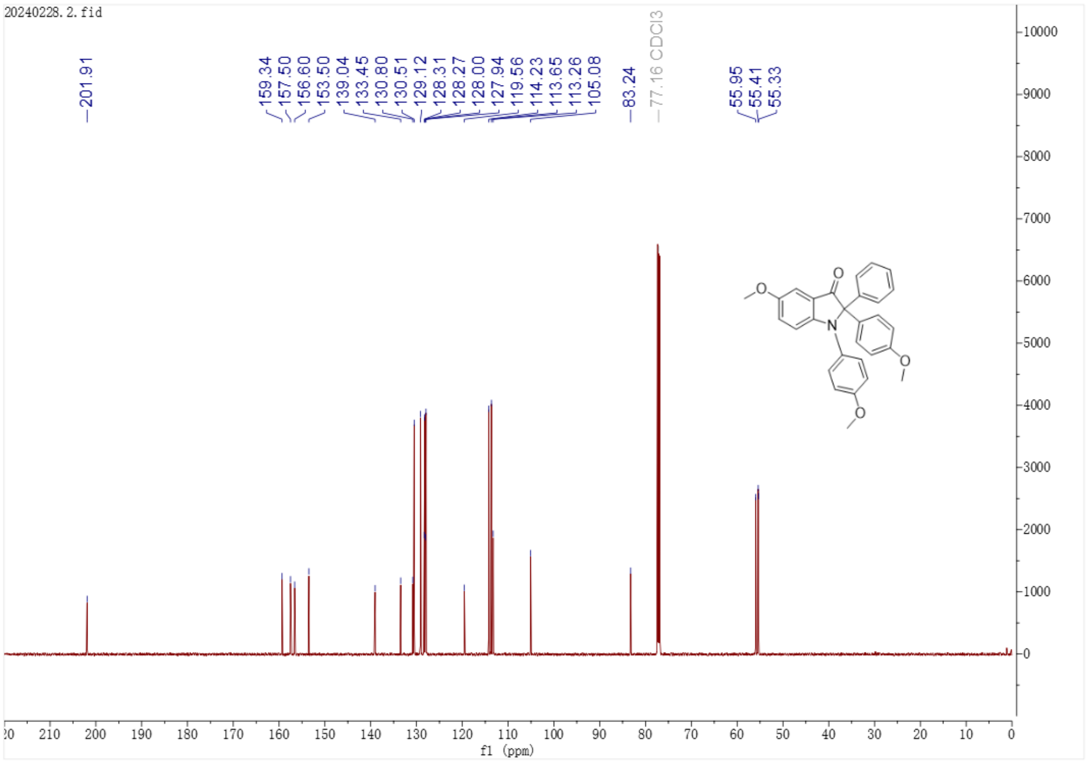

^{1}H NMR of compound **8h** (in $CDCl_3$)

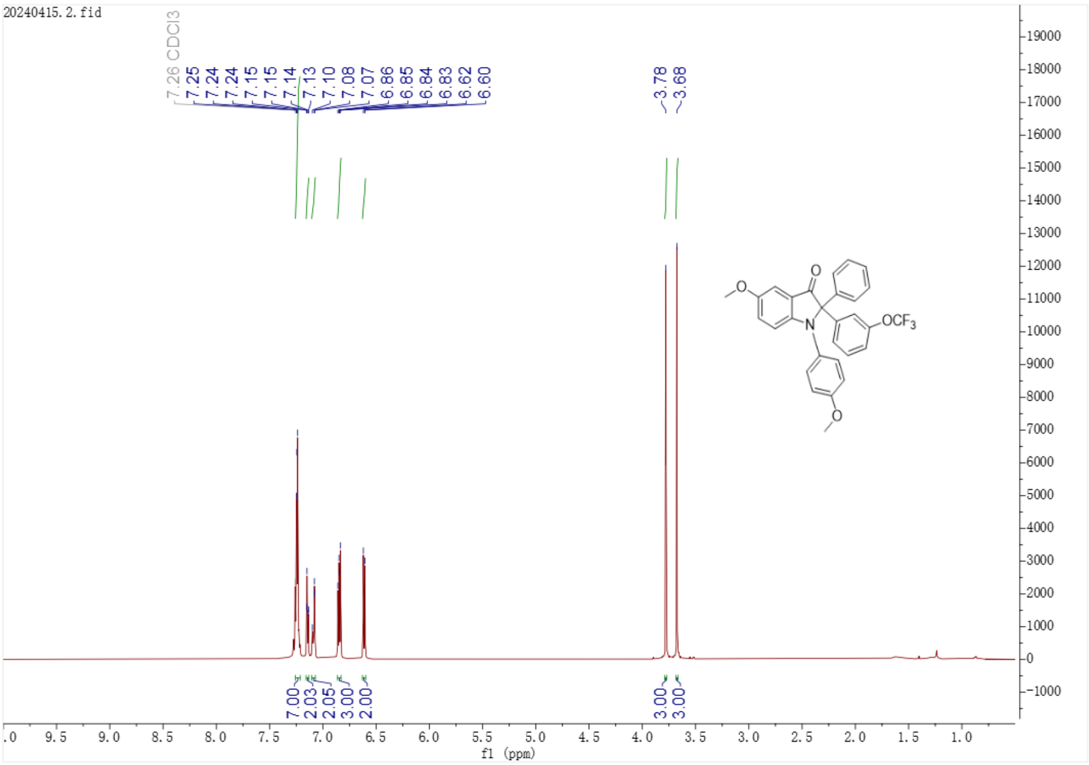

^{13}C NMR of compound **8h** (in $CDCl_3$)

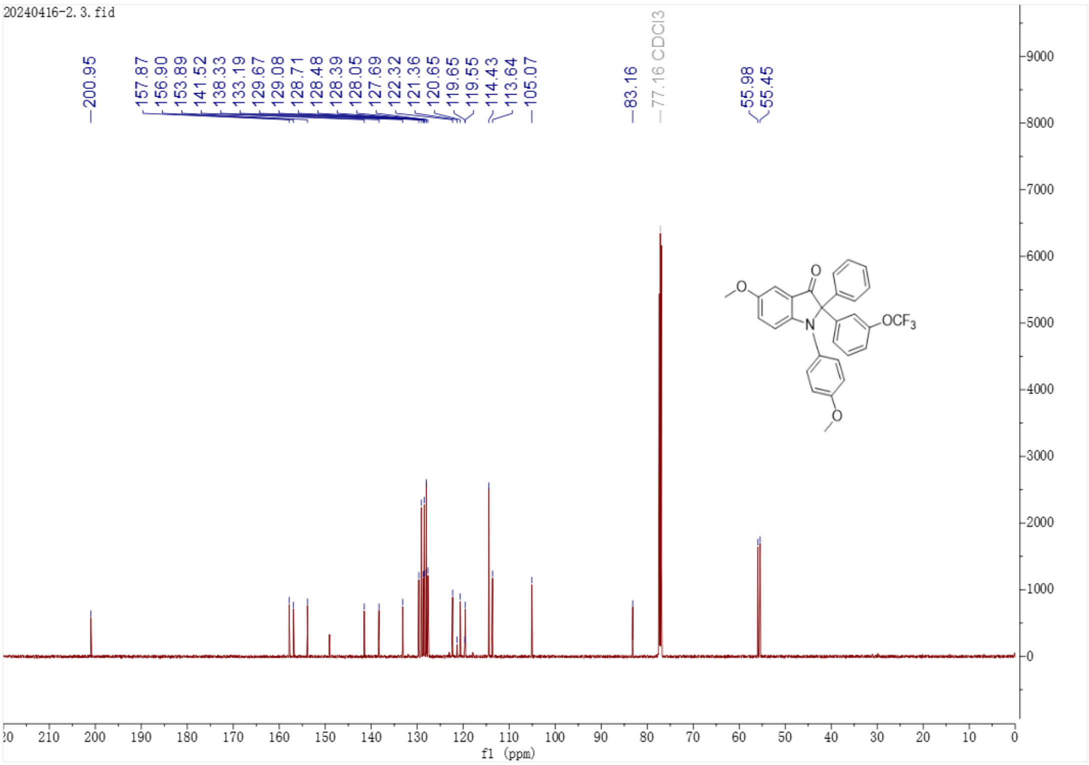

^{19}F NMR of compound **8h** (in $CDCl_3$)

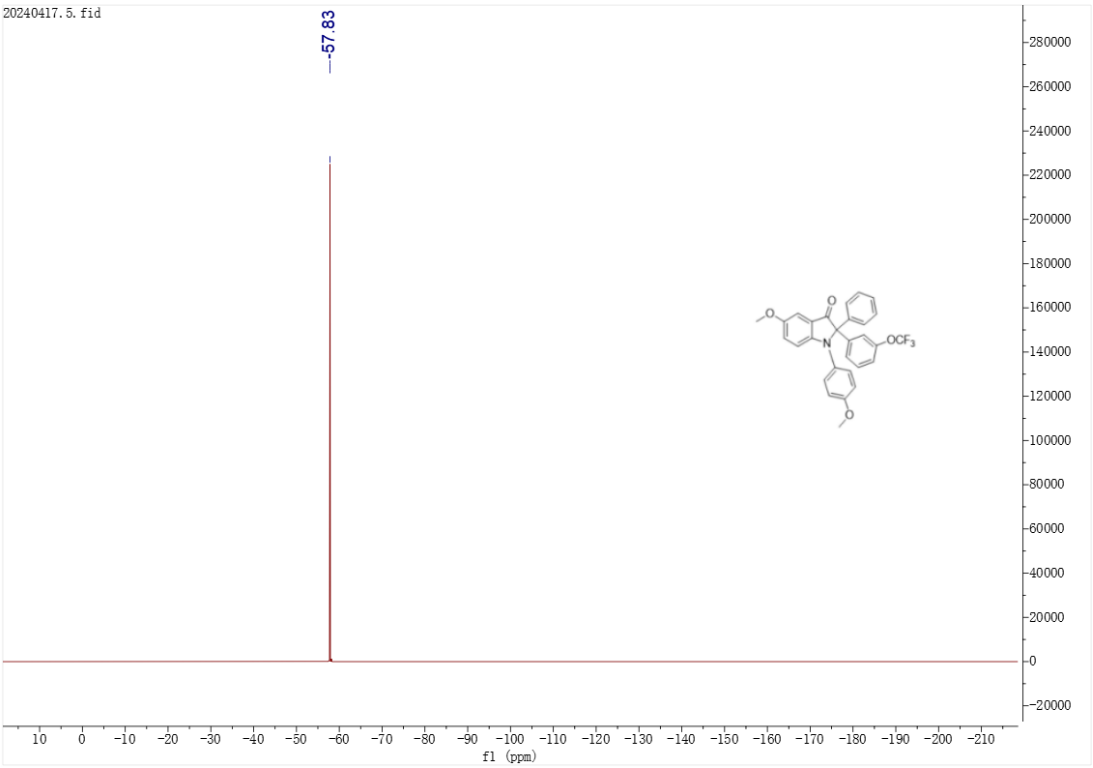

^{1}H NMR of compound **8i** (in DMSO-d_6)

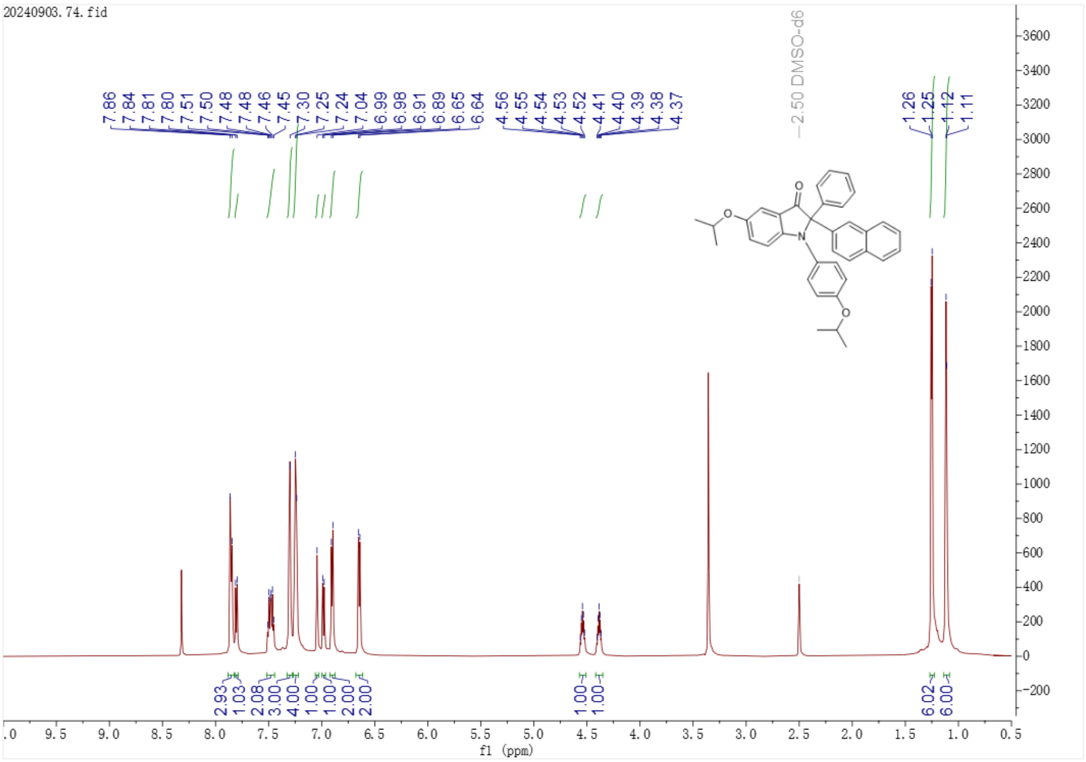

^{13}C NMR of compound **8i** (in $CDCl_3$)

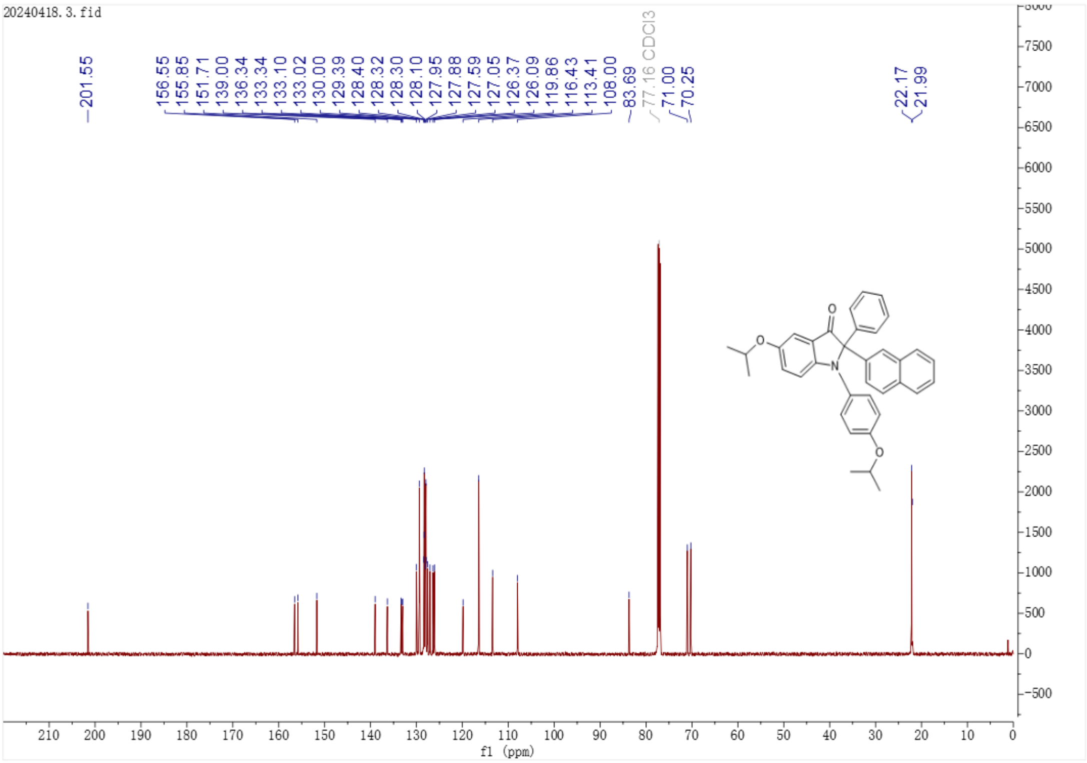

^{1}H NMR of compound **8j** (in DMSO-d_6)

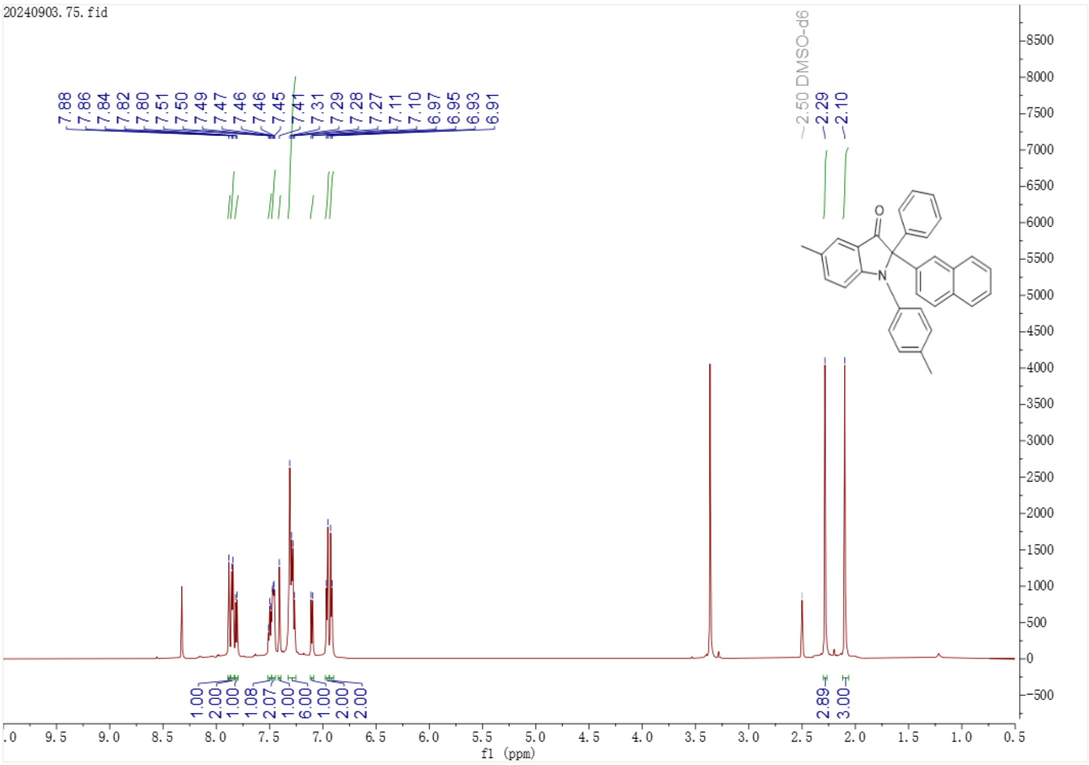

^{13}C NMR of compound **8j** (in $CDCl_3$)

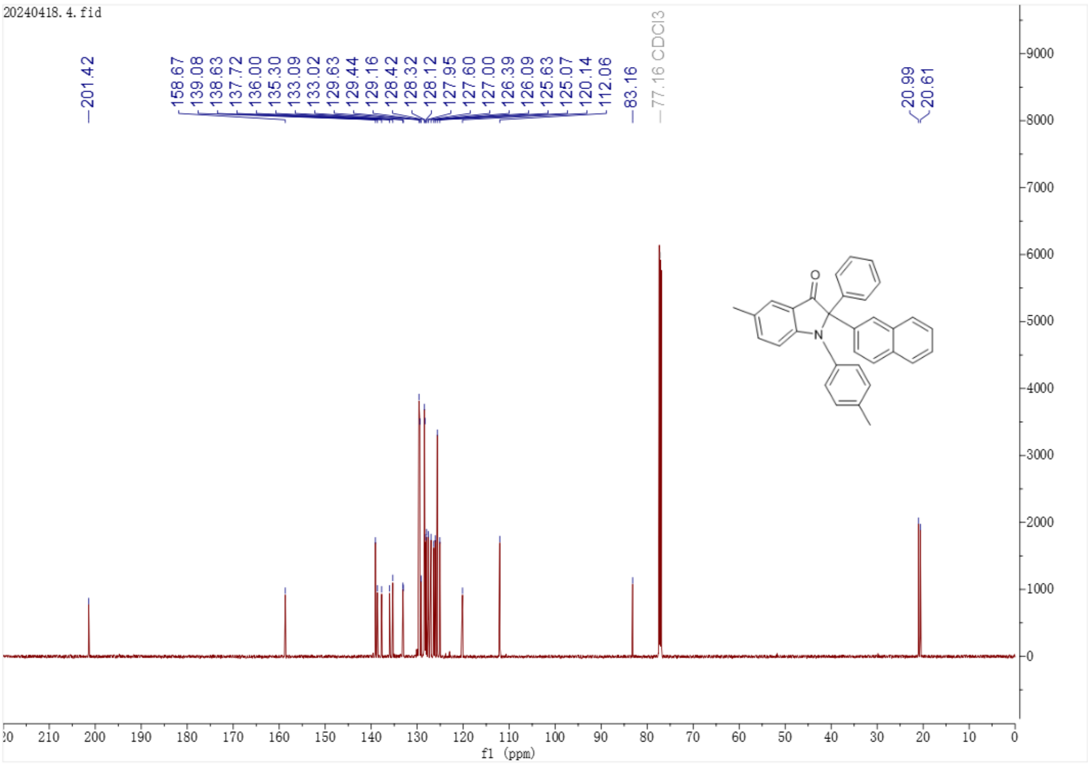

^{1}H NMR of compound **8k** (in $CDCl_3$)

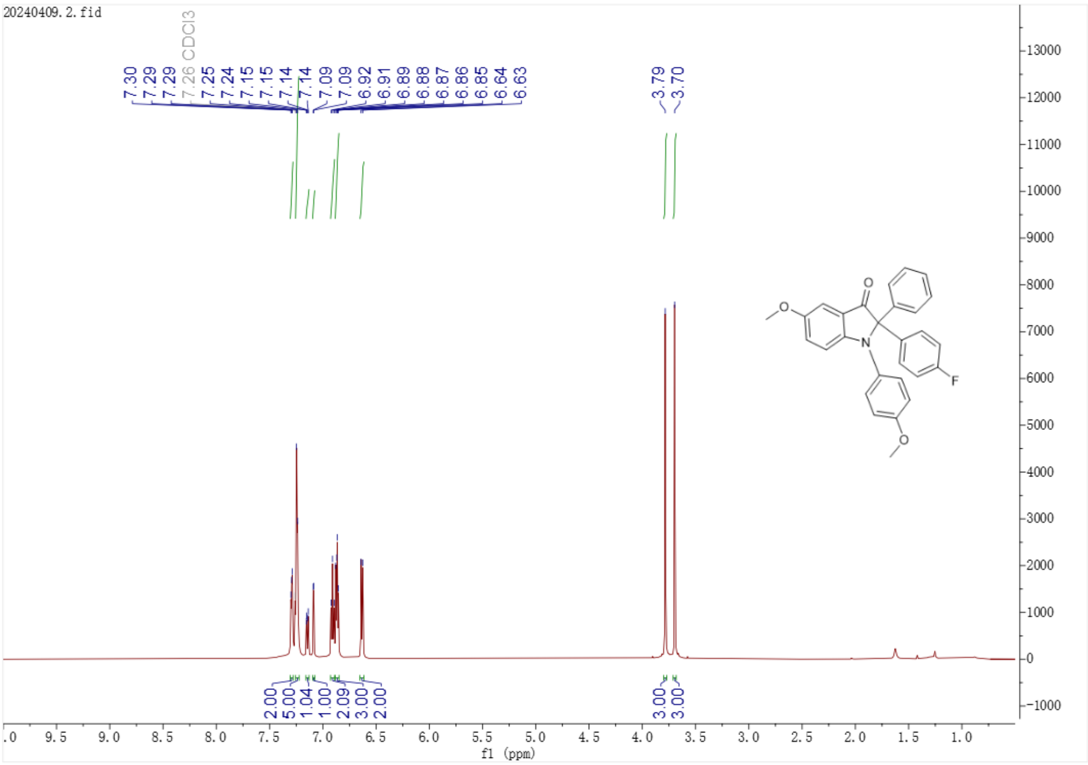

^{13}C NMR of compound **8k** (in $CDCl_3$)

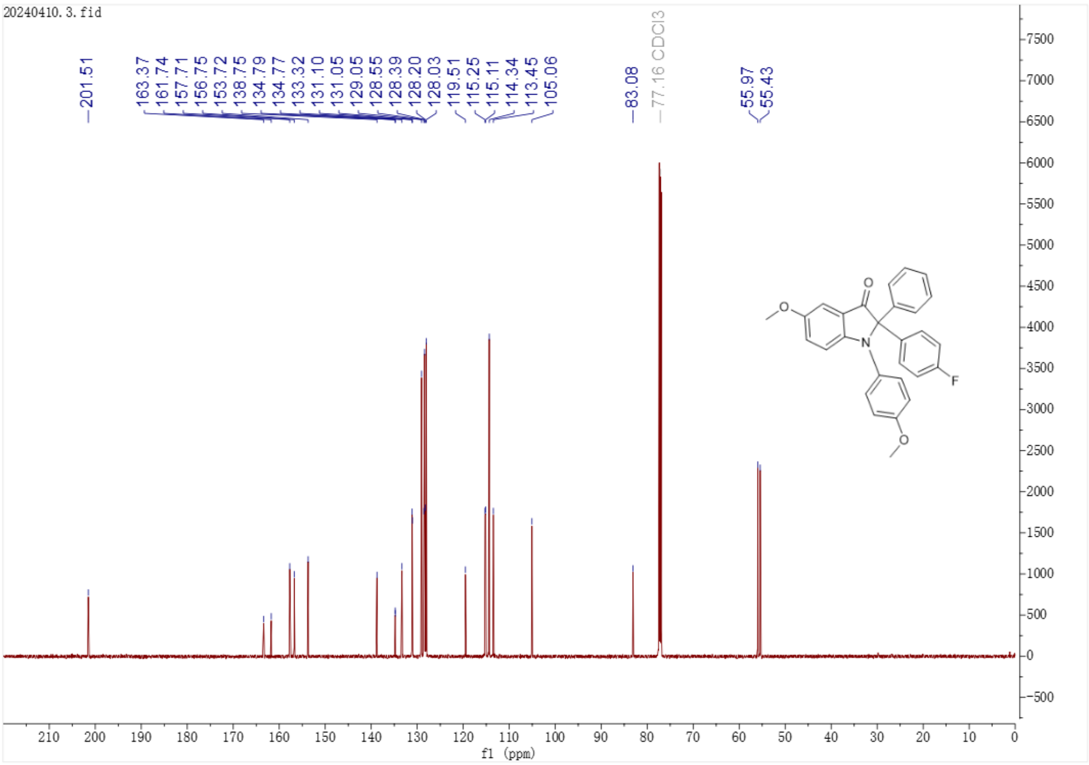

^{19}F NMR of compound **8k** (in $CDCl_3$)

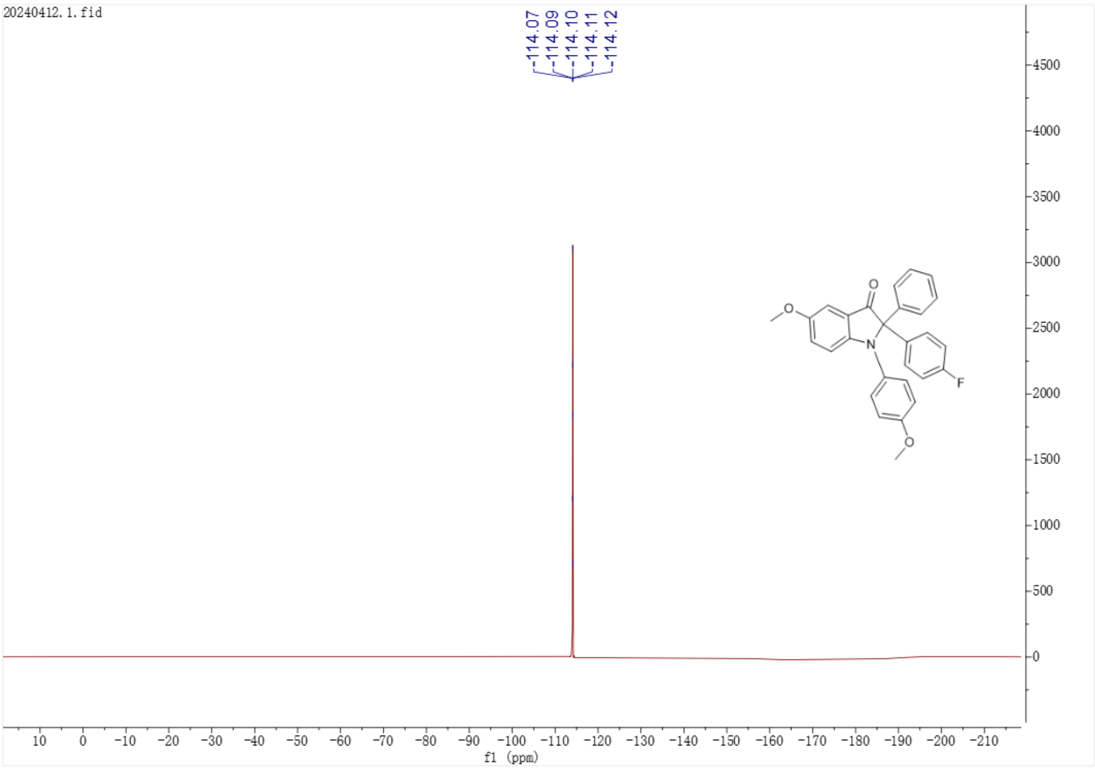

^{1}H NMR of compound **8l** (in $CDCl_3$)

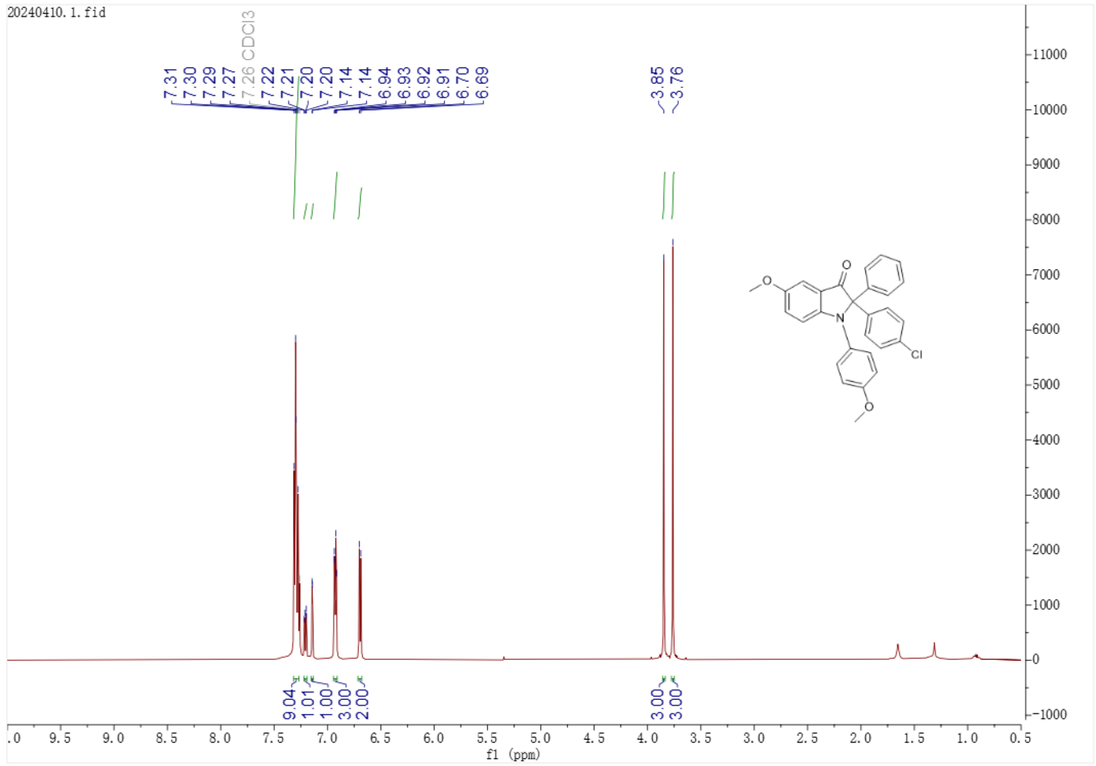

^{13}C NMR of compound **8l** (in $CDCl_3$)

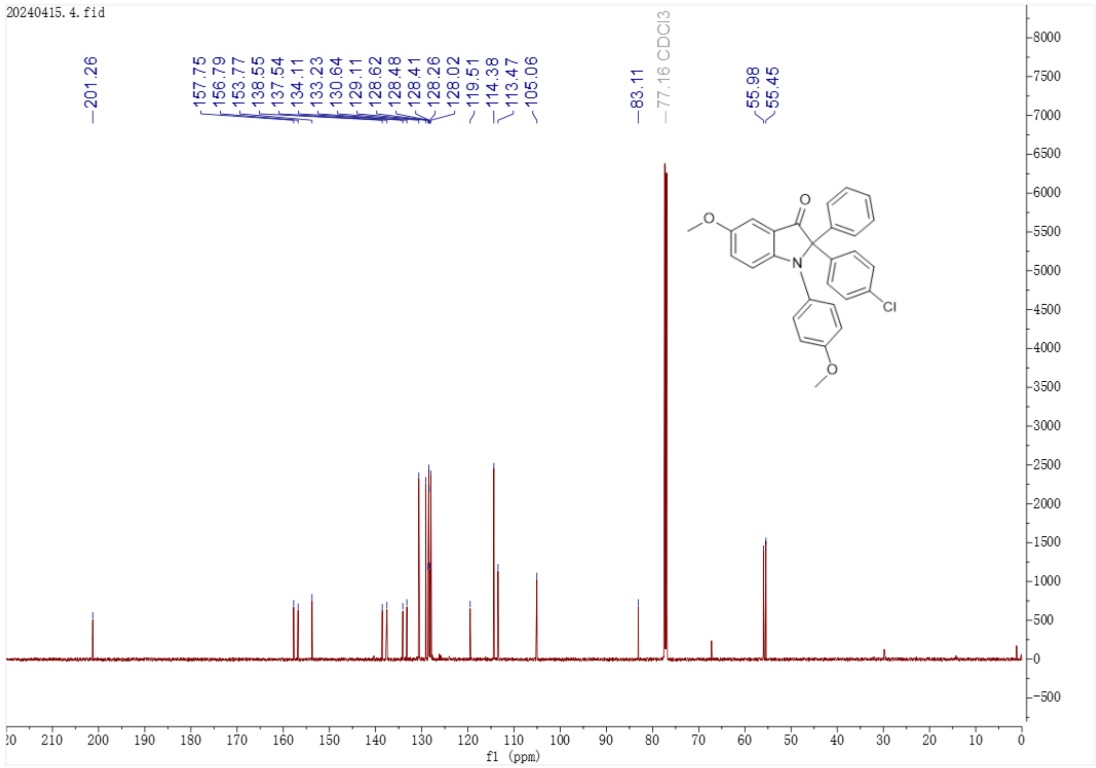

^{1}H NMR of compound **8m** (in $CDCl_3$)

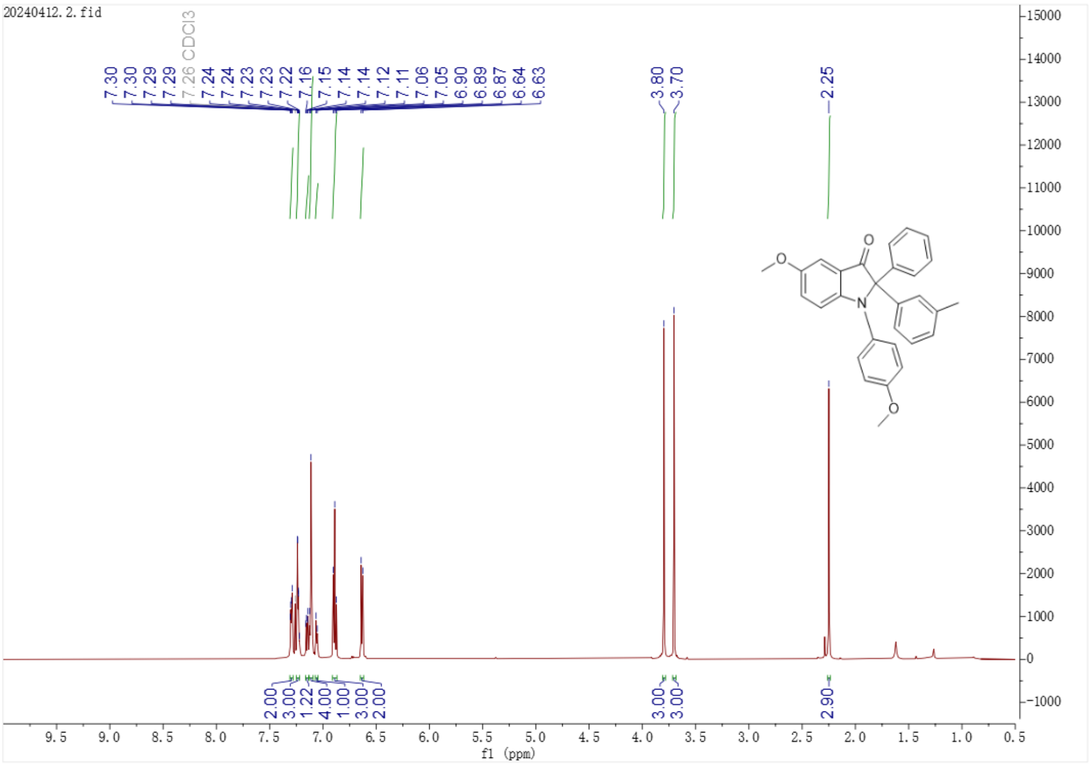

^{13}C NMR of compound **8m** (in $CDCl_3$)

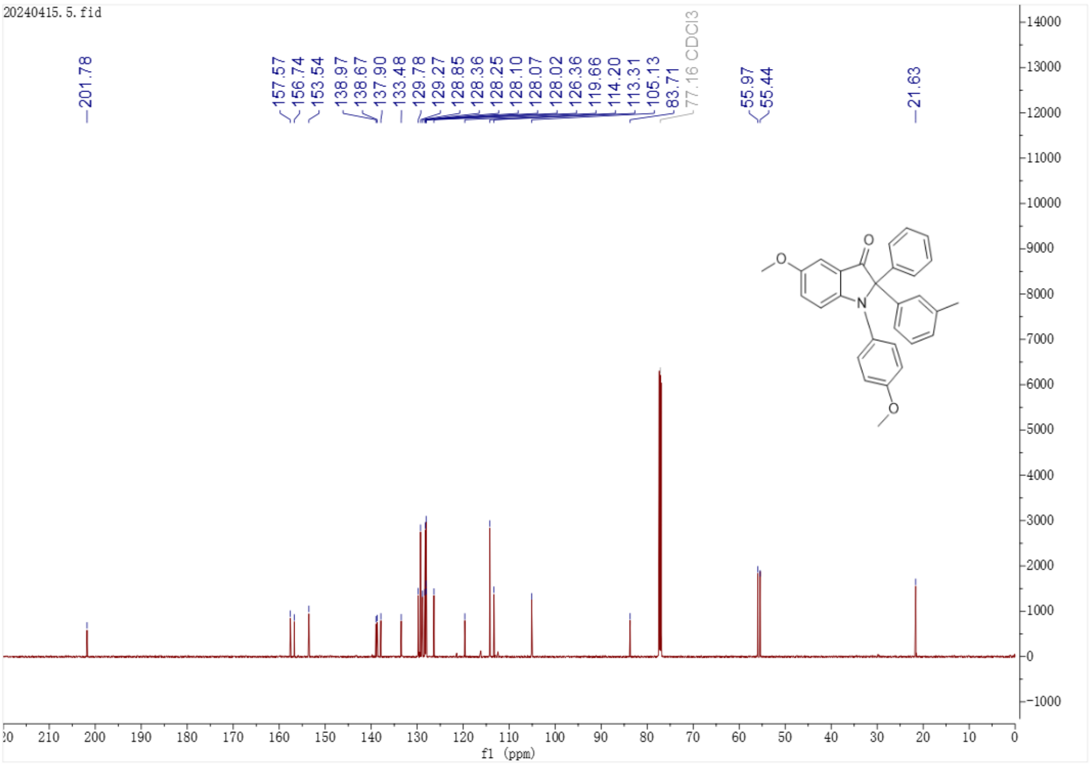

^{1}H NMR of compound **8n** (in $CDCl_3$)

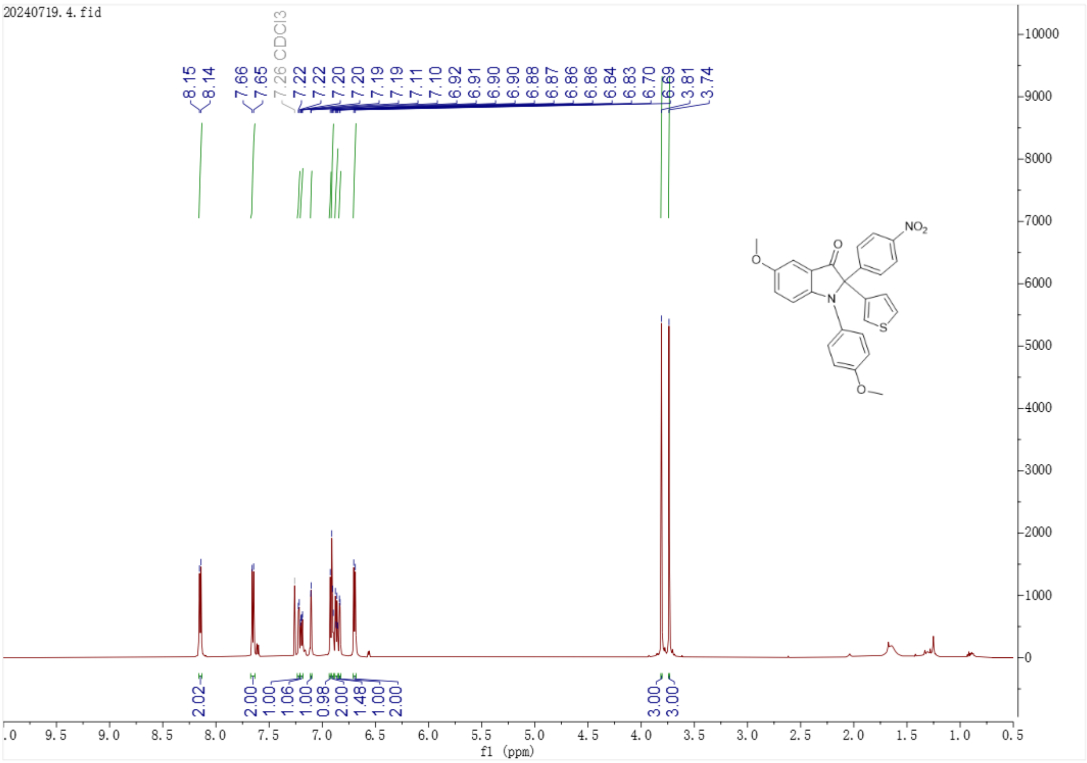

^{13}C NMR of compound **8n** (in $CDCl_3$)

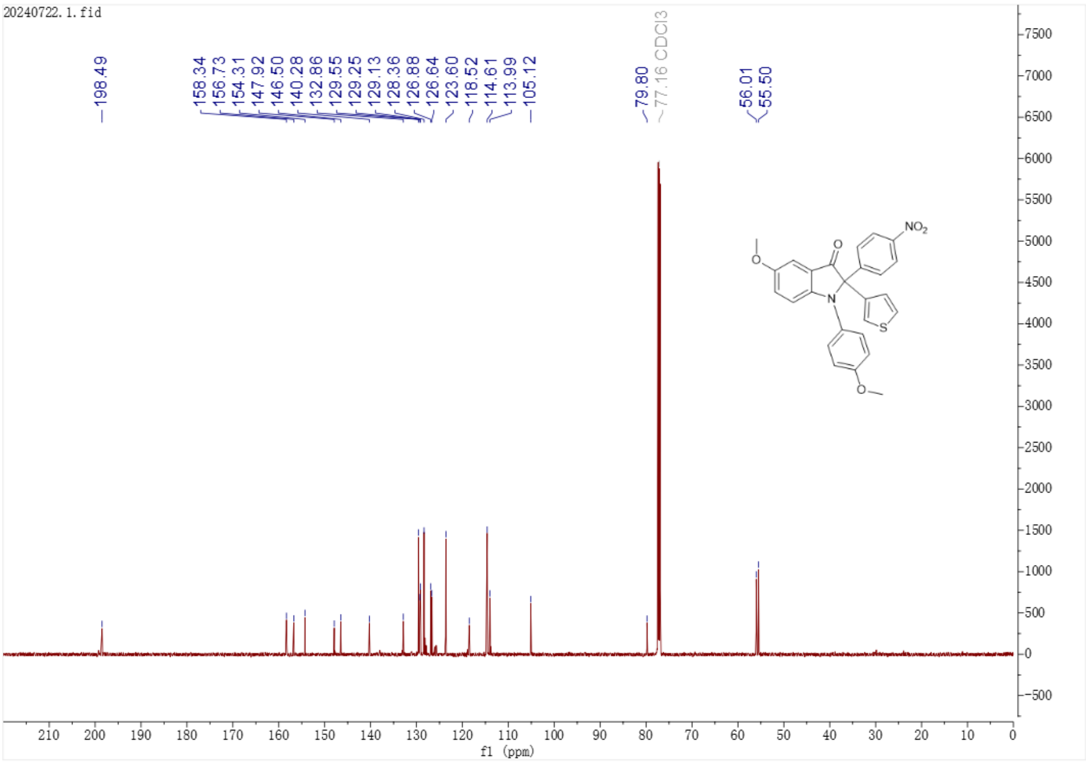

^{1}H NMR of compound **8o** (in $CDCl_3$)

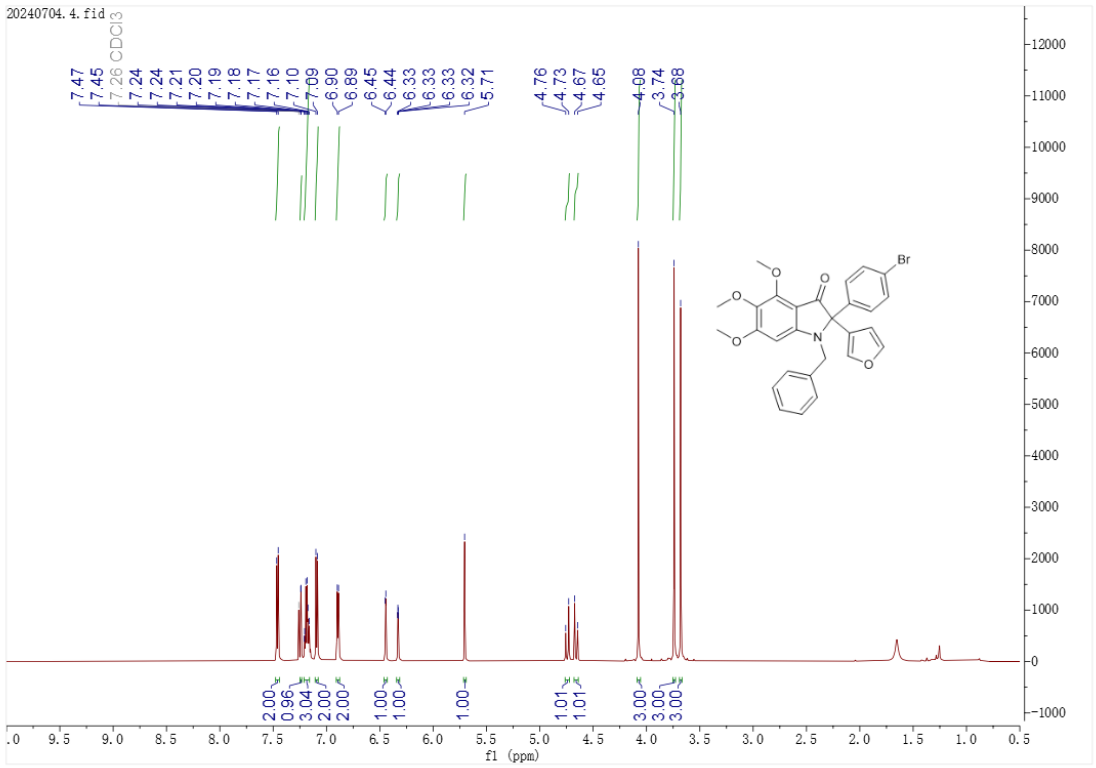

^{13}C NMR of compound **8o** (in $CDCl_3$)

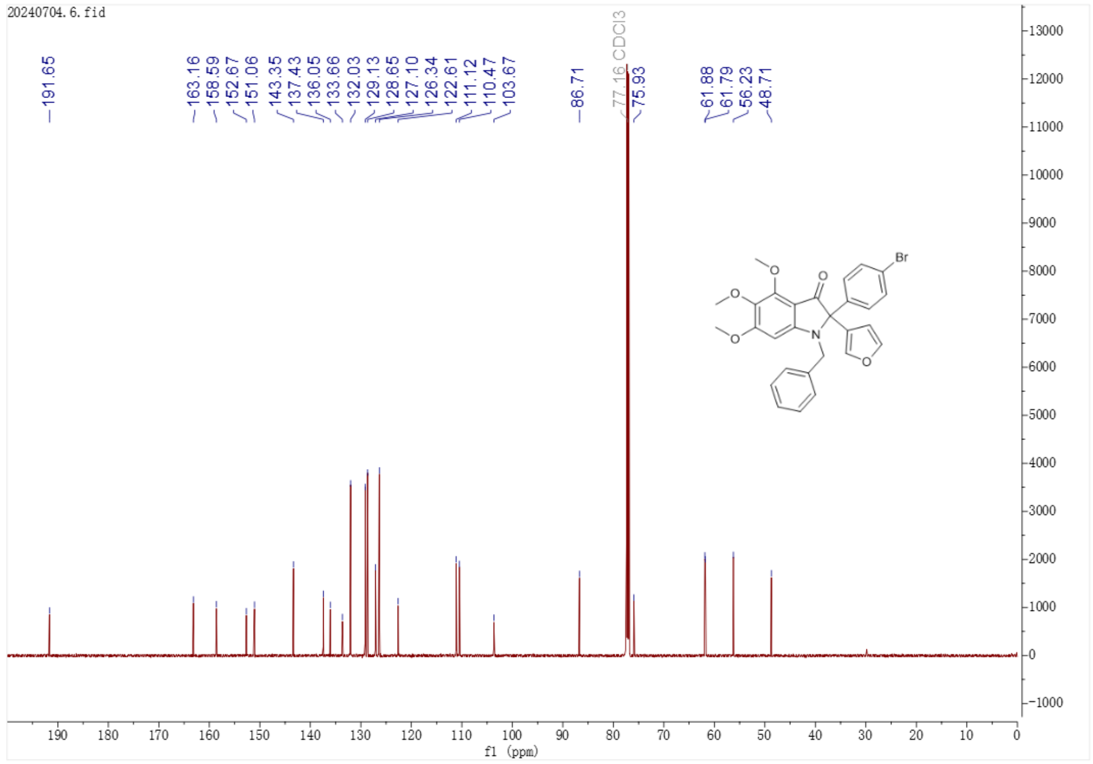

12. Crystallographic data and molecular structure of 4i and 8d

Datablock: 1_a

Bond precision:	C-C = 0.0047 A		Wavelength=1.54178
Cell:	a=9.1466(14)	b=21.345(3)	c=24.514(4)
	alpha=90	beta=90	gamma=90
Temperature:	273 K		

	Calculated	Reported
Volume	4786.0(13)	4785.9(12)
Space group	P b c a	P b c a
Hall group	-P 2ac 2ab	-P 2ac 2ab
Moiety formula	C28 H21 Br N2 O4	C28 H21 Br N2 O4
Sum formula	C28 H21 Br N2 O4	C28 H21 Br N2 O4
Mr	529.37	529.38
Dx,g cm-3	1.469	1.469
Z	8	8
Mu (mm-1)	2.657	2.657
F000	2160.0	2160.0
F000'	2160.41	
h,k,lmax	10,25,29	10,25,29
Nref	4265	4237
Tmin,Tmax	0.585,0.620	0.458,0.753
Tmin'	0.531	

Correction method= # Reported T Limits: Tmin=0.458 Tmax=0.753
AbsCorr = NONE

Data completeness= 0.993 Theta(max)= 66.954

R(reflections)= 0.0591(3828) wR2(reflections)= 0.1764(4237)

S = 1.062 Npar= 318

Datablock 1_a - ellipsoid plot

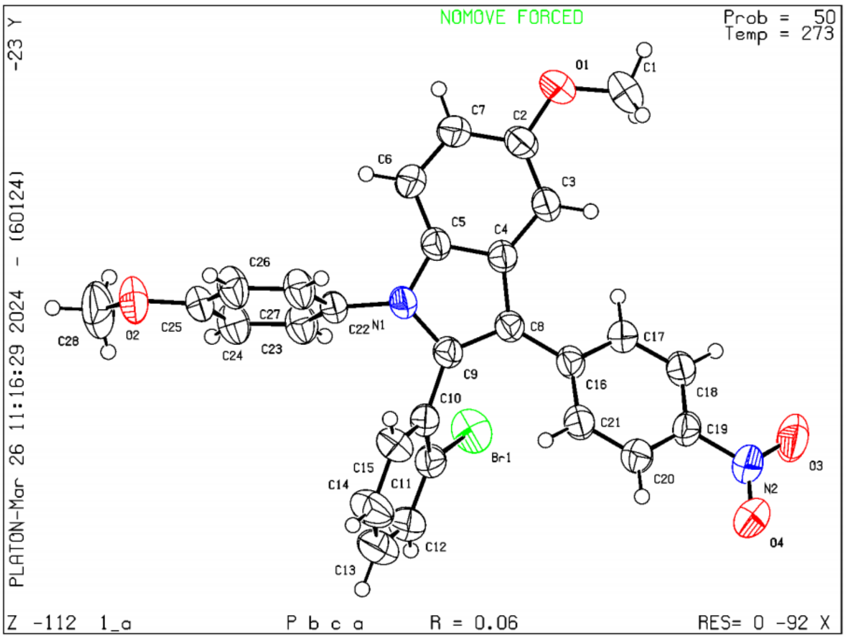

Structure of **4i**

Datablock: 1_a

Bond precision:	C-C = 0.0114 A		Wavelength=1.54178
Cell:	a=8.37600	b=9.69800	c=16.17600
	alpha=97.3600	beta=99.9400	gamma=112.7100
Temperature:	273 K		

	Calculated	Reported
Volume	1166.172	1166
Space group	P -1	P -1
Hall group	-P 1	-P 1
Moiety formula	C28 H22 Br N O3	C28 H22 Br N O3
Sum formula	C28 H22 Br N O3	C28 H22 Br N O3
Mr	500.37	500.37
Dx,g cm-3	1.425	1.425
Z	2	2
Mu (mm-1)	2.644	2.644
F000	512.0	512.0
F000'	511.95	
h,k,lmax	9,11,19	0,0,0
Nref	4131	4027
Tmin,Tmax	0.765,0.809	0.864,0.864
Tmin'	0.693	

Correction method= # Reported T Limits: Tmin=0.864 Tmax=0.864
AbsCorr = MULTI-SCAN

Data completeness= 0.975 Theta(max)= 66.837

R(reflections)= 0.1411(2249) wR2(reflections)= 0.3435(4027)

S = 0.999 Npar= 252

Datablock 1_a - ellipsoid plot

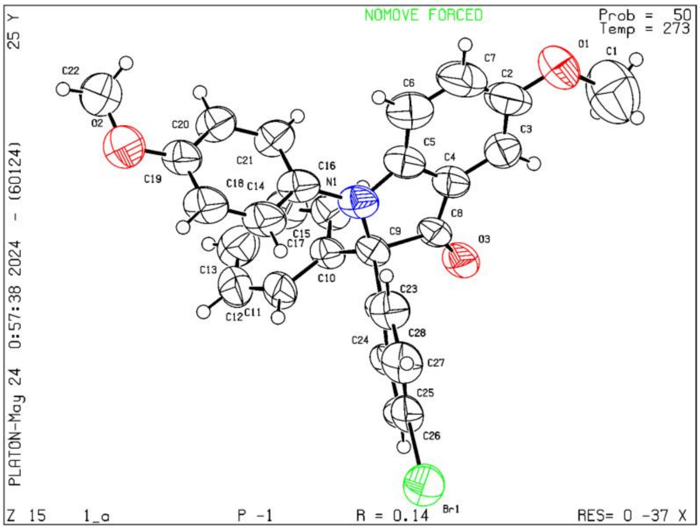

Structure of **8d**
