# Supplementary material for: Intercepting an avoided α-iminol rearrangement with a Petasis reaction for the synthesis of 2,3-diaryl substituted indoles
Source: Commun Chem. 2025 May 15;8:152. doi: 10.1038/s42004-025-01528-9 (PMC12081858; doi:10.1038/s42004-025-01528-9)
Supplement: Supplementary file 3 — Description of Additional Supplementary Files [file 42004_2025_1528_MOESM3_ESM.pdf]

Description of Additional Supplementary Files

File name: Supplementary Data 1

Description: Cif for 4i

File name: Supplementary Data 2

Description: Cif for 8d
